# Treatment duration of complicated urinary tract infections by extended-spectrum beta-lactamases producing enterobacterales

**DOI:** 10.1371/journal.pone.0237365

**Published:** 2020-10-19

**Authors:** Judith Álvarez Otero, Jose Luis Lamas Ferreiro, Ana Sanjurjo Rivo, Fernando Maroto Piñeiro, Lucía González González, Ignacio Enríquez de Salamanca Holzinger, Jorge Cavero, Irene Rodríguez Conde, María Fernández Soneira, Javier de la Fuente Aguado

**Affiliations:** 1 Internal Medicine, Povisa Hospital, Vigo, Spain; 2 Preventive Medicine, Povisa Hospital, Vigo, Spain; 3 Microbiology, Povisa Hospital, Vigo, Spain; Vita Salute University of Milan, ITALY

## Abstract

**Background:**

Urinary tract infections caused by extended-spectrum beta-lactamase producing Enterobacterales (ESBL-EB) are a problem increasing in our clinical practice.

**Objectives:**

The aim of this study was to evaluate the clinical outcome in patients who received short (≤ 7 days) versus long courses (>7 days) of antimicrobial therapy for complicated ESBL-EB urinary tract infections.

**Methods:**

This is a retrospective and observational study. Positive urine cultures for ESBL-EB in our hospital between March 2015 and July 2017 were identified. Patients with complicated urinary tract infection were included. Differences between treatment groups (7 days or less vs more than 7 days) were analyzed according to baseline characteristics and severity of clinical presentation. Primary outcome was all cause 30-day mortality. Secondary outcome was a combined item of all cause mortality and reinfection by the same enterobacteria at 30 days.

**Results:**

273 urine cultures were positive for ESBL-EB during the study period. 75 episodes were included, 40 in the long treatment group and 35 in the short treatment group. Mean treatment duration in short and long treatment groups was 6,1 and 13,8 days respectively. Mortality at 30 days was 5,7% in the short treatment group and 5% in the long treatment group without significant differences (P = 0,8). Mortality or reinfection by the same ESBL-EB at 30 days was 8,6% in the short treatment group and 10% in the long treatment group, without significant differences (P = 0,8).

**Conclusions:**

Short courses of antimicrobial treatment seems to be effective as treatment of complicated urinary tract infections by ESBL-EB.

## Introduction

Infections by multi-resistant microorganism are a problem increasing in our clinical practice. Specially, extended-spectrum beta-lactamase producing enterobacterales (ESBL-EB) are a priority. They are part of the group posing the highest public health risk according to the WHO statement published in 2018 [1]. These microorganisms are a frequent cause of urinary tract infection, with a high incidence in the hospital setting. In Europe, *E*. *coli* resistance to third generation cephalosporins in 2018 was 15,1% and *K*. *pneumoniae* resistance to third generation cephalosporins was 31,7% [2]. In United States, there are high levels of antimicrobial resistance in enterobacterales in hospitalized patients [3] and rates of ESBL-EB increased between 2013 and 2017 [4].

Several strategies have been developed to reduce the incidence of infections by these microorganisms. It is essential to optimize antibiotic therapy in accordance to the need, the drug choice and the duration of the treatment [5, 6]. There is evidence on the effectiveness of short course of antibiotic therapy in the treatment of urinary tract infections [7]. However, there are no specific studies on the duration of antimicrobial treatment in complicated urinary tract infections caused by ESBL-EB.

The aim of this study was to evaluate the clinical outcome in patients who received short (≤ 7 days) versus long courses (>7 days) of antimicrobial therapy for complicated ESBL-EB urinary tract infections.

## Material and methods

This is an observational and retrospective study. Positive urine cultures for ESBL-EB in our hospital between March 2015 and July 2017 were identified. The database of the Microbiology department of the hospital was used.

Patients who met criteria for complicated urinary tract infection according to the IDSA guidelines were included [8]. A second analysis was performed including only cases that met the recommended criteria for conducting clinical trials by the FDA: clinical syndrome characterized by pyuria and a documented microbial pathogen on culture of urine or blood, accompanied by local and systemic signs and symptoms, including fever (i.e., oral or tympanic temperature greater than 38 degrees Celsius), chills, malaise, flank pain, back pain, and/or costovertebral joints pain or tenderness, which occur in the presence of a functional or anatomical abnormality of the urinary tract or in the presence of catheterization [9].

Exclusion criteria were: age under 18 years old, asymptomatic bacteriuria, uncomplicated urinary tract infections, recurrences in the first week after the end of treatment, polymicrobial urine cultures, inadequate treatment according to antimicrobial susceptibility results and death before the end of the antibiotic treatment.

The study was approved by “Galicia Research Ethics Committee”. All data were deidentified before we accesed them.

Both Specialist and Primary Care electronic medical records were reviewed and a database was created with the following variables:

- **Demographic and anthropometric variables:** Age, sex, weight, height and BMI.
- **Origin of infection:** Community, nosocomial (defined as occurring after the second day of admission or within 10 days after discharge) or healthcare-associated (defined as admission to the hospital in the previous 90 days, institutionalized patient, treatment in day units, dialysis or home hospitalization).
- **Devices within 7 days prior to collection of urine culture:** Central and urinary catheter or gastrostomy
- **Comorbidities:** Hypertension, heart failure stage C (rated by ACC / AHA), heart disease of any etiology (including hypertensive, ischemic or valvular heart disease, heart failure, arrhythmias and cardiomyopathy), diabetes mellitus with or without target organ damage (neuropathy, nephropathy, retinopathy), estimated glomerular filtration rate calculated by the MDRD modified formula (categorizing the degree of renal failure as severe < 30 ml/min and moderate between 30–60 ml/min according to the KDOQI guidelines of the National Kidney Foundation), COPD confirmed by spirometry, peripheral arterial disease (intermittent claudication, acute arterial ischemia, aortic aneurysm > 6 cm, peripheral by-pass), cerebrovascular disease (transient ischemic attack or ischemic stroke), cognitive impairment, solid tumor, leukemia or lymphoma, connective tissue disease, mild chronic liver disease (non portal hypertension) or severe with portal hypertension, AIDS, neutropenia (< 500 neutrophils at the time of urine culture), corticosteroids or immunosuppressive treatment. Charlson comorbidity index was calculated for each patient.
- **Barthel index**
- **Clinical and analytical features of each episode:** Measure of systolic and diastolic blood pressure, heart rate, temperature, serum leukocyte, hemoglobin, platelets, urea and creatinine in the urine culture collection day or in the nearest time. Presence of sepsis or septic shock criteria were evaluated.
- **Microbiological features:** Urine samples were cultured in blood agar and CPS agar and they were incubated a minimum of 24 hours at 35–37°C in aerobic atmosphere. The microorganism was identified and sensitivity tests were carried out in the Biomerieux VITEK 2 system. For the identification of ESBL production, phenotypic methods were used according to the CLSI recommendations.
- Adequate empirical antibiotic treatment, adequate definitive treatment according to antibiogram and antibiotic treatment duration.

The primary outcome analyzed was all cause 30-day mortality.

The secondary outcome was a combined item of all cause mortality and reinfection by the same enterobacteria at 30 days.

### Statistical analysis

Differences in baseline characteristics between treatment groups were analyzed using the Chi-square test or Fisher's exact test for the analysis of dichotomous variables and the Student's T test in the quantitative variables with normal distribution or the Mann-Withney U test in the quantitative variables without normal distribution. Quantitative variables with statistically significant differences were dichotomized using their median as a cut-off point. Variables clinically relevant and with P <0.05 were included in a binary logistic regression model, calculating a propensity index for assigning each patient to short treatment. For the bivariate survival analysis, Kaplan-meier curves were performed and the Log-rank test was used to assess the differences between curves. Finally, a multivariate survival analysis was performed using Cox regression, using a temporary variable (number of days to the event) and a dichotomous variable (occurrence of the event). All the variables that presented a P <0.1, as well as the Charlson comorbidity index, sex, age and the previously calculated propensity index were included in the multivariate analysis. Stratified analysis by sex was subsequently performed. Statistical significance was established with P<0,05. The statistical program SPSS 21 was used.

## Results

273 urine cultures were positive for ESBL-EB during the study period. 75 episodes in 70 patients met the inclusion criteria and were included, 40 in the long treatment group and 35 in the short treatment group. The median age was 79 (ST 16.1) years and 43 (57.3%) were women. The median Charlson index was 2 (ST 2.02). Proportion of male sex was higher in long treatment group (55% vs 28,5%; OR 3; 95% CI 1,1–7,9; P = 0,02).

In bivariate analysis there were more patients with hypertension in the short treatment group (47,5% vs 71,42%; OR 2,76, 95% CI 1,05–7,22; P = 0,03). Although in the multivariate analysis the only significant difference between groups was male sex. There were no other differences in baseline characteristics between groups (Table 1).

**Table 1:** Baseline characteristics.

BASELINE CHARACTERISTICS	LONG TREATMENT	SHORT TREATMENT	OR	95% CI	P
Male sex	22/40 (55%)	10/35 (28,5%)	3,05	1,16–7,99	0,02
Age ≥ 80 years	20/40 (50%)	14/35 (40%)	0,66	0,26–1,66	0,38
Charlson index > 2	20/40 (50%)	16/35 (45,7%)	0,84	0,33–2,09	0,71
Urinary catheter	10/40 (25%)	6/35 (17,14%)	0,62	0,20–1,92	0,40
Cognitive impairment	7/40 (17,5%)	12/35 (34,28%)	2,46	0,84–7,19	0,09
Cerebrovascular disease	9/40 (22,5%)	7/35 (20%)	0,86	0,28–2,61	0,79
Immunosupression	1/40 (2,5%)	3/35 (8,57%)	3.65	0,36–36,87	0,33
COPD	3/40 (7,5%)	2/35 (5,71%)	0,74	0,11–4,75	1
Heart failure	4/40 (10%)	8/35 (22,85%)	2,66	0,72–9,78	0,13
Peripheral arterial disease	6/40 (15%)	2/35 (5,71%)	0,34	0,06–1,82	0,27
Hypertension	19/40 (47,5%)	25/35 (71,42%)	2,76	1,05–7,22	0,03
Dyslipidemia	15/40 (37,5%)	17/35 (51,42%)	1,76	0,70–4,43	0,22
Diabetes mellitus	11/40 (27,5%)	13/35 (37,14%)	1,55	0,58–4,13	0,37
Solid tumor	7/40 (17,5%)	3/35 (8,57%)	0,44	0,1–1,86	0,32
Solid tumor with metastases	1/40 (2,5%)	1/35 (2,85%)	1,14	0,06–19,04	1
Chronic liver disease	6/40 (15%)	2/35 (5,71%)	0,34	0,06–1,82	0,27
Moderate /severe chronic kidney disease	2/40 (5%)	7/35 (20%)	4,75	0,91–24,62	0,07
Connective tissue disease	1/40 (2,5%)	3/35 (8,57%)	3,65	0,36–36,87	0,33

The most frequently isolated microorganism was *Escherichia coli* (61 cases, 81.2%), followed by *Klebsiella spp* (11 cases, 14.7%) and *Proteus mirabilis* (2 cases, 2.7%). 36 patients (48%) presented urinary tract infection without fever, 26 patients (34.6%) febrile urinary tract infection and 13 patients (17.4%) pyelonephritis.

Blood cultures were performed in 16 cases (21.3%) and 4 patients (5.3%) presented bacteremia due to the same enterobacteria. Sepsis or septic was observed in 8 patients (10,7%).

93.1% of patients presented a temperature 37.8ºC or less during the first 48 hours and 94.8% hemodynamic stability during the first 48 hours. There were no differences in the type of infection, the severity of infection or vital signs at day 2 between both groups (Table 2).

**Table 2:** Clinical feature.

VARIABLE	LONG TREATMENT	SHORT TREATMENT	OR	95% CI	P
Sepsis/Septic shock	5/40 (12,5%)	3/35 (8,6%)	0,65	0,14–2,96	0,71
Cystitis	16/40 (40%)	20/35 (57,14%)	2	0,79–5,02	0,13
Febrile UTI	14/40 (35%)	12/35 (34,28%)	0,96	0,37–2,51	0,94
Pyelonephritis	10/40 (25%)	3/35 (8,6%)	0,28	0,07–1,12	0,06
Leukocytosis	13/39 (33,33%)	7/32 (21,87%)	0,56	0,19–1,63	0,28
Thrombopenia	3/39 (7,69%)	3/32 (9,37%)	1,24	0,23–6,61	1
Renal failure	14/39 (35,89%)	17/32 (53,12%)	2,02	0,78–5,25	0,14
Nosocomial	7/40 (17,5%)	2/35 (5,7%)	0,28	0,55–1,47	0,11

55 patients (73.3%) were hospitalized at the time of the urine culture. Community-acquired urinary tract infections were observed in 32 patients (42.7%), healthcare-associated in 34 patients (45.3%) and hospital-acquired in 9 patients (12%). 22 patients (29.3%) had an urological abnormality and 12 (16%) had permanent urinary catheter.

In 36 patients (48%) adequate empirical antibiotic therapy was performed. Definitive treatment with carbapenem was used in 36 patients (48%), followed by fosfomycin in 13 (17.3%), quinolones in 12 (16%), cotrimoxazole in 7 (9.3%), beta-lactam plus betalactamase inhibitor in 5 (6.7%), furantoin in 2 (2.7%) and aminoglycosides in 1 (1.3%).

Mean treatment duration in short and long treatment groups was 6,1 and 13,8 days respectively.

**Fig 1:**
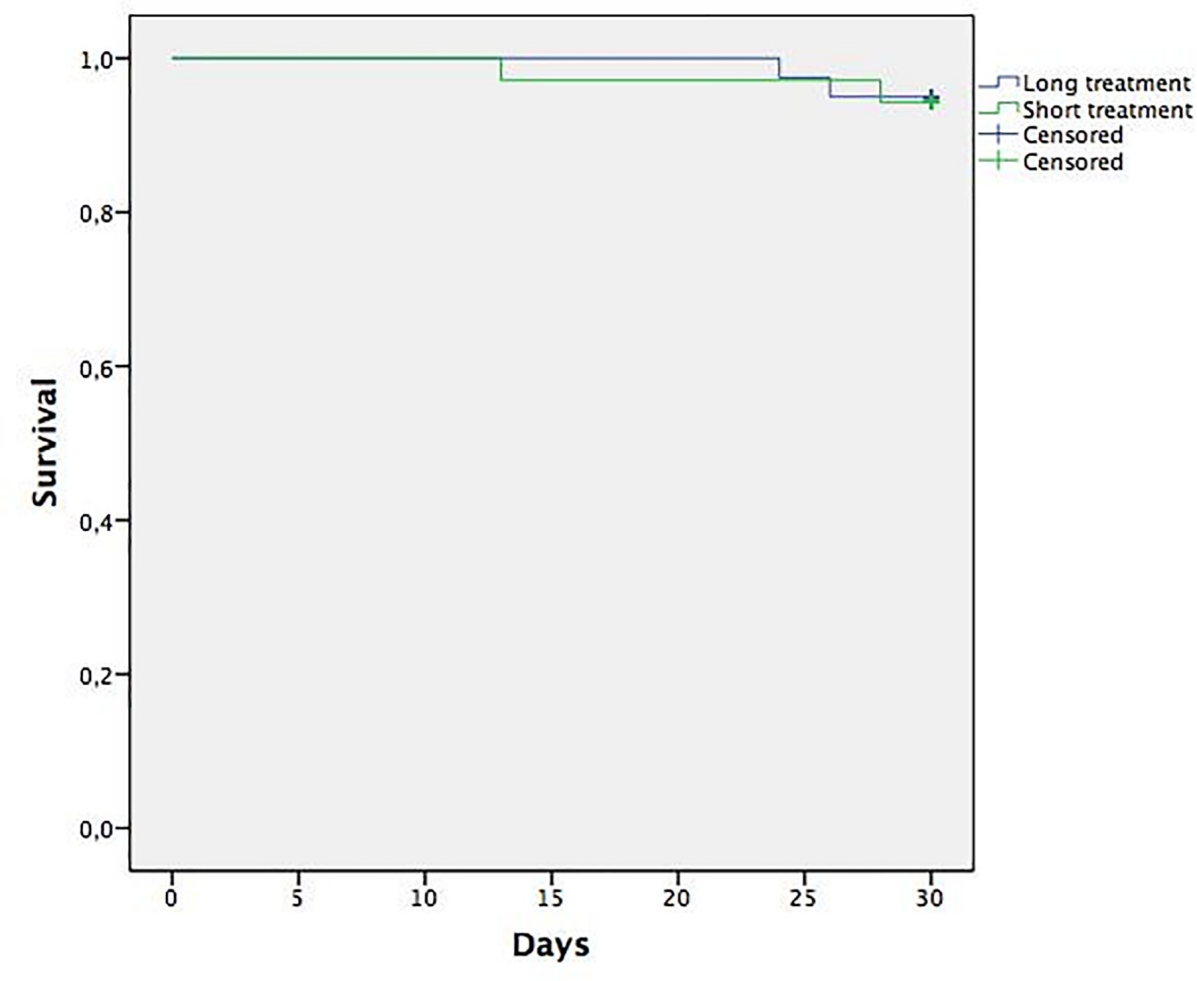
Mortality at 30 days.

**Fig 2:**
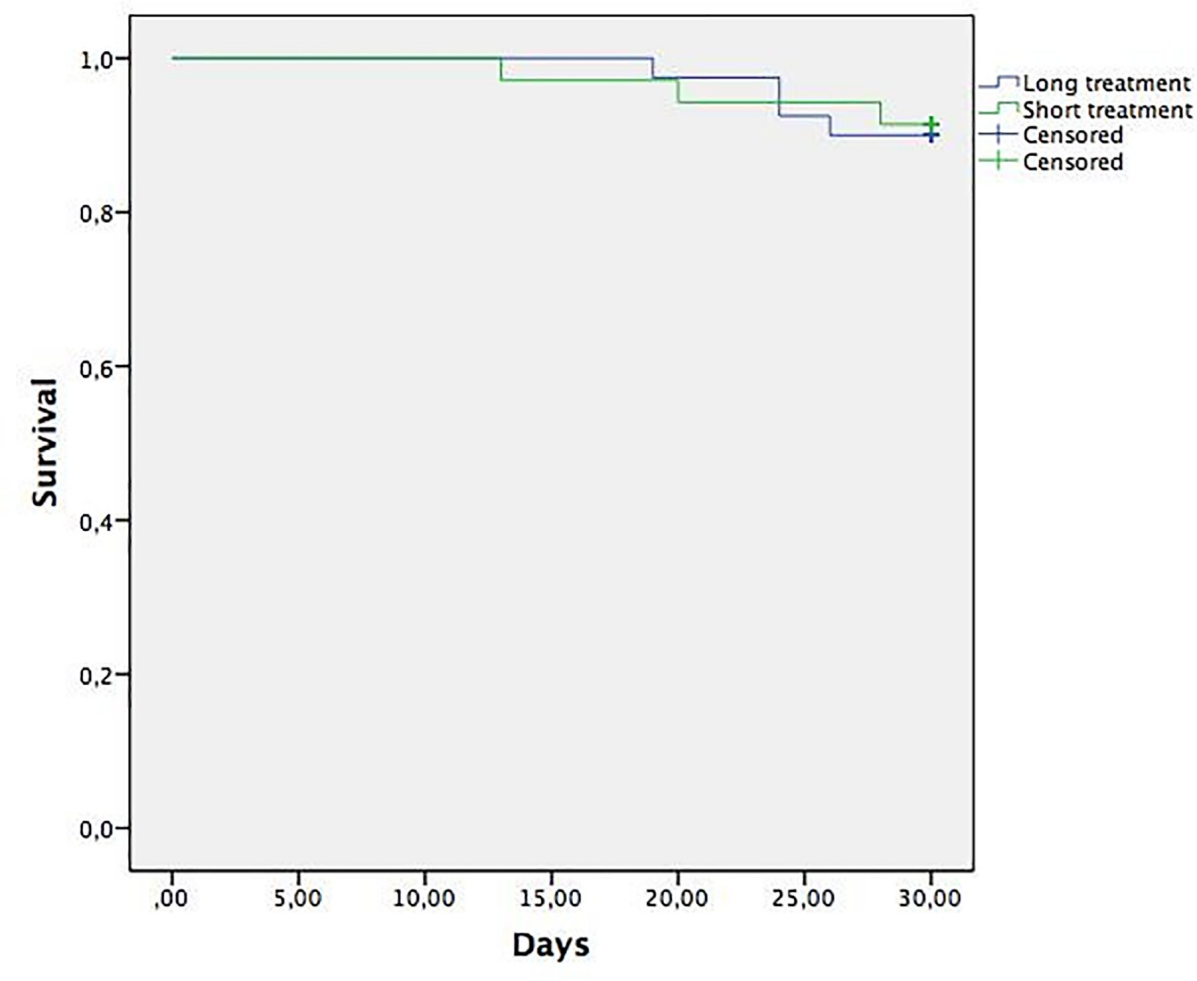
Mortality or reinfection at 30 days.

Mortality at 30 days was 5.7% in the short treatment group and 5% in the long treatment group without significant differences (P = 0,8) (Fig 1). Mortality or reinfection by the same microorganism at 30 days was 8.6% in the short treatment group and 10% in the long one (P = 0.8) (Fig 2). Mortality at 30 days in the male subgroup was 0% in the short treatment group and 10% in the long treatment group (P = 0.1). Mortality or reinfection at 30 days in the male subgroup was 10% in the short treatment group and 9.1% in the long treatment group (P = 0.9).

For the analysis of patients following the FDA criteria for complicated urinary tract infection, we excluded 24 patients. Finally, 51 cases met the inclusion criteria and were included, 22 in the long treatment group and 29 in the short treatment group. There was no difference in 30-day mortality (4.5% in short treatment vs 3.4% in long treatment, P = 0,8) nor in 30-day mortality or reinfection (9.1% in short treatment vs 6.9% in long treatment, P = 0.7).

In the multivariate analysis, the factors associated with higher mortality at 30 days were the presence of leukopenia at the time of diagnosis (HR 16.4; 95% CI 1.4–181.1; P = 0.02) and the history of metastatic cancer (HR 1.6; 95% CI 1.09–2.4; P = 0.01). The factors associated with higher mortality or reinfection were the history of lymphoma (HR 9; 95% CI 1.004–81.2; P = 0.05) and definitive treatment with beta-lactam associated with beta-lactamase inhibitor (HR 8.4; CI 95% 1.5–46.1; P = 0.01).

## Discussion

We have not found differences in mortality and reinfection between long and short treatment groups in patients with complicated urinary tract infection caused by ESBL-EB.

There are no clear recommendations in guidelines regarding the duration of antibiotic treatment in complicated urinary tract infections. However, there are studies that also support a short course of antibiotic therapy in these types of infections. A prospective, non-inferiority trial undertaken at 21 centres of infectious diseases in Sweden showed that 7 days of ciprofloxacin was not inferior to 14 days of treatment in women with acute pyelonephritis, including older women and those with a more severe infection. The clinical and bacteriological cure rates were high for both regimens [10]. A randomized, placebo-controlled trial conducted in women and men with febrile urinary tract infection showed that 7 days of antimicrobial treatment in women were non-inferior to 14 days of therapy. In men, 7 days of antibiotic treatment was inferior to 14 days during short-term follow-up (10–18 day post-treatment) but it was non-inferior when looking at longer follow-up (70–84 days post-treatment) [11]. A meta-analysis revealed that seven days of treatment for acute pyelonephritis is equivalent to a longer treatment in terms of clinical failure and microbiological failure, including bacteremic patients. However, in patients with urogenital abnormalities, the evidence suggested that a longer treatment is required [7]. In the randomized trial by Dinh *et al*., the efficacy of 5 days of fluoroquinolone treatment does not seem different from 10 days of treatment for acute uncomplicated pyelonephritis [12]. Similar results were observed in the study published by Peterson *et al*. They compared 5 days of levofloxacin 750 mg once daily to 10 days of a standard dose of ciprofloxacin among patients with complicated UTI and pyelonephritis in a double-blind randomized trial including 619 patients [13]. In a retrospective study, the treatment with a 7-day antibiotic course in men with urinary tract infection without evidence of complicating conditions was not associated with increased risk of recurrence [14].

To our knowledge, there are no studies that evaluate the duration of antimicrobial therapy in patients with urinary tract infections caused by ESBL-EB. There is a tendency to perform longer antibiotic treatments in patients with infections by these microorganisms. In a retrospective study that evaluated treatment with ertapenem administered through outpatient parenteral antibiotic therapy (OPAT) in patients with urinary tract infections caused by ESBL-EB, the mean duration of antimicrobial treatment was 11.2 days [15]. A prospective and observational study that included patients with complicated urinary tract infection caused by ESBL-producing *E*. *coli* evaluated the treatment with meropenem or imipenem for 14 days compared to 3 doses of 3 gr fosfomycin tromethanol every other day. Clinical and microbiological success in the carbapenem and fosfomycin groups was similar [16]. Other retrospective study compared ertapenem to oral fosfomycin for outpatient treatment of ESBL-EB urinary tract infections. Total antibiotic duration was 10 days (IQR 7–12 days) in the fosfomycin group and 15 days (IQR 12–16 days) in the ertapenem group, without differences in infection-related hospital readmissions within 30 days [17].

Our study is, to our knowledge, the first one conducted in patients with complicated urinary tract infection by ESBL-EB. No worse evolution was observed in patients treated with short antibiotic courses. We have not observed a worse evolution using different criteria to define complicated urinary tract infection or in patients more susceptible *a priori* to needing a longer antibiotic treatment, which could be the case of men or patients with urological abnormalities.

There are, however, some limitations. First of all, it is a retrospective study, so there may be uncontrolled confounding factors despite the different statistical analysis performed. Secondly, it has been done in a single center, so the results may not be generalizable to different population groups.

## Conclusions

Patients with complicated urinary tract infections caused by ESBL-EB can be treated with a short course of antimicrobial therapy. Shorter duration of antibiotic treatment may lead to decreased risk of antibiotic resistance, fewer adverse effects, and lower costs. It is necessary to conduct randomized, controlled trial to establish the adequate duration of antibiotic treatment in these types of infections.

## Supporting information

S1 File Variables description. (DOCX)

S2 File Baseline characteristics. (PDF)

S3 File Mortality at 30 days. Bivariant analysis.
(PDF)

S4 File Mortality or reinfection at 30 days. Bivariant analysis.
(PDF)

S5 File Mortality at 30 days. Multivariate analysis.
(PDF)

S6 File Mortality or reinfection at 30 days. Multivariate analysis.
(PDF)
