# Supplementary material for: Treatment duration of complicated urinary tract infections by extended-spectrum beta-lactamases producing enterobacterales
Source: PLoS One. 2020 Oct 19;15(10):e0237365. doi: 10.1371/journal.pone.0237365 (PMC7571686; doi:10.1371/journal.pone.0237365)
Supplement: S1 File — (DOCX) [file pone.0237365.s001.docx]

**Variables**

-Date urine culture

-Concomitant bacteremia by extended-spectrum beta-lactamases producing enterobacterales

-Type of microorganism

-Infection type: cystitis (urinary symptons without fever), febrile UTI (with fever 38 or more or sepsis) or pyelonephritis

-Complicated UTI: UTI in patients with comorbidities (diabetes, immunosuppression, cronic kidney disease, etc.), UTI in patients with functional or anatomical abnormality of the urinary tract or in the presence of catheterization, pyelonephritis, UTI in men and UTI during pregnancy

-Urological abnormality (lithiasis, congenital pathology, ureteral obstruction)

-Sex of the patient

-Age of the patient

-Malnutrition: BMI <18

-Obese: BMI> 30

-BMI

-Devices within 7 days prior to collection of urine culture: central and urinary catheter or gastrostomy

-Permanent urinary catheter: > 1 month with the catheter

-Cognitive impairment

-Solid tumor: cancer without documented metastases

-Solid tumor with metastases

-Leukemia

-Lymphoma

-Chronic liver disease without portal hypertension

-Chronic liver disease with portal hypertension

-Connective disease

-Diabetes mellitus without target organ damage (neuropathy, nephropathy, retinopathy)

-Diabetes mellitus with or target organ damage (neuropathy, nephropathy, retinopathy)

-Hypertension

-Dyslipidemia

-Previous chronic respiratory pathology: restrictive syndrome, COPD, bronchiectasis, asthma, EPID, CF, deficit 1AT

-COPD confirmed by spirometry

-Heart failure stage C (rated by ACC / AHA )

-Heart disease of any etiology (including hypertensive, ischemic or valvular heart disease, heart failure, arrhythmias and cardiomyopathy)

-Peripheral arterial disease (intermittent claudication, acute arterial ischemia, aortic aneurysm > 6 cm, peripheral by-pass),

-Cerebrovascular disease (transient ischemic attack or ischemic stroke)

-Hemiplegia: hemiparesis, hemiplegia or paraplegia of any cause

-Peptic ulcer disease

-Moderate /severe chronic kidney disease

-Dialysis

-HIV

-AIDS

-Neutropenia: neutrophils <500 cell / mm at the time of urine culture

-Transplant

-Corticosteroids

-Immunosuppressive treatment

-Barthel index

-Charlon index

-Surgery 30 days before of urine culture

-Leukocytosis: >12,000/mm

-Leukopenia: <4000/mm

-Thrombopenia: <100,000/mm

-Renal failure at the moment of urine culture

-Sepsis: meets SOFA criteria

-Septic shock: sepsis with amines/intubation

-Antibiotic in the previous 90 days

-UTI repetition: 3 or more in the last year

-Hospitalized patient at the time of urine culture

-Number of days admitted 90 days prior

-Intensive care

-Number of days in intensive care

-Origin of infection: Community, nosocomial (defined as occurring after the second day of admission or within 10 days after discharge) or healthcare-associated (defined as admission to the hospital in the previous 90 days, institutionalized patient, treatment in day hospital, dialysis or home hospitalization).

-Afebrile during the first 48 hours

-Hemodynamic stability during the first 48 hours

-Type of empirical antibiotic treatment

-Adequate empirical antibiotic treatment

-Adequate definitive treatment according to antibiogram

-Antibiotic treatment duration: number of days of adequate treatment

-Type of definitive antibiotic treatment

-Reinfection by the same enterobacteria at 30 days

-Date of reinfection by the same enterobacteria

-Death

-Date of death

-Cause of death
