# Supplementary material for: Treatment duration of complicated urinary tract infections by extended-spectrum beta-lactamases producing enterobacterales
Source: PLoS One. 2020 Oct 19;15(10):e0237365. doi: 10.1371/journal.pone.0237365 (PMC7571686; doi:10.1371/journal.pone.0237365)
Supplement: S2 File — (PDF) [file pone.0237365.s002.pdf]

Crosstabs

Urolithiasis * Short_treatment

Crosstab

			Short_treatment		Total
			,00	1,00	
Urolithiasis	,00	Count	34	28	62
		% within Urolithiasis	54,8%	45,2%	100,0%
		% within Short_treatment	85,0%	80,0%	82,7%
		% of Total	45,3%	37,3%	82,7%
	1,00	Count	6	7	13
		% within Urolithiasis	46,2%	53,8%	100,0%
		% within Short_treatment	15,0%	20,0%	17,3%
		% of Total	8,0%	9,3%	17,3%
Total		Count	40	35	75
		% within Urolithiasis	53,3%	46,7%	100,0%
		% within Short_treatment	100,0%	100,0%	100,0%
		% of Total	53,3%	46,7%	100,0%

Chi-Square Tests

	Value	df	Asymp. Sig. (2-sided)	Exact Sig. (2-sided)	Exact Sig. (1-sided)
Pearson Chi-Square	,326[a]	1	,568		
Continuity Correction[b]	,070	1	,791		
Likelihood Ratio	,325	1	,569		
Fisher's Exact Test				,761	,394
Linear-by-Linear Association	,321	1	,571		
N of Valid Cases	75				

a. 0 cells (0,0%) have expected count less than 5. The minimum expected count is 6,07.

b. Computed only for a 2x2 table

Risk Estimate

	Value	95% Confidence Interval	
		Lower	Upper
Odds Ratio for Urolithiasis (,00 / 1,00)	1,417	,427	4,702
For cohort Short_treatment = ,00	1,188	,633	2,229
For cohort Short_treatment = 1,00	,839	,473	1,488
N of Valid Cases	75		

Nephrostomy * Short_treatment

Crosstab

			Short_treatment		Total
			,00	1,00	
Nephrostomy	,00	Count	38	35	73
		% within Nephrostomy	52,1%	47,9%	100,0%
		% within Short_treatment	95,0%	100,0%	97,3%
		% of Total	50,7%	46,7%	97,3%
	1,00	Count	2	0	2
		% within Nephrostomy	100,0%	0,0%	100,0%
		% within Short_treatment	5,0%	0,0%	2,7%
		% of Total	2,7%	0,0%	2,7%
Total		Count	40	35	75
		% within Nephrostomy	53,3%	46,7%	100,0%
		% within Short_treatment	100,0%	100,0%	100,0%
		% of Total	53,3%	46,7%	100,0%

Chi-Square Tests

	Value	df	Asymp. Sig. (2-sided)	Exact Sig. (2-sided)	Exact Sig. (1-sided)
Pearson Chi-Square	1,798[a]	1	,180		
Continuity Correction[b]	,388	1	,534		
Likelihood Ratio	2,562	1	,109		
Fisher's Exact Test				,495	,281
Linear-by-Linear Association	1,774	1	,183		
N of Valid Cases	75				

a. 2 cells (50,0%) have expected count less than 5. The minimum expected count is ,93.

b. Computed only for a 2x2 table

Risk Estimate

	Value	95% Confidence Interval	
		Lower	Upper
For cohort Short_treatment = ,00	,521	,418	,649
N of Valid Cases	75		

Previous_ESBL * Short_treatment

Crosstab

			Short_treatment		Total
			,00	1,00	
Previous_ESBL	0	Count	8	4	12
		% within Previous_ESBL	66,7%	33,3%	100,0%
		% within Short_treatment	72,7%	80,0%	75,0%
		% of Total	50,0%	25,0%	75,0%
	1	Count	3	1	4
		% within Previous_ESBL	75,0%	25,0%	100,0%
		% within Short_treatment	27,3%	20,0%	25,0%
		% of Total	18,8%	6,3%	25,0%
Total		Count	11	5	16
		% within Previous_ESBL	68,8%	31,3%	100,0%
		% within Short_treatment	100,0%	100,0%	100,0%
		% of Total	68,8%	31,3%	100,0%

Chi-Square Tests

	Value	df	Asymp. Sig. (2-sided)	Exact Sig. (2-sided)	Exact Sig. (1-sided)
Pearson Chi-Square	,097[a]	1	,755		
Continuity Correction[b]	,000	1	1,000		
Likelihood Ratio	,100	1	,752		
Fisher's Exact Test				1,000	,635
Linear-by-Linear Association	,091	1	,763		
N of Valid Cases	16				

a. 3 cells (75,0%) have expected count less than 5. The minimum expected count is 1,25.

b. Computed only for a 2x2 table

Risk Estimate

	Value	95% Confidence Interval	
		Lower	Upper
Odds Ratio for Previous_ESBL (0 / 1)	,667	,051	8,639
For cohort Short_treatment = ,00	,889	,445	1,777
For cohort Short_treatment = 1,00	1,333	,204	8,708
N of Valid Cases	16		

SEPSIS_SHOCK * Short_treatment

Crosstab

			Short_treatment		Total
			,00	1,00	
SEPSIS_SHOCK	,00	Count	35	32	67
		% within SEPSIS_SHOCK	52,2%	47,8%	100,0%
		% within Short_treatment	87,5%	91,4%	89,3%
		% of Total	46,7%	42,7%	89,3%
	1,00	Count	5	3	8
		% within SEPSIS_SHOCK	62,5%	37,5%	100,0%
		% within Short_treatment	12,5%	8,6%	10,7%
		% of Total	6,7%	4,0%	10,7%
Total		Count	40	35	75
		% within SEPSIS_SHOCK	53,3%	46,7%	100,0%
		% within Short_treatment	100,0%	100,0%	100,0%
		% of Total	53,3%	46,7%	100,0%

Chi-Square Tests

	Value	df	Asymp. Sig. (2-sided)	Exact Sig. (2-sided)	Exact Sig. (1-sided)
Pearson Chi-Square	,302[a]	1	,582		
Continuity Correction[b]	,031	1	,861		
Likelihood Ratio	,306	1	,580		
Fisher's Exact Test				,716	,434
Linear-by-Linear Association	,298	1	,585		
N of Valid Cases	75				

a. 2 cells (50,0%) have expected count less than 5. The minimum expected count is 3,73.

b. Computed only for a 2x2 table

Risk Estimate

	Value	95% Confidence Interval	
		Lower	Upper
Odds Ratio for SEPSIS_SHOCK (,00 / 1,00)	,656	,145	2,969
For cohort Short_treatment = ,00	,836	,466	1,498
For cohort Short_treatment = 1,00	1,274	,503	3,225
N of Valid Cases	75		

Sepsis * Short_treatment

Crosstab

			Short_treatment		Total
			,00	1,00	
Sepsis	,0	Count	36	32	68
		% within Sepsis	52,9%	47,1%	100,0%
		% within Short_treatment	90,0%	91,4%	90,7%
		% of Total	48,0%	42,7%	90,7%
	1,0	Count	4	3	7
		% within Sepsis	57,1%	42,9%	100,0%
		% within Short_treatment	10,0%	8,6%	9,3%
		% of Total	5,3%	4,0%	9,3%
Total		Count	40	35	75
		% within Sepsis	53,3%	46,7%	100,0%
		% within Short_treatment	100,0%	100,0%	100,0%
		% of Total	53,3%	46,7%	100,0%

Chi-Square Tests

	Value	df	Asymp. Sig. (2-sided)	Exact Sig. (2-sided)	Exact Sig. (1-sided)
Pearson Chi-Square	,045[a]	1	,832		
Continuity Correction[b]	,000	1	1,000		
Likelihood Ratio	,045	1	,832		
Fisher's Exact Test				1,000	,576
Linear-by-Linear Association	,044	1	,833		
N of Valid Cases	75				

a. 2 cells (50,0%) have expected count less than 5. The minimum expected count is 3,27.

b. Computed only for a 2x2 table

Risk Estimate

	Value	95% Confidence Interval	
		Lower	Upper
Odds Ratio for Sepsis (,0 / 1,0)	,844	,175	4,059
For cohort Short_treatment = ,00	,926	,470	1,828
For cohort Short_treatment = 1,00	1,098	,450	2,679
N of Valid Cases	75		

Septic_shock * Short_treatment

Crosstab

			Short_treatment		Total
			,00	1,00	
Septic_shock	,0	Count	39	35	74
		% within Septic_shock	52,7%	47,3%	100,0%
		% within Short_treatment	97,5%	100,0%	98,7%
		% of Total	52,0%	46,7%	98,7%
	1,0	Count	1	0	1
		% within Septic_shock	100,0%	0,0%	100,0%
		% within Short_treatment	2,5%	0,0%	1,3%
		% of Total	1,3%	0,0%	1,3%
Total		Count	40	35	75
		% within Septic_shock	53,3%	46,7%	100,0%
		% within Short_treatment	100,0%	100,0%	100,0%
		% of Total	53,3%	46,7%	100,0%

Chi-Square Tests

	Value	df	Asymp. Sig. (2-sided)	Exact Sig. (2-sided)	Exact Sig. (1-sided)
Pearson Chi-Square	,887[a]	1	,346		
Continuity Correction[b]	,000	1	1,000		
Likelihood Ratio	1,269	1	,260		
Fisher's Exact Test				1,000	,533
Linear-by-Linear Association	,875	1	,350		
N of Valid Cases	75				

a. 2 cells (50,0%) have expected count less than 5. The minimum expected count is ,47.

b. Computed only for a 2x2 table

Risk Estimate

	Value	95% Confidence Interval	
		Lower	Upper
For cohort Short_treatment = ,00	,527	,425	,654
N of Valid Cases	75		

Infection_type * Short_treatment

Crosstab

			Short_treatment		Total
			,00	1,00	
Infection_type	,0	Count	16	20	36
		% within Infection_type	44,4%	55,6%	100,0%
		% within Short_treatment	40,0%	57,1%	48,0%
		% of Total	21,3%	26,7%	48,0%
	1,0	Count	14	11	25
		% within Infection_type	56,0%	44,0%	100,0%
		% within Short_treatment	35,0%	31,4%	33,3%
		% of Total	18,7%	14,7%	33,3%
	2,0	Count	10	3	13
		% within Infection_type	76,9%	23,1%	100,0%
		% within Short_treatment	25,0%	8,6%	17,3%
		% of Total	13,3%	4,0%	17,3%
	3,0	Count	0	1	1
		% within Infection_type	0,0%	100,0%	100,0%
		% within Short_treatment	0,0%	2,9%	1,3%
		% of Total	0,0%	1,3%	1,3%
Total		Count	40	35	75
		% within Infection_type	53,3%	46,7%	100,0%
		% within Short_treatment	100,0%	100,0%	100,0%
		% of Total	53,3%	46,7%	100,0%

Chi-Square Tests

	Value	df	Asymp. Sig. (2-sided)
Pearson Chi-Square	5,264[a]	3	,153
Likelihood Ratio	5,835	3	,120
Linear-by-Linear Association	2,275	1	,131
N of Valid Cases	75		

a. 2 cells (25,0%) have expected count less than 5. The minimum expected count is ,47.

Risk Estimate

	Value
Odds Ratio for Infection_type (,0 / 1,0)	[a]

a. Risk Estimate statistics cannot be computed. They are only computed for a 2*2 table without empty cells.

Urological_abnormality * Short_treatment

Crosstab

			Short_treatment		Total
			,00	1,00	
Urological_abnormality	,0	Count	30	23	53
		% within Urological_abnormality	56,6%	43,4%	100,0%
		% within Short_treatment	75,0%	65,7%	70,7%
		% of Total	40,0%	30,7%	70,7%
	1,0	Count	10	12	22
		% within Urological_abnormality	45,5%	54,5%	100,0%
		% within Short_treatment	25,0%	34,3%	29,3%
		% of Total	13,3%	16,0%	29,3%
Total		Count	40	35	75
		% within Urological_abnormality	53,3%	46,7%	100,0%
		% within Short_treatment	100,0%	100,0%	100,0%
		% of Total	53,3%	46,7%	100,0%

Chi-Square Tests

	Value	df	Asymp. Sig. (2-sided)	Exact Sig. (2-sided)	Exact Sig. (1-sided)
Pearson Chi-Square	,776[a]	1	,378		
Continuity Correction[b]	,393	1	,531		
Likelihood Ratio	,776	1	,378		
Fisher's Exact Test				,450	,265
Linear-by-Linear Association	,766	1	,381		
N of Valid Cases	75				

a. 0 cells (0,0%) have expected count less than 5. The minimum expected count is 10,27.

b. Computed only for a 2x2 table

Risk Estimate

	Value	95% Confidence Interval	
		Lower	Upper
Odds Ratio for Urological_abnormality (,0 / 1,0)	1,565	,576	4,253
For cohort Short_treatment = ,00	1,245	,744	2,084
For cohort Short_treatment = 1,00	,796	,487	1,299
N of Valid Cases	75		

Sex * Short_treatment

Crosstab

			Short_treatment		Total
			,00	1,00	
Sex	,0	Count	22	10	32
		% within Sex	68,8%	31,3%	100,0%
		% within Short_treatment	55,0%	28,6%	42,7%
		% of Total	29,3%	13,3%	42,7%
	1,0	Count	18	25	43
		% within Sex	41,9%	58,1%	100,0%
		% within Short_treatment	45,0%	71,4%	57,3%
		% of Total	24,0%	33,3%	57,3%
Total		Count	40	35	75
		% within Sex	53,3%	46,7%	100,0%
		% within Short_treatment	100,0%	100,0%	100,0%
		% of Total	53,3%	46,7%	100,0%

Chi-Square Tests

	Value	df	Asymp. Sig. (2-sided)	Exact Sig. (2-sided)	Exact Sig. (1-sided)
Pearson Chi-Square	5,330[a]	1	,021		
Continuity Correction[b]	4,304	1	,038		
Likelihood Ratio	5,423	1	,020		
Fisher's Exact Test				,035	,018
Linear-by-Linear Association	5,259	1	,022		
N of Valid Cases	75				

a. 0 cells (0,0%) have expected count less than 5. The minimum expected count is 14,93.

b. Computed only for a 2x2 table

Risk Estimate

	Value	95% Confidence Interval	
		Lower	Upper
Odds Ratio for Sex (,0 / 1,0)	3,056	1,167	7,998
For cohort Short_treatment = ,00	1,642	1,076	2,506
For cohort Short_treatment = 1,00	,538	,303	,953
N of Valid Cases	75		

Mechanic_ventilation * Short_treatment

Crosstab

			Short_treatment		Total
			,00	1,00	
Mechanic_ventilation	0	Count	39	35	74
		% within Mechanic_ventilation	52,7%	47,3%	100,0%
		% within Short_treatment	97,5%	100,0%	98,7%
		% of Total	52,0%	46,7%	98,7%
	1	Count	1	0	1
		% within Mechanic_ventilation	100,0%	0,0%	100,0%
		% within Short_treatment	2,5%	0,0%	1,3%
		% of Total	1,3%	0,0%	1,3%
Total		Count	40	35	75
		% within Mechanic_ventilation	53,3%	46,7%	100,0%
		% within Short_treatment	100,0%	100,0%	100,0%
		% of Total	53,3%	46,7%	100,0%

Chi-Square Tests

	Value	df	Asymp. Sig. (2-sided)	Exact Sig. (2-sided)	Exact Sig. (1-sided)
Pearson Chi-Square	,887[a]	1	,346		
Continuity Correction[b]	,000	1	1,000		
Likelihood Ratio	1,269	1	,260		
Fisher's Exact Test				1,000	,533
Linear-by-Linear Association	,875	1	,350		
N of Valid Cases	75				

a. 2 cells (50,0%) have expected count less than 5. The minimum expected count is ,47.

b. Computed only for a 2x2 table

Risk Estimate

	Value	95% Confidence Interval	
		Lower	Upper
For cohort Short_treatment = ,00	,527	,425	,654
N of Valid Cases	75		

Urinary_catheter * Short_treatment

Crosstab

			Short_treatment		Total
			,00	1,00	
Urinary_catheter	0	Count	30	29	59
		% within Urinary_catheter	50,8%	49,2%	100,0%
		% within Short_treatment	75,0%	82,9%	78,7%
		% of Total	40,0%	38,7%	78,7%
	1	Count	10	6	16
		% within Urinary_catheter	62,5%	37,5%	100,0%
		% within Short_treatment	25,0%	17,1%	21,3%
		% of Total	13,3%	8,0%	21,3%
Total		Count	40	35	75
		% within Urinary_catheter	53,3%	46,7%	100,0%
		% within Short_treatment	100,0%	100,0%	100,0%
		% of Total	53,3%	46,7%	100,0%

Chi-Square Tests

	Value	df	Asymp. Sig. (2-sided)	Exact Sig. (2-sided)	Exact Sig. (1-sided)
Pearson Chi-Square	,687[a]	1	,407		
Continuity Correction[b]	,298	1	,585		
Likelihood Ratio	,694	1	,405		
Fisher's Exact Test				,573	,294
Linear-by-Linear Association	,678	1	,410		
N of Valid Cases	75				

a. 0 cells (0,0%) have expected count less than 5. The minimum expected count is 7,47.

b. Computed only for a 2x2 table

Risk Estimate

	Value	95% Confidence Interval	
		Lower	Upper
Odds Ratio for Urinary_catheter (0 / 1)	,621	,200	1,928
For cohort Short_treatment = ,00	,814	,516	1,282
For cohort Short_treatment = 1,00	1,311	,662	2,597
N of Valid Cases	75		

Indwelling_urinary_catheter * Short_treatment

Crosstab

			Short_treatment		Total
			,00	1,00	
Indwelling_urinary_catheter	0	Count	32	31	63
		% within Indwelling_urinary_catheter	50,8%	49,2%	100,0%
		% within Short_treatment	80,0%	88,6%	84,0%
		% of Total	42,7%	41,3%	84,0%
	1	Count	8	4	12
		% within Indwelling_urinary_catheter	66,7%	33,3%	100,0%
		% within Short_treatment	20,0%	11,4%	16,0%
		% of Total	10,7%	5,3%	16,0%
Total		Count	40	35	75
		% within Indwelling_urinary_catheter	53,3%	46,7%	100,0%
		% within Short_treatment	100,0%	100,0%	100,0%
		% of Total	53,3%	46,7%	100,0%

Chi-Square Tests

	Value	df	Asymp. Sig. (2-sided)	Exact Sig. (2-sided)	Exact Sig. (1-sided)
Pearson Chi-Square	1,020[a]	1	,312		
Continuity Correction[b]	,482	1	,487		
Likelihood Ratio	1,041	1	,307		
Fisher's Exact Test				,360	,245
Linear-by-Linear Association	1,007	1	,316		
N of Valid Cases	75				

a. 0 cells (0,0%) have expected count less than 5. The minimum expected count is 5,60.

b. Computed only for a 2x2 table

Risk Estimate

	Value	95% Confidence Interval	
		Lower	Upper
Odds Ratio for Indwelling_urinary_catheter (0 / 1)	,516	,141	1,890
For cohort Short_treatment = ,00	,762	,477	1,217
For cohort Short_treatment = 1,00	1,476	,638	3,414
N of Valid Cases	75		

Cognitive_impairment * Short_treatment

Crosstab

			Short_treatment		Total
			,00	1,00	
Cognitive_impairment	0	Count	33	23	56
		% within Cognitive_impairment	58,9%	41,1%	100,0%
		% within Short_treatment	82,5%	65,7%	74,7%
		% of Total	44,0%	30,7%	74,7%
	1	Count	7	12	19
		% within Cognitive_impairment	36,8%	63,2%	100,0%
		% within Short_treatment	17,5%	34,3%	25,3%
		% of Total	9,3%	16,0%	25,3%
Total		Count	40	35	75
		% within Cognitive_impairment	53,3%	46,7%	100,0%
		% within Short_treatment	100,0%	100,0%	100,0%
		% of Total	53,3%	46,7%	100,0%

Chi-Square Tests

	Value	df	Asymp. Sig. (2-sided)	Exact Sig. (2-sided)	Exact Sig. (1-sided)
Pearson Chi-Square	2,781[a]	1	,095		
Continuity Correction[b]	1,964	1	,161		
Likelihood Ratio	2,793	1	,095		
Fisher's Exact Test				,116	,081
Linear-by-Linear Association	2,743	1	,098		
N of Valid Cases	75				

a. 0 cells (0,0%) have expected count less than 5. The minimum expected count is 8,87.

b. Computed only for a 2x2 table

Risk Estimate

	Value	95% Confidence Interval	
		Lower	Upper
Odds Ratio for Cognitive_impairment (0 / 1)	2,460	,841	7,196
For cohort Short_treatment = ,00	1,599	,854	2,997
For cohort Short_treatment = 1,00	,650	,408	1,035
N of Valid Cases	75		

Solid_tumor * Short_treatment

Crosstab

			Short_treatment		Total
			,00	1,00	
Solid_tumor	0	Count	33	32	65
		% within Solid_tumor	50,8%	49,2%	100,0%
		% within Short_treatment	82,5%	91,4%	86,7%
		% of Total	44,0%	42,7%	86,7%
	2	Count	7	3	10
		% within Solid_tumor	70,0%	30,0%	100,0%
		% within Short_treatment	17,5%	8,6%	13,3%
		% of Total	9,3%	4,0%	13,3%
Total		Count	40	35	75
		% within Solid_tumor	53,3%	46,7%	100,0%
		% within Short_treatment	100,0%	100,0%	100,0%
		% of Total	53,3%	46,7%	100,0%

Chi-Square Tests

	Value	df	Asymp. Sig. (2-sided)	Exact Sig. (2-sided)	Exact Sig. (1-sided)
Pearson Chi-Square	1,288[a]	1	,256		
Continuity Correction[b]	,631	1	,427		
Likelihood Ratio	1,327	1	,249		
Fisher's Exact Test				,321	,215
Linear-by-Linear Association	1,271	1	,260		
N of Valid Cases	75				

a. 1 cells (25,0%) have expected count less than 5. The minimum expected count is 4,67.

b. Computed only for a 2x2 table

Risk Estimate

	Value	95% Confidence Interval	
		Lower	Upper
Odds Ratio for Solid_tumor (0 / 2)	,442	,105	1,860
For cohort Short_treatment = ,00	,725	,453	1,162
For cohort Short_treatment = 1,00	1,641	,617	4,365
N of Valid Cases	75		

Metastatic_solid_tumor * Short_treatment

Crosstab

			Short_treatment		Total
			,00	1,00	
Metastatic_solid_tumor	0	Count	39	34	73
		% within Metastatic_solid_tumor	53,4%	46,6%	100,0%
		% within Short_treatment	97,5%	97,1%	97,3%
		% of Total	52,0%	45,3%	97,3%
	6	Count	1	1	2
		% within Metastatic_solid_tumor	50,0%	50,0%	100,0%
		% within Short_treatment	2,5%	2,9%	2,7%
		% of Total	1,3%	1,3%	2,7%
Total		Count	40	35	75
		% within Metastatic_solid_tumor	53,3%	46,7%	100,0%
		% within Short_treatment	100,0%	100,0%	100,0%
		% of Total	53,3%	46,7%	100,0%

Chi-Square Tests

	Value	df	Asymp. Sig. (2-sided)	Exact Sig. (2-sided)	Exact Sig. (1-sided)
Pearson Chi-Square	,009[a]	1	,924		
Continuity Correction[b]	,000	1	1,000		
Likelihood Ratio	,009	1	,924		
Fisher's Exact Test				1,000	,719
Linear-by-Linear Association	,009	1	,924		
N of Valid Cases	75				

a. 2 cells (50,0%) have expected count less than 5. The minimum expected count is ,93.

b. Computed only for a 2x2 table

Risk Estimate

	Value	95% Confidence Interval	
		Lower	Upper
Odds Ratio for Metastatic_solid_tumor (0 / 6)	1,147	,069	19,047
For cohort Short_treatment = ,00	1,068	,263	4,343
For cohort Short_treatment = 1,00	,932	,228	3,806
N of Valid Cases	75		

Lymphoma * Short_treatment

Crosstab

			Short_treatment		
			,00	1,00	Total
Lymphoma	0	Count	38	35	73
		% within Lymphoma	52,1%	47,9%	100,0%
		% within Short_treatment	95,0%	100,0%	97,3%
		% of Total	50,7%	46,7%	97,3%
	2	Count	2	0	2
		% within Lymphoma	100,0%	0,0%	100,0%
		% within Short_treatment	5,0%	0,0%	2,7%
		% of Total	2,7%	0,0%	2,7%
Total		Count	40	35	75
		% within Lymphoma	53,3%	46,7%	100,0%
		% within Short_treatment	100,0%	100,0%	100,0%
		% of Total	53,3%	46,7%	100,0%

Chi-Square Tests

	Value	df	Asymp. Sig. (2-sided)	Exact Sig. (2-sided)	Exact Sig. (1-sided)
Pearson Chi-Square	1,798[a]	1	,180		
Continuity Correction[b]	,388	1	,534		
Likelihood Ratio	2,562	1	,109		
Fisher's Exact Test				,495	,281
Linear-by-Linear Association	1,774	1	,183		
N of Valid Cases	75				

a. 2 cells (50,0%) have expected count less than 5. The minimum expected count is ,93.

b. Computed only for a 2x2 table

Risk Estimate

	Value	95% Confidence Interval	
		Lower	Upper
For cohort Short_treatment = ,00	,521	,418	,649
N of Valid Cases	75		

Leukemia * Short_treatment

Crosstab

			Short_treatment		
			,00	1,00	Total
Leukemia	0	Count	40	35	75
		% within Leukemia	53,3%	46,7%	100,0%
		% within Short_treatment	100,0%	100,0%	100,0%
		% of Total	53,3%	46,7%	100,0%
Total		Count	40	35	75
		% within Leukemia	53,3%	46,7%	100,0%
		% within Short_treatment	100,0%	100,0%	100,0%
		% of Total	53,3%	46,7%	100,0%

Chi-Square Tests

	Value
Pearson Chi-Square	.[a]
N of Valid Cases	75

a. No statistics are computed because Leukemia is a constant.

Risk Estimate

	Value
Odds Ratio for Leukemia (0 / .)	.[a]

a. No statistics are computed because Leukemia is a constant.

Chronic_liver_disease * Short_treatment

Crosstab

			Short_treatment		Total
			,00	1,00	
Chronic_liver_disease	0	Count	34	33	67
		% within Chronic_liver_disease	50,7%	49,3%	100,0%
		% within Short_treatment	85,0%	94,3%	89,3%
		% of Total	45,3%	44,0%	89,3%
	1	Count	6	2	8
		% within Chronic_liver_disease	75,0%	25,0%	100,0%
		% within Short_treatment	15,0%	5,7%	10,7%
		% of Total	8,0%	2,7%	10,7%
Total		Count	40	35	75
		% within Chronic_liver_disease	53,3%	46,7%	100,0%
		% within Short_treatment	100,0%	100,0%	100,0%
		% of Total	53,3%	46,7%	100,0%

Chi-Square Tests

	Value	df	Asymp. Sig. (2-sided)	Exact Sig. (2-sided)	Exact Sig. (1-sided)
Pearson Chi-Square	1,689[a]	1	,194		
Continuity Correction[b]	,855	1	,355		
Likelihood Ratio	1,774	1	,183		
Fisher's Exact Test				,271	,179
Linear-by-Linear Association	1,667	1	,197		
N of Valid Cases	75				

a. 2 cells (50,0%) have expected count less than 5. The minimum expected count is 3,73.

b. Computed only for a 2x2 table

Risk Estimate

	Value	95% Confidence Interval	
		Lower	Upper
Odds Ratio for Chronic_liver_disease (0 / 1)	,343	,065	1,825
For cohort Short_treatment = ,00	,677	,425	1,077
For cohort Short_treatment = 1,00	1,970	,579	6,704
N of Valid Cases	75		

Chronic_liver_disease_without_portal_hypertension * Short_treatment

Crosstab

			Short_treatment		Total
			,00	1,00	
Chronic_liver_disease_without_portal_hypertension	0	Count	35	34	69
		% within Chronic_liver_disease_without_portal_hypertension	50,7%	49,3%	100,0%
		% within Short_treatment	87,5%	97,1%	92,0%
		% of Total	46,7%	45,3%	92,0%
	1	Count	5	1	6
		% within Chronic_liver_disease_without_portal_hypertension	83,3%	16,7%	100,0%
		% within Short_treatment	12,5%	2,9%	8,0%
		% of Total	6,7%	1,3%	8,0%
Total		Count	40	35	75
		% within Chronic_liver_disease_without_portal_hypertension	53,3%	46,7%	100,0%
		% within Short_treatment	100,0%	100,0%	100,0%
		% of Total	53,3%	46,7%	100,0%

Chi-Square Tests

	Value	df	Asymp. Sig. (2-sided)	Exact Sig. (2-sided)	Exact Sig. (1-sided)
Pearson Chi-Square	2,358[a]	1	,125		
Continuity Correction[b]	1,230	1	,267		
Likelihood Ratio	2,592	1	,107		
Fisher's Exact Test				,206	,133
Linear-by-Linear Association	2,327	1	,127		
N of Valid Cases	75				

a. 2 cells (50,0%) have expected count less than 5. The minimum expected count is 2,80.

b. Computed only for a 2x2 table

Risk Estimate

	Value	95% Confidence Interval	
		Lower	Upper
Odds Ratio for Chronic_liver_disease_without_portal_hypertension (0 / 1)	,206	,023	1,855
For cohort Short_treatment = ,00	,609	,397	,933
For cohort Short_treatment = 1,00	2,957	,486	17,978
N of Valid Cases	75		

Chronic_liver_disease_with_portal_hypertension * Short_treatment

Crosstab

			Short_treatment		Total
			,00	1,00	
Chronic_liver_disease_with_portal_hypertension	0	Count	39	34	73
		% within Chronic_liver_disease_with_portal_hypertension	53,4%	46,6%	100,0%
		% within Short_treatment	97,5%	97,1%	97,3%
		% of Total	52,0%	45,3%	97,3%
	3	Count	1	1	2
		% within Chronic_liver_disease_with_portal_hypertension	50,0%	50,0%	100,0%
		% within Short_treatment	2,5%	2,9%	2,7%
		% of Total	1,3%	1,3%	2,7%
Total		Count	40	35	75
		% within Chronic_liver_disease_with_portal_hypertension	53,3%	46,7%	100,0%
		% within Short_treatment	100,0%	100,0%	100,0%
		% of Total	53,3%	46,7%	100,0%

Chi-Square Tests

	Value	df	Asymp. Sig. (2-sided)	Exact Sig. (2-sided)	Exact Sig. (1-sided)
Pearson Chi-Square	,009[a]	1	,924		
Continuity Correction[b]	,000	1	1,000		
Likelihood Ratio	,009	1	,924		
Fisher's Exact Test				1,000	,719
Linear-by-Linear Association	,009	1	,924		
N of Valid Cases	75				

a. 2 cells (50,0%) have expected count less than 5. The minimum expected count is ,93.

b. Computed only for a 2x2 table

Risk Estimate

	Value	95% Confidence Interval	
		Lower	Upper
Odds Ratio for Chronic_liver_disease_with_portal_hypertension (0 / 3)	1,147	,069	19,047
For cohort Short_treatment = ,00	1,068	,263	4,343
For cohort Short_treatment = 1,00	,932	,228	3,806
N of Valid Cases	75		

Connective_tissue_disease * Short_treatment

Crosstab

			Short_treatment		Total
			,00	1,00	
Connective_tissue_disease	0	Count	39	32	71
		% within Connective_tissue_disease	54,9%	45,1%	100,0%
		% within Short_treatment	97,5%	91,4%	94,7%
		% of Total	52,0%	42,7%	94,7%
	1	Count	1	3	4
		% within Connective_tissue_disease	25,0%	75,0%	100,0%
		% within Short_treatment	2,5%	8,6%	5,3%
		% of Total	1,3%	4,0%	5,3%
Total		Count	40	35	75
		% within Connective_tissue_disease	53,3%	46,7%	100,0%
		% within Short_treatment	100,0%	100,0%	100,0%
		% of Total	53,3%	46,7%	100,0%

Chi-Square Tests

	Value	df	Asymp. Sig. (2-sided)	Exact Sig. (2-sided)	Exact Sig. (1-sided)
Pearson Chi-Square	1,363[a]	1	,243		
Continuity Correction[b]	,426	1	,514		
Likelihood Ratio	1,404	1	,236		
Fisher's Exact Test				,334	,258
Linear-by-Linear Association	1,345	1	,246		
N of Valid Cases	75				

a. 2 cells (50,0%) have expected count less than 5. The minimum expected count is 1,87.

b. Computed only for a 2x2 table

Risk Estimate

	Value	95% Confidence Interval	
		Lower	Upper
Odds Ratio for Connective_tissue_disease (0 / 1)	3,656	,363	36,870
For cohort Short_treatment = ,00	2,197	,397	12,153
For cohort Short_treatment = 1,00	,601	,323	1,119
N of Valid Cases	75		

Diabetes_mellitus * Short_treatment

Crosstab

			Short_treatment		Total
			,00	1,00	
Diabetes_mellitus	0	Count	29	22	51
		% within Diabetes_mellitus	56,9%	43,1%	100,0%
		% within Short_treatment	72,5%	62,9%	68,0%
		% of Total	38,7%	29,3%	68,0%
	1	Count	11	13	24
		% within Diabetes_mellitus	45,8%	54,2%	100,0%
		% within Short_treatment	27,5%	37,1%	32,0%
		% of Total	14,7%	17,3%	32,0%
Total		Count	40	35	75
		% within Diabetes_mellitus	53,3%	46,7%	100,0%
		% within Short_treatment	100,0%	100,0%	100,0%
		% of Total	53,3%	46,7%	100,0%

Chi-Square Tests

	Value	df	Asymp. Sig. (2-sided)	Exact Sig. (2-sided)	Exact Sig. (1-sided)
Pearson Chi-Square	,798[a]	1	,372		
Continuity Correction[b]	,416	1	,519		
Likelihood Ratio	,797	1	,372		
Fisher's Exact Test				,459	,259
Linear-by-Linear Association	,787	1	,375		
N of Valid Cases	75				

a. 0 cells (0,0%) have expected count less than 5. The minimum expected count is 11,20.

b. Computed only for a 2x2 table

Risk Estimate

	Value	95% Confidence Interval	
		Lower	Upper
Odds Ratio for Diabetes_mellitus (0 / 1)	1,558	,587	4,133
For cohort Short_treatment = ,00	1,241	,755	2,038
For cohort Short_treatment = 1,00	,796	,491	1,293
N of Valid Cases	75		

DM_with_target_organ_damage * Short_treatment

Crosstab

			Short_treatment		Total
			,00	1,00	
DM_with_target_organ_damage	0	Count	35	32	67
		% within DM_with_target_organ_damage	52,2%	47,8%	100,0%
		% within Short_treatment	87,5%	91,4%	89,3%
		% of Total	46,7%	42,7%	89,3%
	2	Count	5	3	8
		% within DM_with_target_organ_damage	62,5%	37,5%	100,0%
		% within Short_treatment	12,5%	8,6%	10,7%
		% of Total	6,7%	4,0%	10,7%
Total		Count	40	35	75
		% within DM_with_target_organ_damage	53,3%	46,7%	100,0%
		% within Short_treatment	100,0%	100,0%	100,0%
		% of Total	53,3%	46,7%	100,0%

Chi-Square Tests

	Value	df	Asymp. Sig. (2-sided)	Exact Sig. (2-sided)	Exact Sig. (1-sided)
Pearson Chi-Square	,302[a]	1	,582		
Continuity Correction[b]	,031	1	,861		
Likelihood Ratio	,306	1	,580		
Fisher's Exact Test				,716	,434
Linear-by-Linear Association	,298	1	,585		
N of Valid Cases	75				

a. 2 cells (50,0%) have expected count less than 5. The minimum expected count is 3,73.

b. Computed only for a 2x2 table

Risk Estimate

	Value	95% Confidence Interval Lower	Upper
Odds Ratio for DM_with_target_organ_damage (0 / 2)	,656	,145	2,969
For cohort Short_treatment = ,00	,836	,466	1,498
For cohort Short_treatment = 1,00	1,274	,503	3,225
N of Valid Cases	75		

Hypertension * Short_treatment

Crosstab

			Short_treatment ,00	1,00	Total
Hypertension	0	Count	21	10	31
		% within Hypertension	67,7%	32,3%	100,0%
		% within Short_treatment	52,5%	28,6%	41,3%
		% of Total	28,0%	13,3%	41,3%
	1	Count	19	25	44
		% within Hypertension	43,2%	56,8%	100,0%
		% within Short_treatment	47,5%	71,4%	58,7%
		% of Total	25,3%	33,3%	58,7%
Total		Count	40	35	75
		% within Hypertension	53,3%	46,7%	100,0%
		% within Short_treatment	100,0%	100,0%	100,0%
		% of Total	53,3%	46,7%	100,0%

Chi-Square Tests

	Value	df	Asymp. Sig. (2-sided)	Exact Sig. (2-sided)	Exact Sig. (1-sided)
Pearson Chi-Square	4,408[a]	1	,036		
Continuity Correction[b]	3,476	1	,062		
Likelihood Ratio	4,477	1	,034		
Fisher's Exact Test				,059	,031
Linear-by-Linear Association	4,349	1	,037		
N of Valid Cases	75				

a. 0 cells (0,0%) have expected count less than 5. The minimum expected count is 14,47.

b. Computed only for a 2x2 table

Risk Estimate

	Value	95% Confidence Interval Lower	Upper
Odds Ratio for Hypertension (0 / 1)	2,763	1,057	7,221
For cohort Short_treatment = ,00	1,569	1,034	2,380
For cohort Short_treatment = 1,00	,568	,321	1,005
N of Valid Cases	75		

Dyslipidemia * Short_treatment

Crosstab

			Short_treatment		Total
			,00	1,00	
Dyslipidemia	0	Count	25	17	42
		% within Dyslipidemia	59,5%	40,5%	100,0%
		% within Short_treatment	62,5%	48,6%	56,0%
		% of Total	33,3%	22,7%	56,0%
	1	Count	15	18	33
		% within Dyslipidemia	45,5%	54,5%	100,0%
		% within Short_treatment	37,5%	51,4%	44,0%
		% of Total	20,0%	24,0%	44,0%
Total		Count	40	35	75
		% within Dyslipidemia	53,3%	46,7%	100,0%
		% within Short_treatment	100,0%	100,0%	100,0%
		% of Total	53,3%	46,7%	100,0%

Chi-Square Tests

	Value	df	Asymp. Sig. (2-sided)	Exact Sig. (2-sided)	Exact Sig. (1-sided)
Pearson Chi-Square	1,470[a]	1	,225		
Continuity Correction[b]	,959	1	,327		
Likelihood Ratio	1,473	1	,225		
Fisher's Exact Test				,252	,164
Linear-by-Linear Association	1,450	1	,229		
N of Valid Cases	75				

a. 0 cells (0,0%) have expected count less than 5. The minimum expected count is 15,40.

b. Computed only for a 2x2 table

Risk Estimate

	Value	95% Confidence Interval	
		Lower	Upper
Odds Ratio for Dyslipidemia (0 / 1)	1,765	,702	4,435
For cohort Short_treatment = ,00	1,310	,836	2,052
For cohort Short_treatment = 1,00	,742	,459	1,201
N of Valid Cases	75		

Chronic_respiratory_disease * Short_treatment

Crosstab

			Short_treatment		Total
			,00	1,00	
Chronic_respiratory_disease	0	Count	34	30	64
		% within Chronic_respiratory_disease	53,1%	46,9%	100,0%
		% within Short_treatment	85,0%	85,7%	85,3%
		% of Total	45,3%	40,0%	85,3%
	1	Count	6	5	11
		% within Chronic_respiratory_disease	54,5%	45,5%	100,0%
		% within Short_treatment	15,0%	14,3%	14,7%
		% of Total	8,0%	6,7%	14,7%
Total		Count	40	35	75
		% within Chronic_respiratory_disease	53,3%	46,7%	100,0%
		% within Short_treatment	100,0%	100,0%	100,0%
		% of Total	53,3%	46,7%	100,0%

Chi-Square Tests

	Value	df	Asymp. Sig. (2-sided)	Exact Sig. (2-sided)	Exact Sig. (1-sided)
Pearson Chi-Square	,008[a]	1	,930		
Continuity Correction[b]	,000	1	1,000		
Likelihood Ratio	,008	1	,930		
Fisher's Exact Test				1,000	,596
Linear-by-Linear Association	,008	1	,931		
N of Valid Cases	75				

a. 0 cells (0,0%) have expected count less than 5. The minimum expected count is 5,13.

b. Computed only for a 2x2 table

Risk Estimate

	Value	95% Confidence Interval	
		Lower	Upper
Odds Ratio for Chronic_respiratory_disease (0 / 1)	,944	,261	3,412
For cohort Short_treatment = ,00	,974	,542	1,751
For cohort Short_treatment = 1,00	1,031	,513	2,072
N of Valid Cases	75		

COPD * Short_treatment

Crosstab

			Short_treatment		Total
			,00	1,00	
COPD	0	Count	37	33	70
		% within COPD	52,9%	47,1%	100,0%
		% within Short_treatment	92,5%	94,3%	93,3%
		% of Total	49,3%	44,0%	93,3%
	1	Count	3	2	5
		% within COPD	60,0%	40,0%	100,0%
		% within Short_treatment	7,5%	5,7%	6,7%
		% of Total	4,0%	2,7%	6,7%
Total		Count	40	35	75
		% within COPD	53,3%	46,7%	100,0%
		% within Short_treatment	100,0%	100,0%	100,0%
		% of Total	53,3%	46,7%	100,0%

Chi-Square Tests

	Value	df	Asymp. Sig. (2-sided)	Exact Sig. (2-sided)	Exact Sig. (1-sided)
Pearson Chi-Square	,096[a]	1	,757		
Continuity Correction[b]	,000	1	1,000		
Likelihood Ratio	,096	1	,756		
Fisher's Exact Test				1,000	,564
Linear-by-Linear Association	,094	1	,759		
N of Valid Cases	75				

a. 2 cells (50,0%) have expected count less than 5. The minimum expected count is 2,33.

b. Computed only for a 2x2 table

Risk Estimate

	Value	95% Confidence Interval	
		Lower	Upper
Odds Ratio for COPD (0 / 1)	,747	,118	4,752
For cohort Short_treatment = ,00	,881	,417	1,863
For cohort Short_treatment = 1,00	1,179	,392	3,547
N of Valid Cases	75		

Heart_failure * Short_treatment

Crosstab

			Short_treatment		Total
			,00	1,00	
Heart_failure	0	Count	36	27	63
		% within Heart_failure	57,1%	42,9%	100,0%
		% within Short_treatment	90,0%	77,1%	84,0%
		% of Total	48,0%	36,0%	84,0%
	1	Count	4	8	12
		% within Heart_failure	33,3%	66,7%	100,0%
		% within Short_treatment	10,0%	22,9%	16,0%
		% of Total	5,3%	10,7%	16,0%
Total		Count	40	35	75
		% within Heart_failure	53,3%	46,7%	100,0%
		% within Short_treatment	100,0%	100,0%	100,0%
		% of Total	53,3%	46,7%	100,0%

Chi-Square Tests

	Value	df	Asymp. Sig. (2-sided)	Exact Sig. (2-sided)	Exact Sig. (1-sided)
Pearson Chi-Square	2,296[a]	1	,130		
Continuity Correction[b]	1,439	1	,230		
Likelihood Ratio	2,316	1	,128		
Fisher's Exact Test				,206	,115
Linear-by-Linear Association	2,265	1	,132		
N of Valid Cases	75				

a. 0 cells (0,0%) have expected count less than 5. The minimum expected count is 5,60.

b. Computed only for a 2x2 table

Risk Estimate

	Value	95% Confidence Interval	
		Lower	Upper
Odds Ratio for Heart_failure (0 / 1)	2,667	,727	9,783
For cohort Short_treatment = ,00	1,714	,749	3,924
For cohort Short_treatment = 1,00	,643	,393	1,051
N of Valid Cases	75		

Myocardial_infarction * Short_treatment

Crosstab

			Short_treatment		Total
			,00	1,00	
Myocardial_infarction	0	Count	37	29	66
		% within Myocardial_infarction	56,1%	43,9%	100,0%
		% within Short_treatment	92,5%	82,9%	88,0%
		% of Total	49,3%	38,7%	88,0%
	1	Count	3	6	9
		% within Myocardial_infarction	33,3%	66,7%	100,0%
		% within Short_treatment	7,5%	17,1%	12,0%
		% of Total	4,0%	8,0%	12,0%
Total		Count	40	35	75
		% within Myocardial_infarction	53,3%	46,7%	100,0%
		% within Short_treatment	100,0%	100,0%	100,0%
		% of Total	53,3%	46,7%	100,0%

Chi-Square Tests

	Value	df	Asymp. Sig. (2-sided)	Exact Sig. (2-sided)	Exact Sig. (1-sided)
Pearson Chi-Square	1,644[a]	1	,200		
Continuity Correction[b]	,857	1	,354		
Likelihood Ratio	1,658	1	,198		
Fisher's Exact Test				,289	,178
Linear-by-Linear Association	1,622	1	,203		
N of Valid Cases	75				

a. 2 cells (50,0%) have expected count less than 5. The minimum expected count is 4,20.

b. Computed only for a 2x2 table

Risk Estimate

	Value	95% Confidence Interval	
		Lower	Upper
Odds Ratio for Myocardial_infarction (0 / 1)	2,552	,587	11,083
For cohort Short_treatment = ,00	1,682	,652	4,341
For cohort Short_treatment = 1,00	,659	,385	1,127
N of Valid Cases	75		

Peripheral_arterial_disease * Short_treatment

Crosstab

			Short_treatment		Total
			,00	1,00	
Peripheral_arterial_disease	0	Count	34	33	67
		% within Peripheral_arterial_disease	50,7%	49,3%	100,0%
		% within Short_treatment	85,0%	94,3%	89,3%
		% of Total	45,3%	44,0%	89,3%
	1	Count	6	2	8
		% within Peripheral_arterial_disease	75,0%	25,0%	100,0%
		% within Short_treatment	15,0%	5,7%	10,7%
		% of Total	8,0%	2,7%	10,7%
Total		Count	40	35	75
		% within Peripheral_arterial_disease	53,3%	46,7%	100,0%
		% within Short_treatment	100,0%	100,0%	100,0%
		% of Total	53,3%	46,7%	100,0%

Chi-Square Tests

	Value	df	Asymp. Sig. (2-sided)	Exact Sig. (2-sided)	Exact Sig. (1-sided)
Pearson Chi-Square	1,689[a]	1	,194		
Continuity Correction[b]	,855	1	,355		
Likelihood Ratio	1,774	1	,183		
Fisher's Exact Test				,271	,179
Linear-by-Linear Association	1,667	1	,197		
N of Valid Cases	75				

a. 2 cells (50,0%) have expected count less than 5. The minimum expected count is 3,73.

b. Computed only for a 2x2 table

Risk Estimate

	Value	95% Confidence Interval	
		Lower	Upper
Odds Ratio for Peripheral_arterial_disease (0 / 1)	,343	,065	1,825
For cohort Short_treatment = ,00	,677	,425	1,077
For cohort Short_treatment = 1,00	1,970	,579	6,704
N of Valid Cases	75		

Cerebrovascular_disease * Short_treatment

Crosstab

			Short_treatment		Total
			,00	1,00	
Cerebrovascular_disease	0	Count	31	28	59
		% within Cerebrovascular_disease	52,5%	47,5%	100,0%
		% within Short_treatment	77,5%	80,0%	78,7%
		% of Total	41,3%	37,3%	78,7%
	1	Count	9	7	16
		% within Cerebrovascular_disease	56,3%	43,8%	100,0%
		% within Short_treatment	22,5%	20,0%	21,3%
		% of Total	12,0%	9,3%	21,3%
Total		Count	40	35	75
		% within Cerebrovascular_disease	53,3%	46,7%	100,0%
		% within Short_treatment	100,0%	100,0%	100,0%
		% of Total	53,3%	46,7%	100,0%

Chi-Square Tests

	Value	df	Asymp. Sig. (2-sided)	Exact Sig. (2-sided)	Exact Sig. (1-sided)
Pearson Chi-Square	,070[a]	1	,792		
Continuity Correction[b]	,000	1	1,000		
Likelihood Ratio	,070	1	,792		
Fisher's Exact Test				1,000	,509
Linear-by-Linear Association	,069	1	,793		
N of Valid Cases	75				

a. 0 cells (0,0%) have expected count less than 5. The minimum expected count is 7,47.

b. Computed only for a 2x2 table

Risk Estimate

	Value	95% Confidence Interval	
		Lower	Upper
Odds Ratio for Cerebrovascular_disease (0 / 1)	,861	,283	2,618
For cohort Short_treatment = ,00	,934	,569	1,533
For cohort Short_treatment = 1,00	1,085	,585	2,011
N of Valid Cases	75		

Hemiplegia * Short_treatment

Crosstab

			Short_treatment		Total
			,00	1,00	
Hemiplegia	0	Count	32	33	65
		% within Hemiplegia	49,2%	50,8%	100,0%
		% within Short_treatment	80,0%	94,3%	86,7%
		% of Total	42,7%	44,0%	86,7%
	2	Count	8	2	10
		% within Hemiplegia	80,0%	20,0%	100,0%
		% within Short_treatment	20,0%	5,7%	13,3%
		% of Total	10,7%	2,7%	13,3%
Total		Count	40	35	75
		% within Hemiplegia	53,3%	46,7%	100,0%
		% within Short_treatment	100,0%	100,0%	100,0%
		% of Total	53,3%	46,7%	100,0%

Chi-Square Tests

	Value	df	Asymp. Sig. (2-sided)	Exact Sig. (2-sided)	Exact Sig. (1-sided)
Pearson Chi-Square	3,297[a]	1	,069		
Continuity Correction[b]	2,176	1	,140		
Likelihood Ratio	3,537	1	,060		
Fisher's Exact Test				,094	,068
Linear-by-Linear Association	3,253	1	,071		
N of Valid Cases	75				

a. 1 cells (25,0%) have expected count less than 5. The minimum expected count is 4,67.

b. Computed only for a 2x2 table

Risk Estimate

	Value	95% Confidence Interval	
		Lower	Upper
Odds Ratio for Hemiplegia (0 / 2)	,242	,048	1,230
For cohort Short_treatment = ,00	,615	,414	,915
For cohort Short_treatment = 1,00	2,538	,718	8,971
N of Valid Cases	75		

Gastroduodenal_ulcer * Short_treatment

Crosstab

			Short_treatment		Total
			,00	1,00	
Gastroduodenal_ulcer	0	Count	38	31	69
		% within Gastroduodenal_ulcer	55,1%	44,9%	100,0%
		% within Short_treatment	95,0%	88,6%	92,0%
		% of Total	50,7%	41,3%	92,0%
	1	Count	2	4	6
		% within Gastroduodenal_ulcer	33,3%	66,7%	100,0%
		% within Short_treatment	5,0%	11,4%	8,0%
		% of Total	2,7%	5,3%	8,0%
Total		Count	40	35	75
		% within Gastroduodenal_ulcer	53,3%	46,7%	100,0%
		% within Short_treatment	100,0%	100,0%	100,0%
		% of Total	53,3%	46,7%	100,0%

Chi-Square Tests

	Value	df	Asymp. Sig. (2-sided)	Exact Sig. (2-sided)	Exact Sig. (1-sided)
Pearson Chi-Square	1,048[a]	1	,306		
Continuity Correction[b]	,357	1	,550		
Likelihood Ratio	1,057	1	,304		
Fisher's Exact Test				,409	,275
Linear-by-Linear Association	1,034	1	,309		
N of Valid Cases	75				

a. 2 cells (50,0%) have expected count less than 5. The minimum expected count is 2,80.

b. Computed only for a 2x2 table

Risk Estimate

	Value	95% Confidence Interval	
		Lower	Upper
Odds Ratio for Gastroduodenal_ulcer (0 / 1)	2,452	,421	14,284
For cohort Short_treatment = ,00	1,652	,522	5,226
For cohort Short_treatment = 1,00	,674	,361	1,257
N of Valid Cases	75		

Chronic_kidney_disease * Short_treatment

Crosstab

			Short_treatment		Total
			,00	1,00	
Chronic_kidney_disease	0	Count	31	24	55
		% within Chronic_kidney_disease	56,4%	43,6%	100,0%
		% within Short_treatment	77,5%	68,6%	73,3%
		% of Total	41,3%	32,0%	73,3%
	1	Count	9	11	20
		% within Chronic_kidney_disease	45,0%	55,0%	100,0%
		% within Short_treatment	22,5%	31,4%	26,7%
		% of Total	12,0%	14,7%	26,7%
Total		Count	40	35	75
		% within Chronic_kidney_disease	53,3%	46,7%	100,0%
		% within Short_treatment	100,0%	100,0%	100,0%
		% of Total	53,3%	46,7%	100,0%

Chi-Square Tests

	Value	df	Asymp. Sig. (2-sided)	Exact Sig. (2-sided)	Exact Sig. (1-sided)
Pearson Chi-Square	,761[a]	1	,383		
Continuity Correction[b]	,373	1	,541		
Likelihood Ratio	,760	1	,383		
Fisher's Exact Test				,439	,270
Linear-by-Linear Association	,751	1	,386		
N of Valid Cases	75				

a. 0 cells (0,0%) have expected count less than 5. The minimum expected count is 9,33.

b. Computed only for a 2x2 table

Risk Estimate

	Value	95% Confidence Interval	
		Lower	Upper
Odds Ratio for Chronic_kidney_disease (0 / 1)	1,579	,564	4,420
For cohort Short_treatment = ,00	1,253	,732	2,144
For cohort Short_treatment = 1,00	,793	,482	1,305
N of Valid Cases	75		

Moderate_severe_chronic_kidney_disease * Short_treatment

Crosstab

			Short_treatment		Total
			,00	1,00	
Moderate_severe_chronic_kidney_disease	0	Count	38	28	66
		% within Moderate_severe_chronic_kidney_disease	57,6%	42,4%	100,0%
		% within Short_treatment	95,0%	80,0%	88,0%
		% of Total	50,7%	37,3%	88,0%
	2	Count	2	7	9
		% within Moderate_severe_chronic_kidney_disease	22,2%	77,8%	100,0%
		% within Short_treatment	5,0%	20,0%	12,0%
		% of Total	2,7%	9,3%	12,0%
Total		Count	40	35	75
		% within Moderate_severe_chronic_kidney_disease	53,3%	46,7%	100,0%
		% within Short_treatment	100,0%	100,0%	100,0%
		% of Total	53,3%	46,7%	100,0%

Chi-Square Tests

	Value	df	Asymp. Sig. (2-sided)	Exact Sig. (2-sided)	Exact Sig. (1-sided)
Pearson Chi-Square	3,977[a]	1	,046		
Continuity Correction[b]	2,684	1	,101		
Likelihood Ratio	4,129	1	,042		
Fisher's Exact Test				,073	,050
Linear-by-Linear Association	3,924	1	,048		
N of Valid Cases	75				

a. 2 cells (50,0%) have expected count less than 5. The minimum expected count is 4,20.

b. Computed only for a 2x2 table

Risk Estimate

	Value	95% Confidence Interval	
		Lower	Upper
Odds Ratio for Moderate_severe_chronic_kidney_disease (0 / 2)	4,750	,916	24,623
For cohort Short_treatment = ,00	2,591	,750	8,950
For cohort Short_treatment = 1,00	,545	,348	,854
N of Valid Cases	75		

Dyalisis * Short_treatment

Crosstab

			Short_treatment		Total
			,00	1,00	
Dyalisis	0	Count	40	35	75
		% within Dyalisis	53,3%	46,7%	100,0%
		% within Short_treatment	100,0%	100,0%	100,0%
		% of Total	53,3%	46,7%	100,0%
Total		Count	40	35	75
		% within Dyalisis	53,3%	46,7%	100,0%
		% within Short_treatment	100,0%	100,0%	100,0%
		% of Total	53,3%	46,7%	100,0%

Chi-Square Tests

	Value
Pearson Chi-Square	.[a]
N of Valid Cases	75

a. No statistics are computed because Dyalisis is a constant.

Risk Estimate

	Value
Odds Ratio for Dyalisis (0 / .)	.[a]

a. No statistics are computed because Dyalisis is a constant.

HIV * Short_treatment

Crosstab

			Short_treatment		Total
			,00	1,00	
HIV	0	Count	40	35	75
		% within HIV	53,3%	46,7%	100,0%
		% within Short_treatment	100,0%	100,0%	100,0%
		% of Total	53,3%	46,7%	100,0%
Total		Count	40	35	75
		% within HIV	53,3%	46,7%	100,0%
		% within Short_treatment	100,0%	100,0%	100,0%
		% of Total	53,3%	46,7%	100,0%

Chi-Square Tests

	Value
Pearson Chi-Square	.[a]
N of Valid Cases	75

a. No statistics are computed because HIV is a constant.

Risk Estimate

	Value
Odds Ratio for HIV (0 / .)	.[a]

a. No statistics are computed because HIV is a constant.

AIDS * Short_treatment

Crosstab

			Short_treatment		Total
			,00	1,00	
AIDS	0	Count	40	35	75
		% within AIDS	53,3%	46,7%	100,0%
		% within Short_treatment	100,0%	100,0%	100,0%
		% of Total	53,3%	46,7%	100,0%
Total		Count	40	35	75
		% within AIDS	53,3%	46,7%	100,0%
		% within Short_treatment	100,0%	100,0%	100,0%
		% of Total	53,3%	46,7%	100,0%

Chi-Square Tests

	Value
Pearson Chi-Square	.[a]
N of Valid Cases	75

a. No statistics are computed because AIDS is a constant.

Risk Estimate

	Value
Odds Ratio for AIDS (0 / .)	.[a]

a. No statistics are computed because AIDS is a constant.

Neutrophil_below_1000 * Short_treatment

Crosstab

			Short_treatment		Total
			,00	1,00	
Neutrophil_below_1000	0	Count	40	35	75
		% within Neutrophil_below_1000	53,3%	46,7%	100,0%
		% within Short_treatment	100,0%	100,0%	100,0%
		% of Total	53,3%	46,7%	100,0%
Total		Count	40	35	75
		% within Neutrophil_below_1000	53,3%	46,7%	100,0%
		% within Short_treatment	100,0%	100,0%	100,0%
		% of Total	53,3%	46,7%	100,0%

Chi-Square Tests

	Value
Pearson Chi-Square	.[a]
N of Valid Cases	75

a. No statistics are computed because Neutrophil_below_1000 is a constant.

Risk Estimate

	Value
Odds Ratio for Neutrophil_below_1000 (0 / .)	.[a]

a. No statistics are computed because Neutrophil_below_1000 is a constant.

Neutrophil_below_500 * Short_treatment

Crosstab

			Short_treatment		Total
			,00	1,00	
Neutrophil_below_500	0	Count	40	35	75
		% within Neutrophil_below_500	53,3%	46,7%	100,0%
		% within Short_treatment	100,0%	100,0%	100,0%
		% of Total	53,3%	46,7%	100,0%
Total		Count	40	35	75
		% within Neutrophil_below_500	53,3%	46,7%	100,0%
		% within Short_treatment	100,0%	100,0%	100,0%
		% of Total	53,3%	46,7%	100,0%

Chi-Square Tests

	Value
Pearson Chi-Square	.[a]
N of Valid Cases	75

a. No statistics are computed because Neutrophil_below_500 is a constant.

Risk Estimate

	Value
Odds Ratio for Neutrophil_below_500 (0 / .)	.[a]

a. No statistics are computed because Neutrophil_below_500 is a constant.

Corticosteroids * Short_treatment

Crosstab

			Short_treatment		Total
			,00	1,00	
Corticosteroids	0	Count	39	32	71
		% within Corticosteroids	54,9%	45,1%	100,0%
		% within Short_treatment	97,5%	91,4%	94,7%
		% of Total	52,0%	42,7%	94,7%
	1	Count	1	3	4
		% within Corticosteroids	25,0%	75,0%	100,0%
		% within Short_treatment	2,5%	8,6%	5,3%
		% of Total	1,3%	4,0%	5,3%
Total		Count	40	35	75
		% within Corticosteroids	53,3%	46,7%	100,0%
		% within Short_treatment	100,0%	100,0%	100,0%
		% of Total	53,3%	46,7%	100,0%

Chi-Square Tests

	Value	df	Asymp. Sig. (2-sided)	Exact Sig. (2-sided)	Exact Sig. (1-sided)
Pearson Chi-Square	1,363[a]	1	,243		
Continuity Correction[b]	,426	1	,514		
Likelihood Ratio	1,404	1	,236		
Fisher's Exact Test				,334	,258
Linear-by-Linear Association	1,345	1	,246		
N of Valid Cases	75				

a. 2 cells (50,0%) have expected count less than 5. The minimum expected count is 1,87.

b. Computed only for a 2x2 table

Risk Estimate

	Value	95% Confidence Interval	
		Lower	Upper
Odds Ratio for Corticosteroids (0 / 1)	3,656	,363	36,870
For cohort Short_treatment = ,00	2,197	,397	12,153
For cohort Short_treatment = 1,00	,601	,323	1,119
N of Valid Cases	75		

Transplant * Short_treatment

Crosstab

			Short_treatment		Total
			,00	1,00	
Transplant	0	Count	40	33	73
		% within Transplant	54,8%	45,2%	100,0%
		% within Short_treatment	100,0%	97,1%	98,6%
		% of Total	54,1%	44,6%	98,6%
	1	Count	0	1	1
		% within Transplant	0,0%	100,0%	100,0%
		% within Short_treatment	0,0%	2,9%	1,4%
		% of Total	0,0%	1,4%	1,4%
Total		Count	40	34	74
		% within Transplant	54,1%	45,9%	100,0%
		% within Short_treatment	100,0%	100,0%	100,0%
		% of Total	54,1%	45,9%	100,0%

Chi-Square Tests

	Value	df	Asymp. Sig. (2-sided)	Exact Sig. (2-sided)	Exact Sig. (1-sided)
Pearson Chi-Square	1,193[a]	1	,275		
Continuity Correction[b]	,007	1	,935		
Likelihood Ratio	1,572	1	,210		
Fisher's Exact Test				,459	,459
Linear-by-Linear Association	1,176	1	,278		
N of Valid Cases	74				

a. 2 cells (50,0%) have expected count less than 5. The minimum expected count is ,46.

b. Computed only for a 2x2 table

Risk Estimate

	Value	95% Confidence Interval	
		Lower	Upper
For cohort Short_treatment = 1,00	,452	,351	,582
N of Valid Cases	74		

Immunosuppression * Short_treatment

Crosstab

			Short_treatment		Total
			,00	1,00	
Immunosuppression	0	Count	39	32	71
		% within Immunosuppression	54,9%	45,1%	100,0%
		% within Short_treatment	97,5%	91,4%	94,7%
		% of Total	52,0%	42,7%	94,7%
	1	Count	1	3	4
		% within Immunosuppression	25,0%	75,0%	100,0%
		% within Short_treatment	2,5%	8,6%	5,3%
		% of Total	1,3%	4,0%	5,3%
Total		Count	40	35	75
		% within Immunosuppression	53,3%	46,7%	100,0%
		% within Short_treatment	100,0%	100,0%	100,0%
		% of Total	53,3%	46,7%	100,0%

Chi-Square Tests

	Value	df	Asymp. Sig. (2-sided)	Exact Sig. (2-sided)	Exact Sig. (1-sided)
Pearson Chi-Square	1,363[a]	1	,243		
Continuity Correction[b]	,426	1	,514		
Likelihood Ratio	1,404	1	,236		
Fisher's Exact Test				,334	,258
Linear-by-Linear Association	1,345	1	,246		
N of Valid Cases	75				

a. 2 cells (50,0%) have expected count less than 5. The minimum expected count is 1,87.

b. Computed only for a 2x2 table

Risk Estimate

	Value	95% Confidence Interval	
		Lower	Upper
Odds Ratio for Immunosuppression (0 / 1)	3,656	,363	36,870
For cohort Short_treatment = ,00	2,197	,397	12,153
For cohort Short_treatment = 1,00	,601	,323	1,119
N of Valid Cases	75		

Leukocytosis * Short_treatment

Crosstab

			Short_treatment		Total
			,00	1,00	
Leukocytosis	0	Count	26	25	51
		% within Leukocytosis	51,0%	49,0%	100,0%
		% within Short_treatment	66,7%	78,1%	71,8%
		% of Total	36,6%	35,2%	71,8%
	1	Count	13	7	20
		% within Leukocytosis	65,0%	35,0%	100,0%
		% within Short_treatment	33,3%	21,9%	28,2%
		% of Total	18,3%	9,9%	28,2%
Total		Count	39	32	71
		% within Leukocytosis	54,9%	45,1%	100,0%
		% within Short_treatment	100,0%	100,0%	100,0%
		% of Total	54,9%	45,1%	100,0%

Chi-Square Tests

	Value	df	Asymp. Sig. (2-sided)	Exact Sig. (2-sided)	Exact Sig. (1-sided)
Pearson Chi-Square	1,141[a]	1	,286		
Continuity Correction[b]	,645	1	,422		
Likelihood Ratio	1,156	1	,282		
Fisher's Exact Test				,306	,212
Linear-by-Linear Association	1,124	1	,289		
N of Valid Cases	71				

a. 0 cells (0,0%) have expected count less than 5. The minimum expected count is 9,01.

b. Computed only for a 2x2 table

Risk Estimate

	Value	95% Confidence Interval	
		Lower	Upper
Odds Ratio for Leukocytosis (0 / 1)	,560	,192	1,633
For cohort Short_treatment = ,00	,784	,516	1,193
For cohort Short_treatment = 1,00	1,401	,724	2,709
N of Valid Cases	71		

Leukopenia * Short_treatment

Crosstab

			Short_treatment		Total
			,00	1,00	
Leukopenia	0	Count	39	30	69
		% within Leukopenia	56,5%	43,5%	100,0%
		% within Short_treatment	100,0%	93,8%	97,2%
		% of Total	54,9%	42,3%	97,2%
	1	Count	0	2	2
		% within Leukopenia	0,0%	100,0%	100,0%
		% within Short_treatment	0,0%	6,3%	2,8%
		% of Total	0,0%	2,8%	2,8%
Total		Count	39	32	71
		% within Leukopenia	54,9%	45,1%	100,0%
		% within Short_treatment	100,0%	100,0%	100,0%
		% of Total	54,9%	45,1%	100,0%

Chi-Square Tests

	Value	df	Asymp. Sig. (2-sided)	Exact Sig. (2-sided)	Exact Sig. (1-sided)
Pearson Chi-Square	2,508[a]	1	,113		
Continuity Correction[b]	,745	1	,388		
Likelihood Ratio	3,259	1	,071		
Fisher's Exact Test				,200	,200
Linear-by-Linear Association	2,473	1	,116		
N of Valid Cases	71				

a. 2 cells (50,0%) have expected count less than 5. The minimum expected count is ,90.

b. Computed only for a 2x2 table

Risk Estimate

	Value	95% Confidence Interval	
		Lower	Upper
For cohort Short_treatment = 1,00	,435	,332	,569
N of Valid Cases	71		

Thrombopenia * Short_treatment

Crosstab

			Short_treatment		Total
			,00	1,00	
Thrombopenia	0	Count	36	29	65
		% within Thrombopenia	55,4%	44,6%	100,0%
		% within Short_treatment	92,3%	90,6%	91,5%
		% of Total	50,7%	40,8%	91,5%
	1	Count	3	3	6
		% within Thrombopenia	50,0%	50,0%	100,0%
		% within Short_treatment	7,7%	9,4%	8,5%
		% of Total	4,2%	4,2%	8,5%
Total		Count	39	32	71
		% within Thrombopenia	54,9%	45,1%	100,0%
		% within Short_treatment	100,0%	100,0%	100,0%
		% of Total	54,9%	45,1%	100,0%

Chi-Square Tests

	Value	df	Asymp. Sig. (2-sided)	Exact Sig. (2-sided)	Exact Sig. (1-sided)
Pearson Chi-Square	,064[a]	1	,800		
Continuity Correction[b]	,000	1	1,000		
Likelihood Ratio	,064	1	,800		
Fisher's Exact Test				1,000	,564
Linear-by-Linear Association	,063	1	,801		
N of Valid Cases	71				

a. 2 cells (50,0%) have expected count less than 5. The minimum expected count is 2,70.

b. Computed only for a 2x2 table

Risk Estimate

	Value	95% Confidence Interval	
		Lower	Upper
Odds Ratio for Thrombopenia (0 / 1)	1,241	,233	6,617
For cohort Short_treatment = ,00	1,108	,483	2,539
For cohort Short_treatment = 1,00	,892	,383	2,077
N of Valid Cases	71		

Renal_failure * Short_treatment

Crosstab

			Short_treatment		Total
			,00	1,00	
Renal_failure	0	Count	25	15	40
		% within Renal_failure	62,5%	37,5%	100,0%
		% within Short_treatment	64,1%	46,9%	56,3%
		% of Total	35,2%	21,1%	56,3%
	1	Count	14	17	31
		% within Renal_failure	45,2%	54,8%	100,0%
		% within Short_treatment	35,9%	53,1%	43,7%
		% of Total	19,7%	23,9%	43,7%
Total		Count	39	32	71
		% within Renal_failure	54,9%	45,1%	100,0%
		% within Short_treatment	100,0%	100,0%	100,0%
		% of Total	54,9%	45,1%	100,0%

Chi-Square Tests

	Value	df	Asymp. Sig. (2-sided)	Exact Sig. (2-sided)	Exact Sig. (1-sided)
Pearson Chi-Square	2,121[a]	1	,145		
Continuity Correction[b]	1,478	1	,224		
Likelihood Ratio	2,126	1	,145		
Fisher's Exact Test				,159	,112
Linear-by-Linear Association	2,091	1	,148		
N of Valid Cases	71				

a. 0 cells (0,0%) have expected count less than 5. The minimum expected count is 13,97.

b. Computed only for a 2x2 table

Risk Estimate

	Value	95% Confidence Interval	
		Lower	Upper
Odds Ratio for Renal_failure (0 / 1)	2,024	,780	5,254
For cohort Short_treatment = ,00	1,384	,877	2,184
For cohort Short_treatment = 1,00	,684	,410	1,141
N of Valid Cases	71		

Recurrent_UTI * Short_treatment

Crosstab

			Short_treatment		Total
			,00	1,00	
Recurrent_UTI	0	Count	33	27	60
		% within Recurrent_UTI	55,0%	45,0%	100,0%
		% within Short_treatment	82,5%	79,4%	81,1%
		% of Total	44,6%	36,5%	81,1%
	1	Count	7	7	14
		% within Recurrent_UTI	50,0%	50,0%	100,0%
		% within Short_treatment	17,5%	20,6%	18,9%
		% of Total	9,5%	9,5%	18,9%
Total		Count	40	34	74
		% within Recurrent_UTI	54,1%	45,9%	100,0%
		% within Short_treatment	100,0%	100,0%	100,0%
		% of Total	54,1%	45,9%	100,0%

Chi-Square Tests

	Value	df	Asymp. Sig. (2-sided)	Exact Sig. (2-sided)	Exact Sig. (1-sided)
Pearson Chi-Square	,114[a]	1	,735		
Continuity Correction[b]	,002	1	,968		
Likelihood Ratio	,114	1	,736		
Fisher's Exact Test				,773	,482
Linear-by-Linear Association	,113	1	,737		
N of Valid Cases	74				

a. 0 cells (0,0%) have expected count less than 5. The minimum expected count is 6,43.

b. Computed only for a 2x2 table

Risk Estimate

	Value	95% Confidence Interval	
		Lower	Upper
Odds Ratio for Recurrent_UTI (0 / 1)	1,222	,381	3,917
For cohort Short_treatment = ,00	1,100	,621	1,948
For cohort Short_treatment = 1,00	,900	,497	1,630
N of Valid Cases	74		

Community * Short_treatment

Crosstab

			Short_treatment		
			,00	1,00	Total
Community	0	Count	22	21	43
		% within Community	51,2%	48,8%	100,0%
		% within Short_treatment	55,0%	60,0%	57,3%
		% of Total	29,3%	28,0%	57,3%
	1	Count	18	14	32
		% within Community	56,3%	43,8%	100,0%
		% within Short_treatment	45,0%	40,0%	42,7%
		% of Total	24,0%	18,7%	42,7%
Total		Count	40	35	75
		% within Community	53,3%	46,7%	100,0%
		% within Short_treatment	100,0%	100,0%	100,0%
		% of Total	53,3%	46,7%	100,0%

Chi-Square Tests

	Value	df	Asymp. Sig. (2-sided)	Exact Sig. (2-sided)	Exact Sig. (1-sided)
Pearson Chi-Square	,191[a]	1	,662		
Continuity Correction[b]	,041	1	,839		
Likelihood Ratio	,191	1	,662		
Fisher's Exact Test				,815	,420
Linear-by-Linear Association	,188	1	,664		
N of Valid Cases	75				

a. 0 cells (0,0%) have expected count less than 5. The minimum expected count is 14,93.

b. Computed only for a 2x2 table

Risk Estimate

		95% Confidence Interval	
	Value	Lower	Upper
Odds Ratio for Community (0 / 1)	,815	,325	2,043
For cohort Short_treatment = ,00	,910	,596	1,388
For cohort Short_treatment = 1,00	1,116	,678	1,837
N of Valid Cases	75		

Healthcare_associated * Short_treatment

Crosstab

			Short_treatment		Total
			,00	1,00	
Healthcare_associated	0	Count	25	16	41
		% within Healthcare_associated	61,0%	39,0%	100,0%
		% within Short_treatment	62,5%	45,7%	54,7%
		% of Total	33,3%	21,3%	54,7%
	1	Count	15	19	34
		% within Healthcare_associated	44,1%	55,9%	100,0%
		% within Short_treatment	37,5%	54,3%	45,3%
		% of Total	20,0%	25,3%	45,3%
Total		Count	40	35	75
		% within Healthcare_associated	53,3%	46,7%	100,0%
		% within Short_treatment	100,0%	100,0%	100,0%
		% of Total	53,3%	46,7%	100,0%

Chi-Square Tests

	Value	df	Asymp. Sig. (2-sided)	Exact Sig. (2-sided)	Exact Sig. (1-sided)
Pearson Chi-Square	2,122[a]	1	,145		
Continuity Correction[b]	1,499	1	,221		
Likelihood Ratio	2,130	1	,144		
Fisher's Exact Test				,169	,110
Linear-by-Linear Association	2,094	1	,148		
N of Valid Cases	75				

a. 0 cells (0,0%) have expected count less than 5. The minimum expected count is 15,87.

b. Computed only for a 2x2 table

Risk Estimate

	Value	95% Confidence Interval	
		Lower	Upper
Odds Ratio for Healthcare_associated (0 / 1)	1,979	,786	4,981
For cohort Short_treatment = ,00	1,382	,881	2,169
For cohort Short_treatment = 1,00	,698	,430	1,135
N of Valid Cases	75		

Nosocomial * Short_treatment

Crosstab

			Short_treatment		Total
			,00	1,00	
Nosocomial	0	Count	33	33	66
		% within Nosocomial	50,0%	50,0%	100,0%
		% within Short_treatment	82,5%	94,3%	88,0%
		% of Total	44,0%	44,0%	88,0%
	1	Count	7	2	9
		% within Nosocomial	77,8%	22,2%	100,0%
		% within Short_treatment	17,5%	5,7%	12,0%
		% of Total	9,3%	2,7%	12,0%
Total		Count	40	35	75
		% within Nosocomial	53,3%	46,7%	100,0%
		% within Short_treatment	100,0%	100,0%	100,0%
		% of Total	53,3%	46,7%	100,0%

Chi-Square Tests

	Value	df	Asymp. Sig. (2-sided)	Exact Sig. (2-sided)	Exact Sig. (1-sided)
Pearson Chi-Square	2,455[a]	1	,117		
Continuity Correction[b]	1,466	1	,226		
Likelihood Ratio	2,608	1	,106		
Fisher's Exact Test				,162	,112
Linear-by-Linear Association	2,423	1	,120		
N of Valid Cases	75				

a. 2 cells (50,0%) have expected count less than 5. The minimum expected count is 4,20.

b. Computed only for a 2x2 table

Risk Estimate

	Value	95% Confidence Interval	
		Lower	Upper
Odds Ratio for Nosocomial (0 / 1)	,286	,055	1,479
For cohort Short_treatment = ,00	,643	,421	,983
For cohort Short_treatment = 1,00	2,250	,647	7,821
N of Valid Cases	75		

Nursing_home * Short_treatment

Crosstab

			Short_treatment		Total
			,00	1,00	
Nursing_home	0	Count	38	30	68
		% within Nursing_home	55,9%	44,1%	100,0%
		% within Short_treatment	95,0%	85,7%	90,7%
		% of Total	50,7%	40,0%	90,7%
	1	Count	2	5	7
		% within Nursing_home	28,6%	71,4%	100,0%
		% within Short_treatment	5,0%	14,3%	9,3%
		% of Total	2,7%	6,7%	9,3%
Total		Count	40	35	75
		% within Nursing_home	53,3%	46,7%	100,0%
		% within Short_treatment	100,0%	100,0%	100,0%
		% of Total	53,3%	46,7%	100,0%

Chi-Square Tests

	Value	df	Asymp. Sig. (2-sided)	Exact Sig. (2-sided)	Exact Sig. (1-sided)
Pearson Chi-Square	1,902[a]	1	,168		
Continuity Correction[b]	,963	1	,326		
Likelihood Ratio	1,938	1	,164		
Fisher's Exact Test				,241	,164
Linear-by-Linear Association	1,877	1	,171		
N of Valid Cases	75				

a. 2 cells (50,0%) have expected count less than 5. The minimum expected count is 3,27.

b. Computed only for a 2x2 table

Risk Estimate

	Value	95% Confidence Interval	
		Lower	Upper
Odds Ratio for Nursing_home (0 / 1)	3,167	,574	17,478
For cohort Short_treatment = ,00	1,956	,595	6,430
For cohort Short_treatment = 1,00	,618	,360	1,059
N of Valid Cases	75		

Previous_ESBL_UTI * Short_treatment

Crosstab

			Short_treatment		Total
			,00	1,00	
Previous_ESBL_UTI	0	Count	34	27	61
		% within Previous_ESBL_UTI	55,7%	44,3%	100,0%
		% within Short_treatment	87,2%	77,1%	82,4%
		% of Total	45,9%	36,5%	82,4%
	1	Count	5	8	13
		% within Previous_ESBL_UTI	38,5%	61,5%	100,0%
		% within Short_treatment	12,8%	22,9%	17,6%
		% of Total	6,8%	10,8%	17,6%
Total		Count	39	35	74
		% within Previous_ESBL_UTI	52,7%	47,3%	100,0%
		% within Short_treatment	100,0%	100,0%	100,0%
		% of Total	52,7%	47,3%	100,0%

Chi-Square Tests

	Value	df	Asymp. Sig. (2-sided)	Exact Sig. (2-sided)	Exact Sig. (1-sided)
Pearson Chi-Square	1,283[a]	1	,257		
Continuity Correction[b]	,684	1	,408		
Likelihood Ratio	1,287	1	,257		
Fisher's Exact Test				,361	,204
Linear-by-Linear Association	1,266	1	,261		
N of Valid Cases	74				

a. 0 cells (0,0%) have expected count less than 5. The minimum expected count is 6,15.

b. Computed only for a 2x2 table

Risk Estimate

	Value	95% Confidence Interval	
		Lower	Upper
Odds Ratio for Previous_ESBL_UTI (0 / 1)	2,015	,591	6,867
For cohort Short_treatment = ,00	1,449	,703	2,986
For cohort Short_treatment = 1,00	,719	,430	1,202
N of Valid Cases	74		

Carbapenem * Short_treatment

Crosstab

			Short_treatment ,00	Short_treatment 1,00	Total
Carbapenem	,00	Count	19	20	39
		% within Carbapenem	48,7%	51,3%	100,0%
		% within Short_treatment	47,5%	57,1%	52,0%
		% of Total	25,3%	26,7%	52,0%
	1,00	Count	21	15	36
		% within Carbapenem	58,3%	41,7%	100,0%
		% within Short_treatment	52,5%	42,9%	48,0%
		% of Total	28,0%	20,0%	48,0%
Total		Count	40	35	75
		% within Carbapenem	53,3%	46,7%	100,0%
		% within Short_treatment	100,0%	100,0%	100,0%
		% of Total	53,3%	46,7%	100,0%

Chi-Square Tests

	Value	df	Asymp. Sig. (2-sided)	Exact Sig. (2-sided)	Exact Sig. (1-sided)
Pearson Chi-Square	,695[a]	1	,404		
Continuity Correction[b]	,363	1	,547		
Likelihood Ratio	,697	1	,404		
Fisher's Exact Test				,489	,274
Linear-by-Linear Association	,686	1	,407		
N of Valid Cases	75				

a. 0 cells (0,0%) have expected count less than 5. The minimum expected count is 16,80.

b. Computed only for a 2x2 table

Risk Estimate

	Value	95% Confidence Interval Lower	95% Confidence Interval Upper
Odds Ratio for Carbapenem (,00 / 1,00)	,679	,272	1,691
For cohort Short_treatment = ,00	,835	,546	1,276
For cohort Short_treatment = 1,00	1,231	,752	2,015
N of Valid Cases	75		

Betalactam_betalactamase_inhibitor * Short_treatment

Crosstab

			Short_treatment		Total
			,00	1,00	
Betalactam_betalactamase_inhibitor	,00	Count	36	34	70
		% within Betalactam_betalactamase_inhibitor	51,4%	48,6%	100,0%
		% within Short_treatment	90,0%	97,1%	93,3%
		% of Total	48,0%	45,3%	93,3%
	1,00	Count	4	1	5
		% within Betalactam_betalactamase_inhibitor	80,0%	20,0%	100,0%
		% within Short_treatment	10,0%	2,9%	6,7%
		% of Total	5,3%	1,3%	6,7%
Total		Count	40	35	75
		% within Betalactam_betalactamase_inhibitor	53,3%	46,7%	100,0%
		% within Short_treatment	100,0%	100,0%	100,0%
		% of Total	53,3%	46,7%	100,0%

Chi-Square Tests

	Value	df	Asymp. Sig. (2-sided)	Exact Sig. (2-sided)	Exact Sig. (1-sided)
Pearson Chi-Square	1,531[a]	1	,216		
Continuity Correction[b]	,598	1	,439		
Likelihood Ratio	1,651	1	,199		
Fisher's Exact Test				,364	,223
Linear-by-Linear Association	1,510	1	,219		
N of Valid Cases	75				

a. 2 cells (50,0%) have expected count less than 5. The minimum expected count is 2,33.

b. Computed only for a 2x2 table

Risk Estimate

	Value	95% Confidence Interval	
		Lower	Upper
Odds Ratio for Betalactam_betalactamase_inhibitor (,00 / 1,00)	,265	,028	2,489
For cohort Short_treatment = ,00	,643	,392	1,053
For cohort Short_treatment = 1,00	2,429	,414	14,251
N of Valid Cases	75		

Quinolone * Short_treatment

Crosstab

			Short_treatment		Total
			,00	1,00	
Quinolone	,00	Count	34	29	63
		% within Quinolone	54,0%	46,0%	100,0%
		% within Short_treatment	85,0%	82,9%	84,0%
		% of Total	45,3%	38,7%	84,0%
	1,00	Count	6	6	12
		% within Quinolone	50,0%	50,0%	100,0%
		% within Short_treatment	15,0%	17,1%	16,0%
		% of Total	8,0%	8,0%	16,0%
Total		Count	40	35	75
		% within Quinolone	53,3%	46,7%	100,0%
		% within Short_treatment	100,0%	100,0%	100,0%
		% of Total	53,3%	46,7%	100,0%

Chi-Square Tests

	Value	df	Asymp. Sig. (2-sided)	Exact Sig. (2-sided)	Exact Sig. (1-sided)
Pearson Chi-Square	,064[a]	1	,801		
Continuity Correction[b]	,000	1	1,000		
Likelihood Ratio	,064	1	,801		
Fisher's Exact Test				1,000	,523
Linear-by-Linear Association	,063	1	,802		
N of Valid Cases	75				

a. 0 cells (0,0%) have expected count less than 5. The minimum expected count is 5,60.

b. Computed only for a 2x2 table

Risk Estimate

	Value	95% Confidence Interval	
		Lower	Upper
Odds Ratio for Quinolone (,00 / 1,00)	1,172	,341	4,032
For cohort Short_treatment = ,00	1,079	,586	1,987
For cohort Short_treatment = 1,00	,921	,492	1,721
N of Valid Cases	75		

Trimethoprim_sulfamethoxazole * Short_treatment

Crosstab

			Short_treatment		Total
			,00	1,00	
Trimethoprim_sulfamethoxazole	,00	Count	37	31	68
		% within Trimethoprim_sulfamethoxazole	54,4%	45,6%	100,0%
		% within Short_treatment	92,5%	88,6%	90,7%
		% of Total	49,3%	41,3%	90,7%
	1,00	Count	3	4	7
		% within Trimethoprim_sulfamethoxazole	42,9%	57,1%	100,0%
		% within Short_treatment	7,5%	11,4%	9,3%
		% of Total	4,0%	5,3%	9,3%
Total		Count	40	35	75
		% within Trimethoprim_sulfamethoxazole	53,3%	46,7%	100,0%
		% within Short_treatment	100,0%	100,0%	100,0%
		% of Total	53,3%	46,7%	100,0%

Chi-Square Tests

	Value	df	Asymp. Sig. (2-sided)	Exact Sig. (2-sided)	Exact Sig. (1-sided)
Pearson Chi-Square	,340[a]	1	,560		
Continuity Correction[b]	,034	1	,853		
Likelihood Ratio	,340	1	,560		
Fisher's Exact Test				,699	,424
Linear-by-Linear Association	,336	1	,562		
N of Valid Cases	75				

a. 2 cells (50,0%) have expected count less than 5. The minimum expected count is 3,27.

b. Computed only for a 2x2 table

Risk Estimate

	Value	95% Confidence Interval	
		Lower	Upper
Odds Ratio for Trimethoprim_sulfamethoxazole (,00 / 1,00)	1,591	,331	7,658
For cohort Short_treatment = ,00	1,270	,525	3,069
For cohort Short_treatment = 1,00	,798	,399	1,594
N of Valid Cases	75		

Phosfomycin * Short_treatment

Crosstab

			Short_treatment		Total
			,00	1,00	
Phosfomycin	,00	Count	36	26	62
		% within Phosfomycin	58,1%	41,9%	100,0%
		% within Short_treatment	90,0%	74,3%	82,7%
		% of Total	48,0%	34,7%	82,7%
	1,00	Count	4	9	13
		% within Phosfomycin	30,8%	69,2%	100,0%
		% within Short_treatment	10,0%	25,7%	17,3%
		% of Total	5,3%	12,0%	17,3%
Total		Count	40	35	75
		% within Phosfomycin	53,3%	46,7%	100,0%
		% within Short_treatment	100,0%	100,0%	100,0%
		% of Total	53,3%	46,7%	100,0%

Chi-Square Tests

	Value	df	Asymp. Sig. (2-sided)	Exact Sig. (2-sided)	Exact Sig. (1-sided)
Pearson Chi-Square	3,217[a]	1	,073		
Continuity Correction[b]	2,214	1	,137		
Likelihood Ratio	3,260	1	,071		
Fisher's Exact Test				,124	,068
Linear-by-Linear Association	3,174	1	,075		
N of Valid Cases	75				

a. 0 cells (0,0%) have expected count less than 5. The minimum expected count is 6,07.

b. Computed only for a 2x2 table

Risk Estimate

	Value	95% Confidence Interval	
		Lower	Upper
Odds Ratio for Phosfomycin (,00 / 1,00)	3,115	,865	11,219
For cohort Short_treatment = ,00	1,887	,813	4,382
For cohort Short_treatment = 1,00	,606	,380	,965
N of Valid Cases	75		

Furantoin * Short_treatment

Crosstab

			Short_treatment		Total
			,00	1,00	
Furantoin	,00	Count	40	33	73
		% within Furantoin	54,8%	45,2%	100,0%
		% within Short_treatment	100,0%	94,3%	97,3%
		% of Total	53,3%	44,0%	97,3%
	1,00	Count	0	2	2
		% within Furantoin	0,0%	100,0%	100,0%
		% within Short_treatment	0,0%	5,7%	2,7%
		% of Total	0,0%	2,7%	2,7%
Total		Count	40	35	75
		% within Furantoin	53,3%	46,7%	100,0%
		% within Short_treatment	100,0%	100,0%	100,0%
		% of Total	53,3%	46,7%	100,0%

Chi-Square Tests

	Value	df	Asymp. Sig. (2-sided)	Exact Sig. (2-sided)	Exact Sig. (1-sided)
Pearson Chi-Square	2,348[a]	1	,125		
Continuity Correction[b]	,663	1	,416		
Likelihood Ratio	3,111	1	,078		
Fisher's Exact Test				,214	,214
Linear-by-Linear Association	2,317	1	,128		
N of Valid Cases	75				

a. 2 cells (50,0%) have expected count less than 5. The minimum expected count is ,93.

b. Computed only for a 2x2 table

Risk Estimate

	Value	95% Confidence Interval	
		Lower	Upper
For cohort Short_treatment = 1,00	,452	,351	,582
N of Valid Cases	75		

Aminoglicosyde * Short_treatment

Crosstab

			Short_treatment		Total
			,00	1,00	
Aminoglicosyde	,00	Count	39	35	74
		% within Aminoglicosyde	52,7%	47,3%	100,0%
		% within Short_treatment	97,5%	100,0%	98,7%
		% of Total	52,0%	46,7%	98,7%
	1,00	Count	1	0	1
		% within Aminoglicosyde	100,0%	0,0%	100,0%
		% within Short_treatment	2,5%	0,0%	1,3%
		% of Total	1,3%	0,0%	1,3%
Total		Count	40	35	75
		% within Aminoglicosyde	53,3%	46,7%	100,0%
		% within Short_treatment	100,0%	100,0%	100,0%
		% of Total	53,3%	46,7%	100,0%

Chi-Square Tests

	Value	df	Asymp. Sig. (2-sided)	Exact Sig. (2-sided)	Exact Sig. (1-sided)
Pearson Chi-Square	,887[a]	1	,346		
Continuity Correction[b]	,000	1	1,000		
Likelihood Ratio	1,269	1	,260		
Fisher's Exact Test				1,000	,533
Linear-by-Linear Association	,875	1	,350		
N of Valid Cases	75				

a. 2 cells (50,0%) have expected count less than 5. The minimum expected count is ,47.

b. Computed only for a 2x2 table

Risk Estimate

	Value	95% Confidence Interval	
		Lower	Upper
For cohort Short_treatment = ,00	,527	,425	,654
N of Valid Cases	75		

Cystitis * Short_treatment

Crosstab

			Short_treatment		Total
			,00	1,00	
Cystitis	,00	Count	24	15	39
		% within Cystitis	61,5%	38,5%	100,0%
		% within Short_treatment	60,0%	42,9%	52,0%
		% of Total	32,0%	20,0%	52,0%
	1,00	Count	16	20	36
		% within Cystitis	44,4%	55,6%	100,0%
		% within Short_treatment	40,0%	57,1%	48,0%
		% of Total	21,3%	26,7%	48,0%
Total		Count	40	35	75
		% within Cystitis	53,3%	46,7%	100,0%
		% within Short_treatment	100,0%	100,0%	100,0%
		% of Total	53,3%	46,7%	100,0%

Chi-Square Tests

	Value	df	Asymp. Sig. (2-sided)	Exact Sig. (2-sided)	Exact Sig. (1-sided)
Pearson Chi-Square	2,198[a]	1	,138		
Continuity Correction[b]	1,565	1	,211		
Likelihood Ratio	2,208	1	,137		
Fisher's Exact Test				,168	,105
Linear-by-Linear Association	2,168	1	,141		
N of Valid Cases	75				

a. 0 cells (0,0%) have expected count less than 5. The minimum expected count is 16,80.

b. Computed only for a 2x2 table

Risk Estimate

	Value	95% Confidence Interval	
		Lower	Upper
Odds Ratio for Cystitis (,00 / 1,00)	2,000	,796	5,024
For cohort Short_treatment = ,00	1,385	,890	2,153
For cohort Short_treatment = 1,00	,692	,423	1,133
N of Valid Cases	75		

Febrile_UTI * Short_treatment

Crosstab

			Short_treatment		Total
			,00	1,00	
Febrile_UTI	,00	Count	26	23	49
		% within Febrile_UTI	53,1%	46,9%	100,0%
		% within Short_treatment	65,0%	65,7%	65,3%
		% of Total	34,7%	30,7%	65,3%
	1,00	Count	14	12	26
		% within Febrile_UTI	53,8%	46,2%	100,0%
		% within Short_treatment	35,0%	34,3%	34,7%
		% of Total	18,7%	16,0%	34,7%
Total		Count	40	35	75
		% within Febrile_UTI	53,3%	46,7%	100,0%
		% within Short_treatment	100,0%	100,0%	100,0%
		% of Total	53,3%	46,7%	100,0%

Chi-Square Tests

	Value	df	Asymp. Sig. (2-sided)	Exact Sig. (2-sided)	Exact Sig. (1-sided)
Pearson Chi-Square	,004[a]	1	,948		
Continuity Correction[b]	,000	1	1,000		
Likelihood Ratio	,004	1	,948		
Fisher's Exact Test				1,000	,571
Linear-by-Linear Association	,004	1	,949		
N of Valid Cases	75				

a. 0 cells (0,0%) have expected count less than 5. The minimum expected count is 12,13.

b. Computed only for a 2x2 table

Risk Estimate

	Value	95% Confidence Interval	
		Lower	Upper
Odds Ratio for Febrile_UTI (,00 / 1,00)	,969	,373	2,514
For cohort Short_treatment = ,00	,985	,633	1,534
For cohort Short_treatment = 1,00	1,017	,610	1,695
N of Valid Cases	75		

Pyelonephritis * Short_treatment

Crosstab

			Short_treatment		Total
			,00	1,00	
Pyelonephritis	,00	Count	30	32	62
		% within Pyelonephritis	48,4%	51,6%	100,0%
		% within Short_treatment	75,0%	91,4%	82,7%
		% of Total	40,0%	42,7%	82,7%
	1,00	Count	10	3	13
		% within Pyelonephritis	76,9%	23,1%	100,0%
		% within Short_treatment	25,0%	8,6%	17,3%
		% of Total	13,3%	4,0%	17,3%
Total		Count	40	35	75
		% within Pyelonephritis	53,3%	46,7%	100,0%
		% within Short_treatment	100,0%	100,0%	100,0%
		% of Total	53,3%	46,7%	100,0%

Chi-Square Tests

	Value	df	Asymp. Sig. (2-sided)	Exact Sig. (2-sided)	Exact Sig. (1-sided)
Pearson Chi-Square	3,516[a]	1	,061		
Continuity Correction[b]	2,463	1	,117		
Likelihood Ratio	3,707	1	,054		
Fisher's Exact Test				,073	,056
Linear-by-Linear Association	3,469	1	,063		
N of Valid Cases	75				

a. 0 cells (0,0%) have expected count less than 5. The minimum expected count is 6,07.

b. Computed only for a 2x2 table

Risk Estimate

	Value	95% Confidence Interval	
		Lower	Upper
Odds Ratio for Pyelonephritis (,00 / 1,00)	,281	,071	1,121
For cohort Short_treatment = ,00	,629	,424	,932
For cohort Short_treatment = 1,00	2,237	,805	6,211
N of Valid Cases	75		

Nonparametric Tests

Hypothesis Test Summary

	Null Hypothesis	Test	Sig.	Decision
1	The distribution of Age is normal with mean 74,307 and standard deviation 16,10.	One-Sample Kolmogorov-Smirnov Test	,040	Reject the null hypothesis.
2	The distribution of Barthel is normal with mean 50,424 and standard deviation 41,11.	One-Sample Kolmogorov-Smirnov Test	,012	Reject the null hypothesis.
3	The distribution of Charlson_index is normal with mean 2,693 and standard deviation 2,02.	One-Sample Kolmogorov-Smirnov Test	,056	Retain the null hypothesis.

Asymptotic significances are displayed. The significance level is ,05.

Nonparametric Tests

Hypothesis Test Summary

	Null Hypothesis	Test	Sig.	Decision
1	The distribution of Age is the same across categories of Short_treatment.	Independent-Samples Mann-Whitney U Test	,949	Retain the null hypothesis.
2	The distribution of Barthel is the same across categories of Short_treatment.	Independent-Samples Mann-Whitney U Test	,512	Retain the null hypothesis.

Asymptotic significances are displayed. The significance level is ,05.

T-Test

Group Statistics

	Short_treatment	N	Mean	Std. Deviation	Std. Error Mean
Charlson_index	,00	40	2,63	1,996	,316
	1,00	35	2,77	2,073	,350

Independent Samples Test

		Levene's Test for Equality of Variances		t-test for Equality of Means		
		F	Sig.	t	df	Sig. (2-tailed)
Charlson_index	Equal variances assumed	,120	,730	-,311	73	,756
	Equal variances not assumed			-,310	70,870	,757

Independent Samples Test

		t-test for Equality of Means			
		Mean Difference	Std. Error Difference	95% Confidence Interval of the Difference	
				Lower	Upper
Charlson_index	Equal variances assumed	-,146	,470	-1,084	,791
	Equal variances not assumed	-,146	,472	-1,087	,794

Logistic Regression

Case Processing Summary

Unweighted Cases[a]		N	Percent
Selected Cases	Included in Analysis	75	100,0
	Missing Cases	0	,0
	Total	75	100,0
Unselected Cases		0	,0
Total		75	100,0

a. If weight is in effect, see classification table for the total number of cases.

Dependent Variable Encoding

Original Value	Internal Value
,00	0
1,00	1

Categorical Variables Codings

		Frequency	Parameter coding
			(1)
Hypertension	0	31	,000
	1	44	1,000
Sex	,0	32	,000
	1,0	43	1,000

Block 0: Beginning Block

Classification Table[a,b]

Observed			Predicted: Short_treatment ,00	Predicted: Short_treatment 1,00	Predicted: Percentage Correct
Step 0	Short_treatment	,00	40	0	100,0
		1,00	35	0	,0
	Overall Percentage				53,3

a. Constant is included in the model.

b. The cut value is ,500

Variables in the Equation

		B	S.E.	Wald	df	Sig.	Exp(B)
Step 0	Constant	-,134	,231	,333	1	,564	,875

Variables not in the Equation

			Score	df	Sig.
Step 0	Variables	Sex(1)	5,330	1	,021
		Hypertension(1)	4,408	1	,036
	Overall Statistics		8,461	2	,015

Block 1: Method = Forward Stepwise (Wald)

Omnibus Tests of Model Coefficients

		Chi-square	df	Sig.
Step 1	Step	5,423	1	,020
	Block	5,423	1	,020
	Model	5,423	1	,020

Model Summary

Step	-2 Log likelihood	Cox & Snell R Square	Nagelkerke R Square
1	98,216[a]	,070	,093

a. Estimation terminated at iteration number 3 because parameter estimates changed by less than ,001.

Classification Table[a]

Observed			Predicted: Short_treatment ,00	Predicted: Short_treatment 1,00	Predicted: Percentage Correct
Step 1	Short_treatment	,00	22	18	55,0
		1,00	10	25	71,4
	Overall Percentage				62,7

a. The cut value is ,500

Variables in the Equation

		B	S.E.	Wald	df	Sig.	Exp(B)	95% C.I.for EXP(B) Lower	95% C.I.for EXP(B) Upper
Step 1[a]	Sex(1)	1,117	,491	5,177	1	,023	3,056	1,167	7,998
	Constant	-,788	,381	4,274	1	,039	,455		

a. Variable(s) entered on step 1: Sex.

Variables not in the Equation

			Score	df	Sig.
Step 1	Variables	Hypertension(1)	3,380	1	,066
	Overall Statistics		3,380	1	,066
