# Supplementary material for: Treatment duration of complicated urinary tract infections by extended-spectrum beta-lactamases producing enterobacterales
Source: PLoS One. 2020 Oct 19;15(10):e0237365. doi: 10.1371/journal.pone.0237365 (PMC7571686; doi:10.1371/journal.pone.0237365)

Kaplan-Meier

Warnings

No statistics are computed because all cases are censored.

Case Processing Summary

Nephrostomy	Total N	N of Events	Censored	
			N	Percent
,00	73	4	69	94,5%
1,00	2	0	2	100,0%
Overall	75	4	71	94,7%

Overall Comparisons

	Chi-Square	df	Sig.
Log Rank (Mantel-Cox)	,112	1	,738
Breslow (Generalized Wilcoxon)	,112	1	,738

Test of equality of survival distributions for the different levels of Nephrostomy.

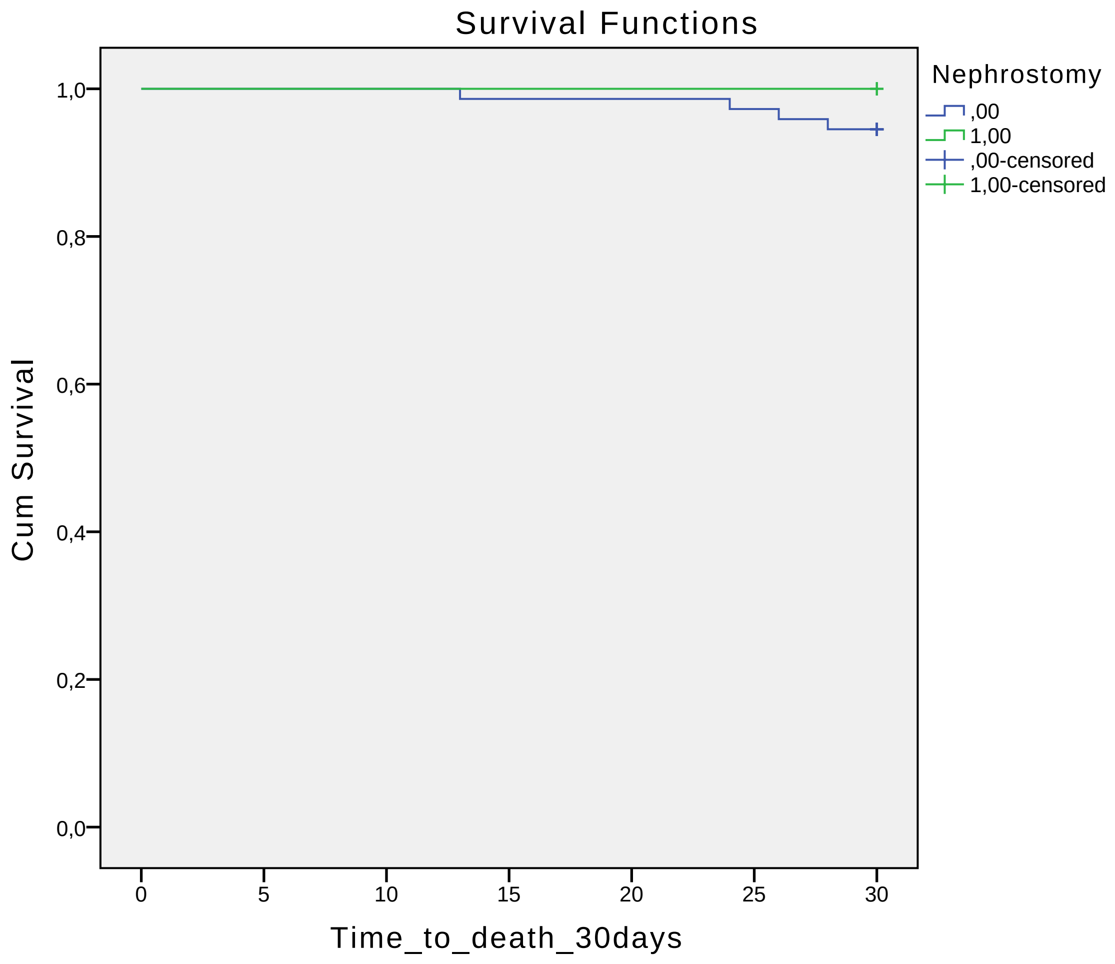

Kaplan-Meier

Warnings

No statistics are computed because all cases are censored.

Case Processing Summary

Urolithiasis	Total N	N of Events	Censored	
			N	Percent
,00	62	4	58	93,5%
1,00	13	0	13	100,0%
Overall	75	4	71	94,7%

Overall Comparisons

	Chi-Square	df	Sig.
Log Rank (Mantel-Cox)	,860	1	,354
Breslow (Generalized Wilcoxon)	,860	1	,354

Test of equality of survival distributions for the different levels of Urolithiasis.

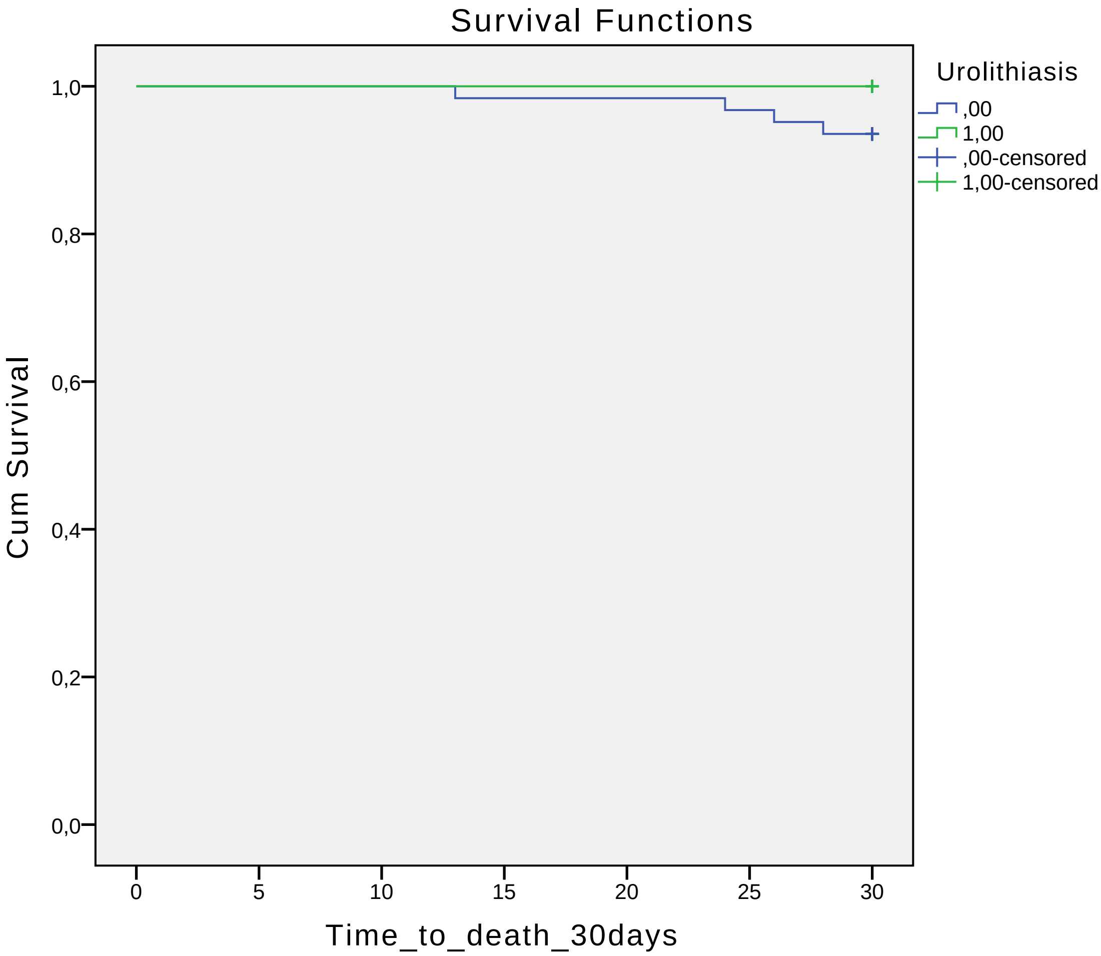

Kaplan-Meier

Warnings

No statistics are computed because all cases are censored.

Case Processing Summary

E_COLLI	Total N	N of Events	Censored	
			N	Percent
,0	14	0	14	100,0%
1,0	61	4	57	93,4%
Overall	75	4	71	94,7%

Overall Comparisons

	Chi-Square	df	Sig.
Log Rank (Mantel-Cox)	,941	1	,332
Breslow (Generalized Wilcoxon)	,941	1	,332

Test of equality of survival distributions for the different levels of E_COLLI.

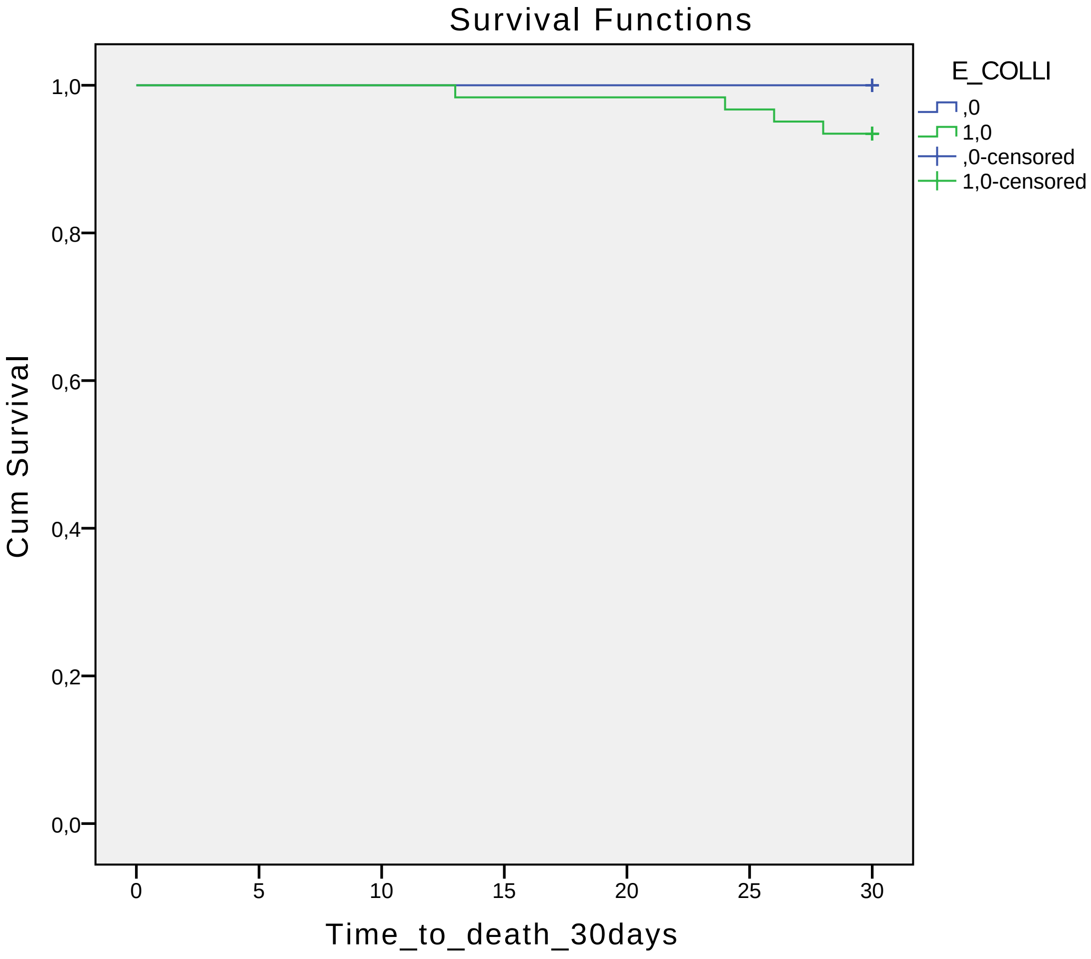

Kaplan-Meier

Warnings

No statistics are computed because all cases are censored.

Case Processing Summary

KLEBSIELLA	Total N	N of Events	Censored	
			N	Percent
,0	64	4	60	93,8%
1,0	11	0	11	100,0%
Overall	75	4	71	94,7%

Overall Comparisons

	Chi-Square	df	Sig.
Log Rank (Mantel-Cox)	,704	1	,401
Breslow (Generalized Wilcoxon)	,704	1	,401

Test of equality of survival distributions for the different levels of KLEBSIELLA.

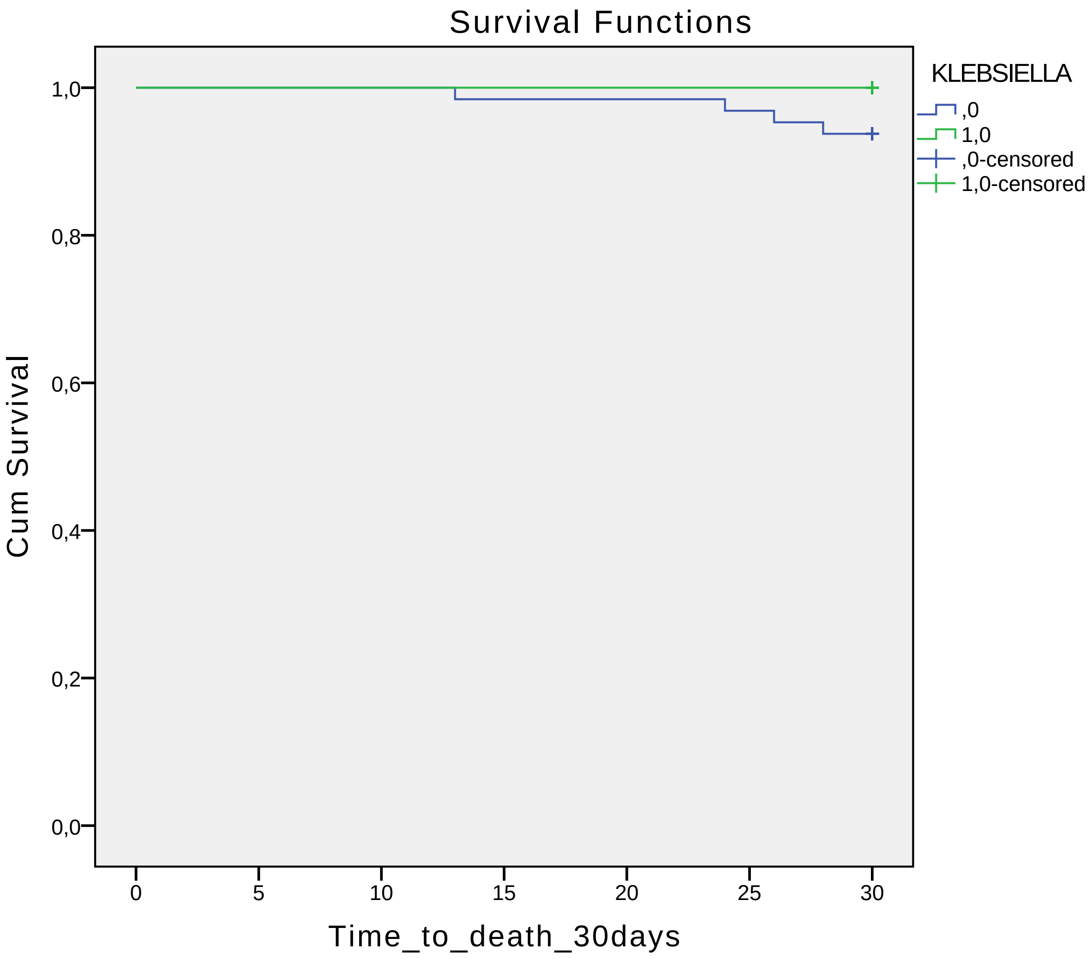

Kaplan-Meier

Warnings

No statistics are computed because all cases are censored.

Case Processing Summary

PROTEUS	Total N	N of Events	Censored	
			N	Percent
,0	73	4	69	94,5%
1,0	2	0	2	100,0%
Overall	75	4	71	94,7%

Overall Comparisons

	Chi-Square	df	Sig.
Log Rank (Mantel-Cox)	,112	1	,738
Breslow (Generalized Wilcoxon)	,112	1	,738

Test of equality of survival distributions for the different levels of PROTEUS.

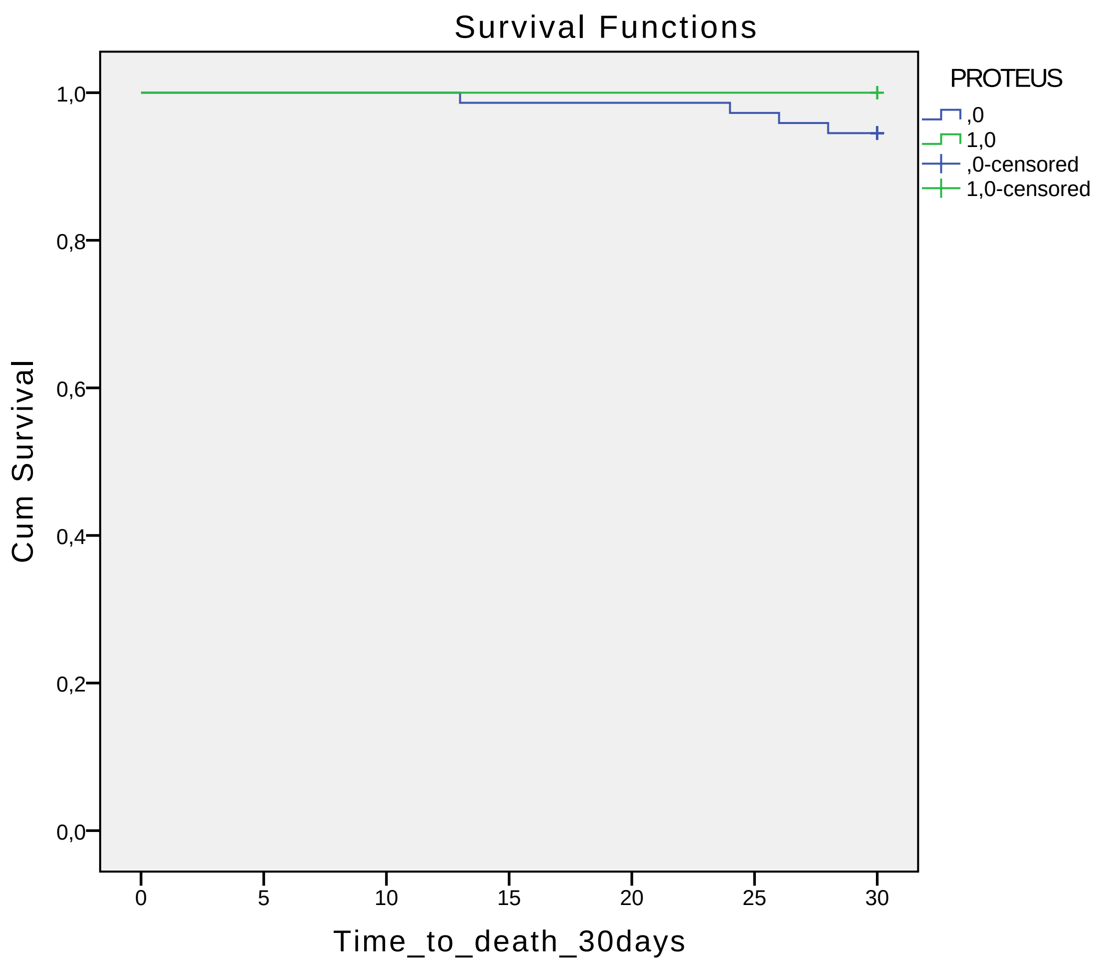

Kaplan-Meier

Warnings

No statistics are computed because all cases are censored.

Case Processing Summary

ENTEROBACTER	Total N	N of Events	Censored	
			N	Percent
,0	74	4	70	94,6%
1,0	1	0	1	100,0%
Overall	75	4	71	94,7%

Overall Comparisons

	Chi-Square	df	Sig.
Log Rank (Mantel-Cox)	,055	1	,814
Breslow (Generalized Wilcoxon)	,055	1	,814

Test of equality of survival distributions for the different levels of ENTEROBACTER.

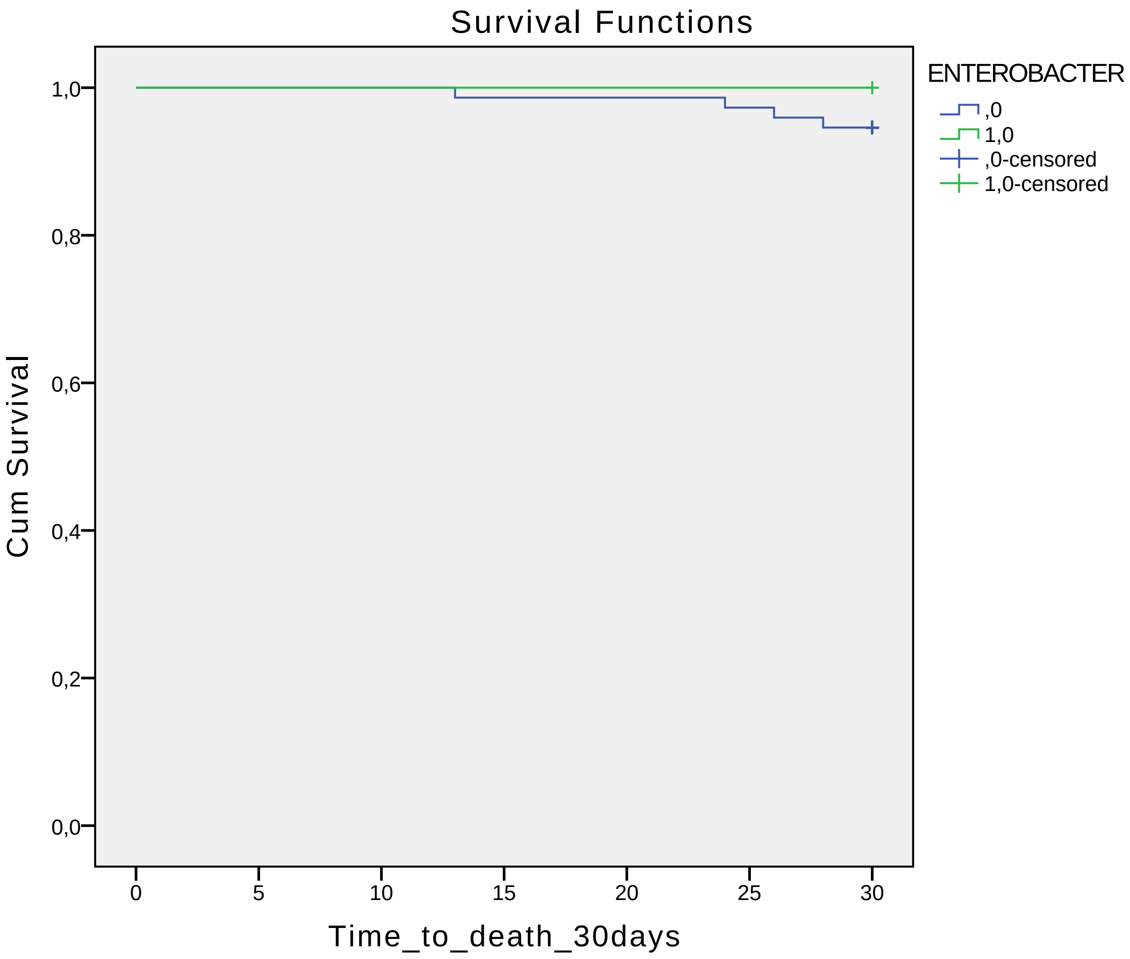

Kaplan-Meier

Warnings

No comparison analysis is performed because the factor variable has only one value for every stratum.

Case Processing Summary

Citrobacter	Total N	N of Events	Censored	
			N	Percent
,0	75	4	71	94,7%
Overall	75	4	71	94,7%

Means and Medians for Survival Time

Citrobacter	Mean[a]				Median			
	Estimate	Std. Error	95% Confidence Interval		Estimate	Std. Error	95% Confidence Interval	
			Lower Bound	Upper Bound			Lower Bound	Upper Bound
,0	29,613	,244	29,136	30,091	.	.	.	.
Overall	29,613	,244	29,136	30,091	.	.	.	.

a. Estimation is limited to the largest survival time if it is censored.

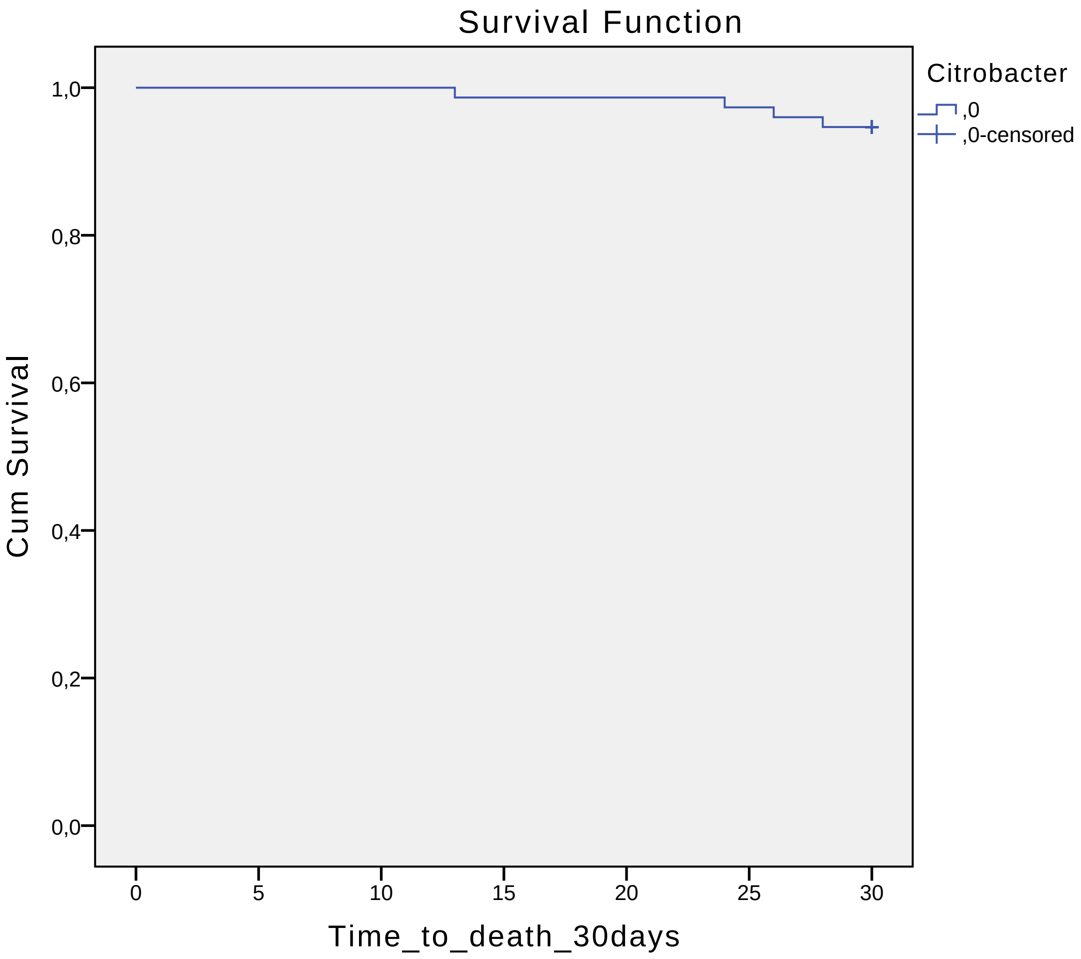

Kaplan-Meier

Warnings

No comparison analysis is performed because the factor variable has only one value for every stratum.

Case Processing Summary

Morganella	Total N	N of Events	Censored	
			N	Percent
,0	75	4	71	94,7%
Overall	75	4	71	94,7%

Means and Medians for Survival Time

Morganella	Mean[a]				Median			
			95% Confidence Interval				95% Confidence Interval	
	Estimate	Std. Error	Lower Bound	Upper Bound	Estimate	Std. Error	Lower Bound	Upper Bound
,0	29,613	,244	29,136	30,091	.	.	.	.
Overall	29,613	,244	29,136	30,091	.	.	.	.

a. Estimation is limited to the largest survival time if it is censored.

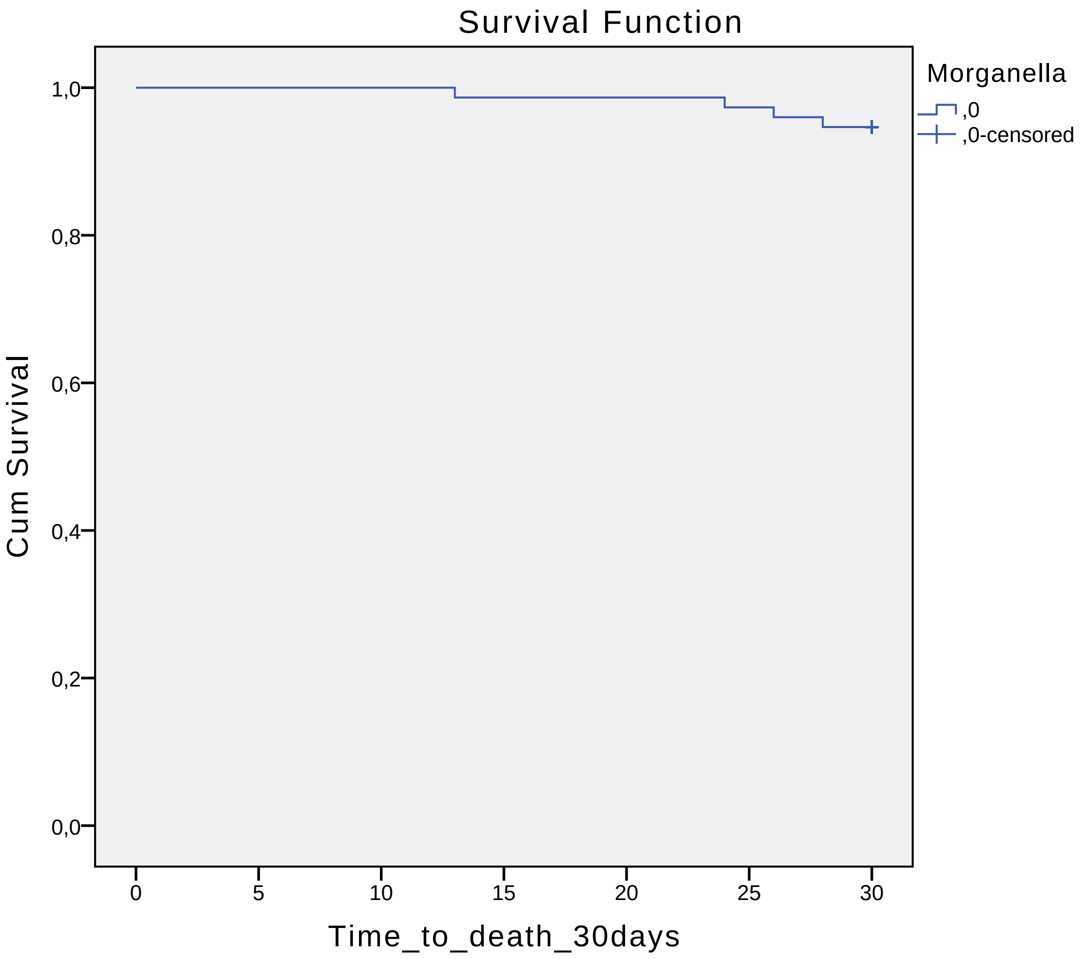

Kaplan-Meier

Warnings

No statistics are computed because all cases are censored.

Case Processing Summary

SEPSIS_SHOCK	Total N	N of Events	Censored	
			N	Percent
,00	67	4	63	94,0%
1,00	8	0	8	100,0%
Overall	75	4	71	94,7%

Overall Comparisons

	Chi-Square	df	Sig.
Log Rank (Mantel-Cox)	,489	1	,485
Breslow (Generalized Wilcoxon)	,489	1	,485

Test of equality of survival distributions for the different levels of SEPSIS_SHOCK.

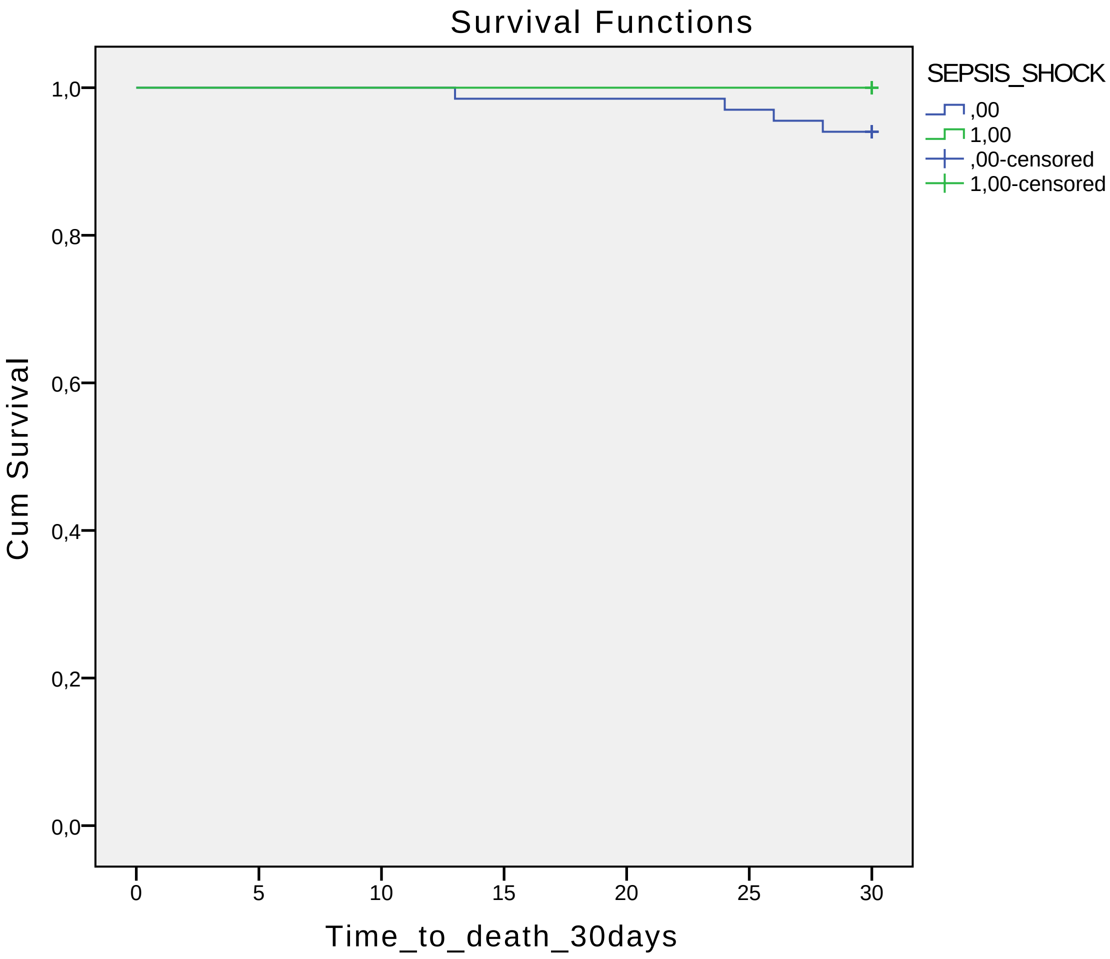

Kaplan-Meier

Warnings

No statistics are computed because all cases are censored.

Case Processing Summary

Sepsis	Total N	N of Events	Censored	
			N	Percent
,0	68	4	64	94,1%
1,0	7	0	7	100,0%
Overall	75	4	71	94,7%

Overall Comparisons

	Chi-Square	df	Sig.
Log Rank (Mantel-Cox)	,421	1	,516
Breslow (Generalized Wilcoxon)	,421	1	,516

Test of equality of survival distributions for the different levels of Sepsis.

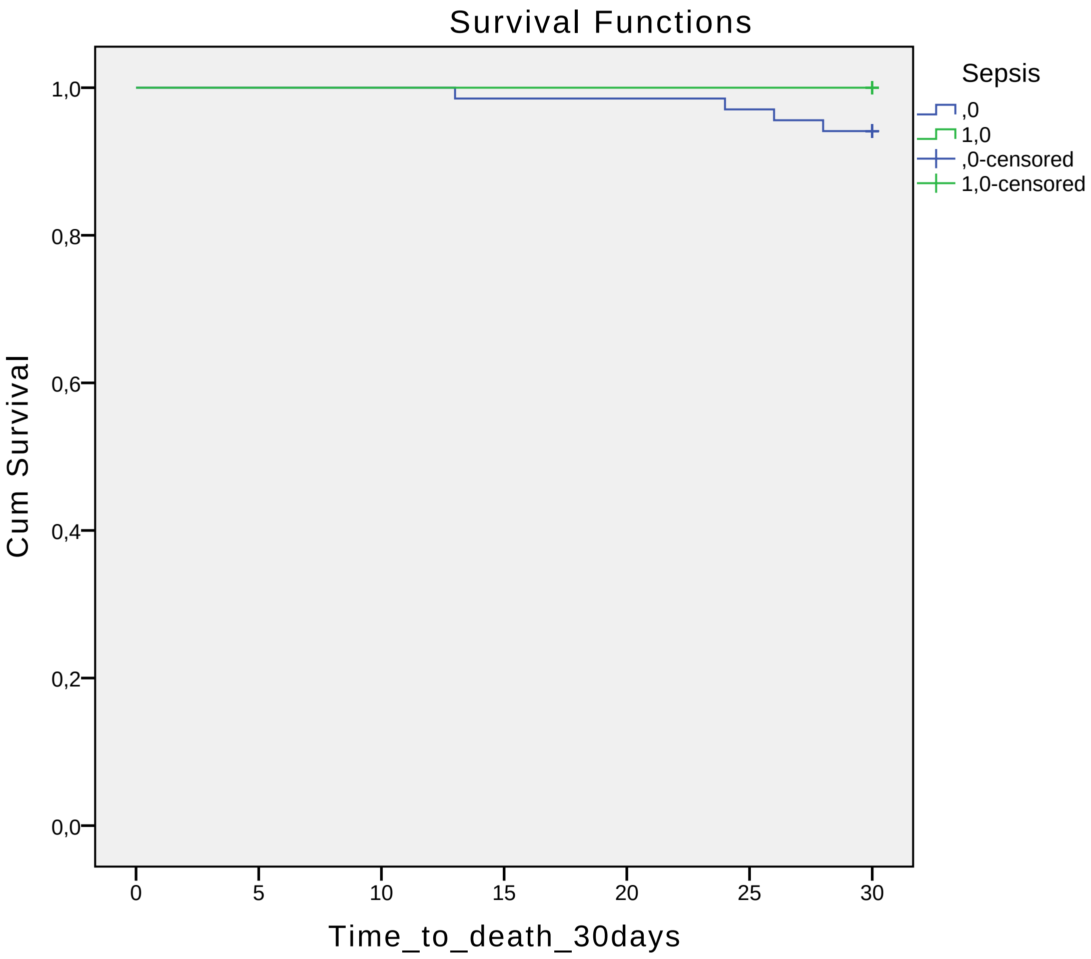

Kaplan-Meier

Warnings

No statistics are computed because all cases are censored.

Case Processing Summary

Septic_shock	Total N	N of Events	Censored	
			N	Percent
,0	74	4	70	94,6%
1,0	1	0	1	100,0%
Overall	75	4	71	94,7%

Overall Comparisons

	Chi-Square	df	Sig.
Log Rank (Mantel-Cox)	,055	1	,814
Breslow (Generalized Wilcoxon)	,055	1	,814

Test of equality of survival distributions for the different levels of Septic_shock.

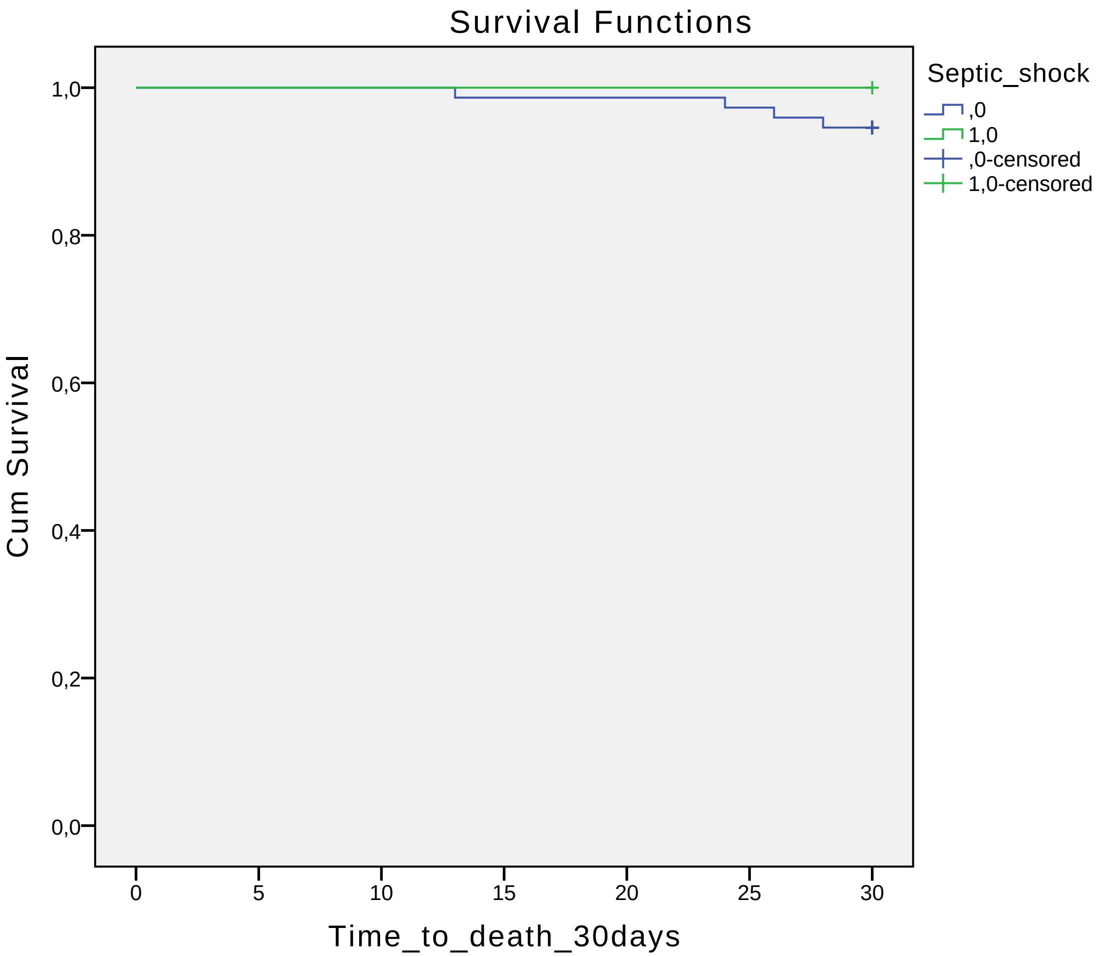

Kaplan-Meier

Warnings

No statistics are computed because all cases are censored.

Case Processing Summary

Urological_abnormality	Total N	N of Events	Censored	
			N	Percent
,0	53	4	49	92,5%
1,0	22	0	22	100,0%
Overall	75	4	71	94,7%

Overall Comparisons

	Chi-Square	df	Sig.
Log Rank (Mantel-Cox)	1,709	1	,191
Breslow (Generalized Wilcoxon)	1,709	1	,191

Test of equality of survival distributions for the different levels of Urological_abnormality.

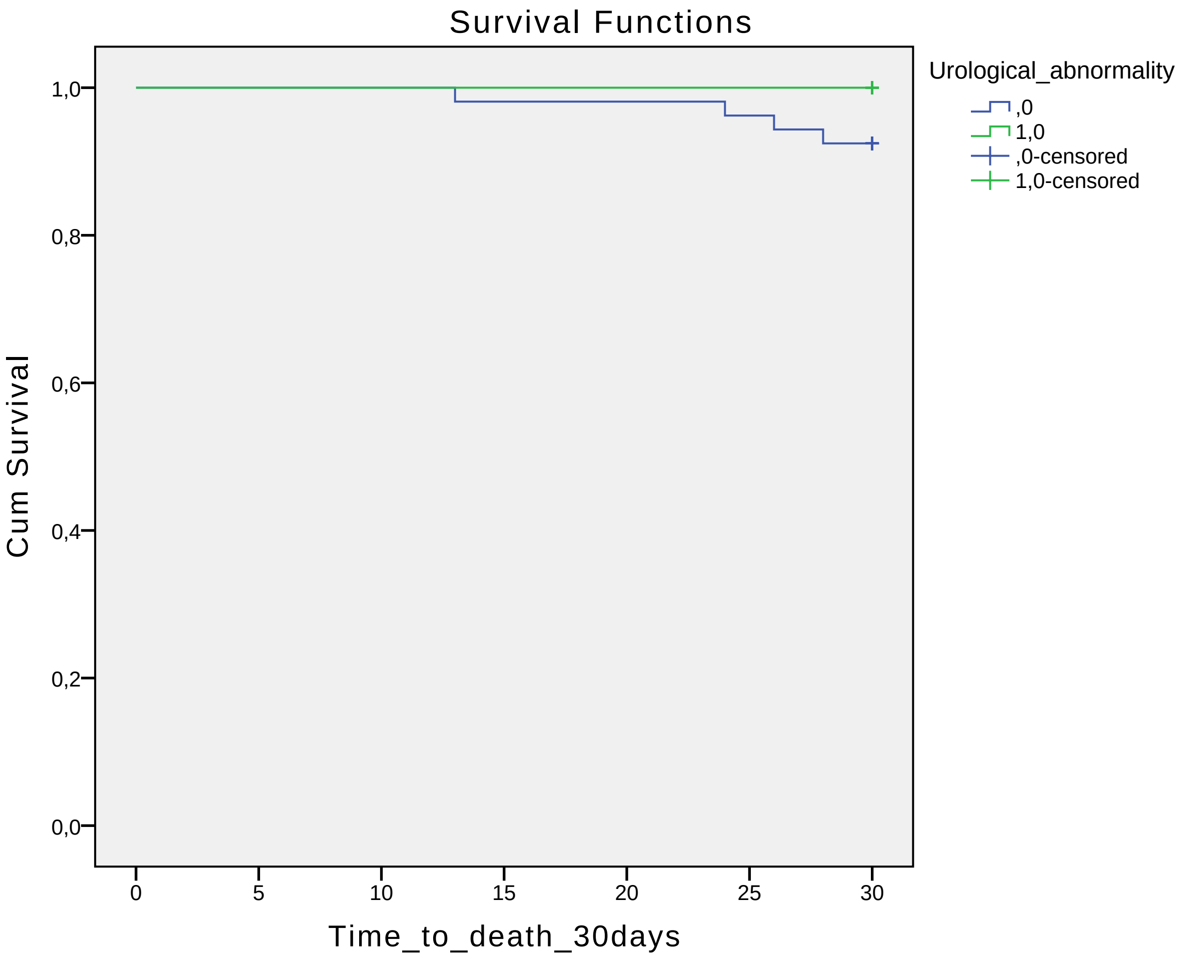

Kaplan-Meier

Case Processing Summary

Sex	Total N	N of Events	Censored	
			N	Percent
,0	32	1	31	96,9%
1,0	43	3	40	93,0%
Overall	75	4	71	94,7%

Means and Medians for Survival Time

Sex	Mean[a]				Median			
	Estimate	Std. Error	95% Confidence Interval		Estimate	Std. Error	95% Confidence Interval	
			Lower Bound	Upper Bound			Lower Bound	Upper Bound
,0	29,469	,523	28,444	30,494	.	.	.	.
1,0	29,721	,169	29,390	30,052	.	.	.	.
Overall	29,613	,244	29,136	30,091	.	.	.	.

a. Estimation is limited to the largest survival time if it is censored.

Overall Comparisons

	Chi-Square	df	Sig.
Log Rank (Mantel-Cox)	,502	1	,478
Breslow (Generalized Wilcoxon)	,473	1	,491

Test of equality of survival distributions for the different levels of Sex.

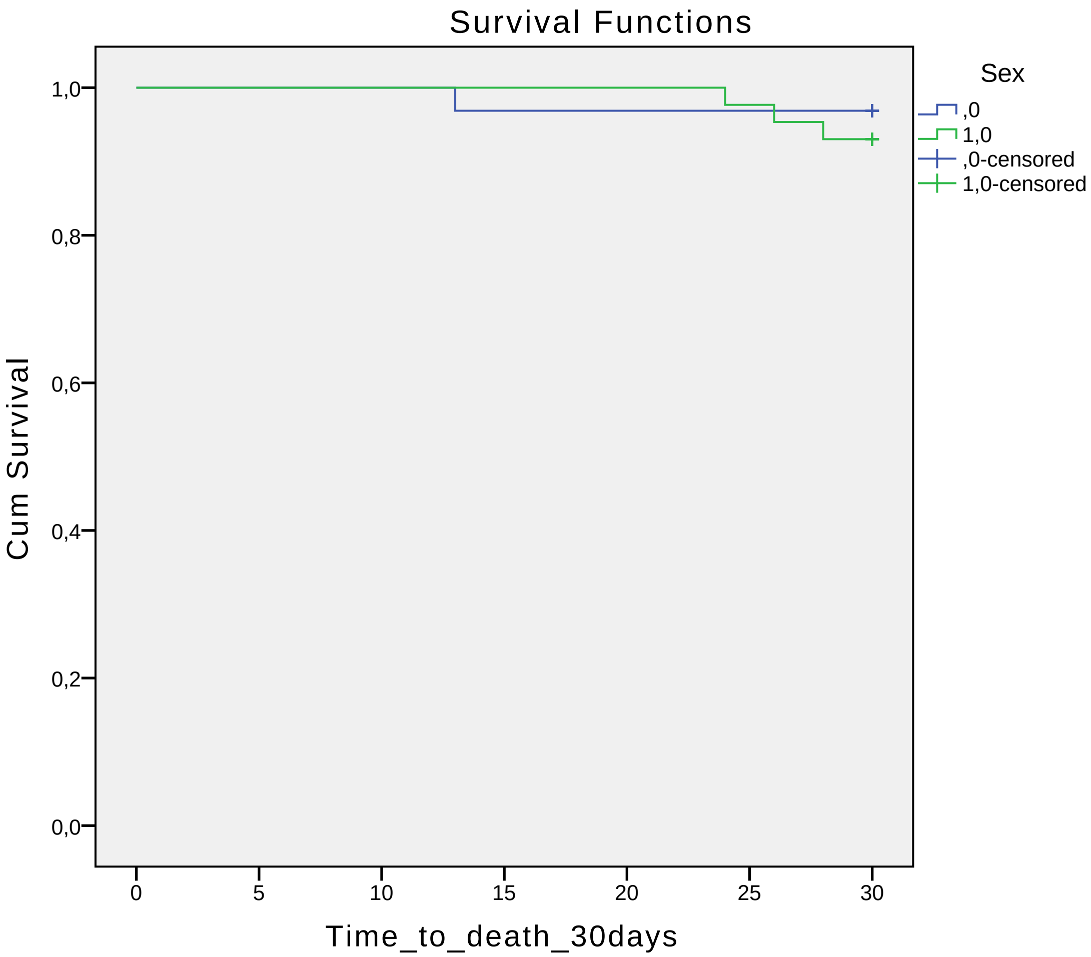

Kaplan-Meier

Case Processing Summary

Charlson_index_over_2	Total N	N of Events	Censored	
			N	Percent
,00	39	3	36	92,3%
1,00	36	1	35	97,2%
Overall	75	4	71	94,7%

Means and Medians for Survival Time

Charlson_index_over_2	Mean[a]				Median		
			95% Confidence Interval				95% ...
	Estimate	Std. Error	Lower Bound	Upper Bound	Estimate	Std. Error	Lower Bound
,00	29,359	,454	28,470	30,248	.	.	.
1,00	29,889	,110	29,674	30,104	.	.	.
Overall	29,613	,244	29,136	30,091	.	.	.

Means and Medians for Survival Time

Charlson_index_over_2	Median
	95% ...
	Upper Bound
,00	.
1,00	.
Overall	.

a. Estimation is limited to the largest survival time if it is censored.

Overall Comparisons

	Chi-Square	df	Sig.
Log Rank (Mantel-Cox)	,895	1	,344
Breslow (Generalized Wilcoxon)	,908	1	,341

Test of equality of survival distributions for the different levels of Charlson_index_over_2.

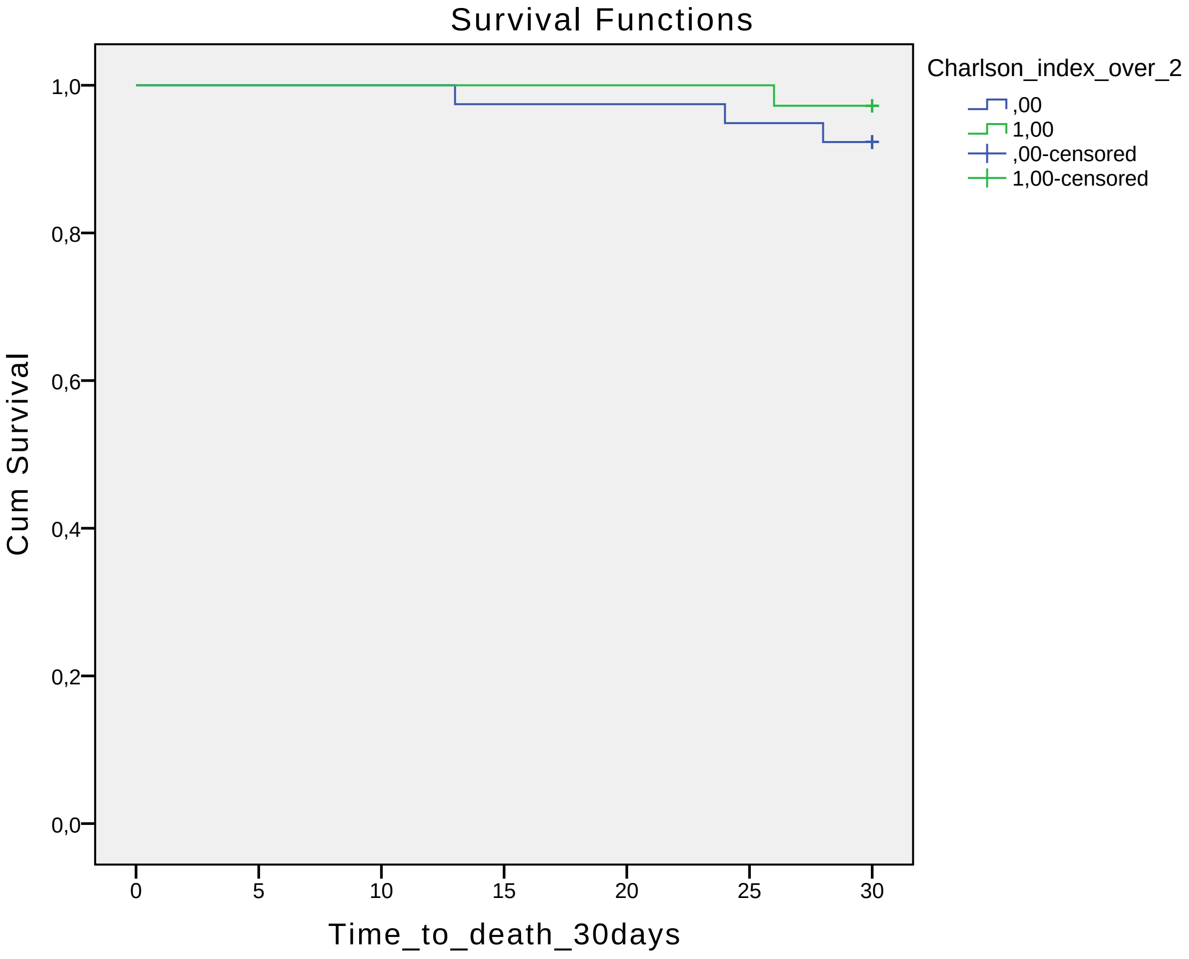

Kaplan-Meier

Case Processing Summary

Urinary_catheter	Total N	N of Events	Censored	
			N	Percent
0	59	2	57	96,6%
1	16	2	14	87,5%
Overall	75	4	71	94,7%

Means and Medians for Survival Time

Urinary_catheter	Mean[a]				Median		
			95% Confidence Interval				95% ...
	Estimate	Std. Error	Lower Bound	Upper Bound	Estimate	Std. Error	Lower Bound
0	29,898	,075	29,752	30,045	.	.	.
1	28,563	1,068	26,469	30,656	.	.	.
Overall	29,613	,244	29,136	30,091	.	.	.

Means and Medians for Survival Time

Urinary_catheter	Median
	95% ...
	Upper Bound
0	.
1	.
Overall	.

a. Estimation is limited to the largest survival time if it is censored.

Overall Comparisons

	Chi-Square	df	Sig.
Log Rank (Mantel-Cox)	2,238	1	,135
Breslow (Generalized Wilcoxon)	2,336	1	,126

Test of equality of survival distributions for the different levels of Urinary_catheter.

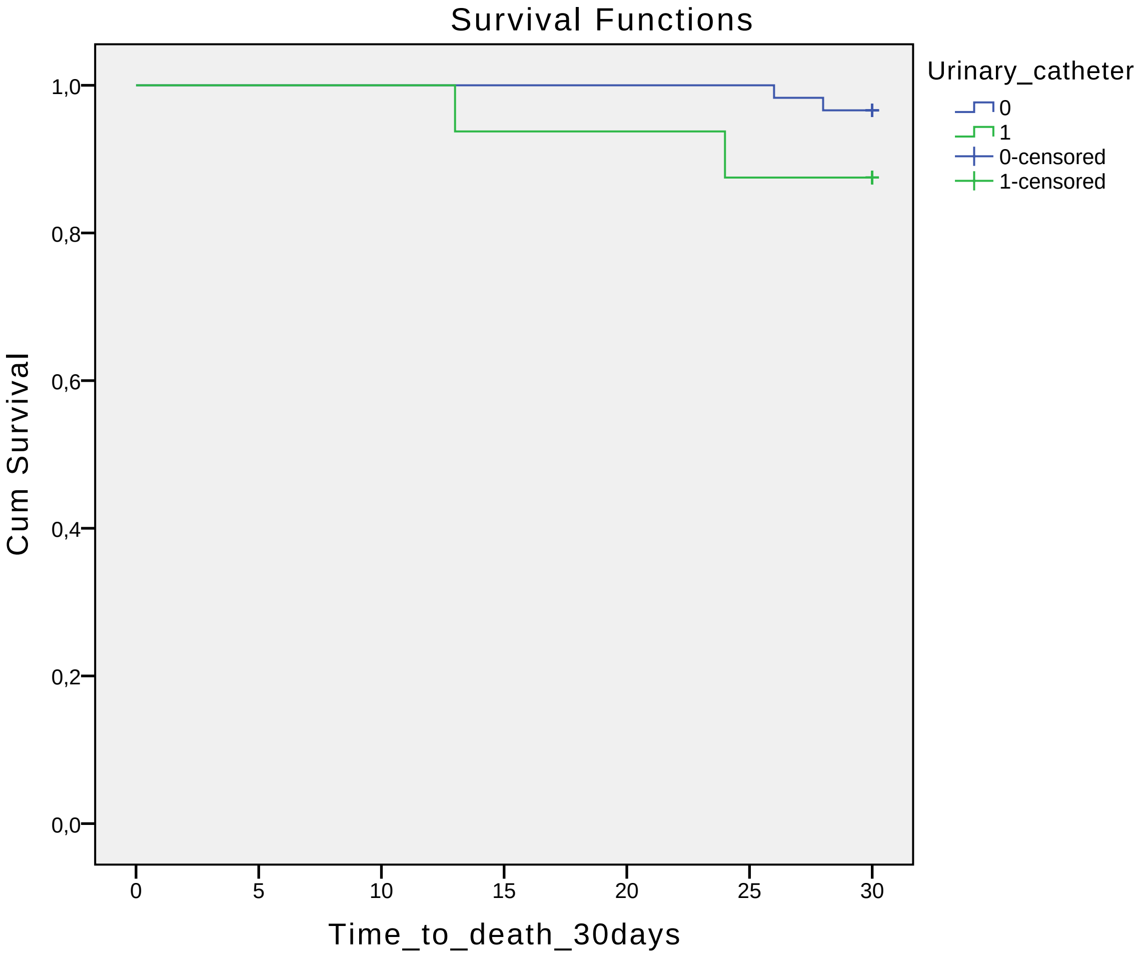

Kaplan-Meier

Case Processing Summary

Indwelling_urinary_catheter	Total N	N of Events	Censored	
			N	Percent
0	63	3	60	95,2%
1	12	1	11	91,7%
Overall	75	4	71	94,7%

Means and Medians for Survival Time

Indwelling_urinary_catheter	Mean[a]				Median		
			95% Confidence Interval				95% ...
	Estimate	Std. Error	Lower Bound	Upper Bound	Estimate	Std. Error	Lower Bound
0	29,810	,116	29,582	30,038	.	.	.
1	28,583	1,356	25,925	31,242	.	.	.
Overall	29,613	,244	29,136	30,091	.	.	.

Means and Medians for Survival Time

Indwelling_urinary_catheter	Median
	95% ...
	Upper Bound
0	.
1	.
Overall	.

a. Estimation is limited to the largest survival time if it is censored.

Overall Comparisons

	Chi-Square	df	Sig.
Log Rank (Mantel-Cox)	,290	1	,590
Breslow (Generalized Wilcoxon)	,321	1	,571

Test of equality of survival distributions for the different levels of Indwelling_urinary_catheter.

Survival Functions

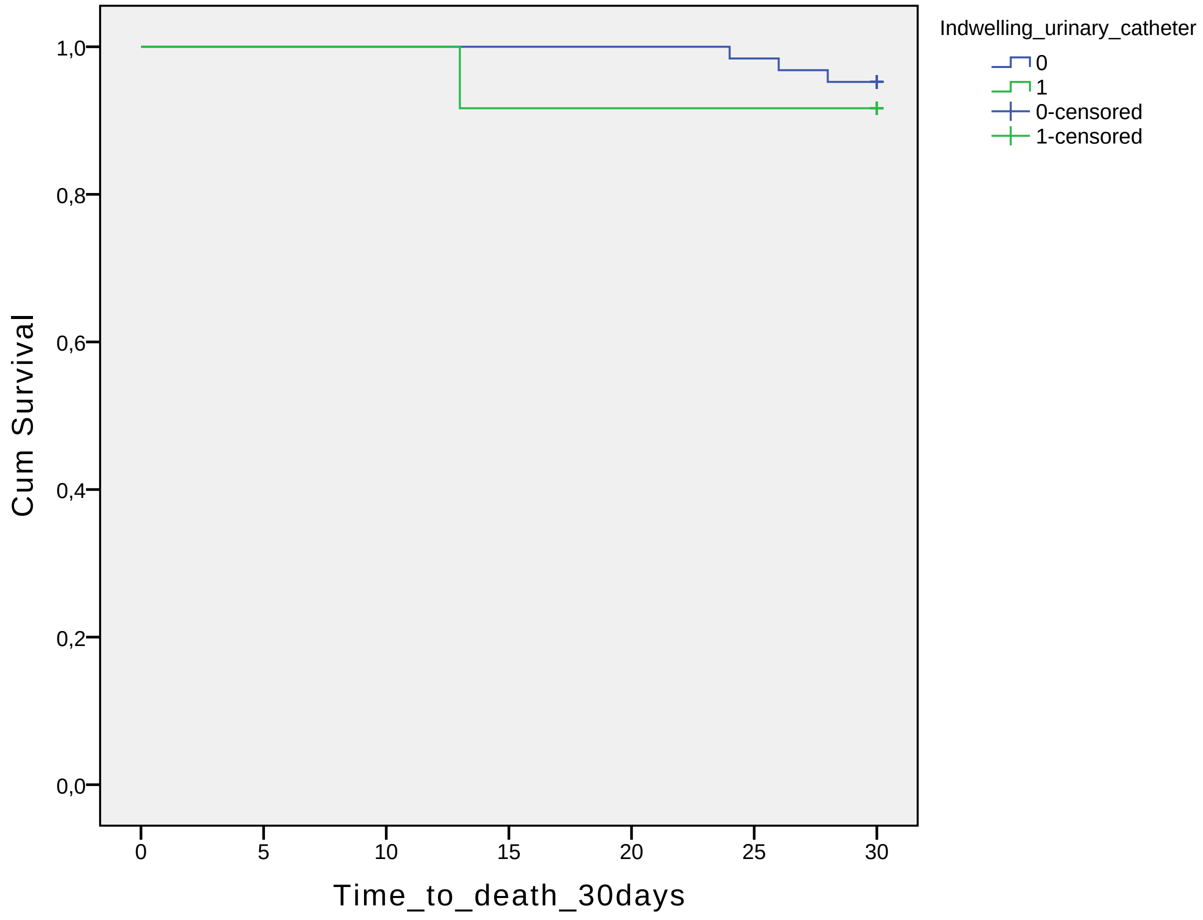

Kaplan-Meier

Case Processing Summary

Cognitive_impairment	Total N	N of Events	Censored	
			N	Percent
0	56	2	54	96,4%
1	19	2	17	89,5%
Overall	75	4	71	94,7%

Means and Medians for Survival Time

Cognitive_impairment	Mean[a]				Median		
	Estimate	Std. Error	95% Confidence Interval		Estimate	Std. Error	95% …
			Lower Bound	Upper Bound			Lower Bound
0	29,821	,127	29,573	30,069	.	.	.
1	29,000	,871	27,292	30,708	.	.	.
Overall	29,613	,244	29,136	30,091	.	.	.

Means and Medians for Survival Time

Cognitive_impairment	Median
	95% …
	Upper Bound
0	.
1	.
Overall	.

a. Estimation is limited to the largest survival time if it is censored.

Overall Comparisons

	Chi-Square	df	Sig.
Log Rank (Mantel-Cox)	1,358	1	,244
Breslow (Generalized Wilcoxon)	1,357	1	,244

Test of equality of survival distributions for the different levels of Cognitive_impairment.

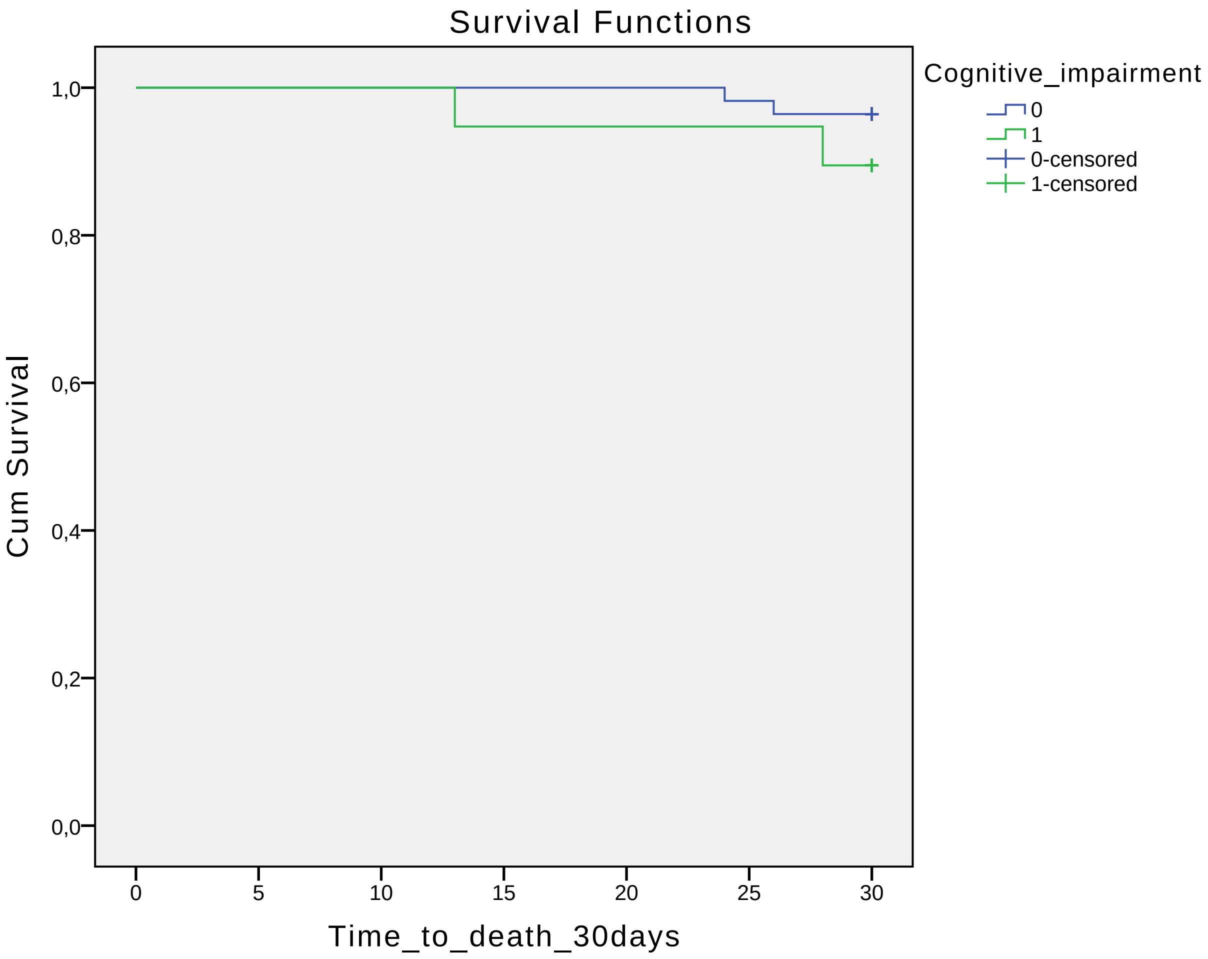

Kaplan-Meier

Warnings

No statistics are computed because all cases are censored.

Case Processing Summary

Solid_tumor	Total N	N of Events	Censored	
			N	Percent
0	65	4	61	93,8%
2	10	0	10	100,0%
Overall	75	4	71	94,7%

Overall Comparisons

	Chi-Square	df	Sig.
Log Rank (Mantel-Cox)	,630	1	,427
Breslow (Generalized Wilcoxon)	,630	1	,427

Test of equality of survival distributions for the different levels of Solid_tumor.

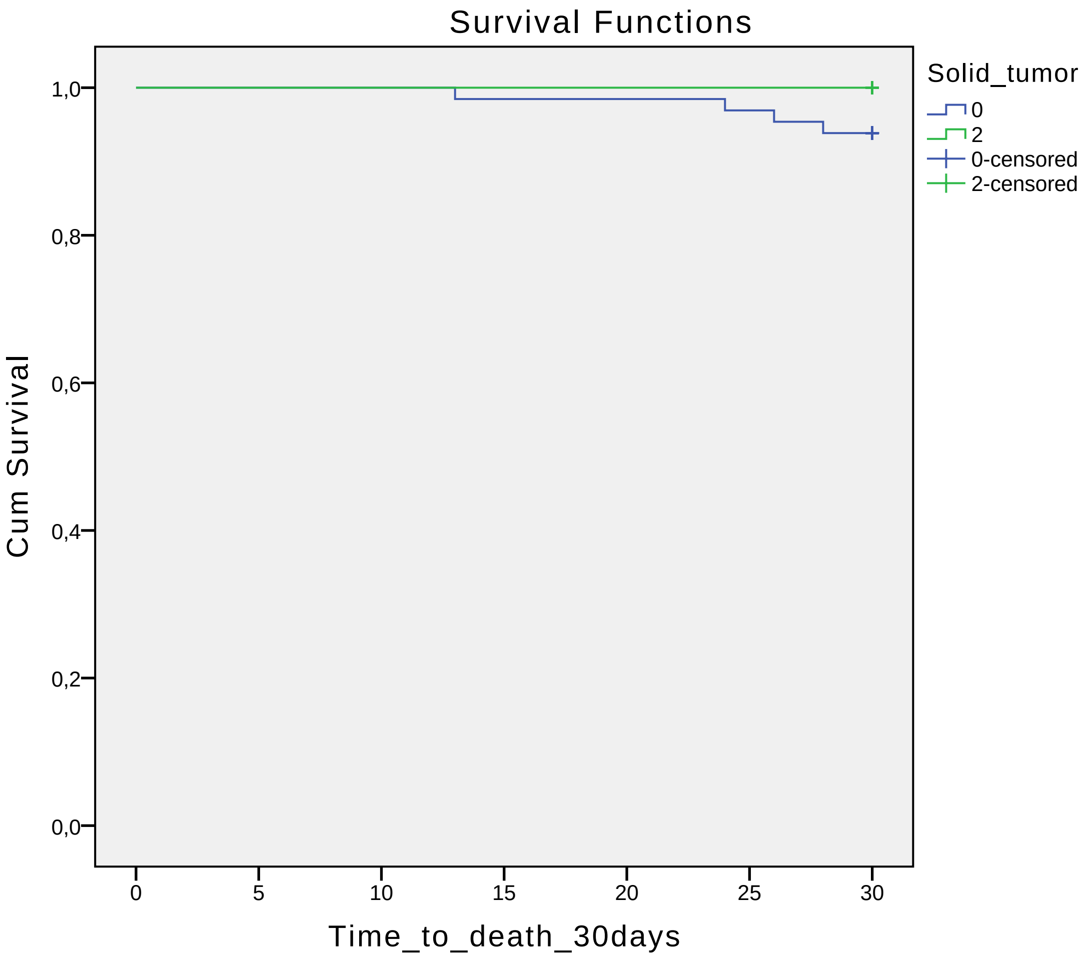

Kaplan-Meier

Case Processing Summary

Metastatic_solid_tumor	Total N	N of Events	Censored	
			N	Percent
0	73	3	70	95,9%
6	2	1	1	50,0%
Overall	75	4	71	94,7%

Means and Medians for Survival Time

Metastatic_solid_tumor	Mean[a]				Median		
	Estimate	Std. Error	95% Confidence Interval		Estimate	Std. Error	95% …
			Lower Bound	Upper Bound			Lower Bound
0	29,658	,245	29,177	30,138	.	.	.
6	28,000	1,414	25,228	30,772	26,000	.	.
Overall	29,613	,244	29,136	30,091	.	.	.

Means and Medians for Survival Time

Metastatic_solid_tumor	Median
	95% …
	Upper Bound
0	.
6	.
Overall	.

a. Estimation is limited to the largest survival time if it is censored.

Overall Comparisons

	Chi-Square	df	Sig.
Log Rank (Mantel-Cox)	8,846	1	,003
Breslow (Generalized Wilcoxon)	8,660	1	,003

Test of equality of survival distributions for the different levels of Metastatic_solid_tumor.

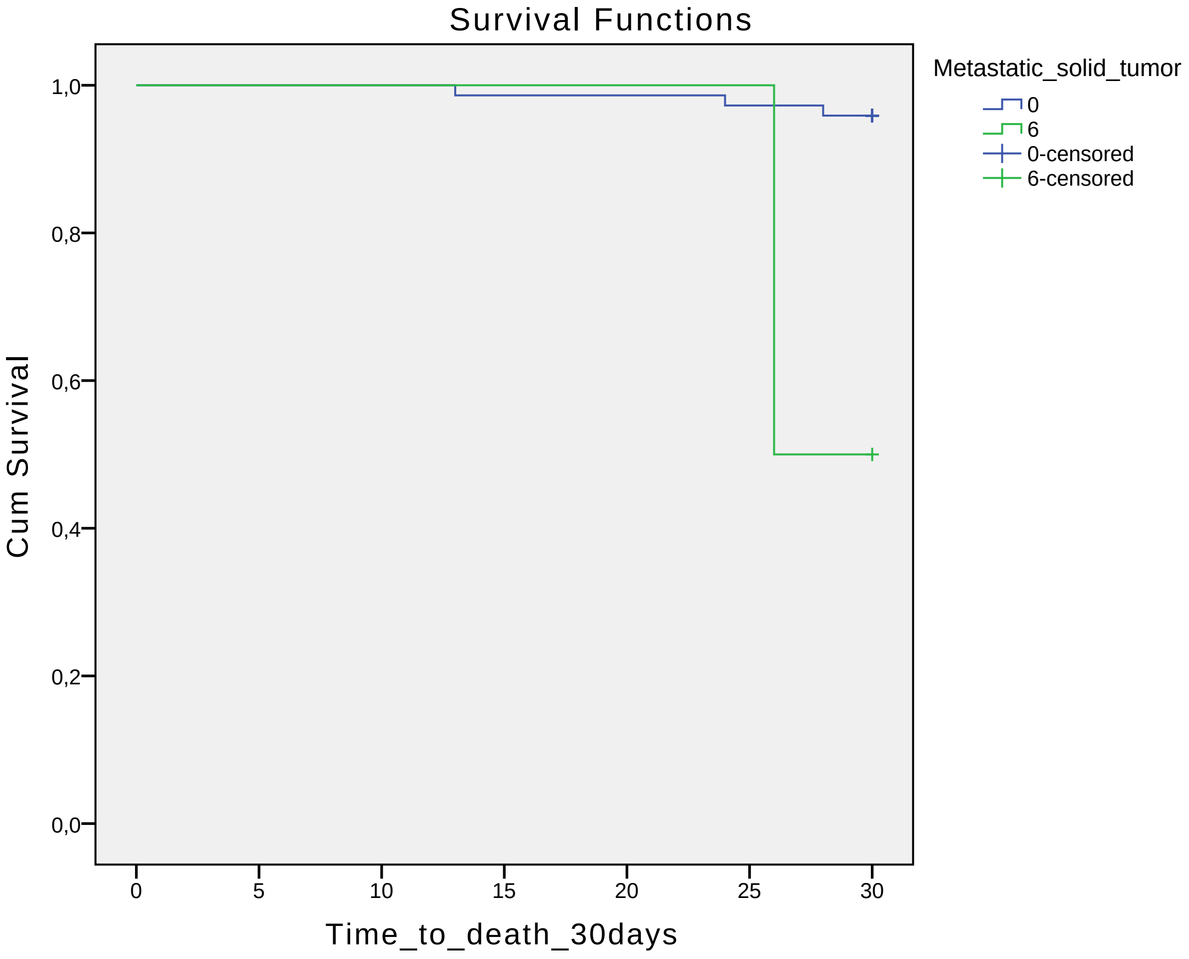

Kaplan-Meier

Case Processing Summary

Lymphoma	Total N	N of Events	Censored	
			N	Percent
0	73	3	70	95,9%
2	2	1	1	50,0%
Overall	75	4	71	94,7%

Means and Medians for Survival Time

Lymphoma	Mean[a]				Median			
	Estimate	Std. Error	95% Confidence Interval		Estimate	Std. Error	95% Confidence Interval	
			Lower Bound	Upper Bound			Lower Bound	Upper Bound
0	29,658	,245	29,177	30,138	.	.	.	.
2	28,000	1,414	25,228	30,772	26,000	.	.	.
Overall	29,613	,244	29,136	30,091	.	.	.	.

a. Estimation is limited to the largest survival time if it is censored.

Overall Comparisons

	Chi-Square	df	Sig.
Log Rank (Mantel-Cox)	8,846	1	,003
Breslow (Generalized Wilcoxon)	8,660	1	,003

Test of equality of survival distributions for the different levels of Lymphoma.

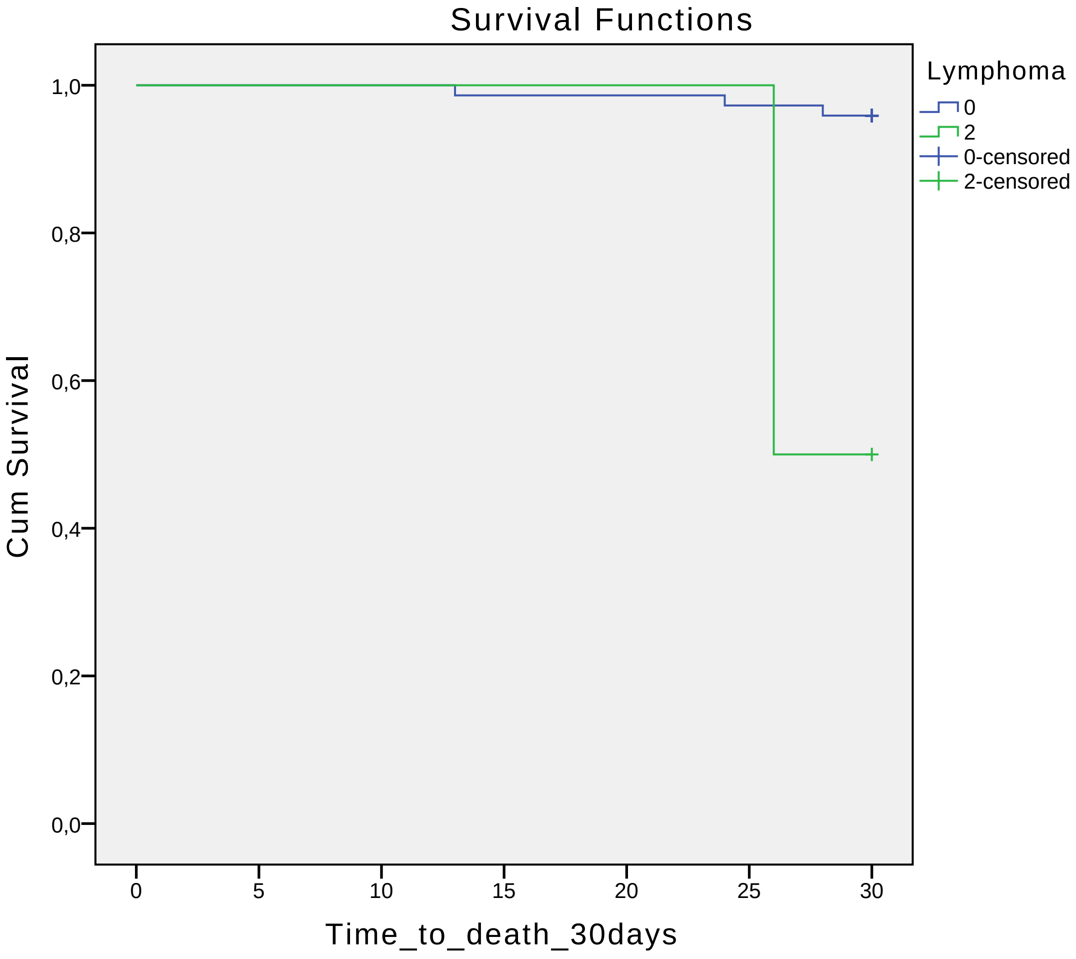

Kaplan-Meier

Warnings

No comparison analysis is performed because the factor variable has only one value for every stratum.

Case Processing Summary

Leukemia	Total N	N of Events	Censored	
			N	Percent
0	75	4	71	94,7%
Overall	75	4	71	94,7%

Means and Medians for Survival Time

Leukemia	Mean[a]				Median			
	Estimate	Std. Error	95% Confidence Interval		Estimate	Std. Error	95% Confidence Interval	
			Lower Bound	Upper Bound			Lower Bound	Upper Bound
0	29,613	,244	29,136	30,091	.	.	.	.
Overall	29,613	,244	29,136	30,091	.	.	.	.

a. Estimation is limited to the largest survival time if it is censored.

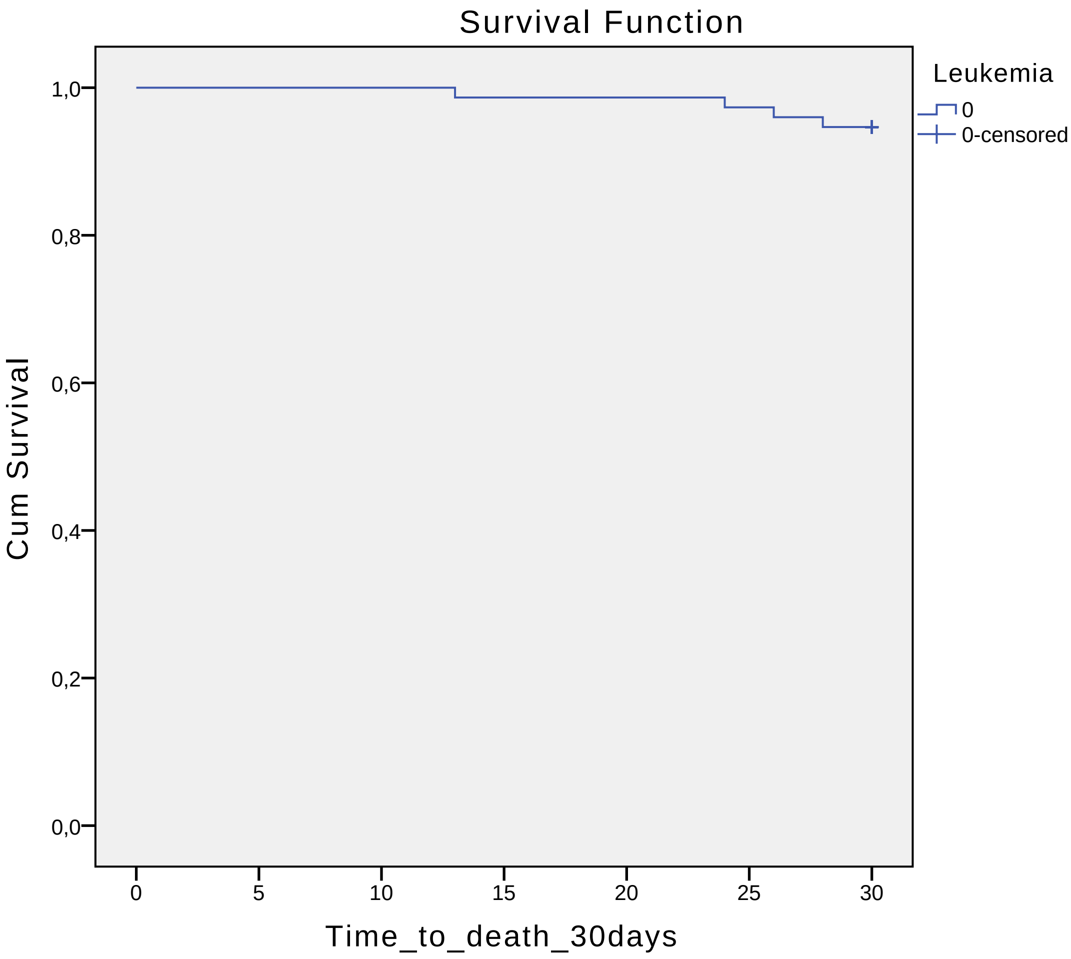

Kaplan-Meier

Case Processing Summary

Chronic_liver_disease	Total N	N of Events	Censored	
			N	Percent
0	67	3	64	95,5%
1	8	1	7	87,5%
Overall	75	4	71	94,7%

Means and Medians for Survival Time

Chronic_liver_disease	Mean[a]				Median		
			95% Confidence Interval				95% …
	Estimate	Std. Error	Lower Bound	Upper Bound	Estimate	Std. Error	Lower Bound
0	29,657	,259	29,149	30,164	.	.	.
1	29,250	,702	27,875	30,625	.	.	.
Overall	29,613	,244	29,136	30,091	.	.	.

Means and Medians for Survival Time

Chronic_liver_disease	Median
	95% …
	Upper Bound
0	.
1	.
Overall	.

a. Estimation is limited to the largest survival time if it is censored.

Overall Comparisons

	Chi-Square	df	Sig.
Log Rank (Mantel-Cox)	,957	1	,328
Breslow (Generalized Wilcoxon)	,977	1	,323

Test of equality of survival distributions for the different levels of Chronic_liver_disease.

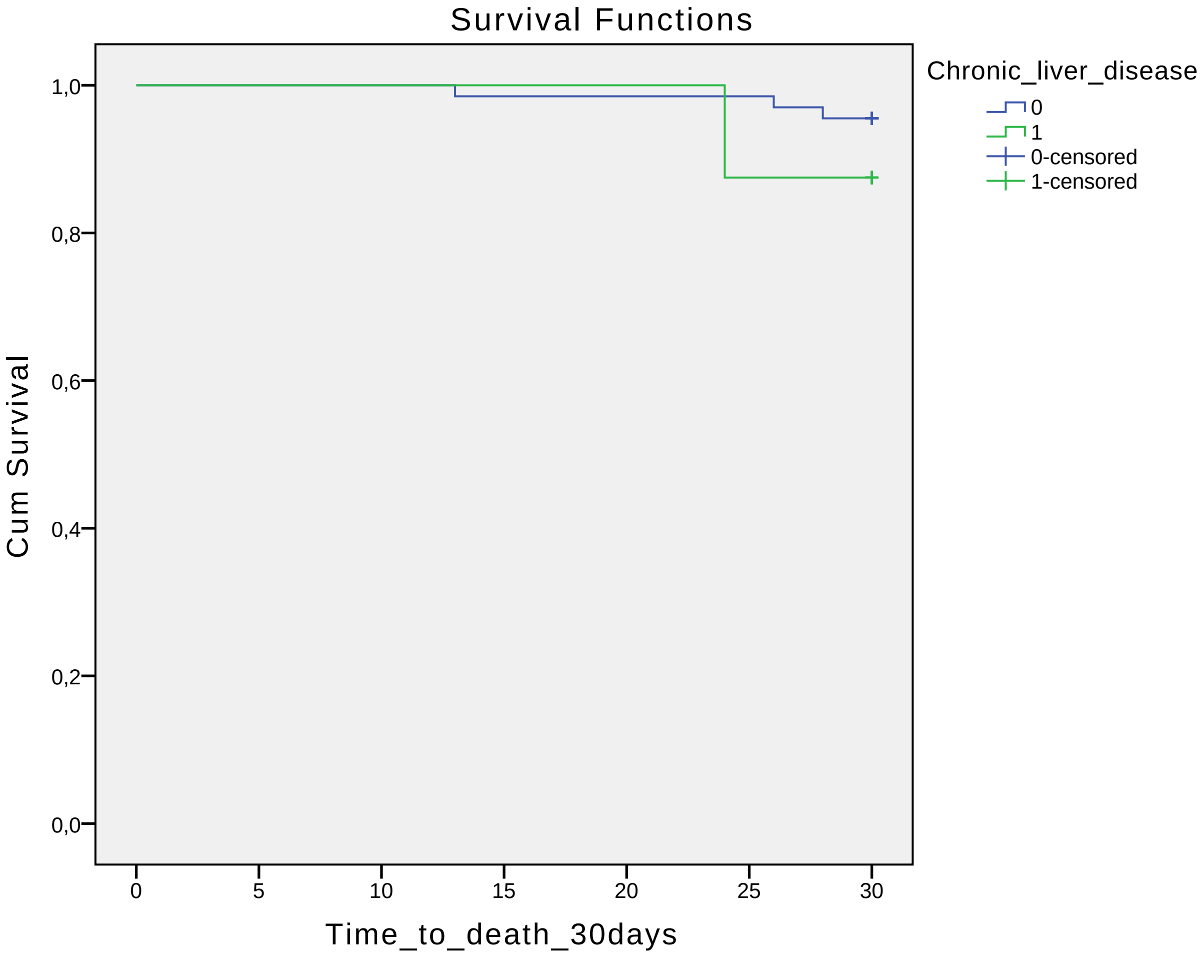

Kaplan-Meier

Case Processing Summary

Chronic_liver_disease_without_portal_hypertension	Total N	N of Events	Censored	
			N	Percent
0	69	3	66	95,7%
1	6	1	5	83,3%
Overall	75	4	71	94,7%

Means and Medians for Survival Time

Chronic_liver_disease_without_portal_hypertension	Mean[a]				Median		
	Estimate	Std. Error	95% Confidence Interval		Estimate	Std. Error	95% …
			Lower Bound	Upper Bound			Lower Bound
0	29,667	,252	29,174	30,160	.	.	.
1	29,000	,913	27,211	30,789	.	.	.
Overall	29,613	,244	29,136	30,091	.	.	.

Means and Medians for Survival Time

Chronic_liver_disease_without_portal_hypertension	Median
	95% ...
	Upper Bound
0	.
1	.
Overall	.

a. Estimation is limited to the largest survival time if it is censored.

Overall Comparisons

	Chi-Square	df	Sig.
Log Rank (Mantel-Cox)	1,777	1	,183
Breslow (Generalized Wilcoxon)	1,806	1	,179

Test of equality of survival distributions for the different levels of Chronic_liver_disease_without_portal_hypertension.

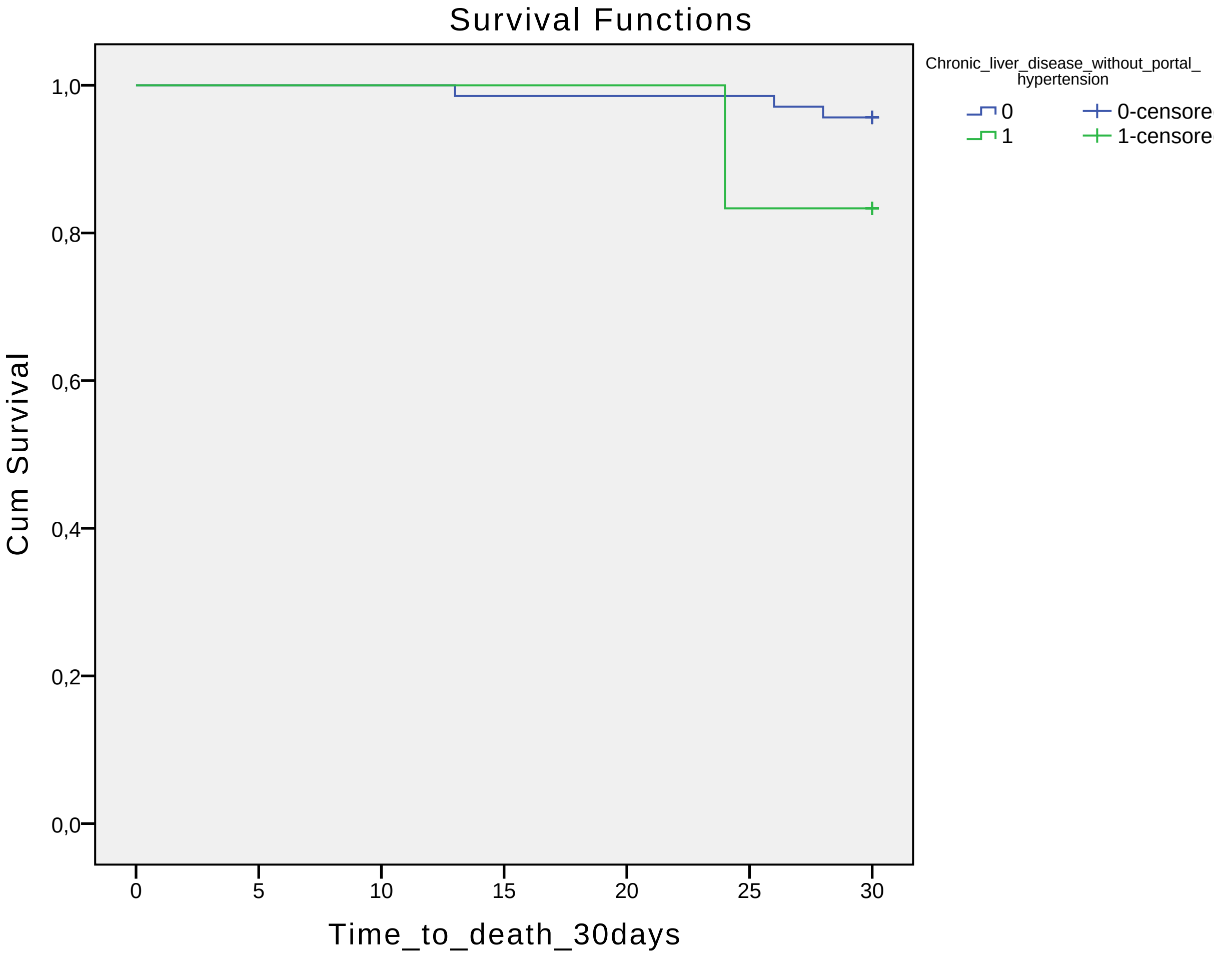

Kaplan-Meier

Warnings

No statistics are computed because all cases are censored.

Case Processing Summary

Chronic_liver_disease_with_portal_hypertension	Total N	N of Events	Censored	
			N	Percent
0	73	4	69	94,5%
3	2	0	2	100,0%
Overall	75	4	71	94,7%

Overall Comparisons

	Chi-Square	df	Sig.
Log Rank (Mantel-Cox)	,112	1	,738
Breslow (Generalized Wilcoxon)	,112	1	,738

Test of equality of survival distributions for the different levels of Chronic_liver_disease_with_portal_hypertension.

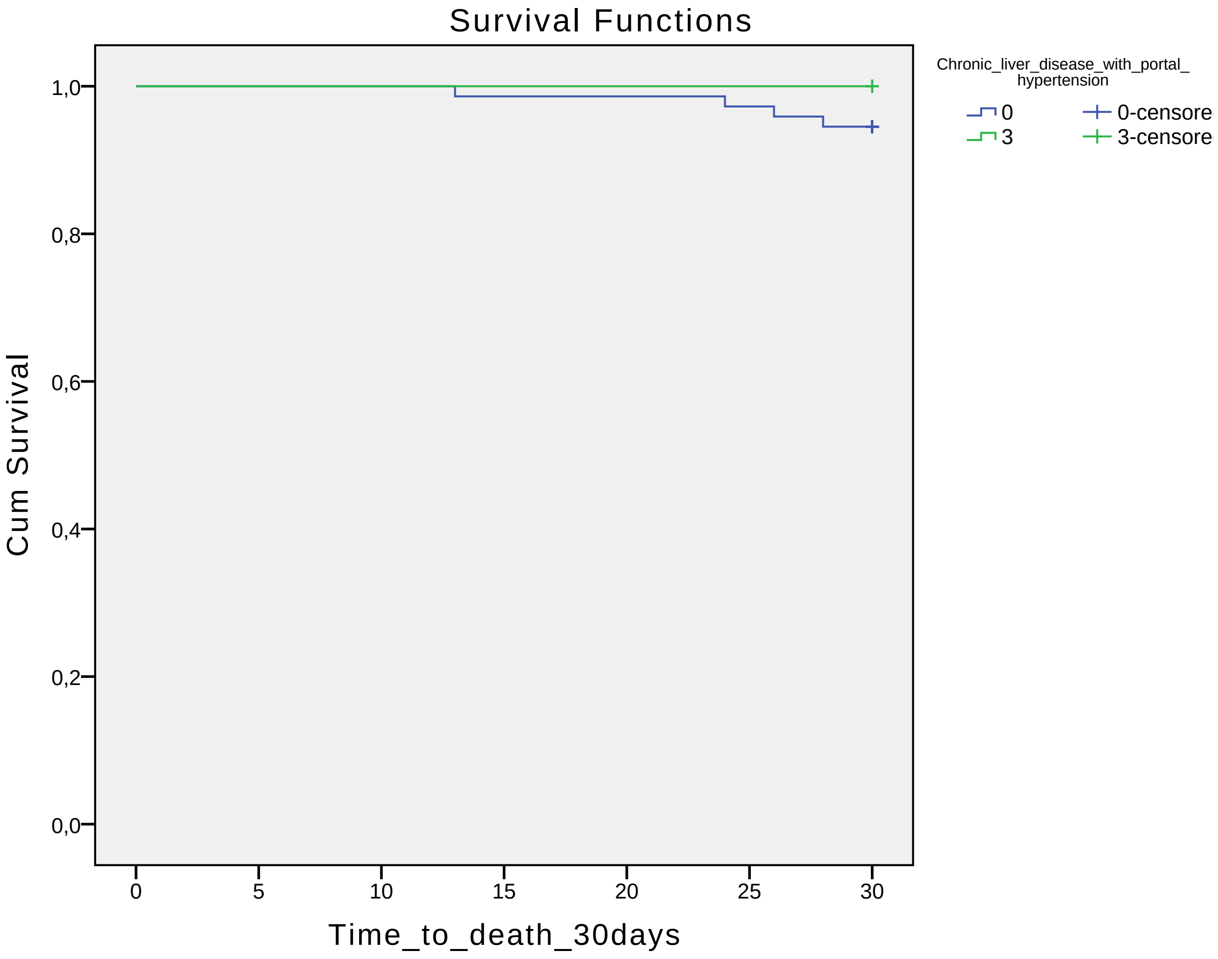

Kaplan-Meier

Warnings

No statistics are computed because all cases are censored.

Case Processing Summary

Connective_tissue_disease	Total N	N of Events	Censored	
			N	Percent
0	71	4	67	94,4%
1	4	0	4	100,0%
Overall	75	4	71	94,7%

Overall Comparisons

	Chi-Square	df	Sig.
Log Rank (Mantel-Cox)	,230	1	,631
Breslow (Generalized Wilcoxon)	,230	1	,631

Test of equality of survival distributions for the different levels of Connective_tissue_disease.

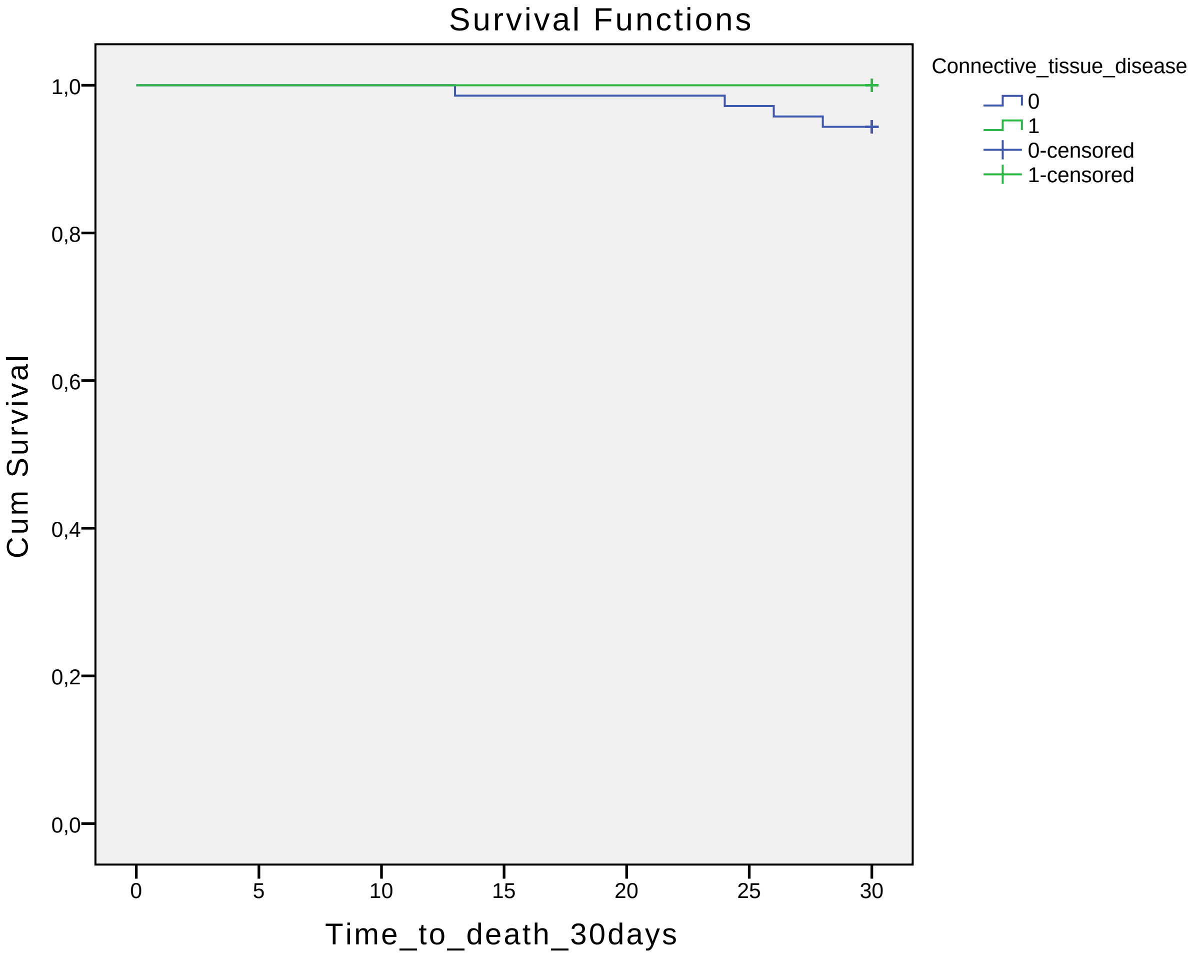

Kaplan-Meier

Case Processing Summary

Diabetes_mellitus	Total N	N of Events	Censored	
			N	Percent
0	51	3	48	94,1%
1	24	1	23	95,8%
Overall	75	4	71	94,7%

Means and Medians for Survival Time

Diabetes_mellitus	Mean[a]				Median		
			95% Confidence Interval				95% …
	Estimate	Std. Error	Lower Bound	Upper Bound	Estimate	Std. Error	Lower Bound
0	29,471	,354	28,776	30,165	.	.	.
1	29,917	,082	29,757	30,077	.	.	.
Overall	29,613	,244	29,136	30,091	.	.	.

Means and Medians for Survival Time

Diabetes_mellitus	Median 95% ... Upper Bound
0	.
1	.
Overall	.

a. Estimation is limited to the largest survival time if it is censored.

Overall Comparisons

	Chi-Square	df	Sig.
Log Rank (Mantel-Cox)	,107	1	,744
Breslow (Generalized Wilcoxon)	,121	1	,728

Test of equality of survival distributions for the different levels of Diabetes_mellitus.

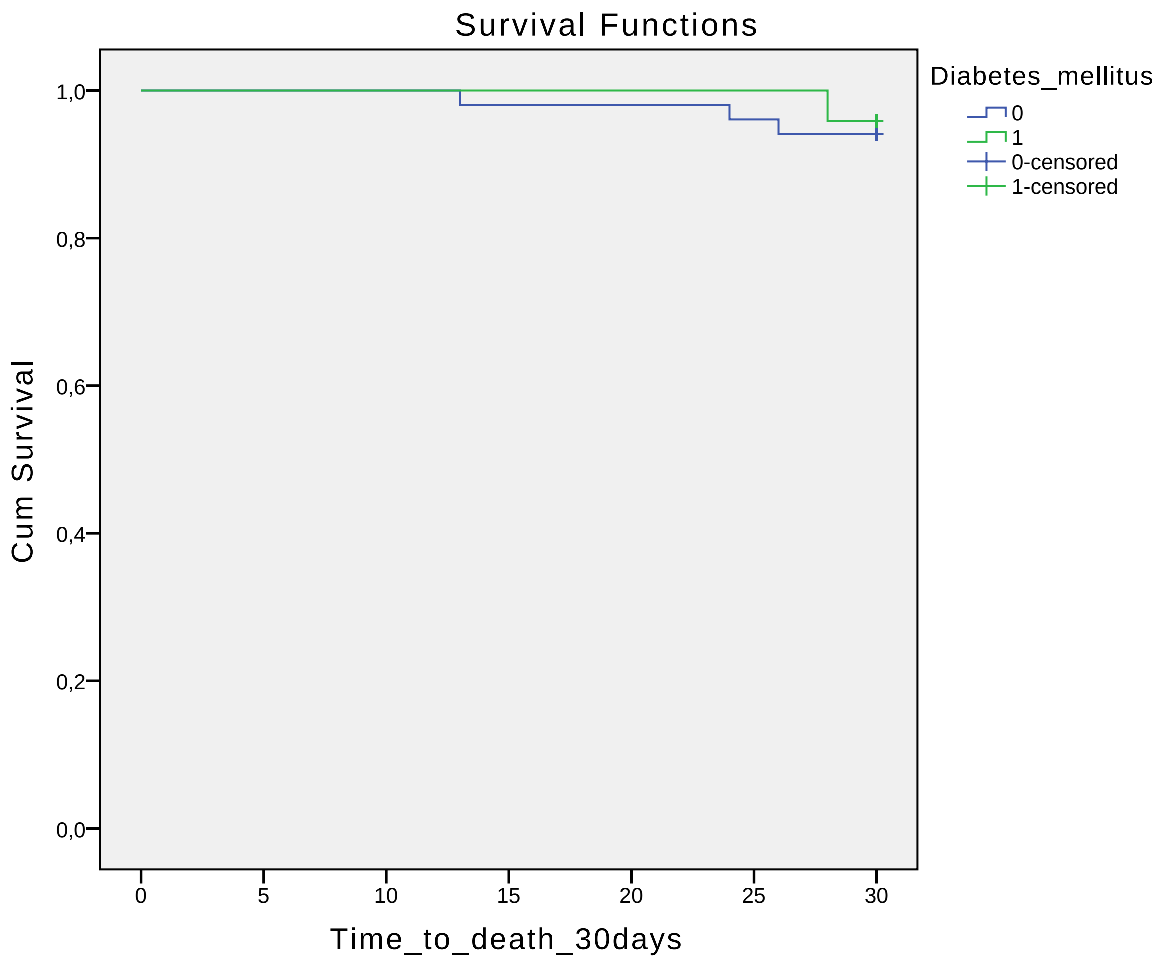

Kaplan-Meier

Warnings

No statistics are computed because all cases are censored.

Case Processing Summary

DM_with_target_organ_damage	Total N	N of Events	Censored	
			N	Percent
0	67	4	63	94,0%
2	8	0	8	100,0%
Overall	75	4	71	94,7%

Overall Comparisons

	Chi-Square	df	Sig.
Log Rank (Mantel-Cox)	,489	1	,485
Breslow (Generalized Wilcoxon)	,489	1	,485

Test of equality of survival distributions for the different levels of DM_with_target_organ_damage.

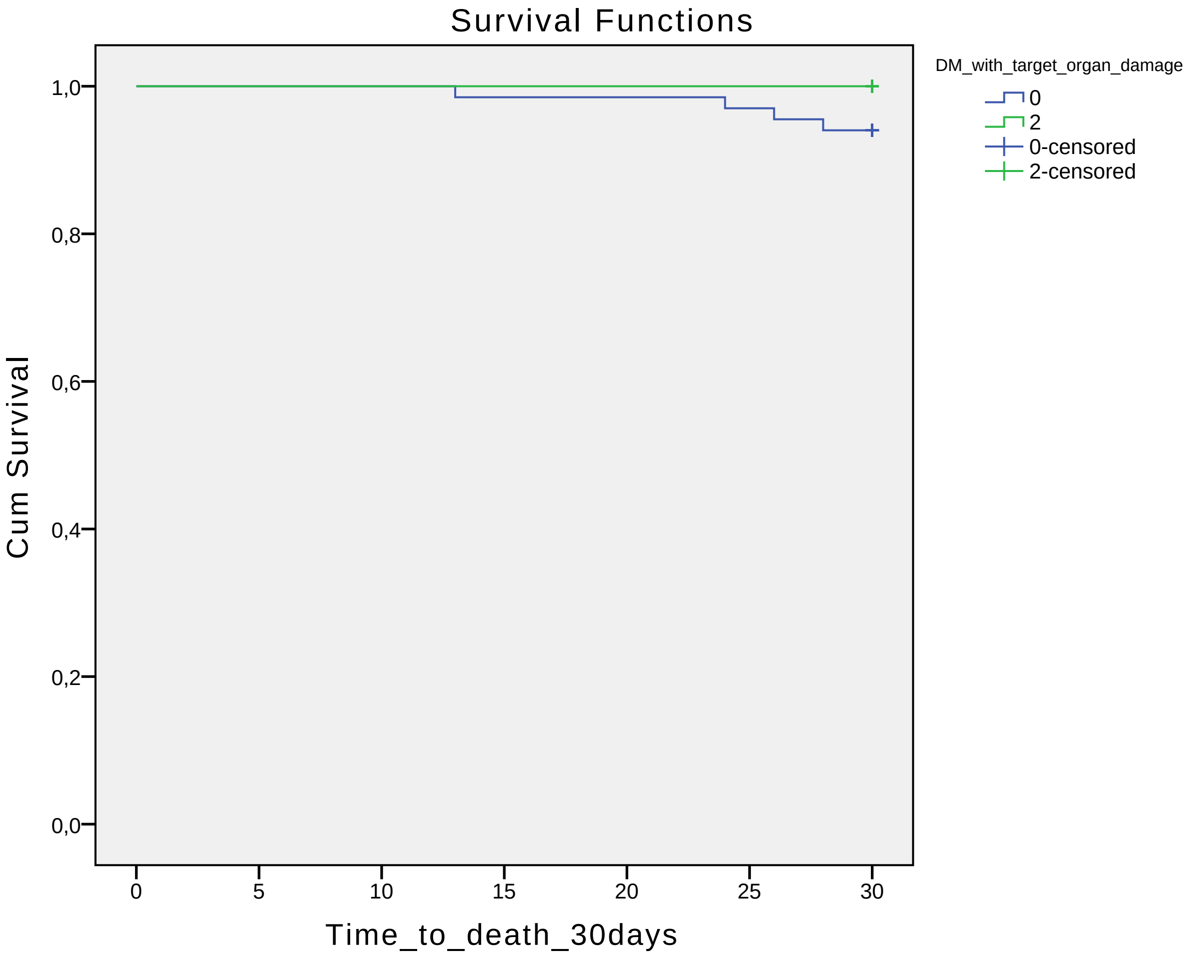

Kaplan-Meier

Case Processing Summary

Hypertension	Total N	N of Events	Censored	
			N	Percent
0	31	1	30	96,8%
1	44	3	41	93,2%
Overall	75	4	71	94,7%

Means and Medians for Survival Time

Hypertension	Mean[a]				Median		
	Estimate	Std. Error	95% Confidence Interval		Estimate	Std. Error	95% …
			Lower Bound	Upper Bound			Lower Bound
0	29,452	,539	28,394	30,509	.	.	.
1	29,727	,165	29,404	30,051	.	.	.
Overall	29,613	,244	29,136	30,091	.	.	.

Means and Medians for Survival Time

Hypertension	Median
	95% …
	Upper Bound
0	.
1	.
Overall	.

a. Estimation is limited to the largest survival time if it is censored.

Overall Comparisons

	Chi-Square	df	Sig.
Log Rank (Mantel-Cox)	,431	1	,511
Breslow (Generalized Wilcoxon)	,404	1	,525

Test of equality of survival distributions for the different levels of Hypertension.

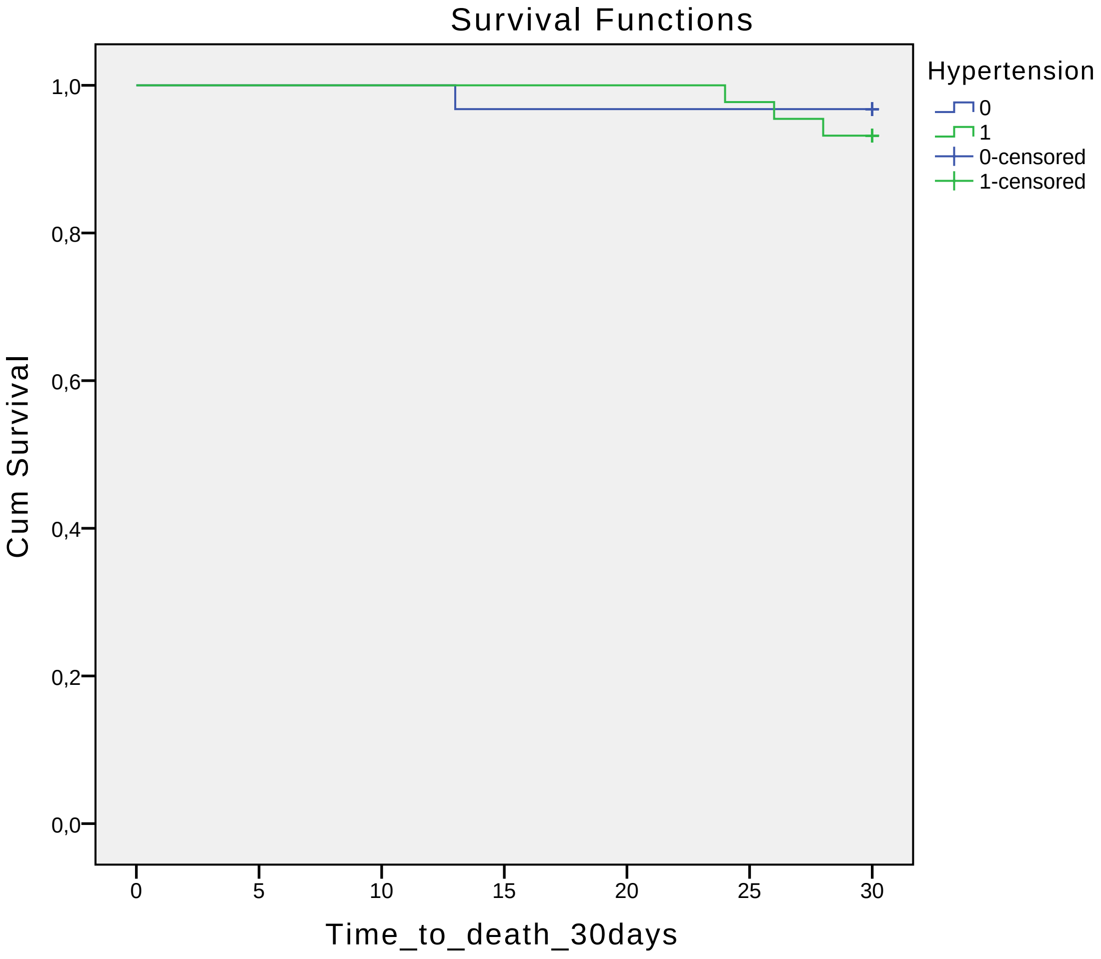

Kaplan-Meier

Case Processing Summary

Dyslipidemia	Total N	N of Events	Censored	
			N	Percent
0	42	2	40	95,2%
1	33	2	31	93,9%
Overall	75	4	71	94,7%

Means and Medians for Survival Time

Dyslipidemia	Mean[a]				Median		
	Estimate	Std. Error	95% Confidence Interval		Estimate	Std. Error	95% …
			Lower Bound	Upper Bound			Lower Bound
0	29,548	,402	28,761	30,335	.	.	.
1	29,697	,212	29,281	30,113	.	.	.
Overall	29,613	,244	29,136	30,091	.	.	.

Means and Medians for Survival Time

Dyslipidemia	Median
	95% …
	Upper Bound
0	.
1	.
Overall	.

a. Estimation is limited to the largest survival time if it is censored.

Overall Comparisons

	Chi-Square	df	Sig.
Log Rank (Mantel-Cox)	,061	1	,805
Breslow (Generalized Wilcoxon)	,061	1	,805

Test of equality of survival distributions for the different levels of Dyslipidemia.

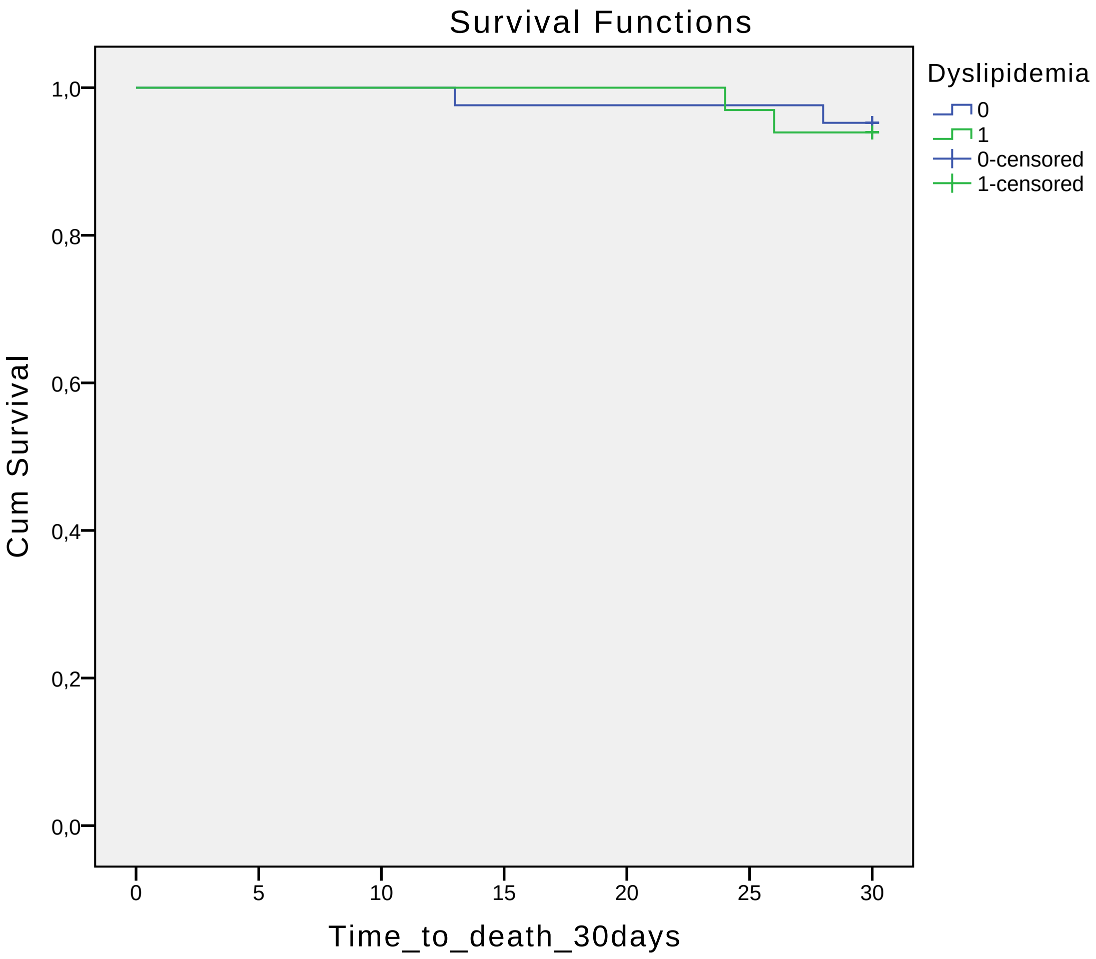

Kaplan-Meier

Warnings

No statistics are computed because all cases are censored.

Case Processing Summary

Chronic_respiratory_disease	Total N	N of Events	Censored	
			N	Percent
0	64	4	60	93,8%
1	11	0	11	100,0%
Overall	75	4	71	94,7%

Overall Comparisons

	Chi-Square	df	Sig.
Log Rank (Mantel-Cox)	,704	1	,401
Breslow (Generalized Wilcoxon)	,704	1	,401

Test of equality of survival distributions for the different levels of Chronic_respiratory_disease.

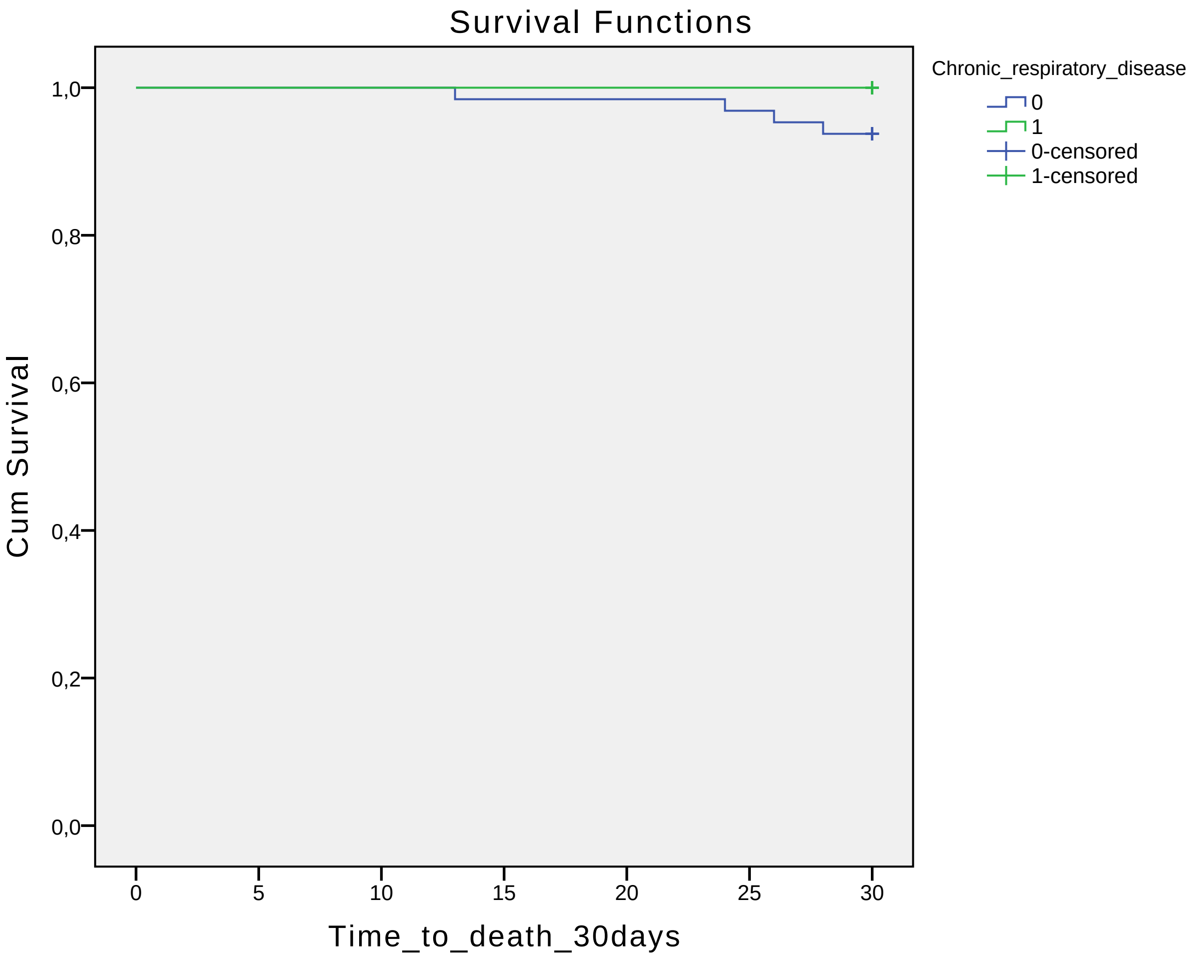

Kaplan-Meier

Warnings

No statistics are computed because all cases are censored.

Case Processing Summary

COPD	Total N	N of Events	Censored	
			N	Percent
0	70	4	66	94,3%
1	5	0	5	100,0%
Overall	75	4	71	94,7%

Overall Comparisons

	Chi-Square	df	Sig.
Log Rank (Mantel-Cox)	,292	1	,589
Breslow (Generalized Wilcoxon)	,292	1	,589

Test of equality of survival distributions for the different levels of COPD.

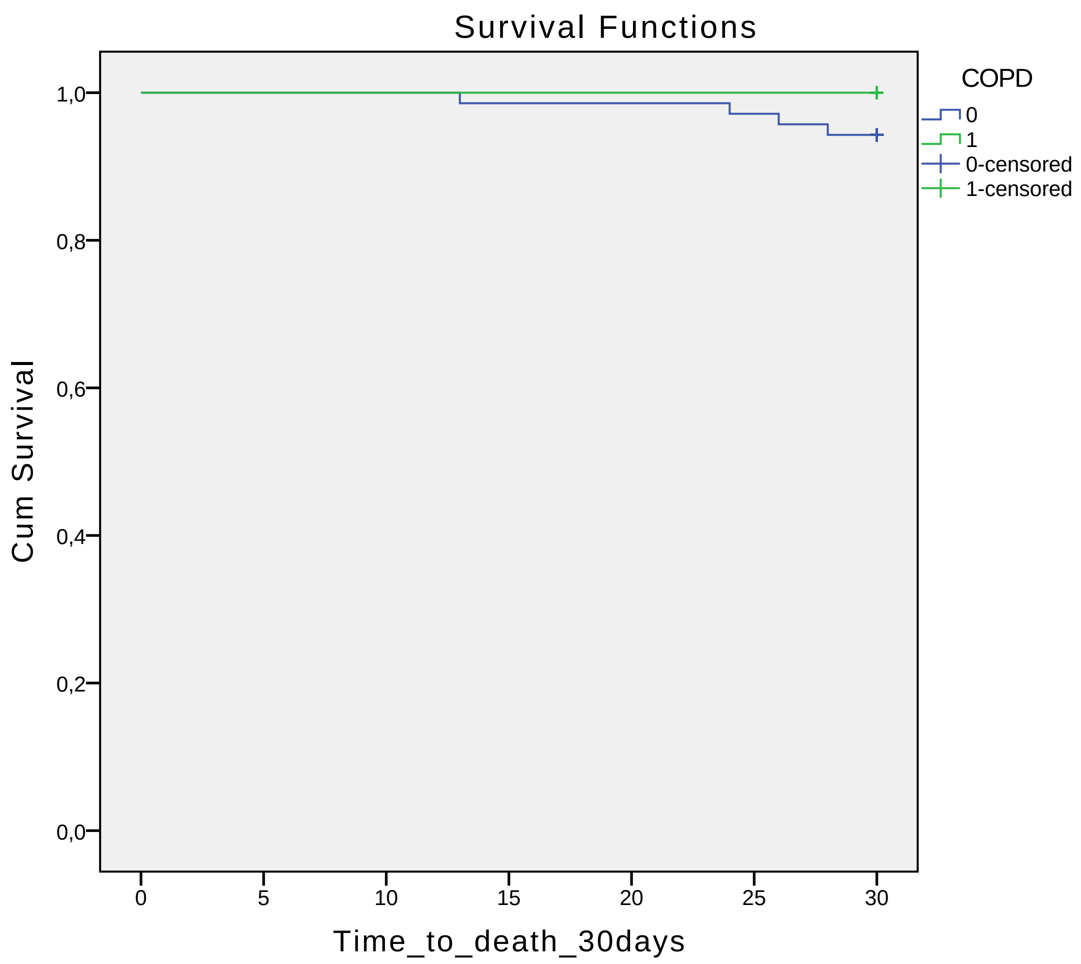

Kaplan-Meier

Case Processing Summary

Heart_failure	Total N	N of Events	Censored	
			N	Percent
0	63	3	60	95,2%
1	12	1	11	91,7%
Overall	75	4	71	94,7%

Means and Medians for Survival Time

Heart_failure	Mean[a]				Median		
			95% Confidence Interval				95% …
	Estimate	Std. Error	Lower Bound	Upper Bound	Estimate	Std. Error	Lower Bound
0	29,635	,275	29,096	30,174	.	.	.
1	29,500	,479	28,562	30,438	.	.	.
Overall	29,613	,244	29,136	30,091	.	.	.

Means and Medians for Survival Time

Heart_failure	Median
	95% …
	Upper Bound
0	.
1	.
Overall	.

a. Estimation is limited to the largest survival time if it is censored.

Overall Comparisons

	Chi-Square	df	Sig.
Log Rank (Mantel-Cox)	,266	1	,606
Breslow (Generalized Wilcoxon)	,275	1	,600

Test of equality of survival distributions for the different levels of Heart_failure.

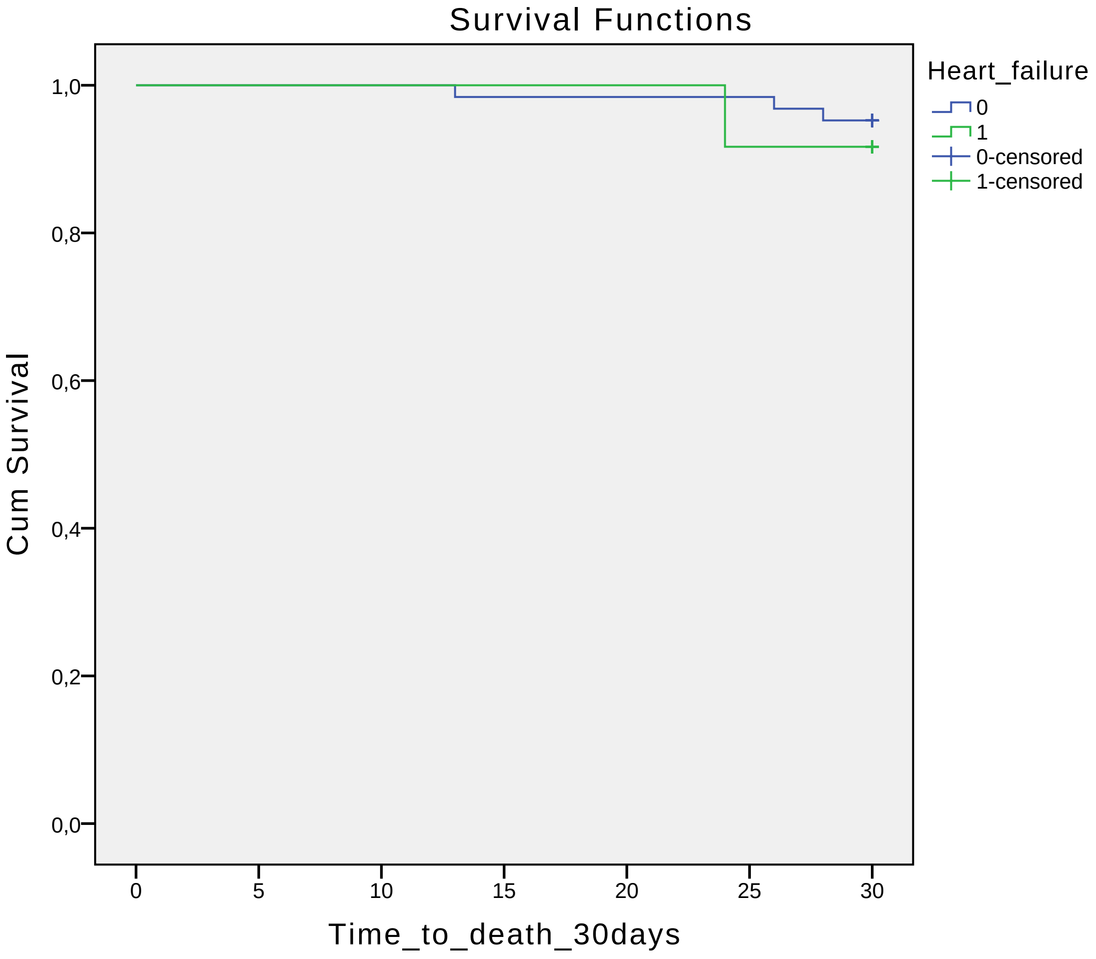

Kaplan-Meier

Warnings

No statistics are computed because all cases are censored.

Case Processing Summary

Myocardial_infarction	Total N	N of Events	Censored	
			N	Percent
0	66	4	62	93,9%
1	9	0	9	100,0%
Overall	75	4	71	94,7%

Overall Comparisons

	Chi-Square	df	Sig.
Log Rank (Mantel-Cox)	,558	1	,455
Breslow (Generalized Wilcoxon)	,558	1	,455

Test of equality of survival distributions for the different levels of Myocardial_infarction.

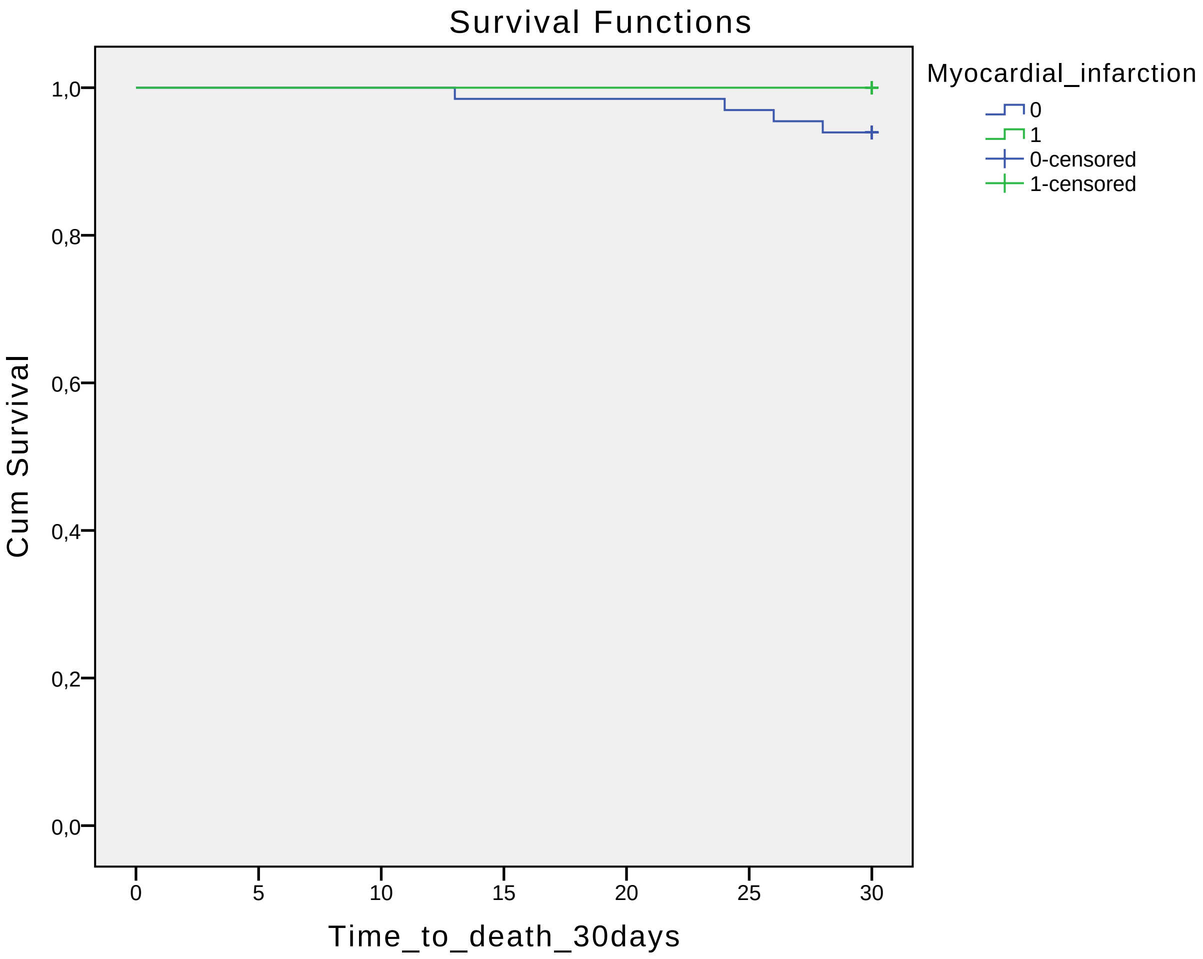

Kaplan-Meier

Warnings

No statistics are computed because all cases are censored.

Case Processing Summary

Peripheral_arterial_disease	Total N	N of Events	Censored	
			N	Percent
0	67	4	63	94,0%
1	8	0	8	100,0%
Overall	75	4	71	94,7%

Overall Comparisons

	Chi-Square	df	Sig.
Log Rank (Mantel-Cox)	,489	1	,485
Breslow (Generalized Wilcoxon)	,489	1	,485

Test of equality of survival distributions for the different levels of Peripheral_arterial_disease.

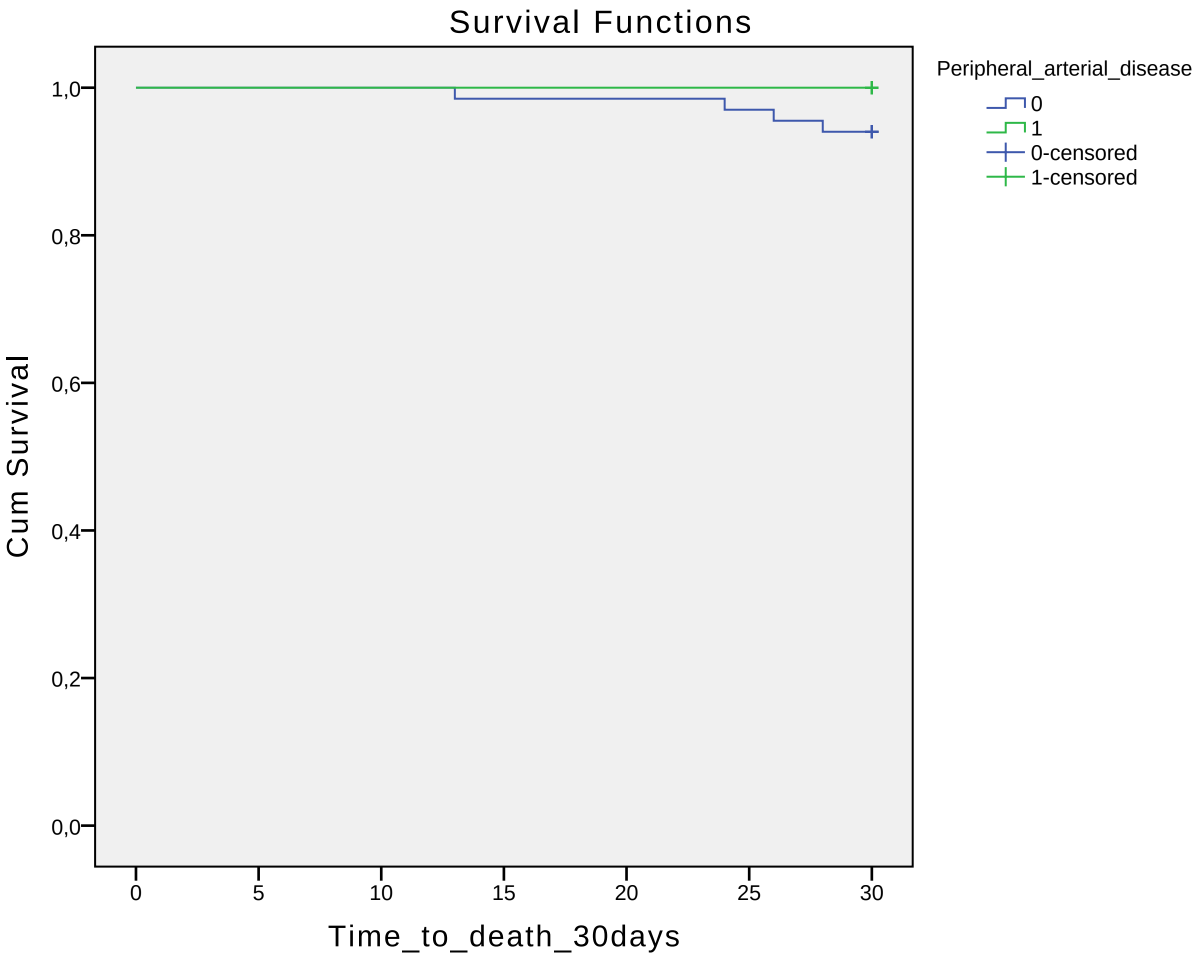

Kaplan-Meier

Warnings

No statistics are computed because all cases are censored.

Case Processing Summary

Cerebrovascular_disease	Total N	N of Events	Censored	
			N	Percent
0	59	4	55	93,2%
1	16	0	16	100,0%
Overall	75	4	71	94,7%

Overall Comparisons

	Chi-Square	df	Sig.
Log Rank (Mantel-Cox)	1,113	1	,291
Breslow (Generalized Wilcoxon)	1,113	1	,291

Test of equality of survival distributions for the different levels of Cerebrovascular_disease.

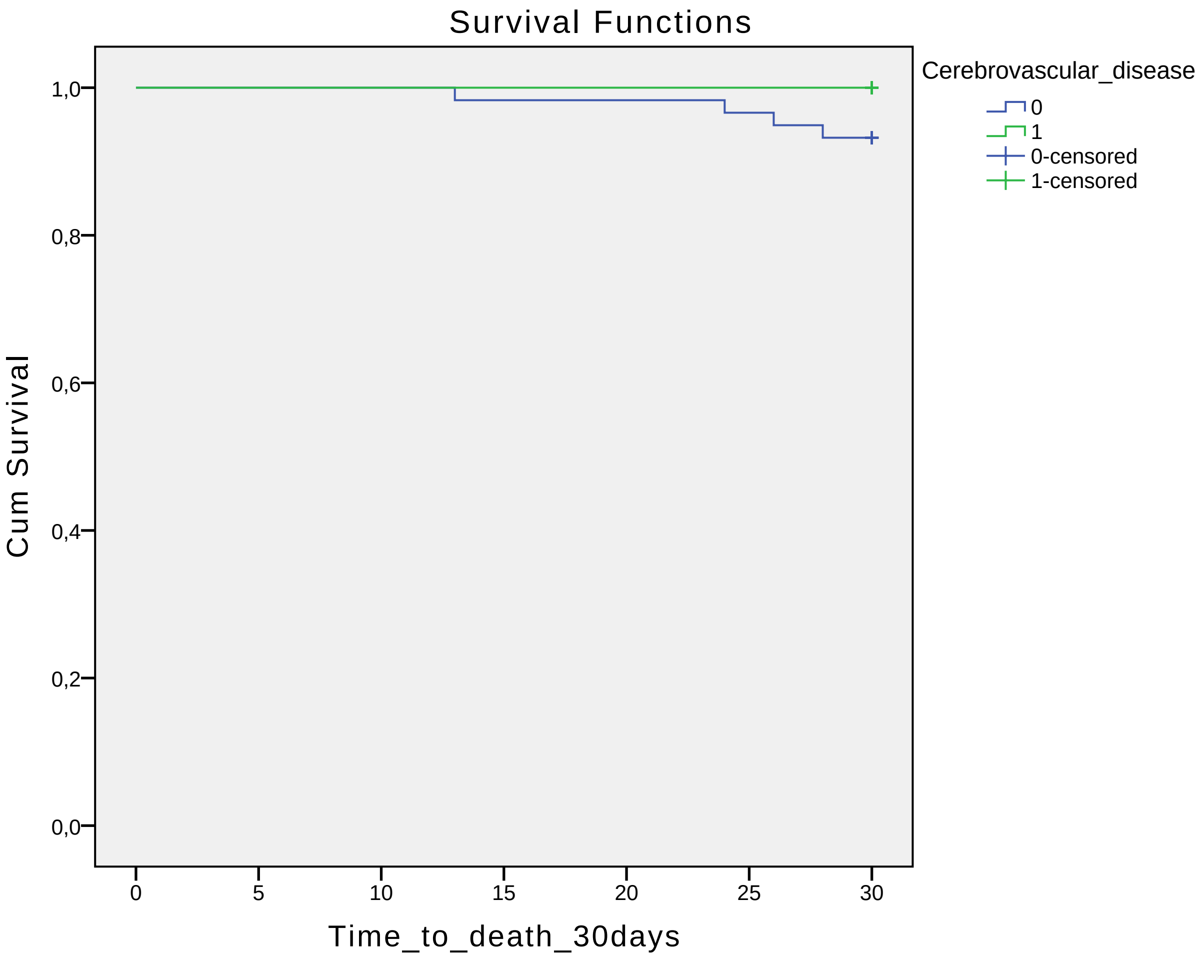

Kaplan-Meier

Warnings

No statistics are computed because all cases are censored.

Case Processing Summary

Hemiplegia	Total N	N of Events	Censored	
			N	Percent
0	65	4	61	93,8%
2	10	0	10	100,0%
Overall	75	4	71	94,7%

Overall Comparisons

	Chi-Square	df	Sig.
Log Rank (Mantel-Cox)	,630	1	,427
Breslow (Generalized Wilcoxon)	,630	1	,427

Test of equality of survival distributions for the different levels of Hemiplegia.

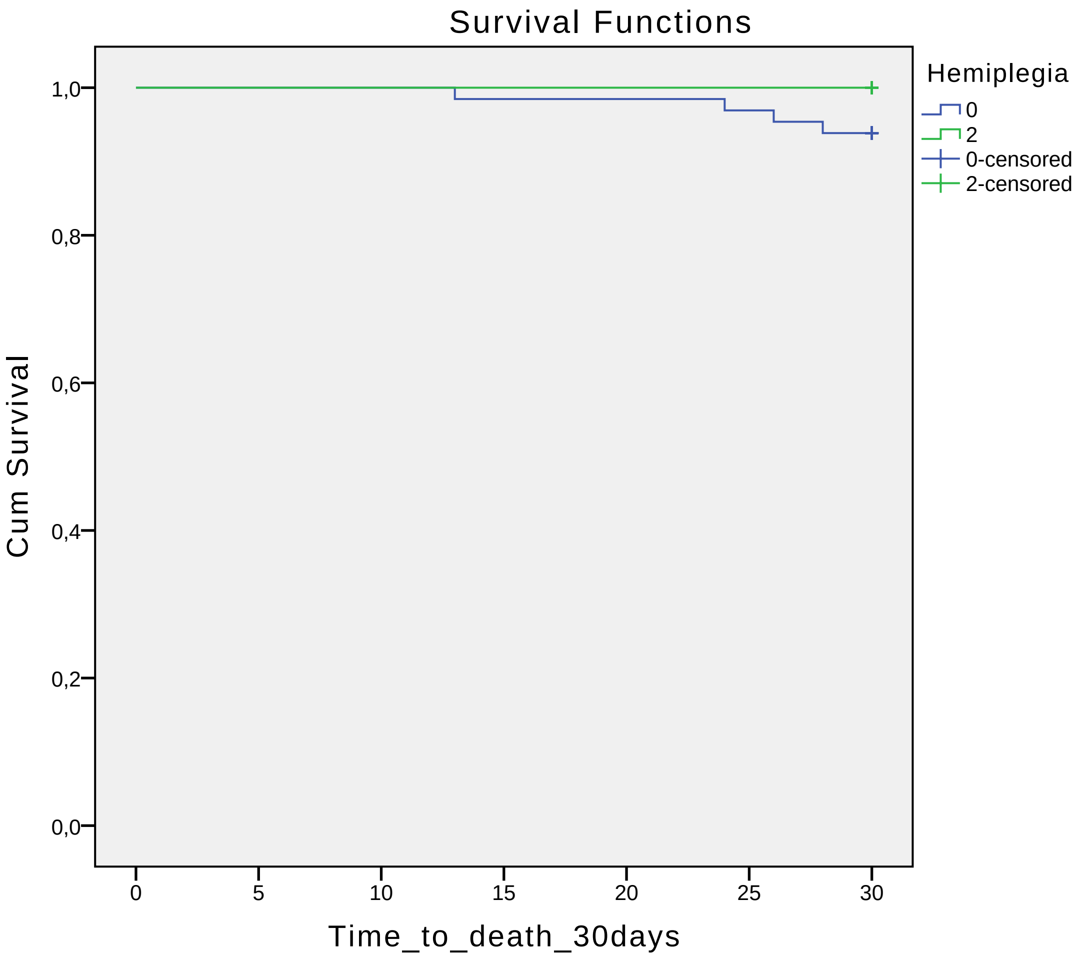

Kaplan-Meier

Warnings

No statistics are computed because all cases are censored.

Case Processing Summary

Gastroduodenal_ulcer	Total N	N of Events	Censored	
			N	Percent
0	69	4	65	94,2%
1	6	0	6	100,0%
Overall	75	4	71	94,7%

Overall Comparisons

	Chi-Square	df	Sig.
Log Rank (Mantel-Cox)	,356	1	,551
Breslow (Generalized Wilcoxon)	,356	1	,551

Test of equality of survival distributions for the different levels of Gastroduodenal_ulcer.

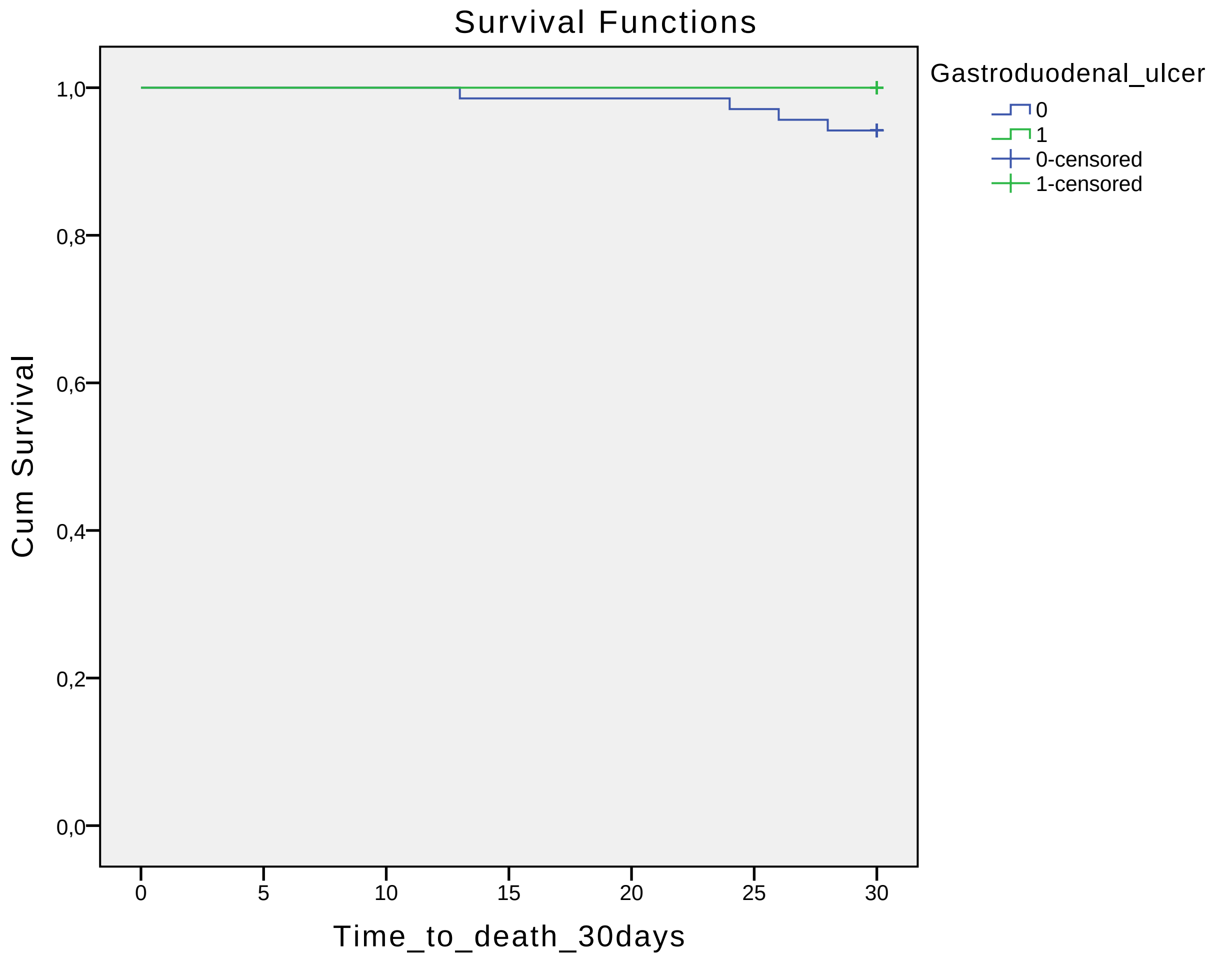

Kaplan-Meier

Warnings

No statistics are computed because all cases are censored.

Case Processing Summary

Chronic_kidney_disease	Total N	N of Events	Censored	
			N	Percent
0	55	4	51	92,7%
1	20	0	20	100,0%
Overall	75	4	71	94,7%

Overall Comparisons

	Chi-Square	df	Sig.
Log Rank (Mantel-Cox)	1,496	1	,221
Breslow (Generalized Wilcoxon)	1,495	1	,221

Test of equality of survival distributions for the different levels of Chronic_kidney_disease.

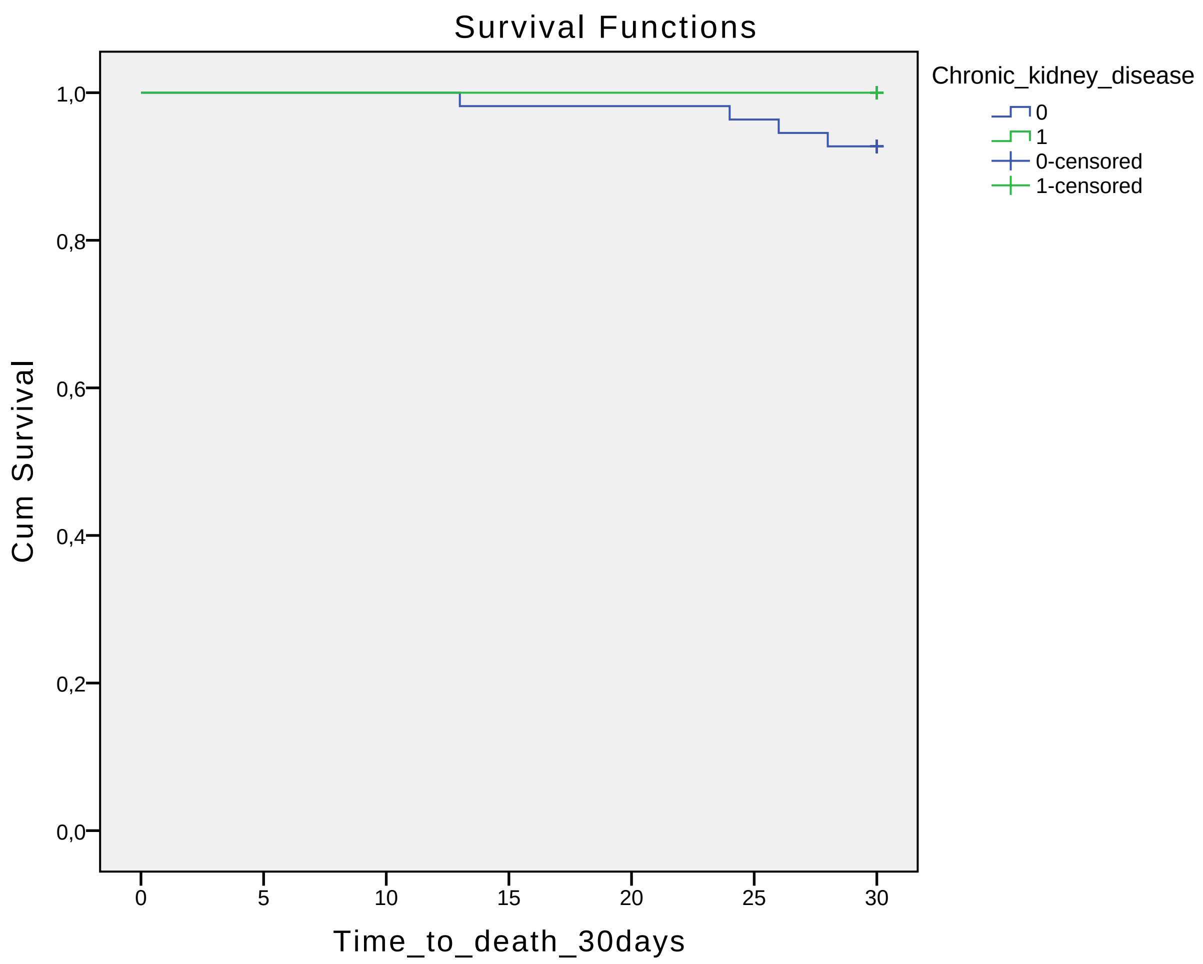

Kaplan-Meier

Warnings

No statistics are computed because all cases are censored.

Case Processing Summary

Moderate_severe_chronic_kidney_disease	Total N	N of Events	Censored	
			N	Percent
0	66	4	62	93,9%
2	9	0	9	100,0%
Overall	75	4	71	94,7%

Overall Comparisons

	Chi-Square	df	Sig.
Log Rank (Mantel-Cox)	,558	1	,455
Breslow (Generalized Wilcoxon)	,558	1	,455

Test of equality of survival distributions for the different levels of Moderate_severe_chronic_kidney_disease.

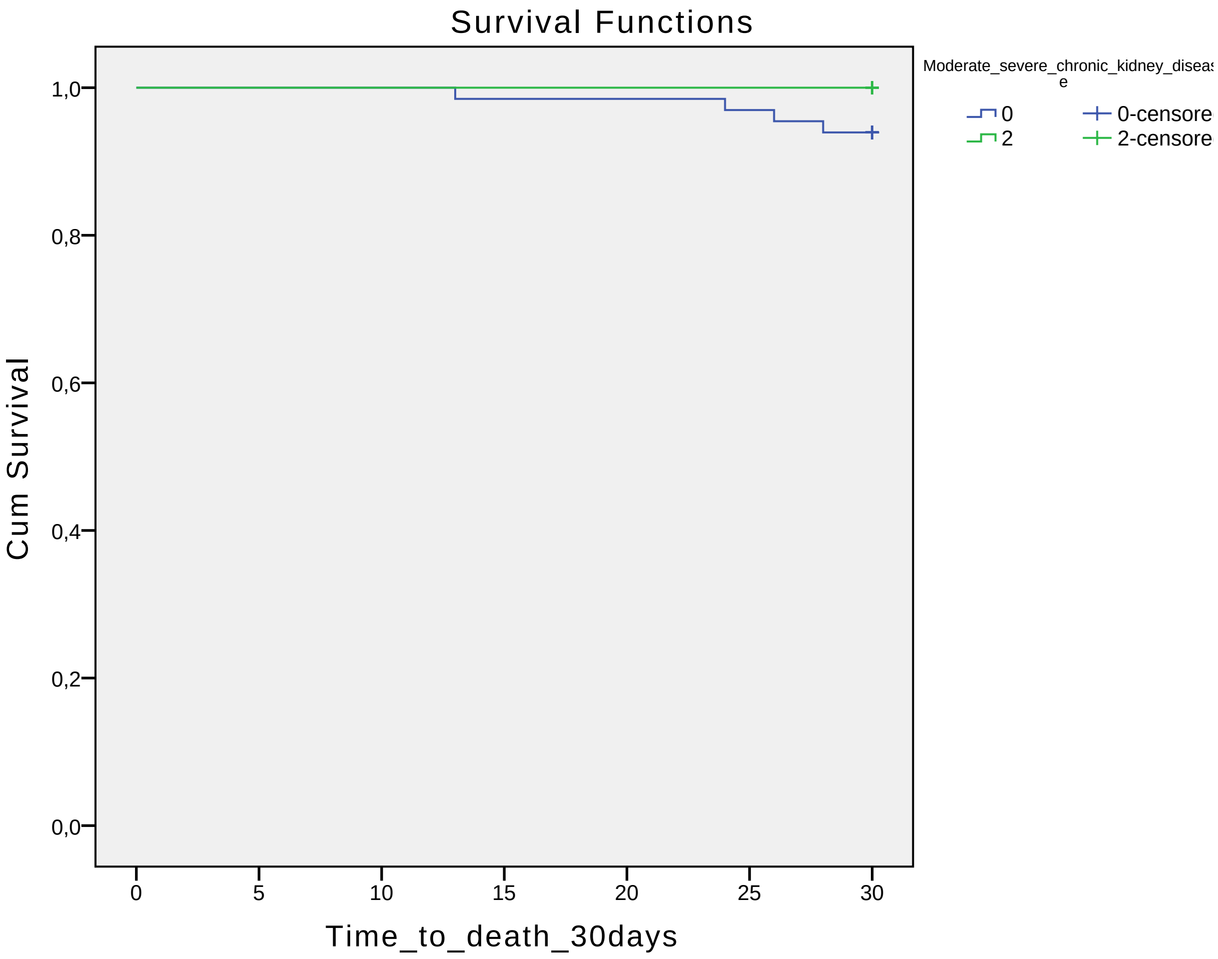

Kaplan-Meier

Warnings

No comparison analysis is performed because the factor variable has only one value for every stratum.

Case Processing Summary

Dyalisis	Total N	N of Events	Censored	
			N	Percent
0	75	4	71	94,7%
Overall	75	4	71	94,7%

Means and Medians for Survival Time

Dyalisis	Mean[a]				Median			
			95% Confidence Interval				95% Confidence Interval	
	Estimate	Std. Error	Lower Bound	Upper Bound	Estimate	Std. Error	Lower Bound	Upper Bound
0	29,613	,244	29,136	30,091	.	.	.	.
Overall	29,613	,244	29,136	30,091	.	.	.	.

a. Estimation is limited to the largest survival time if it is censored.

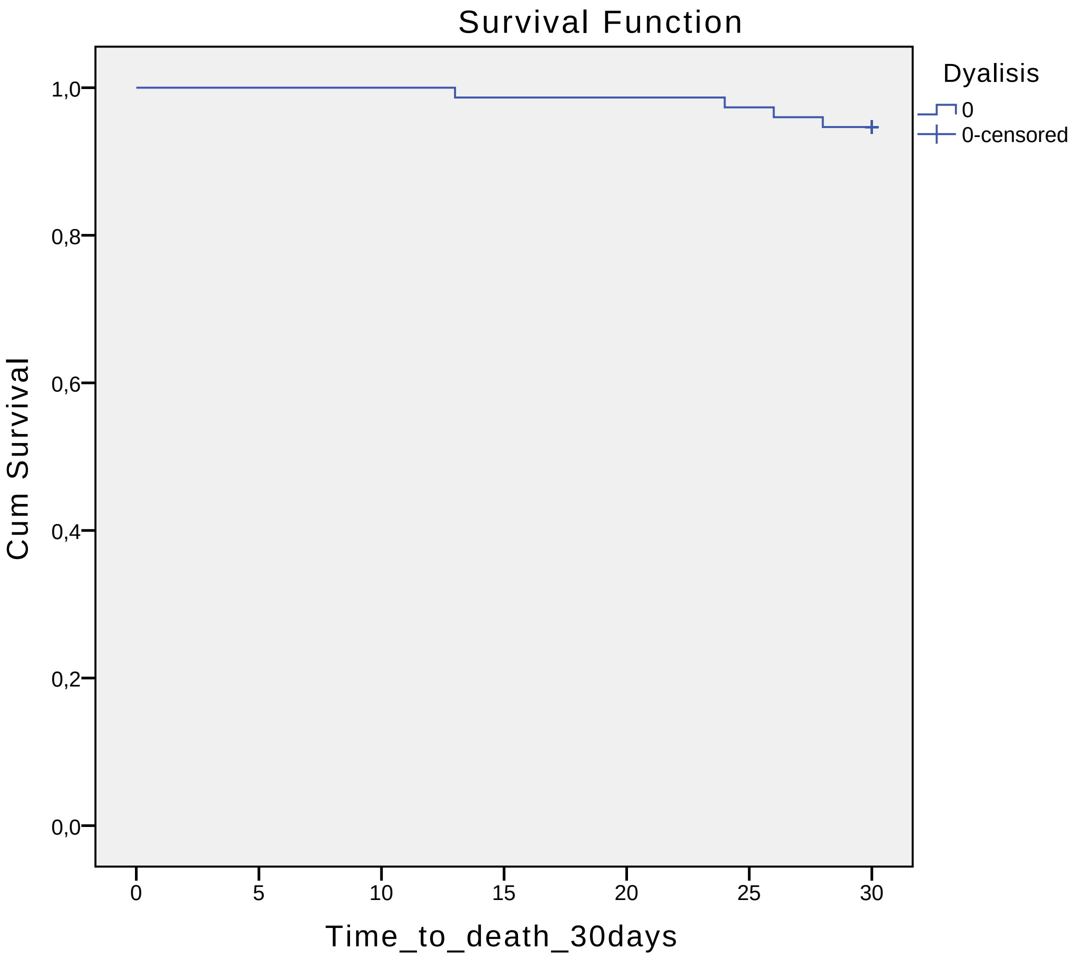

Kaplan-Meier

Warnings

No comparison analysis is performed because the factor variable has only one value for every stratum.

Case Processing Summary

HIV	Total N	N of Events	Censored	
			N	Percent
0	75	4	71	94,7%
Overall	75	4	71	94,7%

Means and Medians for Survival Time

HIV	Mean[a]				Median			
	Estimate	Std. Error	95% Confidence Interval		Estimate	Std. Error	95% Confidence Interval	
			Lower Bound	Upper Bound			Lower Bound	Upper Bound
0	29,613	,244	29,136	30,091	.	.	.	.
Overall	29,613	,244	29,136	30,091	.	.	.	.

a. Estimation is limited to the largest survival time if it is censored.

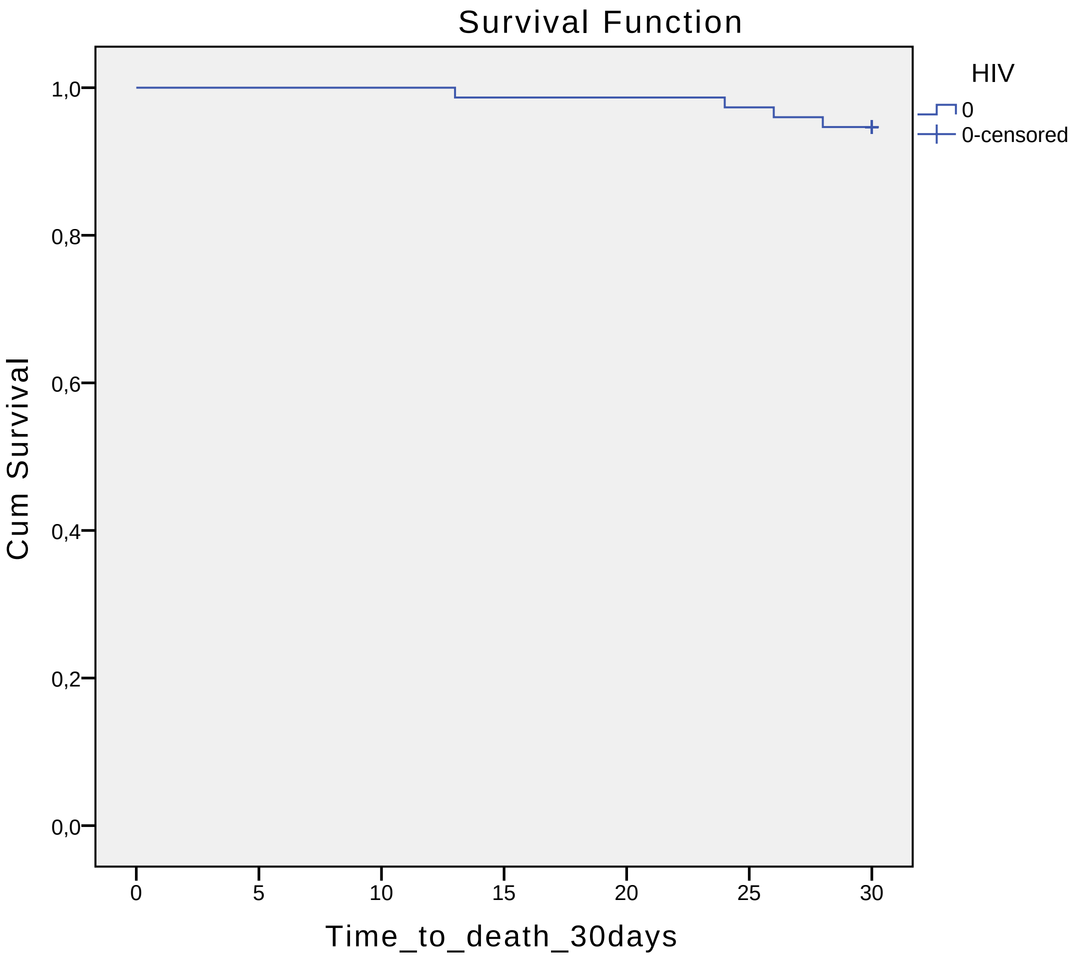

Kaplan-Meier

Warnings

No comparison analysis is performed because the factor variable has only one value for every stratum.

Case Processing Summary

AIDS	Total N	N of Events	Censored	
			N	Percent
0	75	4	71	94,7%
Overall	75	4	71	94,7%

Means and Medians for Survival Time

AIDS	Mean[a]				Median			
	Estimate	Std. Error	95% Confidence Interval		Estimate	Std. Error	95% Confidence Interval	
			Lower Bound	Upper Bound			Lower Bound	Upper Bound
0	29,613	,244	29,136	30,091	.	.	.	.
Overall	29,613	,244	29,136	30,091	.	.	.	.

a. Estimation is limited to the largest survival time if it is censored.

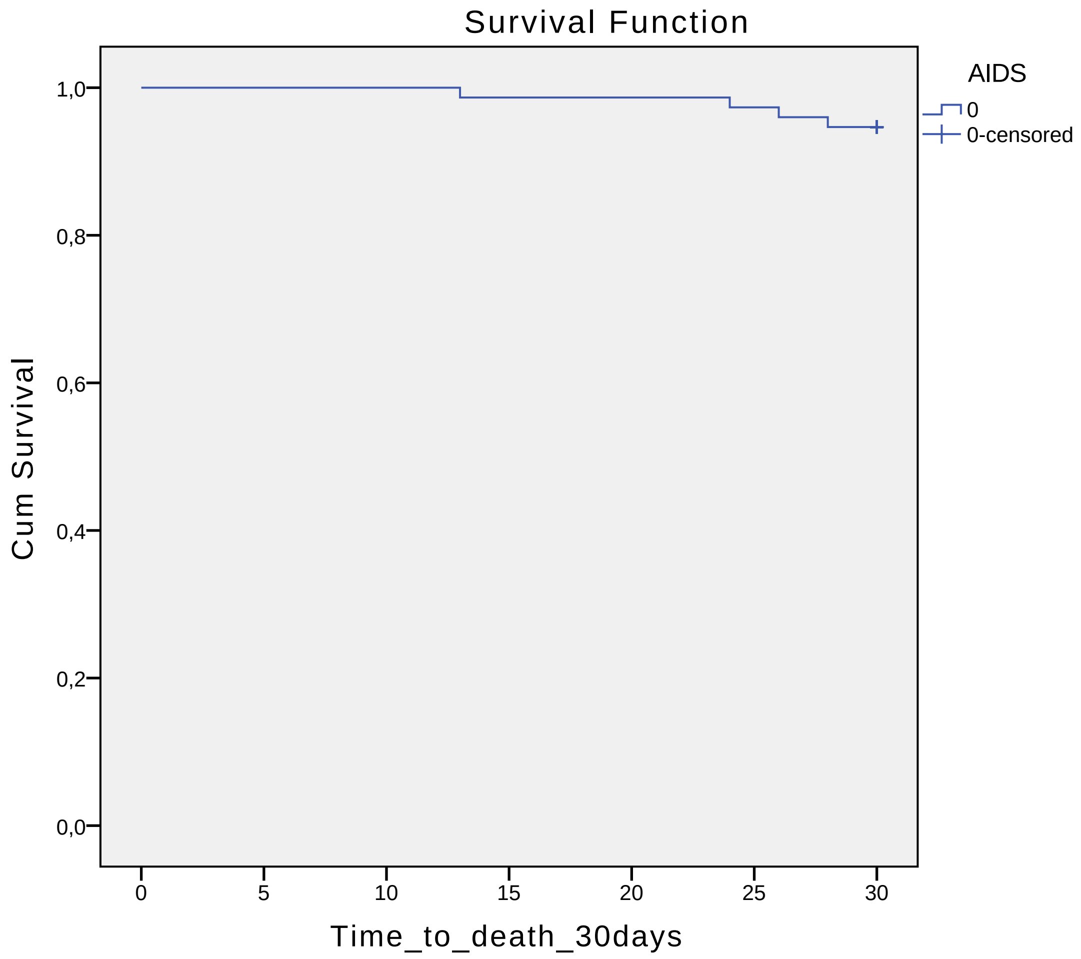

Kaplan-Meier

Warnings

No comparison analysis is performed because the factor variable has only one value for every stratum.

Case Processing Summary

Neutrophil_below_1000	Total N	N of Events	Censored	
			N	Percent
0	75	4	71	94,7%
Overall	75	4	71	94,7%

Means and Medians for Survival Time

Neutrophil_below_1000	Mean[a]				Median		
	Estimate	Std. Error	95% Confidence Interval		Estimate	Std. Error	95% ...
			Lower Bound	Upper Bound			Lower Bound
0	29,613	,244	29,136	30,091	.	.	.
Overall	29,613	,244	29,136	30,091	.	.	.

Means and Medians for Survival Time

Neutrophil_below_1000	Median
	95% ...
	Upper Bound
0	.
Overall	.

a. Estimation is limited to the largest survival time if it is censored.

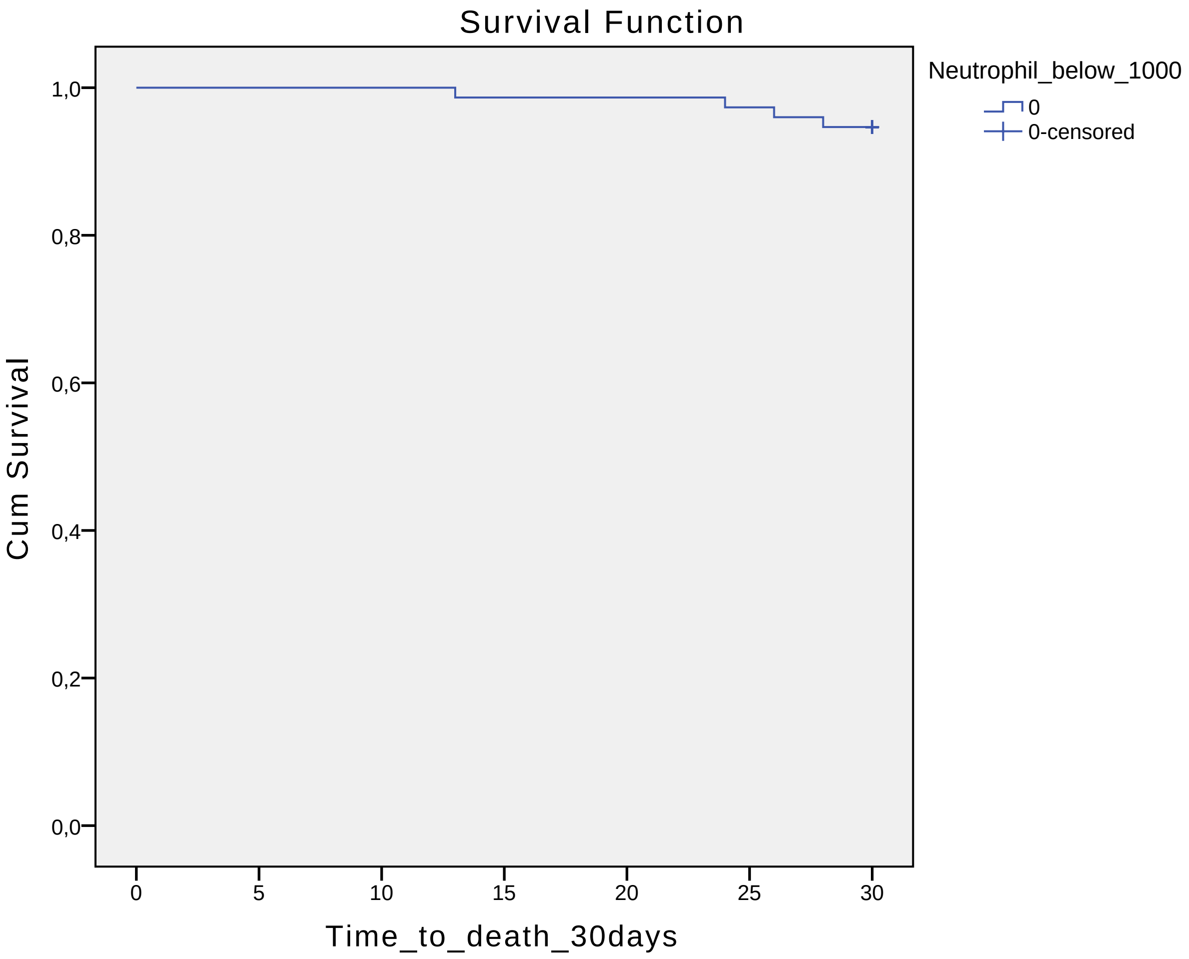

Kaplan-Meier

Warnings

No comparison analysis is performed because the factor variable has only one value for every stratum.

Case Processing Summary

Neutrophil_below_500	Total N	N of Events	Censored	
			N	Percent
0	75	4	71	94,7%
Overall	75	4	71	94,7%

Means and Medians for Survival Time

Neutrophil_below_500	Mean[a]				Median		
	Estimate	Std. Error	95% Confidence Interval		Estimate	Std. Error	95% …
			Lower Bound	Upper Bound			Lower Bound
0	29,613	,244	29,136	30,091	.	.	.
Overall	29,613	,244	29,136	30,091	.	.	.

Means and Medians for Survival Time

Neutrophil_below_500	Median
	95% …
	Upper Bound
0	.
Overall	.

a. Estimation is limited to the largest survival time if it is censored.

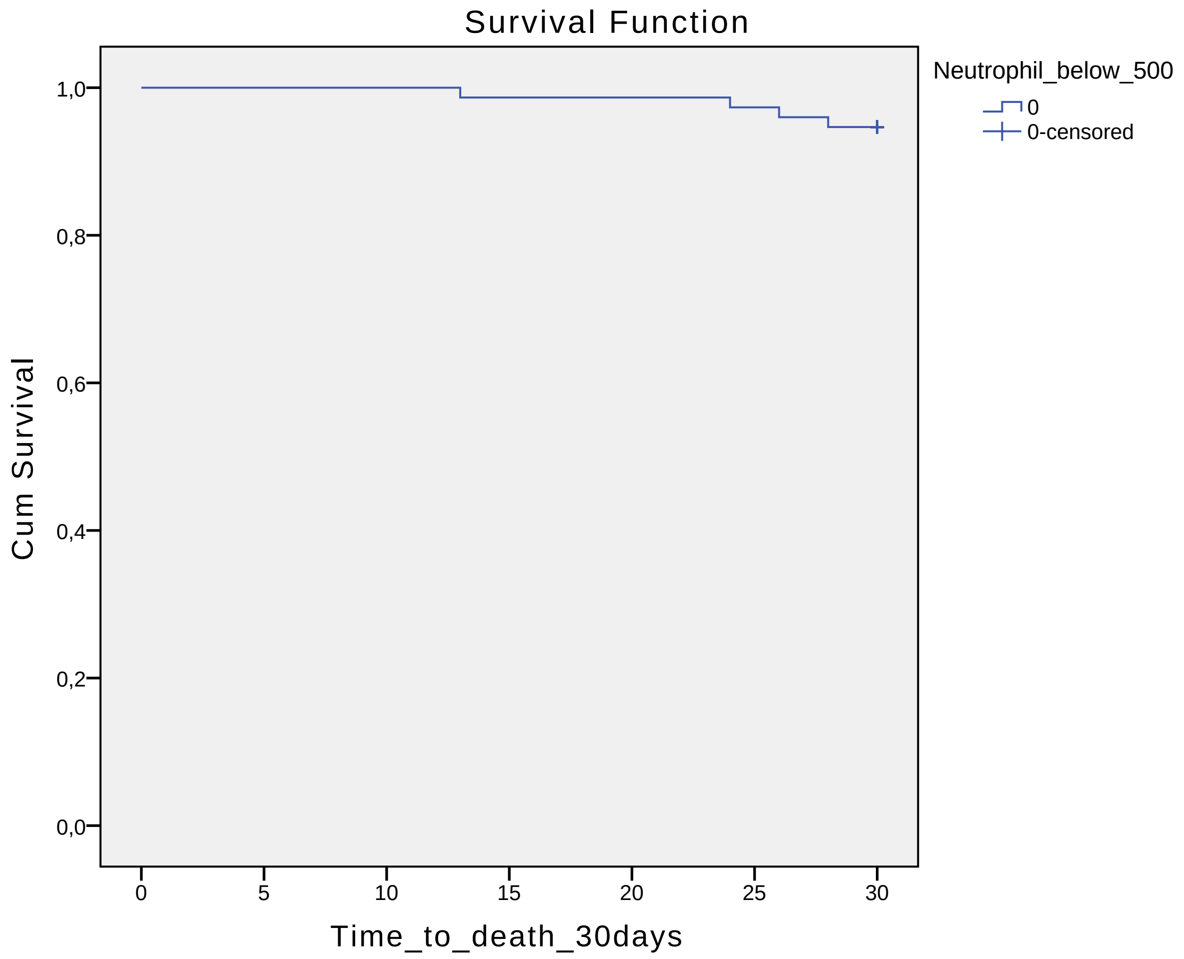

Kaplan-Meier

Warnings

No statistics are computed because all cases are censored.

Case Processing Summary

Corticosteroids	Total N	N of Events	Censored	
			N	Percent
0	71	4	67	94,4%
1	4	0	4	100,0%
Overall	75	4	71	94,7%

Overall Comparisons

	Chi-Square	df	Sig.
Log Rank (Mantel-Cox)	,230	1	,631
Breslow (Generalized Wilcoxon)	,230	1	,631

Test of equality of survival distributions for the different levels of Corticosteroids.

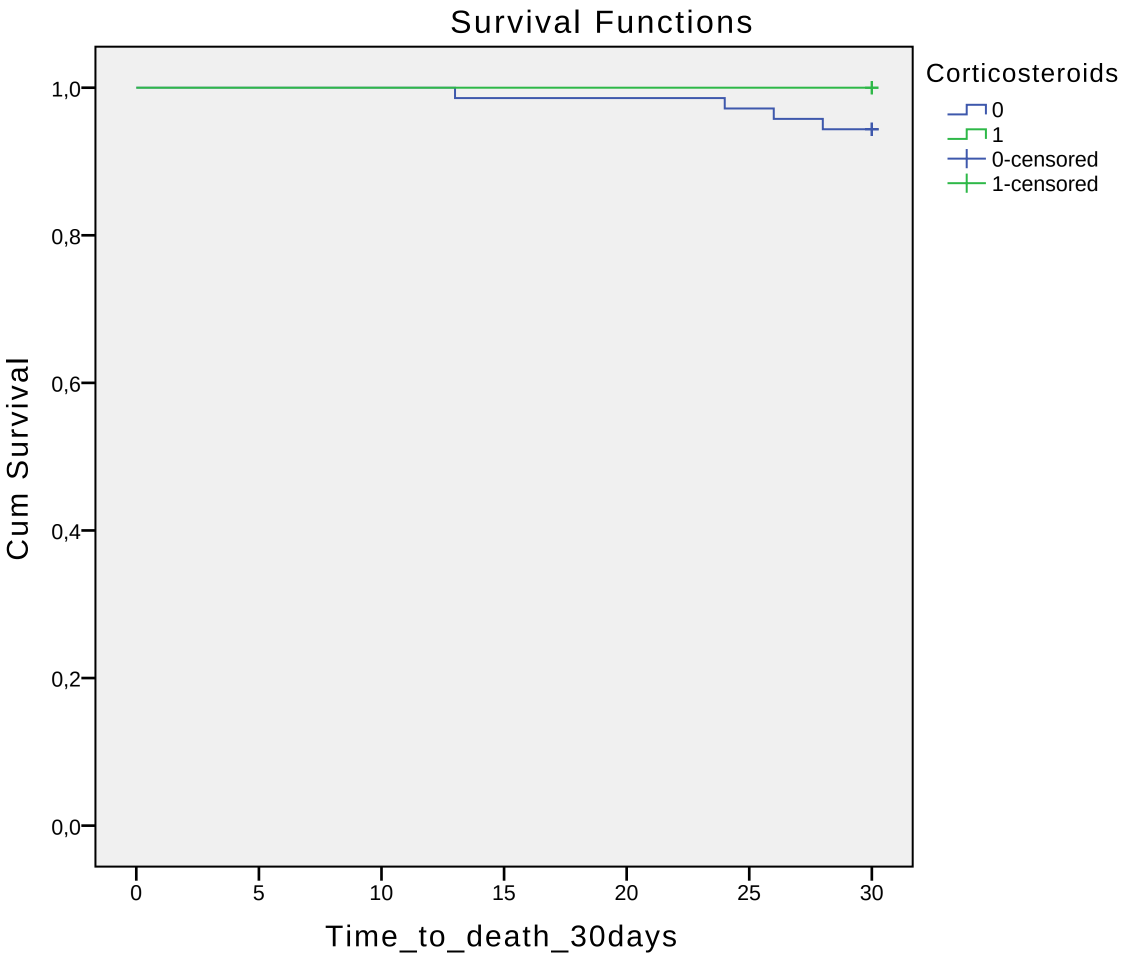

Kaplan-Meier

Warnings

No statistics are computed because all cases are censored.

Case Processing Summary

Transplant	Total N	N of Events	Censored	
			N	Percent
0	73	4	69	94,5%
1	1	0	1	100,0%
Overall	74	4	70	94,6%

Overall Comparisons

	Chi-Square	df	Sig.
Log Rank (Mantel-Cox)	,056	1	,813
Breslow (Generalized Wilcoxon)	,056	1	,813

Test of equality of survival distributions for the different levels of Transplant.

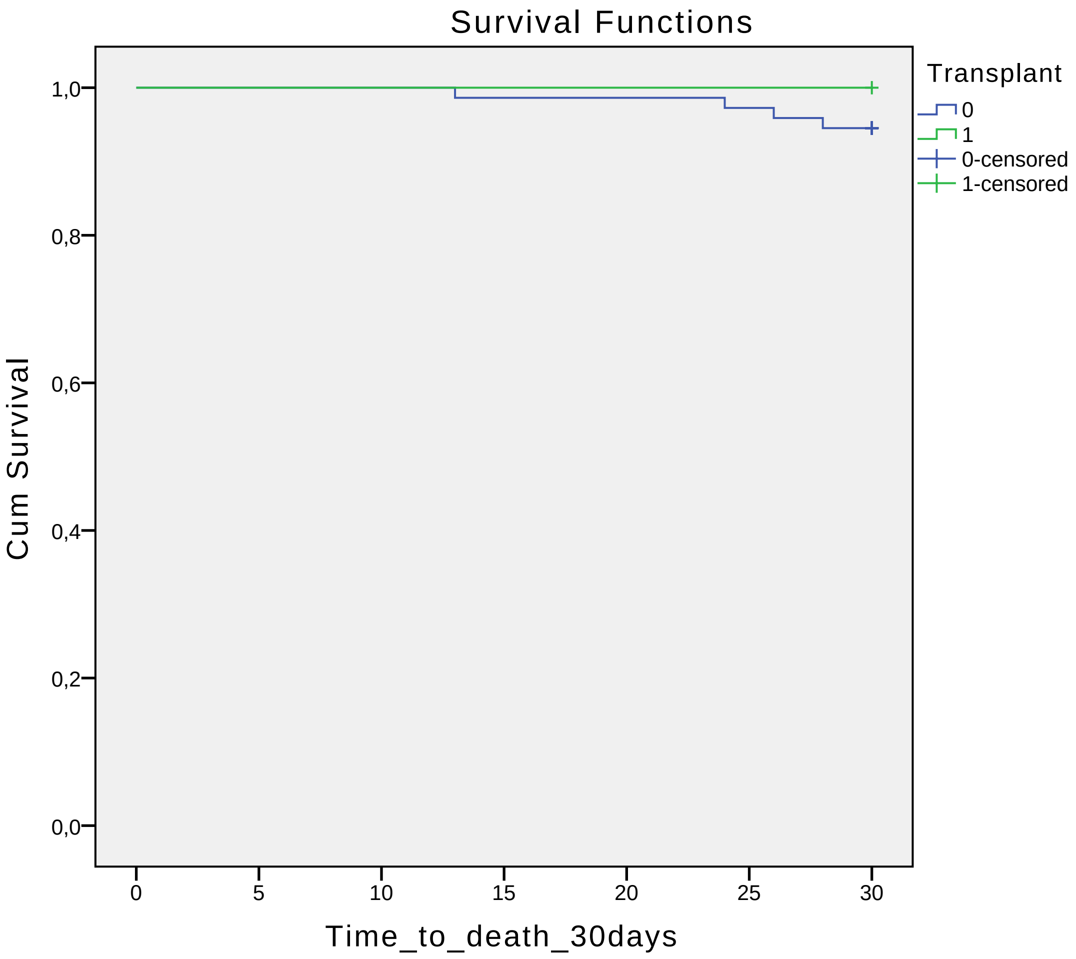

Kaplan-Meier

Warnings

No statistics are computed because all cases are censored.

Case Processing Summary

Immunosuppression	Total N	N of Events	Censored	
			N	Percent
0	71	4	67	94,4%
1	4	0	4	100,0%
Overall	75	4	71	94,7%

Overall Comparisons

	Chi-Square	df	Sig.
Log Rank (Mantel-Cox)	,230	1	,631
Breslow (Generalized Wilcoxon)	,230	1	,631

Test of equality of survival distributions for the different levels of Immunosuppression.

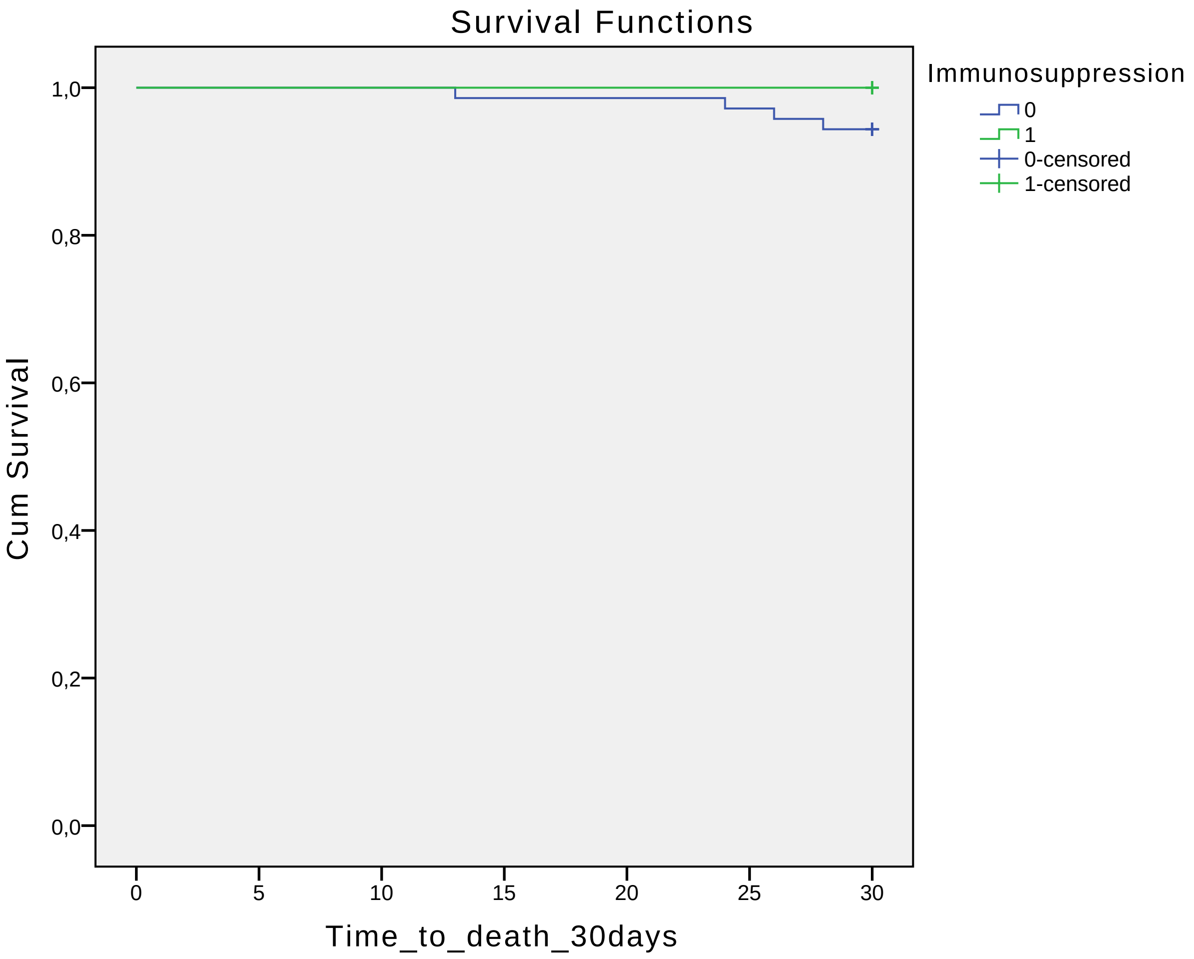

Kaplan-Meier

Warnings

No statistics are computed because all cases are censored.

Case Processing Summary

Leukocytosis	Total N	N of Events	Censored	
			N	Percent
0	51	4	47	92,2%
1	20	0	20	100,0%
Overall	71	4	67	94,4%

Overall Comparisons

	Chi-Square	df	Sig.
Log Rank (Mantel-Cox)	1,617	1	,204
Breslow (Generalized Wilcoxon)	1,616	1	,204

Test of equality of survival distributions for the different levels of Leukocytosis.

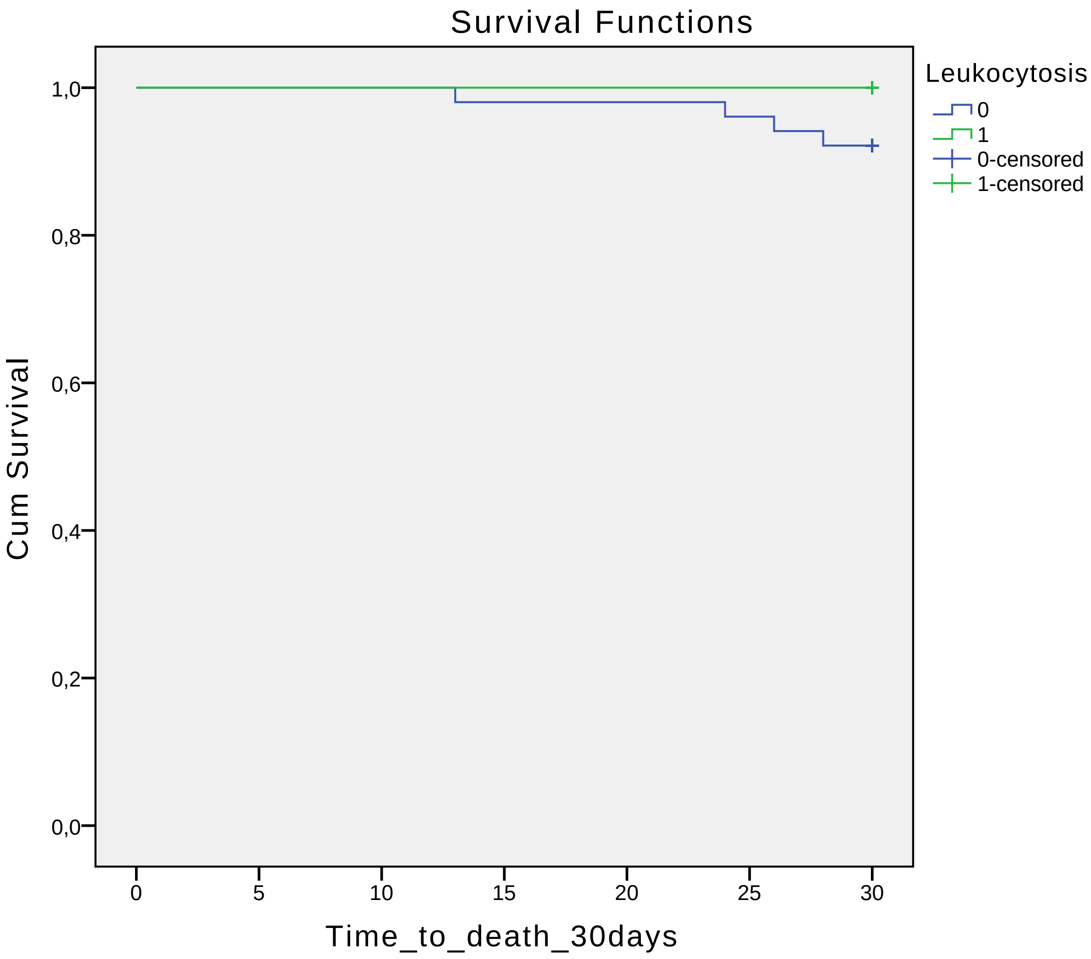

Kaplan-Meier

Case Processing Summary

Leukopenia	Total N	N of Events	Censored	
			N	Percent
0	69	3	66	95,7%
1	2	1	1	50,0%
Overall	71	4	67	94,4%

Means and Medians for Survival Time

Leukopenia	Mean[a]				Median			
	Estimate	Std. Error	95% Confidence Interval		Estimate	Std. Error	95% Confidence Interval	
			Lower Bound	Upper Bound			Lower Bound	Upper Bound
0	29,609	,263	29,092	30,125	.	.	.	.
1	29,000	,707	27,614	30,386	28,000	.	.	.
Overall	29,592	,257	29,088	30,095	.	.	.	.

a. Estimation is limited to the largest survival time if it is censored.

Overall Comparisons

	Chi-Square	df	Sig.
Log Rank (Mantel-Cox)	7,002	1	,008
Breslow (Generalized Wilcoxon)	6,667	1	,010

Test of equality of survival distributions for the different levels of Leukopenia.

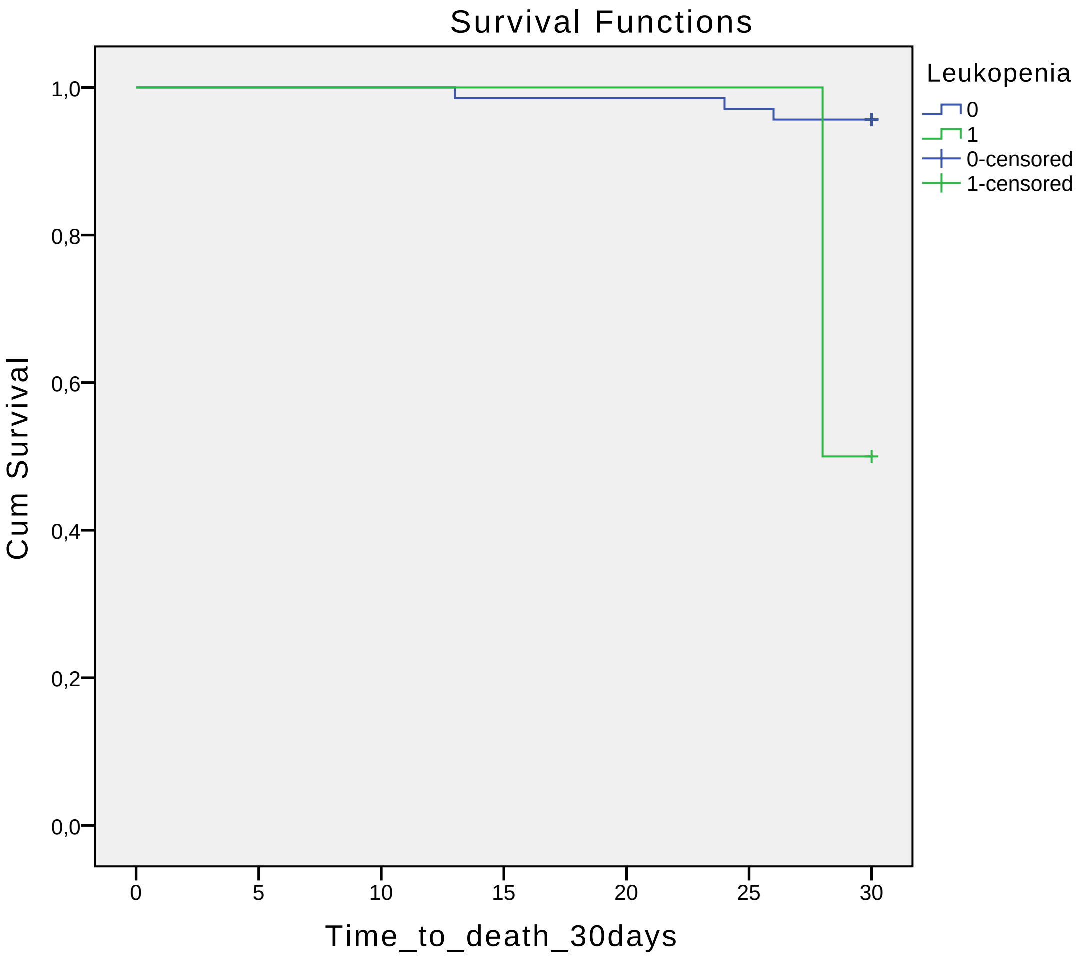

Kaplan-Meier

Case Processing Summary

Thrombopenia	Total N	N of Events	Censored	
			N	Percent
0	65	3	62	95,4%
1	6	1	5	83,3%
Overall	71	4	67	94,4%

Means and Medians for Survival Time

Thrombopenia	Mean[a]				Median		
	Estimate	Std. Error	95% Confidence Interval		Estimate	Std. Error	95% …
			Lower Bound	Upper Bound			Lower Bound
0	29,585	,279	29,037	30,132	.	.	.
1	29,667	,304	29,070	30,263	.	.	.
Overall	29,592	,257	29,088	30,095	.	.	.

Means and Medians for Survival Time

Thrombopenia	Median
	95% …
	Upper Bound
0	.
1	.
Overall	.

a. Estimation is limited to the largest survival time if it is censored.

Overall Comparisons

	Chi-Square	df	Sig.
Log Rank (Mantel-Cox)	1,358	1	,244
Breslow (Generalized Wilcoxon)	1,270	1	,260

Test of equality of survival distributions for the different levels of Thrombopenia.

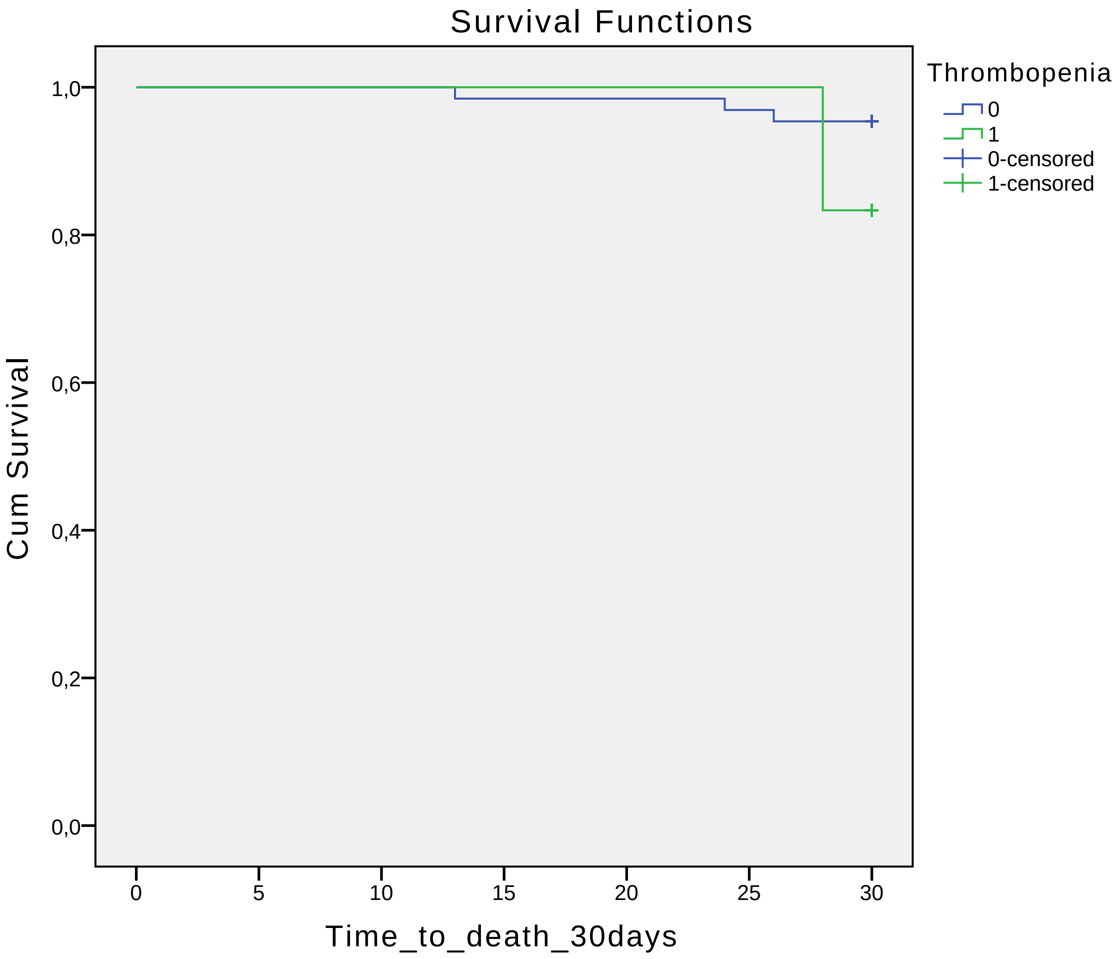

Kaplan-Meier

Case Processing Summary

Renal_failure	Total N	N of Events	Censored	
			N	Percent
0	40	2	38	95,0%
1	31	2	29	93,5%
Overall	71	4	67	94,4%

Means and Medians for Survival Time

Renal_failure	Mean[a]				Median		
			95% Confidence Interval				95% ...
	Estimate	Std. Error	Lower Bound	Upper Bound	Estimate	Std. Error	Lower Bound
0	29,750	,176	29,405	30,095	.	.	.
1	29,387	,541	28,327	30,448	.	.	.
Overall	29,592	,257	29,088	30,095	.	.	.

Means and Medians for Survival Time

Renal_failure	Median
	95% ...
	Upper Bound
0	.
1	.
Overall	.

a. Estimation is limited to the largest survival time if it is censored.

Overall Comparisons

	Chi-Square	df	Sig.
Log Rank (Mantel-Cox)	,068	1	,794
Breslow (Generalized Wilcoxon)	,068	1	,794

Test of equality of survival distributions for the different levels of Renal_failure.

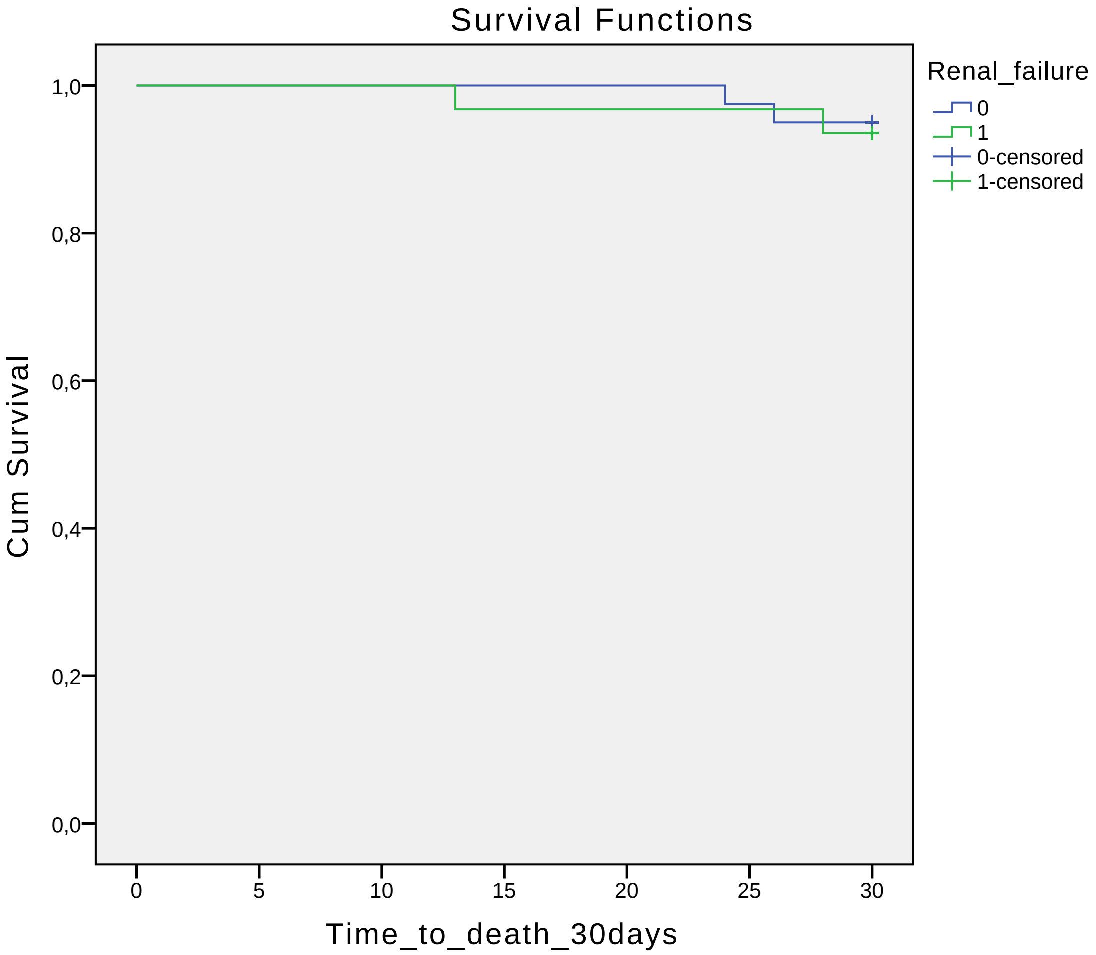

Kaplan-Meier

Case Processing Summary

Recurrent_UTI	Total N	N of Events	Censored	
			N	Percent
0	60	3	57	95,0%
1	14	1	13	92,9%
Overall	74	4	70	94,6%

Means and Medians for Survival Time

Recurrent_UTI	Mean[a]				Median		
			95% Confidence Interval				95% …
	Estimate	Std. Error	Lower Bound	Upper Bound	Estimate	Std. Error	Lower Bound
0	29,583	,297	29,000	30,166	.	.	.
1	29,714	,275	29,175	30,254	.	.	.
Overall	29,608	,247	29,124	30,092	.	.	.

Means and Medians for Survival Time

Recurrent_UTI	Median
	95% …
	Upper Bound
0	.
1	.
Overall	.

a. Estimation is limited to the largest survival time if it is censored.

Overall Comparisons

	Chi-Square	df	Sig.
Log Rank (Mantel-Cox)	,095	1	,758
Breslow (Generalized Wilcoxon)	,089	1	,765

Test of equality of survival distributions for the different levels of Recurrent_UTI.

Survival Functions

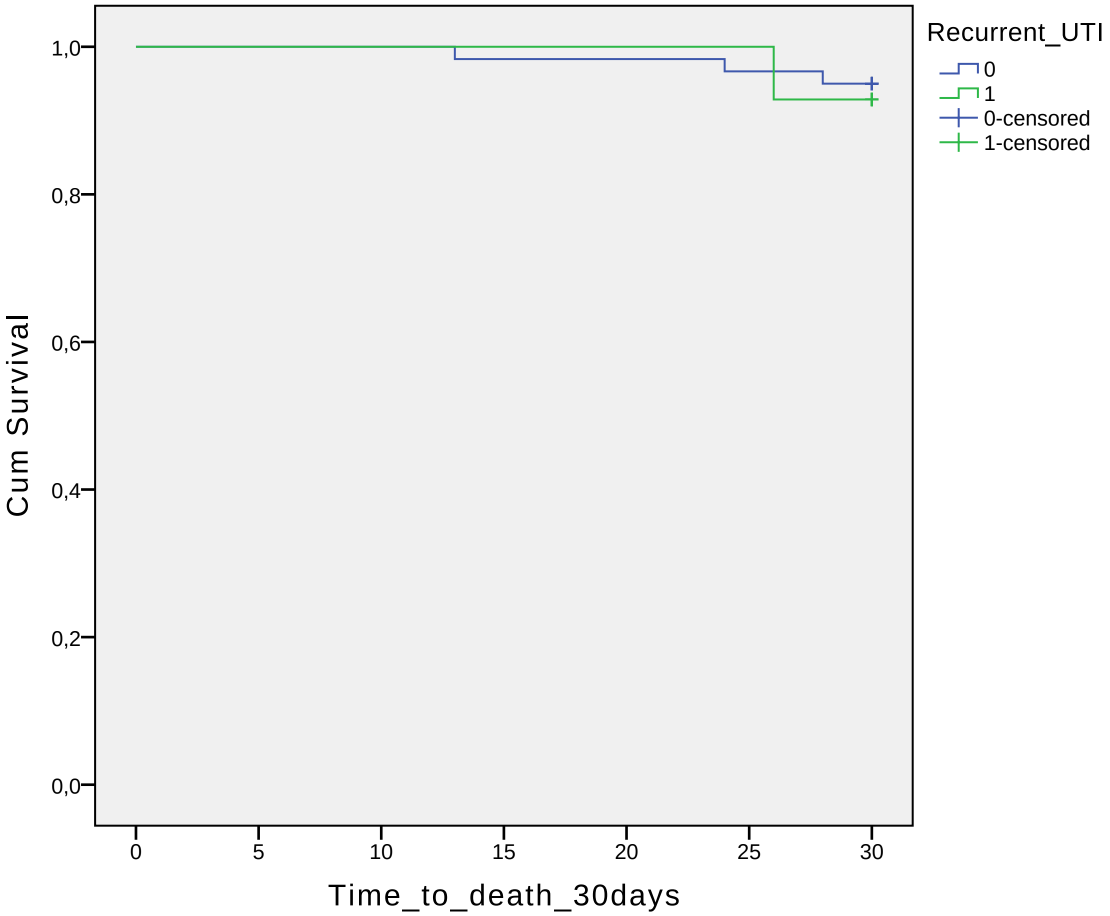

Kaplan-Meier

Case Processing Summary

Community	Total N	N of Events	Censored	
			N	Percent
0	43	3	40	93,0%
1	32	1	31	96,9%
Overall	75	4	71	94,7%

Means and Medians for Survival Time

Community	Mean[a]				Median			
	Estimate	Std. Error	95% Confidence Interval		Estimate	Std. Error	95% Confidence Interval	
			Lower Bound	Upper Bound			Lower Bound	Upper Bound
0	29,419	,412	28,610	30,227	.	.	.	.
1	29,875	,123	29,634	30,116	.	.	.	.
Overall	29,613	,244	29,136	30,091	.	.	.	.

a. Estimation is limited to the largest survival time if it is censored.

Overall Comparisons

	Chi-Square	df	Sig.
Log Rank (Mantel-Cox)	,540	1	,462
Breslow (Generalized Wilcoxon)	,550	1	,458

Test of equality of survival distributions for the different levels of Community.

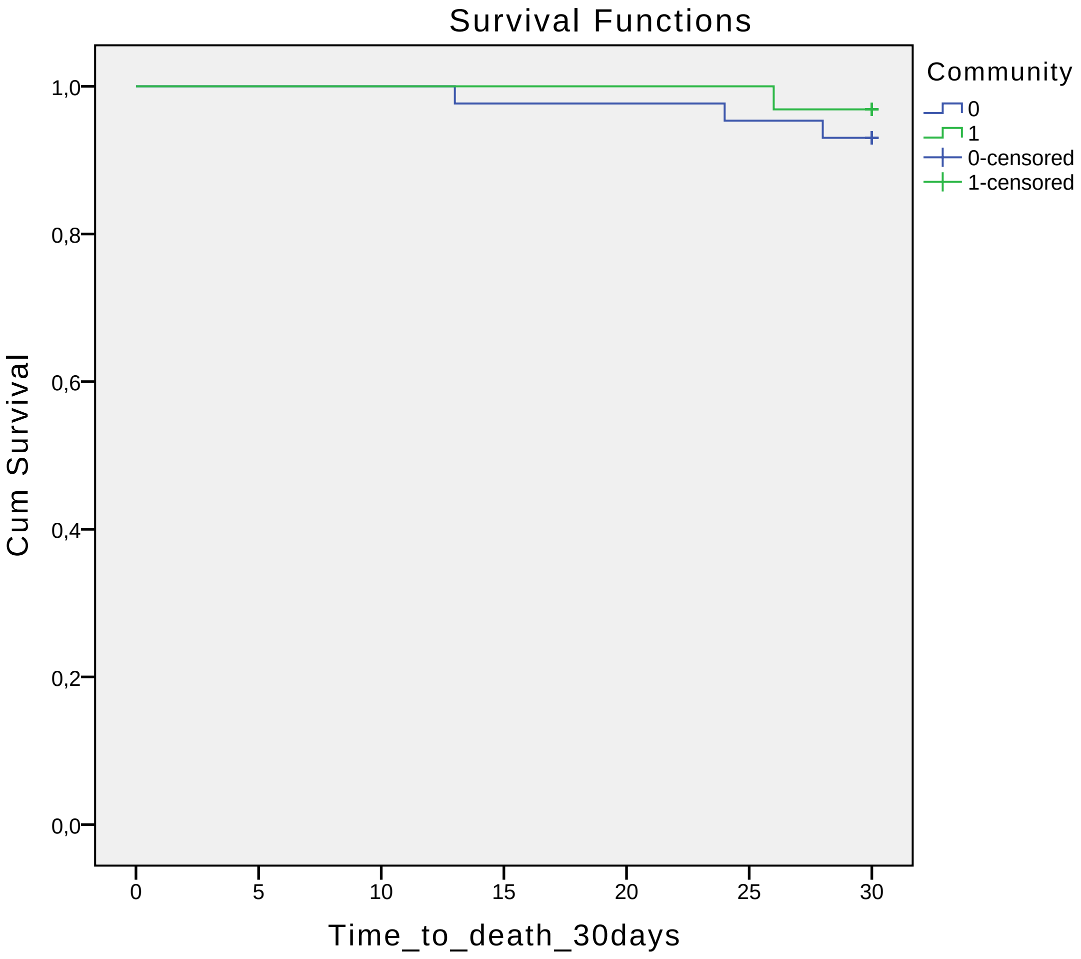

Kaplan-Meier

Case Processing Summary

Healthcare_associated	Total N	N of Events	Censored N	Censored Percent
0	41	2	39	95,1%
1	34	2	32	94,1%
Overall	75	4	71	94,7%

Means and Medians for Survival Time

Healthcare_associated	Mean[a]				Median		
	Estimate	Std. Error	95% Confidence Interval		Estimate	Std. Error	95% …
			Lower Bound	Upper Bound			Lower Bound
0	29,756	,172	29,420	30,093	.	.	.
1	29,441	,494	28,472	30,410	.	.	.
Overall	29,613	,244	29,136	30,091	.	.	.

Means and Medians for Survival Time

Healthcare_associated	Median
	95% …
	Upper Bound
0	.
1	.
Overall	.

a. Estimation is limited to the largest survival time if it is censored.

Overall Comparisons

	Chi-Square	df	Sig.
Log Rank (Mantel-Cox)	,037	1	,848
Breslow (Generalized Wilcoxon)	,037	1	,848

Test of equality of survival distributions for the different levels of Healthcare_associated.

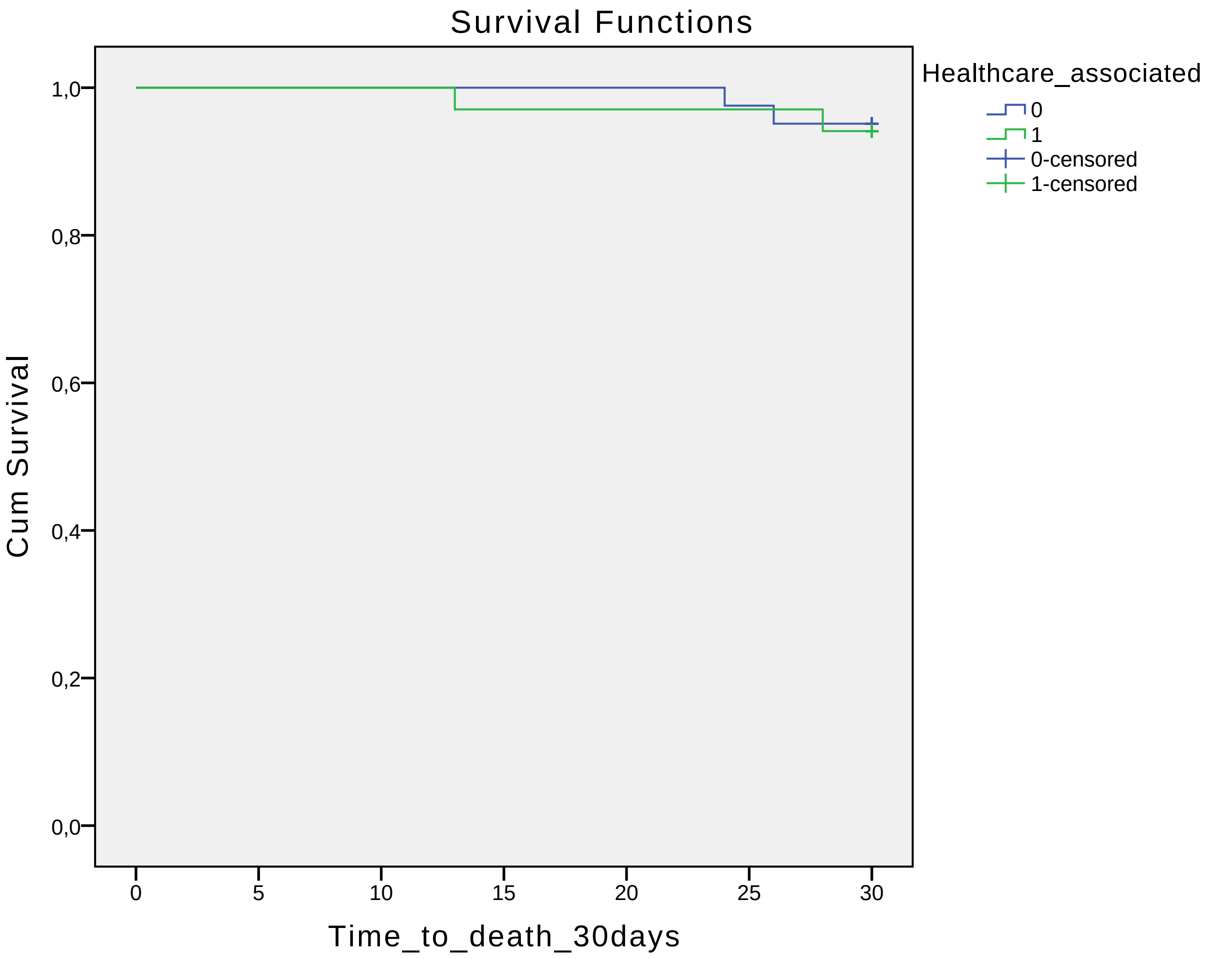

Kaplan-Meier

Case Processing Summary

Nosocomial	Total N	N of Events	Censored	
			N	Percent
0	66	3	63	95,5%
1	9	1	8	88,9%
Overall	75	4	71	94,7%

Means and Medians for Survival Time

Nosocomial	Mean[a]				Median			
	Estimate	Std. Error	95% Confidence Interval		Estimate	Std. Error	95% Confidence Interval	
			Lower Bound	Upper Bound			Lower Bound	Upper Bound
0	29,652	,263	29,136	30,167	.	.	.	.
1	29,333	,629	28,101	30,565	.	.	.	.
Overall	29,613	,244	29,136	30,091	.	.	.	.

a. Estimation is limited to the largest survival time if it is censored.

Overall Comparisons

	Chi-Square	df	Sig.
Log Rank (Mantel-Cox)	,707	1	,400
Breslow (Generalized Wilcoxon)	,724	1	,395

Test of equality of survival distributions for the different levels of Nosocomial.

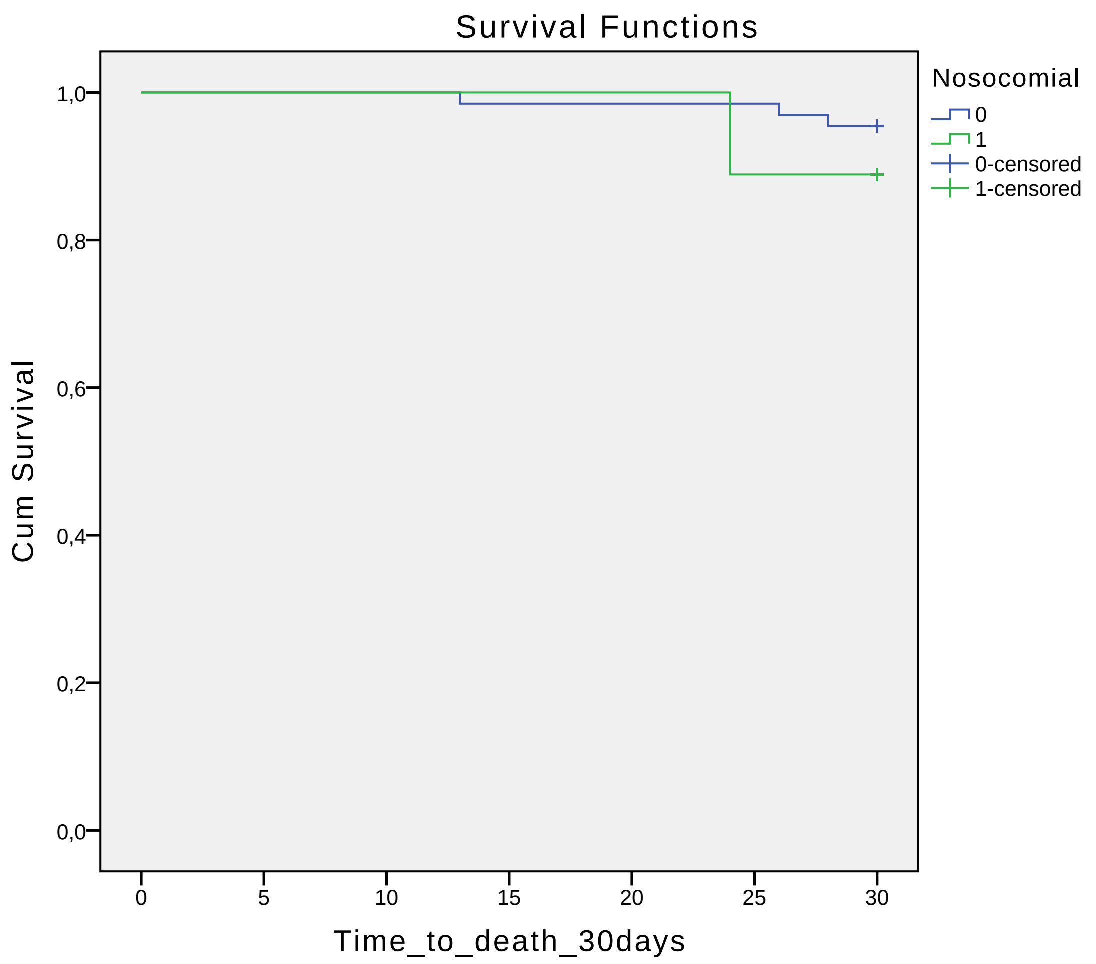

Kaplan-Meier

Case Processing Summary

Nursing_home	Total N	N of Events	Censored	
			N	Percent
0	68	3	65	95,6%
1	7	1	6	85,7%
Overall	75	4	71	94,7%

Means and Medians for Survival Time

Nursing_home	Mean[a]				Median		
			95% Confidence Interval				95% ...
	Estimate	Std. Error	Lower Bound	Upper Bound	Estimate	Std. Error	Lower Bound
0	29,603	,267	29,079	30,127	.	.	.
1	29,714	,265	29,196	30,233	.	.	.
Overall	29,613	,244	29,136	30,091	.	.	.

Means and Medians for Survival Time

Nursing_home	Median
	95% ...
	Upper Bound
0	.
1	.
Overall	.

a. Estimation is limited to the largest survival time if it is censored.

Overall Comparisons

	Chi-Square	df	Sig.
Log Rank (Mantel-Cox)	1,111	1	,292
Breslow (Generalized Wilcoxon)	1,040	1	,308

Test of equality of survival distributions for the different levels of Nursing_home.

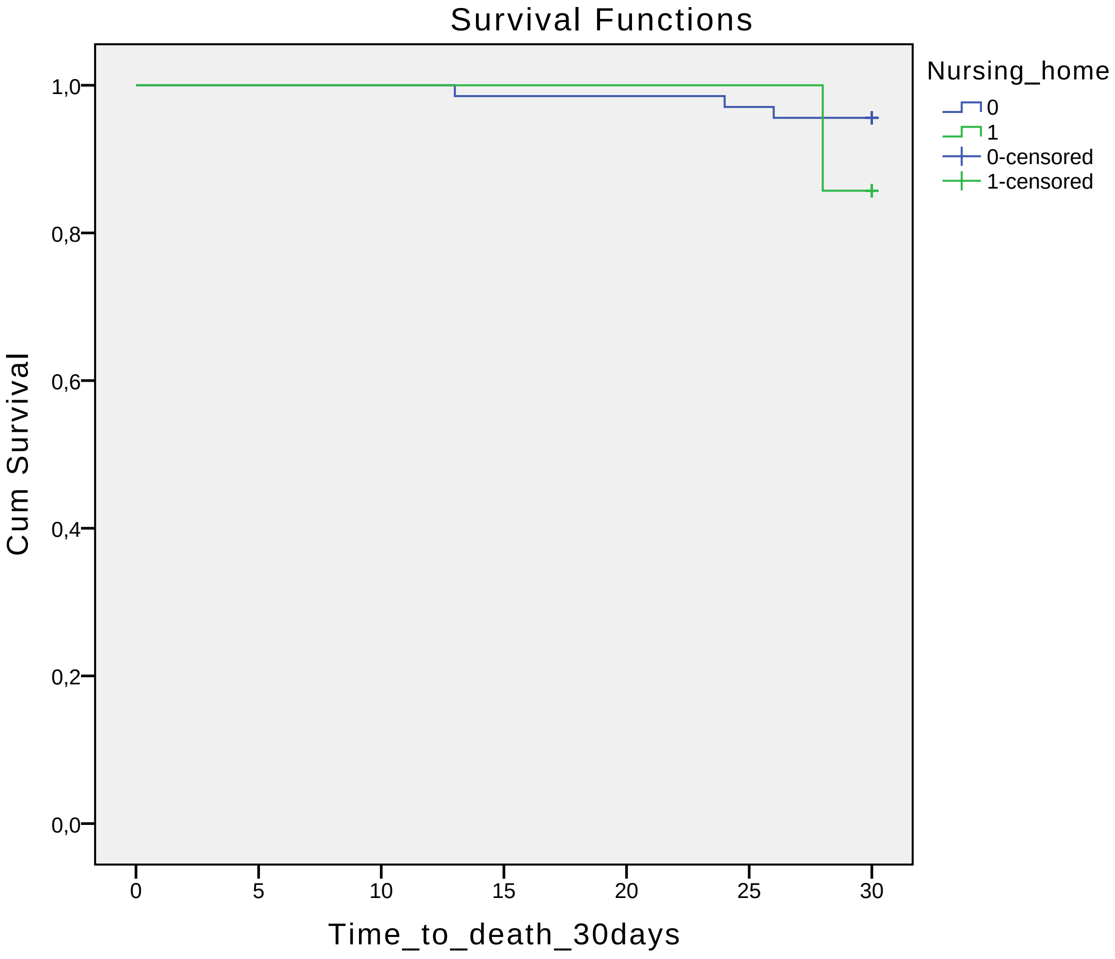

Kaplan-Meier

Case Processing Summary

Previous_ESBL_UTI	Total N	N of Events	Censored	
			N	Percent
0	61	3	58	95,1%
1	13	1	12	92,3%
Overall	74	4	70	94,6%

Means and Medians for Survival Time

Previous_ESBL_UTI	Mean[a]				Median		
			95% Confidence Interval				95% ...
	Estimate	Std. Error	Lower Bound	Upper Bound	Estimate	Std. Error	Lower Bound
0	29,590	,293	29,017	30,164	.	.	.
1	29,692	,296	29,113	30,272	.	.	.
Overall	29,608	,247	29,124	30,092	.	.	.

Means and Medians for Survival Time

Previous_ESBL_UTI	Median 95% ... Upper Bound
0	.
1	.
Overall	.

a. Estimation is limited to the largest survival time if it is censored.

Overall Comparisons

	Chi-Square	df	Sig.
Log Rank (Mantel-Cox)	,152	1	,697
Breslow (Generalized Wilcoxon)	,145	1	,704

Test of equality of survival distributions for the different levels of Previous_ESBL_UTI.

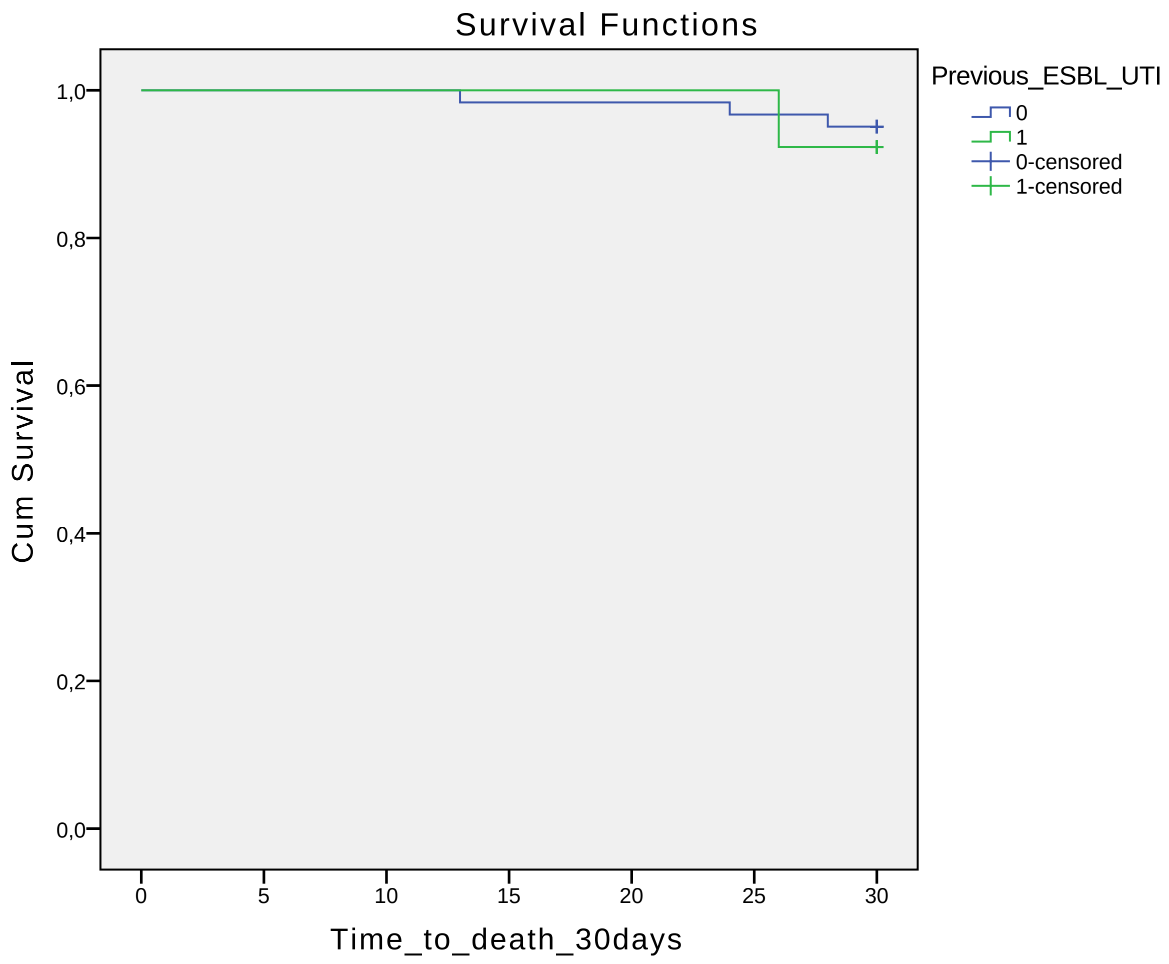

Kaplan-Meier

Case Processing Summary

Carbapenem	Total N	N of Events	Censored N	Censored Percent
,00	39	1	38	97,4%
1,00	36	3	33	91,7%
Overall	75	4	71	94,7%

Means and Medians for Survival Time

Carbapenem	Mean[a]				Median		
			95% Confidence Interval				95% ...
	Estimate	Std. Error	Lower Bound	Upper Bound	Estimate	Std. Error	Lower Bound
,00	29,949	,051	29,850	30,048	.	.	.
1,00	29,250	,497	28,275	30,225	.	.	.
Overall	29,613	,244	29,136	30,091	.	.	.

Means and Medians for Survival Time

Carbapenem	Median
	95% ...
	Upper Bound
,00	.
1,00	.
Overall	.

a. Estimation is limited to the largest survival time if it is censored.

Overall Comparisons

	Chi-Square	df	Sig.
Log Rank (Mantel-Cox)	1,266	1	,260
Breslow (Generalized Wilcoxon)	1,311	1	,252

Test of equality of survival distributions for the different levels of Carbapenem.

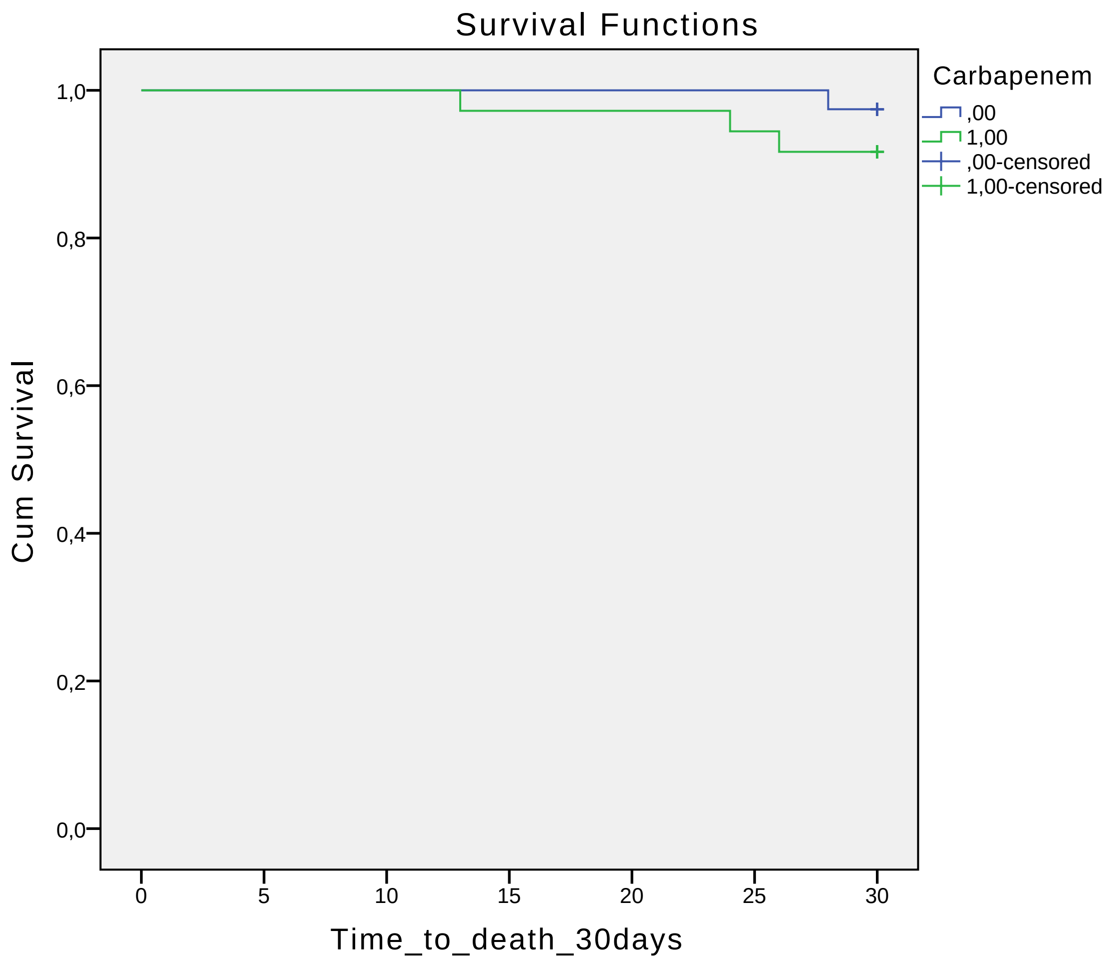

Kaplan-Meier

Warnings

No statistics are computed because all cases are censored.

Case Processing Summary

Betalactam_betalactamase_inhibitor	Total N	N of Events	Censored	
			N	Percent
,00	70	4	66	94,3%
1,00	5	0	5	100,0%
Overall	75	4	71	94,7%

Overall Comparisons

	Chi-Square	df	Sig.
Log Rank (Mantel-Cox)	,292	1	,589
Breslow (Generalized Wilcoxon)	,292	1	,589

Test of equality of survival distributions for the different levels of Betalactam_betalactamase_inhibitor.

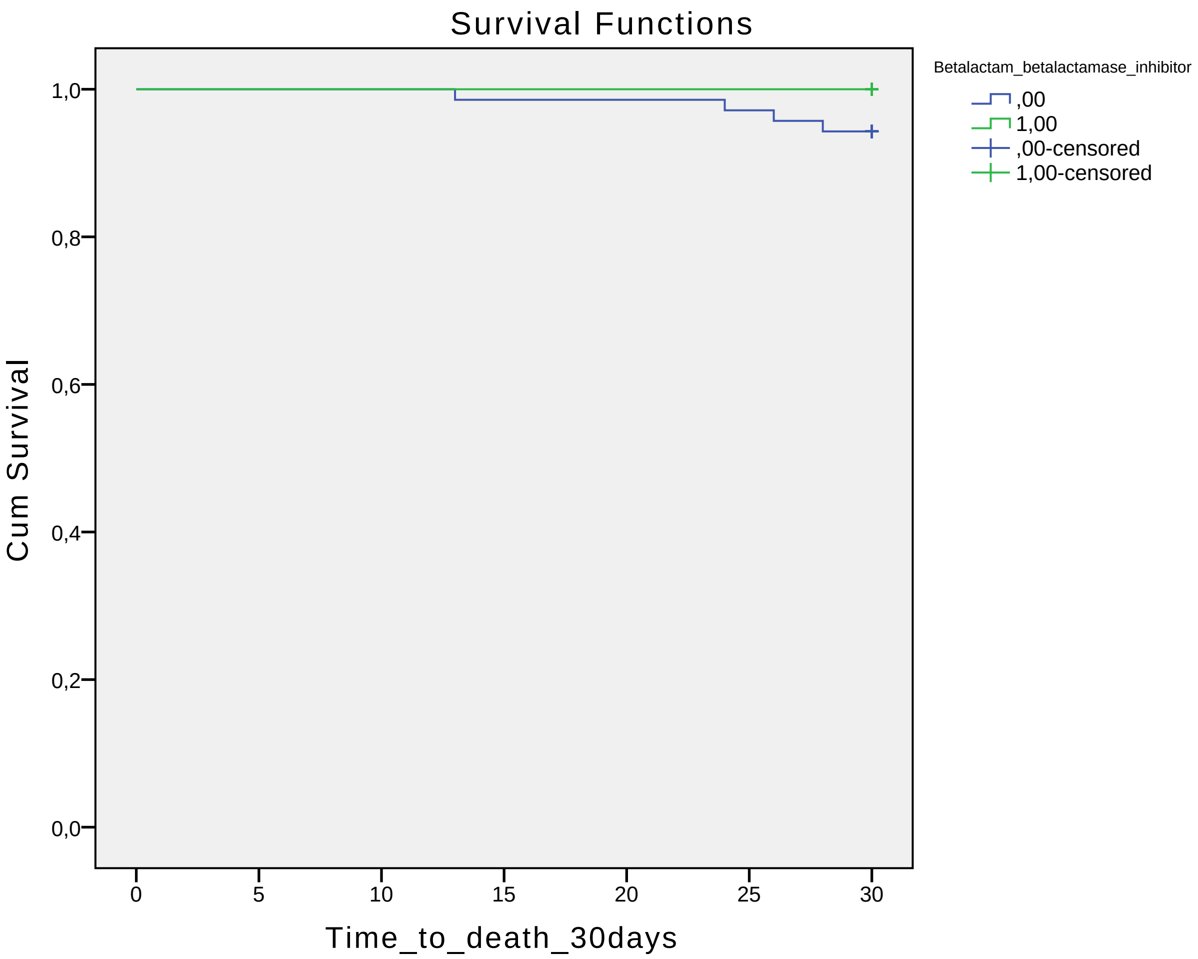

Kaplan-Meier

Warnings

No statistics are computed because all cases are censored.

Case Processing Summary

Quinolone	Total N	N of Events	Censored	
			N	Percent
,00	63	4	59	93,7%
1,00	12	0	12	100,0%
Overall	75	4	71	94,7%

Overall Comparisons

	Chi-Square	df	Sig.
Log Rank (Mantel-Cox)	,781	1	,377
Breslow (Generalized Wilcoxon)	,780	1	,377

Test of equality of survival distributions for the different levels of Quinolone.

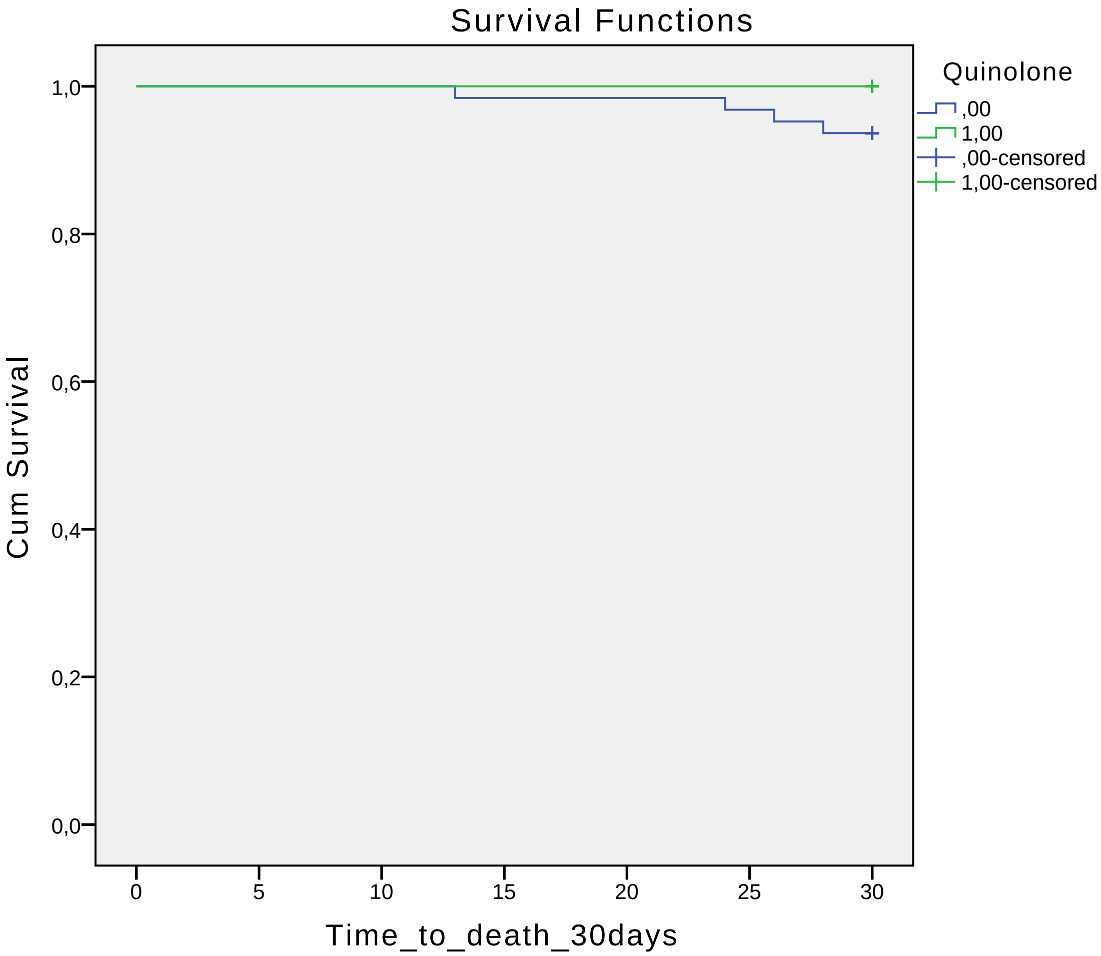

Kaplan-Meier

Warnings

No statistics are computed because all cases are censored.

Case Processing Summary

Trimethoprim_sulfamethoxazole	Total N	N of Events	Censored	
			N	Percent
,00	68	4	64	94,1%
1,00	7	0	7	100,0%
Overall	75	4	71	94,7%

Overall Comparisons

	Chi-Square	df	Sig.
Log Rank (Mantel-Cox)	,421	1	,516
Breslow (Generalized Wilcoxon)	,421	1	,516

Test of equality of survival distributions for the different levels of Trimethoprim_sulfamethoxazole.

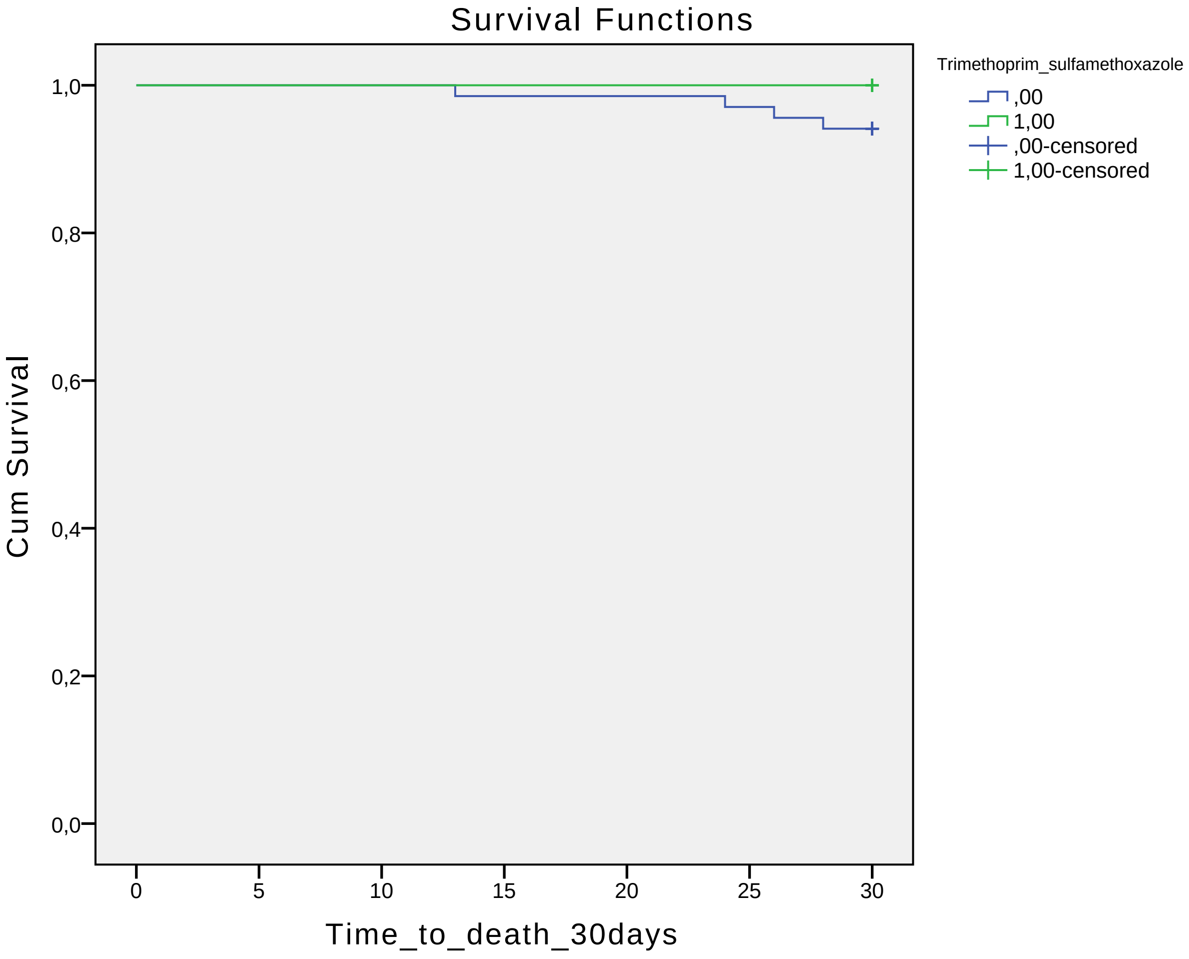

Kaplan-Meier

Case Processing Summary

Phosfomycin	Total N	N of Events	Censored	
			N	Percent
,00	62	3	59	95,2%
1,00	13	1	12	92,3%
Overall	75	4	71	94,7%

Means and Medians for Survival Time

Phosfomycin	Mean[a]				Median		
			95% Confidence Interval				95% ...
	Estimate	Std. Error	Lower Bound	Upper Bound	Estimate	Std. Error	Lower Bound
,00	29,565	,293	28,991	30,138	.	.	.
1,00	29,846	,148	29,556	30,136	.	.	.
Overall	29,613	,244	29,136	30,091	.	.	.

Means and Medians for Survival Time

Phosfomycin	Median 95% ... Upper Bound
,00	.
1,00	.
Overall	.

a. Estimation is limited to the largest survival time if it is censored.

Overall Comparisons

	Chi-Square	df	Sig.
Log Rank (Mantel-Cox)	,147	1	,702
Breslow (Generalized Wilcoxon)	,127	1	,721

Test of equality of survival distributions for the different levels of Phosfomycin.

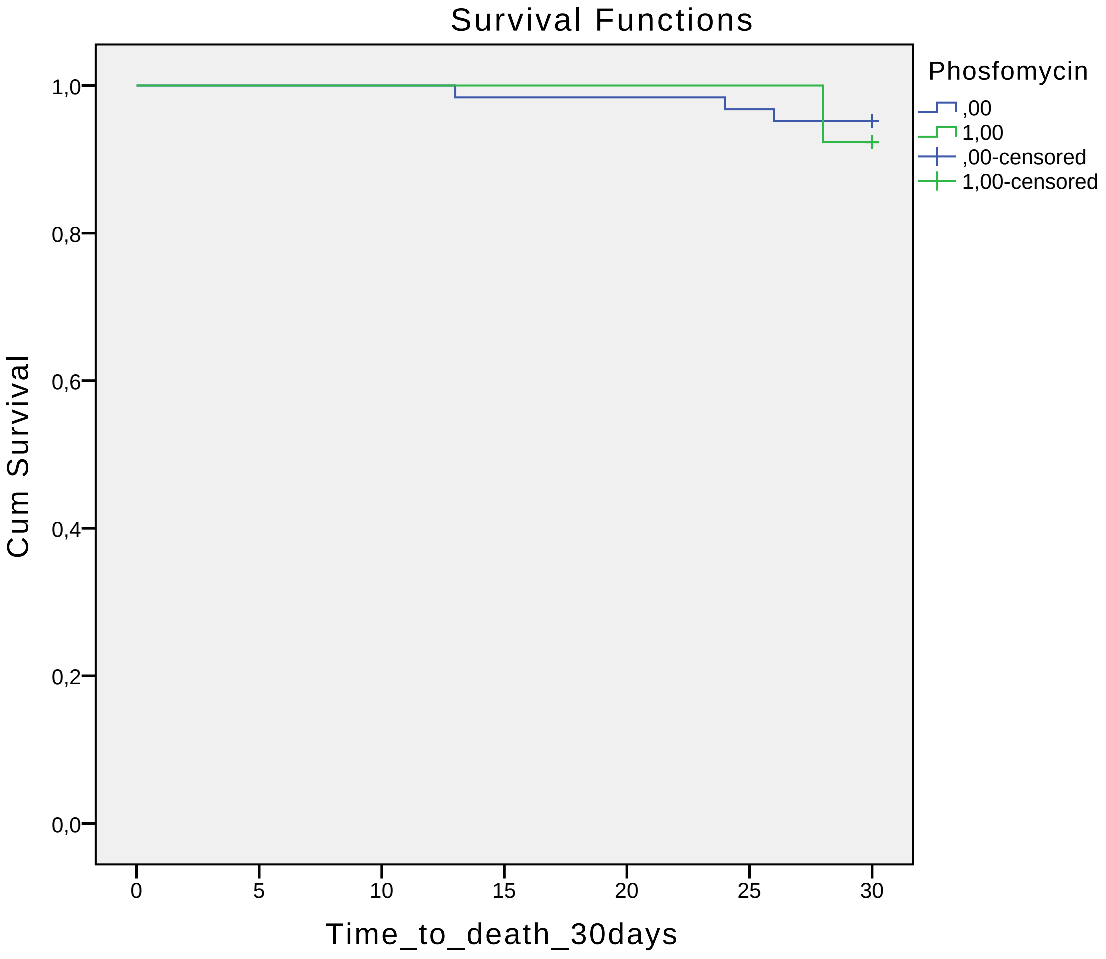

Kaplan-Meier

Warnings

No statistics are computed because all cases are censored.

Case Processing Summary

Furantoin	Total N	N of Events	Censored	
			N	Percent
,00	73	4	69	94,5%
1,00	2	0	2	100,0%
Overall	75	4	71	94,7%

Overall Comparisons

	Chi-Square	df	Sig.
Log Rank (Mantel-Cox)	,112	1	,738
Breslow (Generalized Wilcoxon)	,112	1	,738

Test of equality of survival distributions for the different levels of Furantoin.

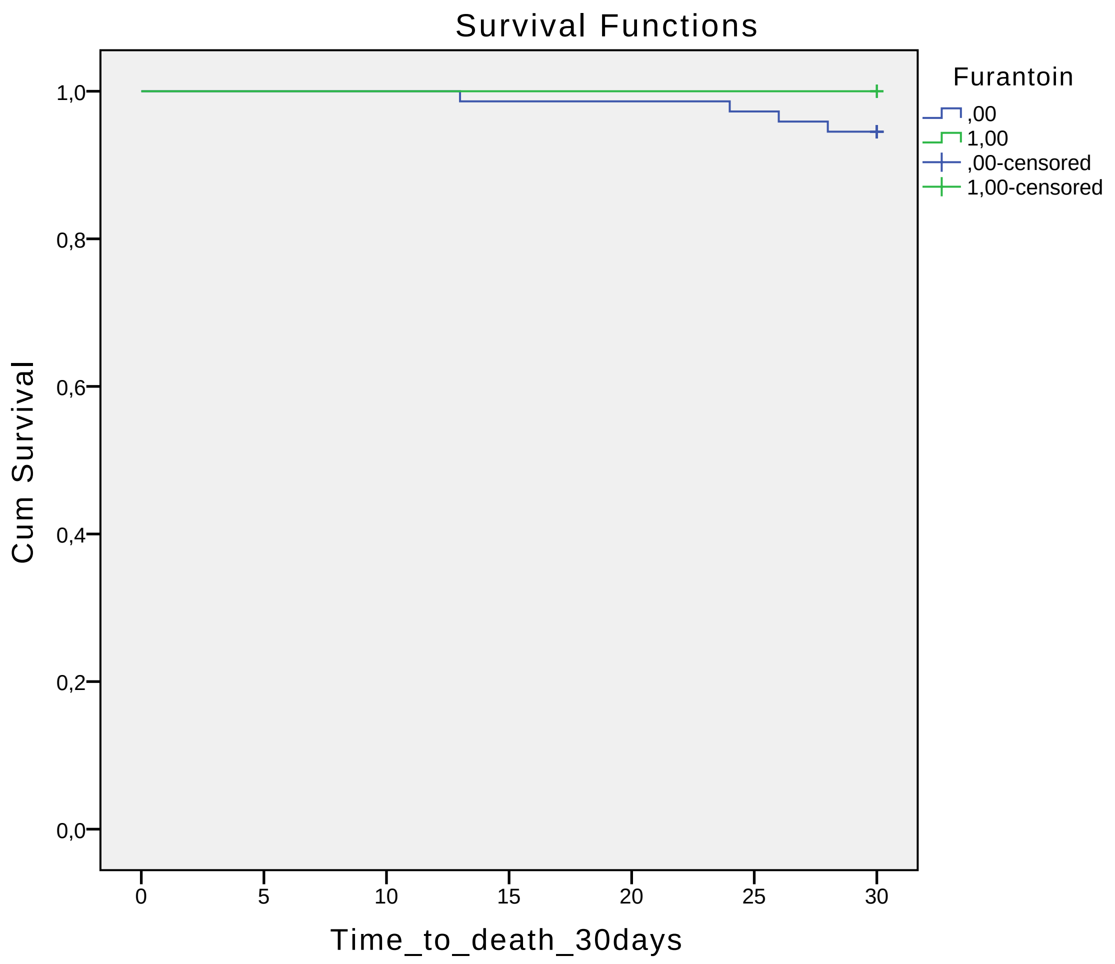

Kaplan-Meier

Warnings

No statistics are computed because all cases are censored.

Case Processing Summary

Aminoglicosyde	Total N	N of Events	Censored	
			N	Percent
,00	74	4	70	94,6%
1,00	1	0	1	100,0%
Overall	75	4	71	94,7%

Overall Comparisons

	Chi-Square	df	Sig.
Log Rank (Mantel-Cox)	,055	1	,814
Breslow (Generalized Wilcoxon)	,055	1	,814

Test of equality of survival distributions for the different levels of Aminoglicosyde.

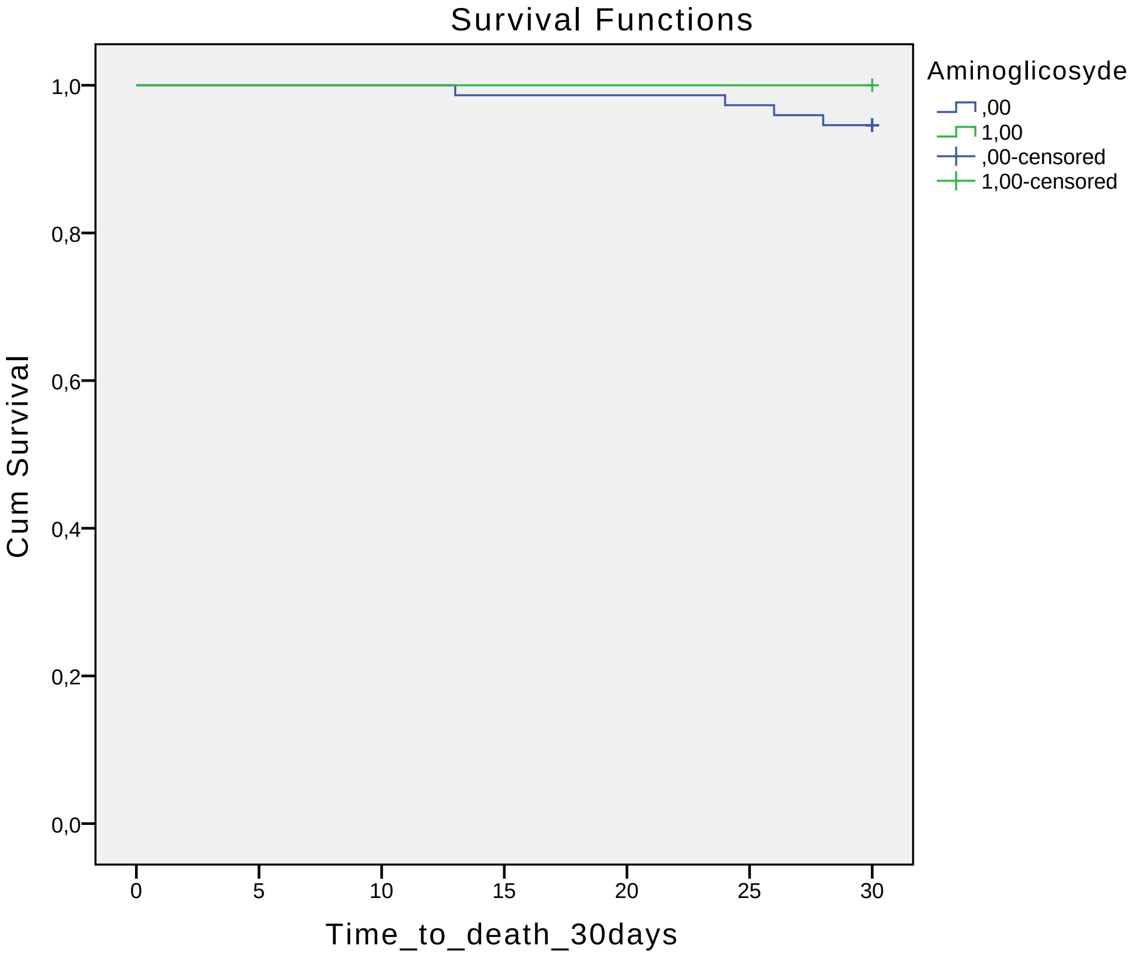

Kaplan-Meier

Case Processing Summary

Short_treatment	Total N	N of Events	Censored	
			N	Percent
,00	40	2	38	95,0%
1,00	35	2	33	94,3%
Overall	75	4	71	94,7%

Means and Medians for Survival Time

Short_treatment	Mean[a]				Median		
			95% Confidence Interval				95% ...
	Estimate	Std. Error	Lower Bound	Upper Bound	Estimate	Std. Error	Lower Bound
,00	29,750	,176	29,405	30,095	.	.	.
1,00	29,457	,480	28,516	30,399	.	.	.
Overall	29,613	,244	29,136	30,091	.	.	.

Means and Medians for Survival Time

Short_treatment	Median
	95% ...
	Upper Bound
,00	.
1,00	.
Overall	.

a. Estimation is limited to the largest survival time if it is censored.

Overall Comparisons

	Chi-Square	df	Sig.
Log Rank (Mantel-Cox)	,019	1	,892
Breslow (Generalized Wilcoxon)	,019	1	,892

Test of equality of survival distributions for the different levels of Short_treatment.

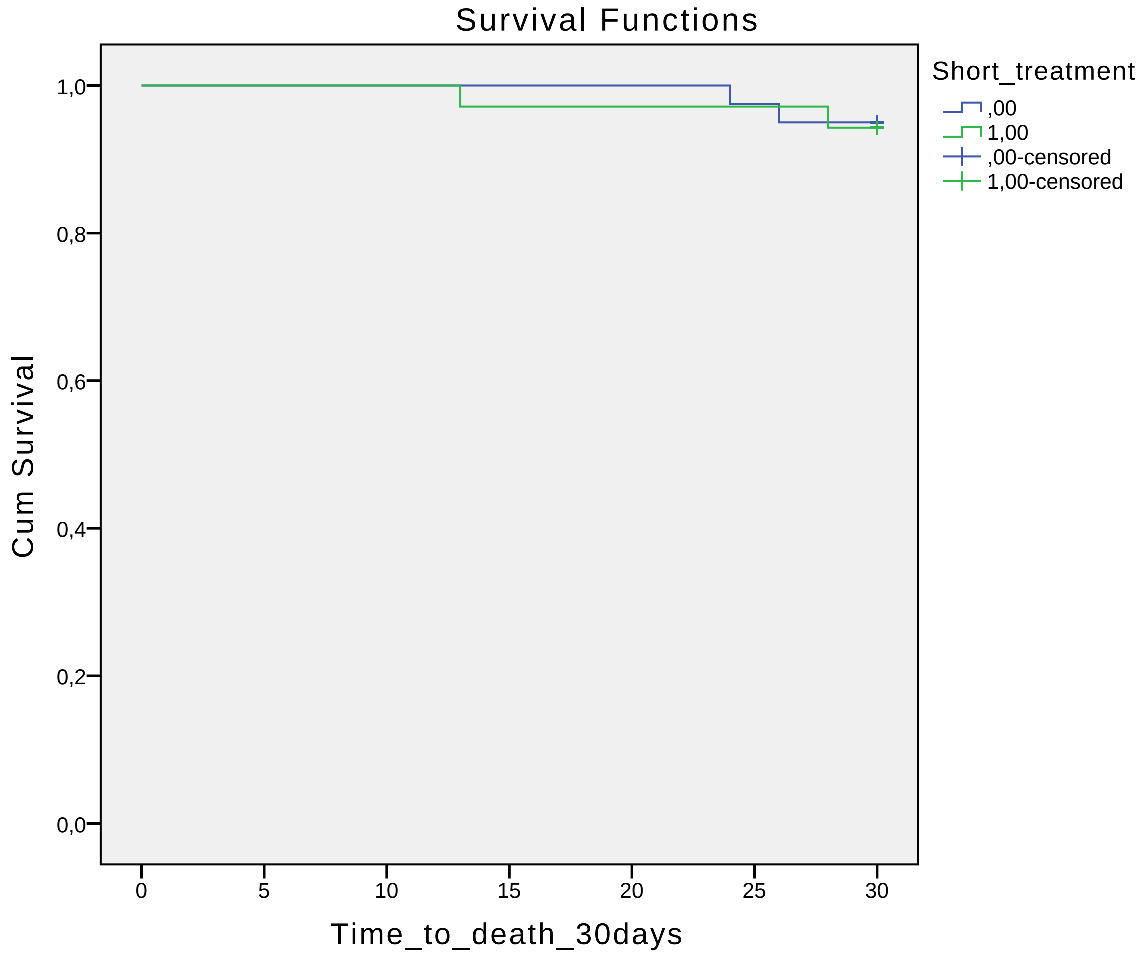

Kaplan-Meier

Case Processing Summary

Barthel_index_below_20	Total N	N of Events	Censored	
			N	Percent
,00	38	3	35	92,1%
1,00	21	1	20	95,2%
Overall	59	4	55	93,2%

Means and Medians for Survival Time

Barthel_index_below_20	Mean[a]				Median		
			95% Confidence Interval				95% ...
	Estimate	Std. Error	Lower Bound	Upper Bound	Estimate	Std. Error	Lower Bound
,00	29,289	,472	28,364	30,215	.	.	.
1,00	29,905	,093	29,723	30,087	.	.	.
Overall	29,508	,308	28,904	30,113	.	.	.

Means and Medians for Survival Time

Barthel_index_below_20	Median
	95% ...
	Upper Bound
,00	.
1,00	.
Overall	.

a. Estimation is limited to the largest survival time if it is censored.

Overall Comparisons

	Chi-Square	df	Sig.
Log Rank (Mantel-Cox)	,230	1	,632
Breslow (Generalized Wilcoxon)	,256	1	,613

Test of equality of survival distributions for the different levels of Barthel_index_below_20.

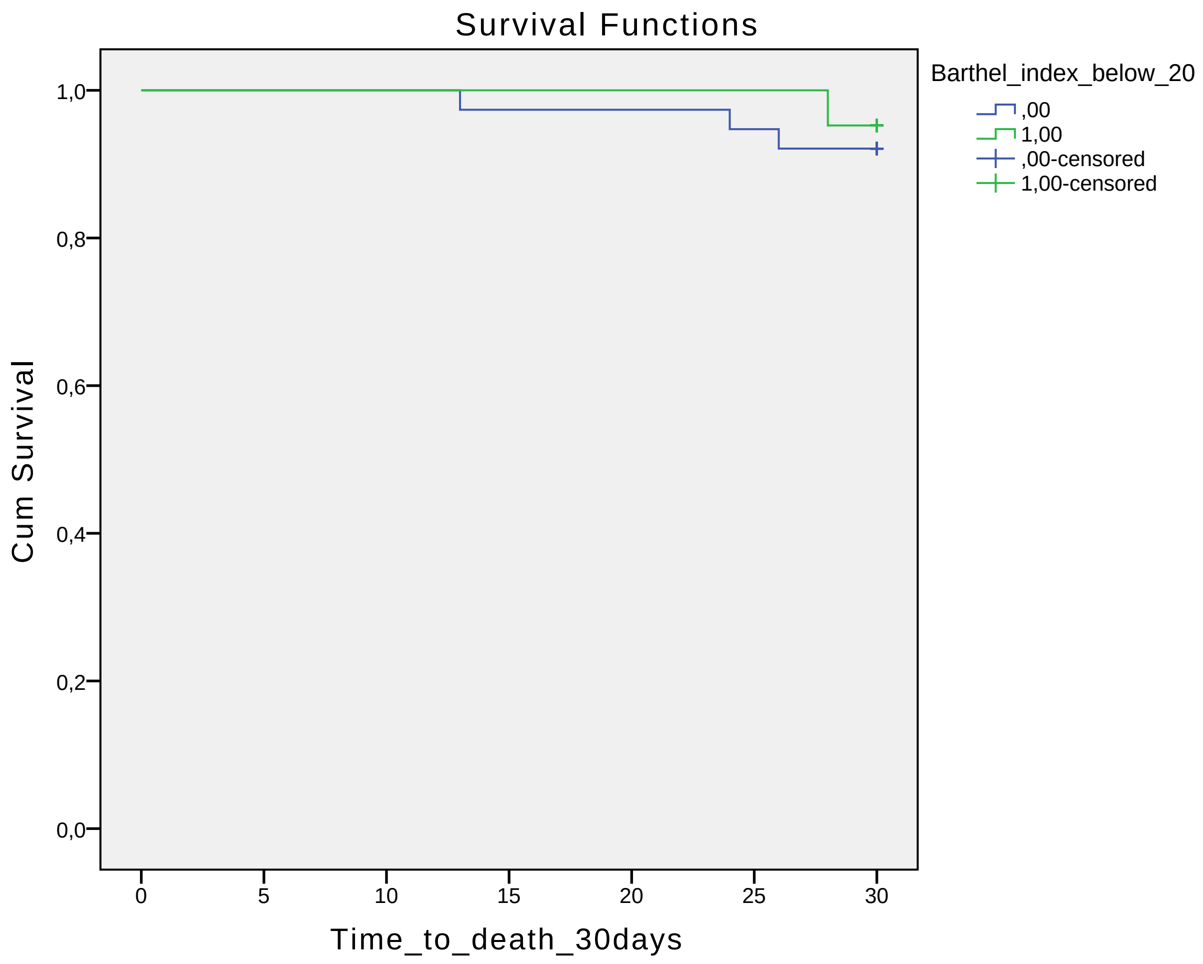

Kaplan-Meier

Warnings

No statistics are computed because all cases are censored.

Case Processing Summary

Age_above_79	Total N	N of Events	Censored	
			N	Percent
,00	41	0	41	100,0%
1,00	34	4	30	88,2%
Overall	75	4	71	94,7%

Overall Comparisons

	Chi-Square	df	Sig.
Log Rank (Mantel-Cox)	5,050	1	,025
Breslow (Generalized Wilcoxon)	5,046	1	,025

Test of equality of survival distributions for the different levels of Age_above_79.

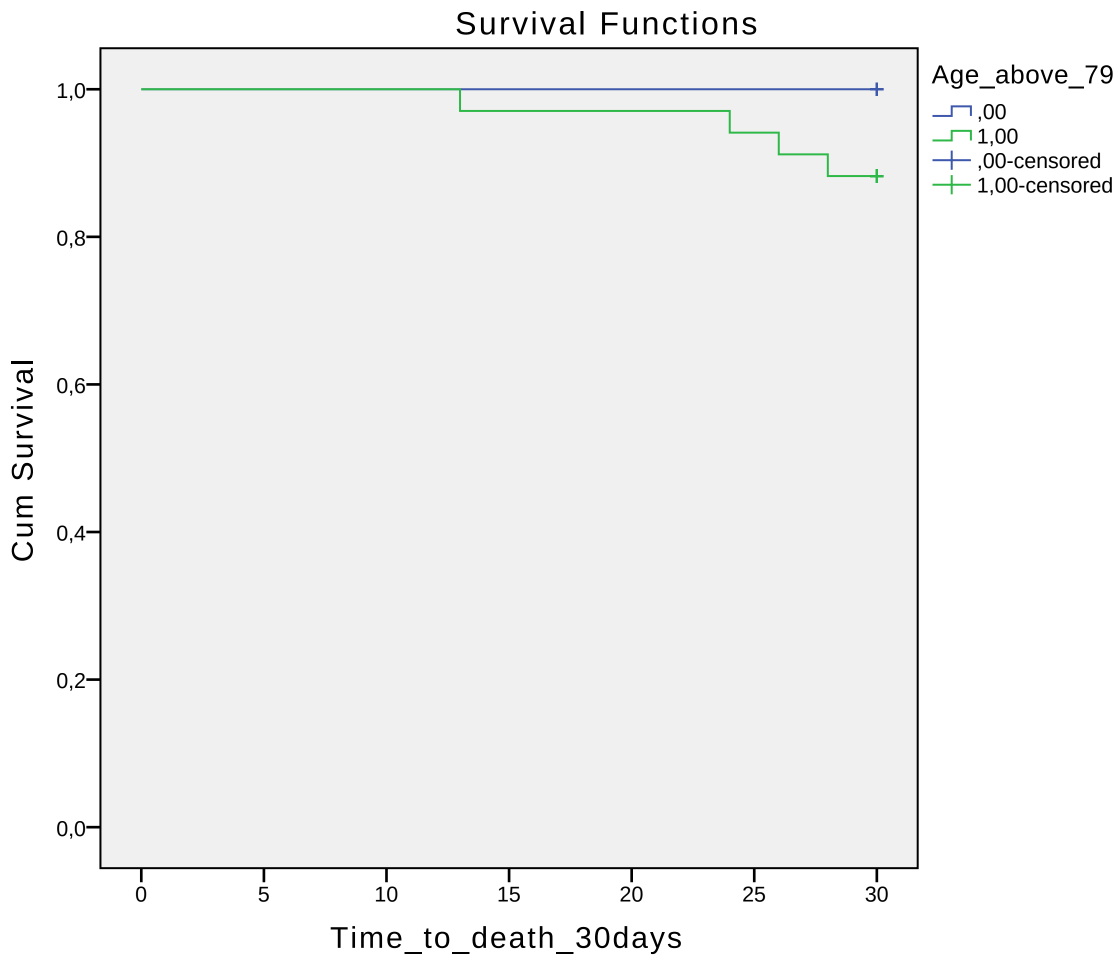

Kaplan-Meier

Case Processing Summary

Cystitis	Total N	N of Events	Censored	
			N	Percent
,00	39	1	38	97,4%
1,00	36	3	33	91,7%
Overall	75	4	71	94,7%

Means and Medians for Survival Time

Cystitis	Mean[a]				Median			
			95% Confidence Interval				95% Confidence Interval	
	Estimate	Std. Error	Lower Bound	Upper Bound	Estimate	Std. Error	Lower Bound	Upper Bound
,00	29,564	,430	28,721	30,407	.	.	.	.
1,00	29,667	,200	29,274	30,059	.	.	.	.
Overall	29,613	,244	29,136	30,091	.	.	.	.

a. Estimation is limited to the largest survival time if it is censored.

Overall Comparisons

	Chi-Square	df	Sig.
Log Rank (Mantel-Cox)	1,172	1	,279
Breslow (Generalized Wilcoxon)	1,128	1	,288

Test of equality of survival distributions for the different levels of Cystitis.

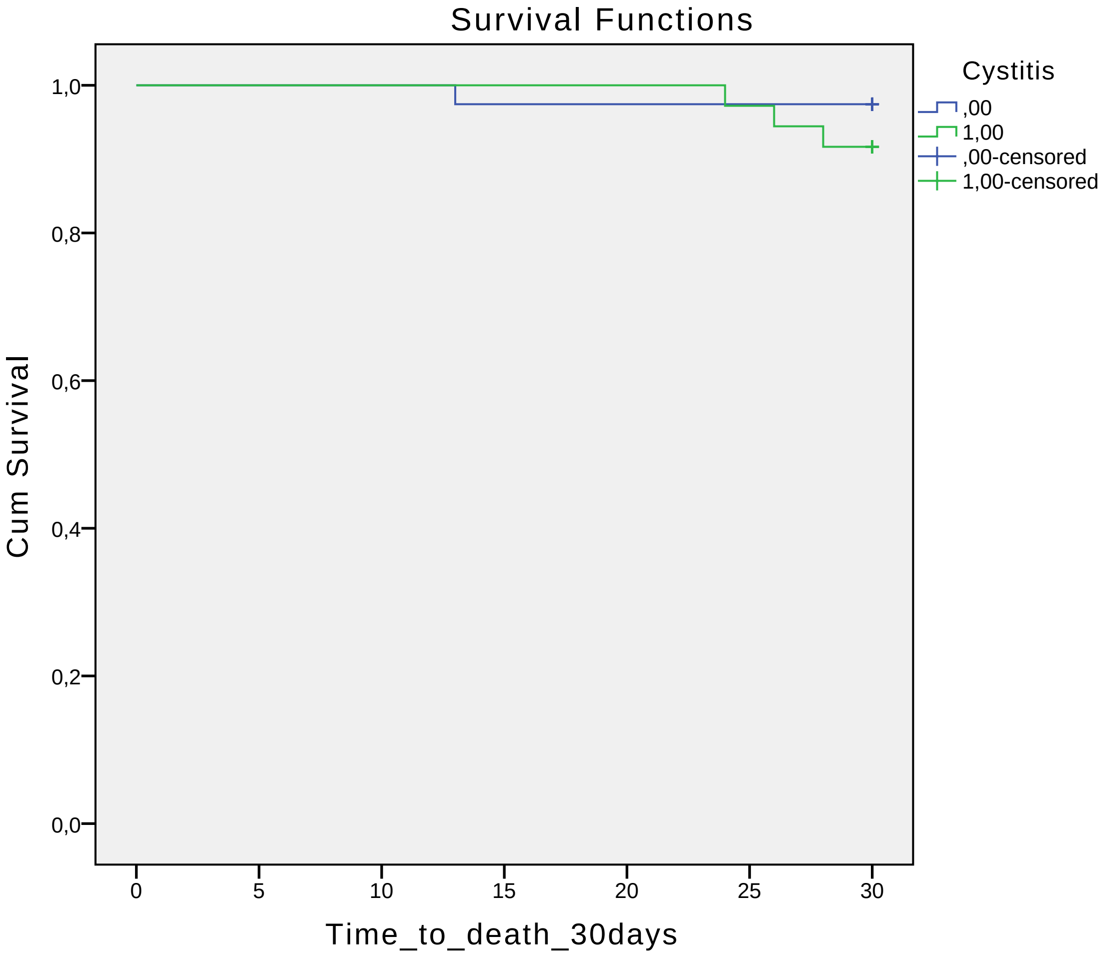

Kaplan-Meier

Case Processing Summary

Febrile_UTI	Total N	N of Events	Censored	
			N	Percent
,00	49	3	46	93,9%
1,00	26	1	25	96,2%
Overall	75	4	71	94,7%

Means and Medians for Survival Time

Febrile_UTI	Mean[a]				Median			
	Estimate	Std. Error	95% Confidence Interval		Estimate	Std. Error	95% Confidence Interval	
			Lower Bound	Upper Bound			Lower Bound	Upper Bound
,00	29,755	,149	29,464	30,046	.	.	.	.
1,00	29,346	,641	28,090	30,603	.	.	.	.
Overall	29,613	,244	29,136	30,091	.	.	.	.

a. Estimation is limited to the largest survival time if it is censored.

Overall Comparisons

	Chi-Square	df	Sig.
Log Rank (Mantel-Cox)	,155	1	,694
Breslow (Generalized Wilcoxon)	,139	1	,710

Test of equality of survival distributions for the different levels of Febrile_UTI.

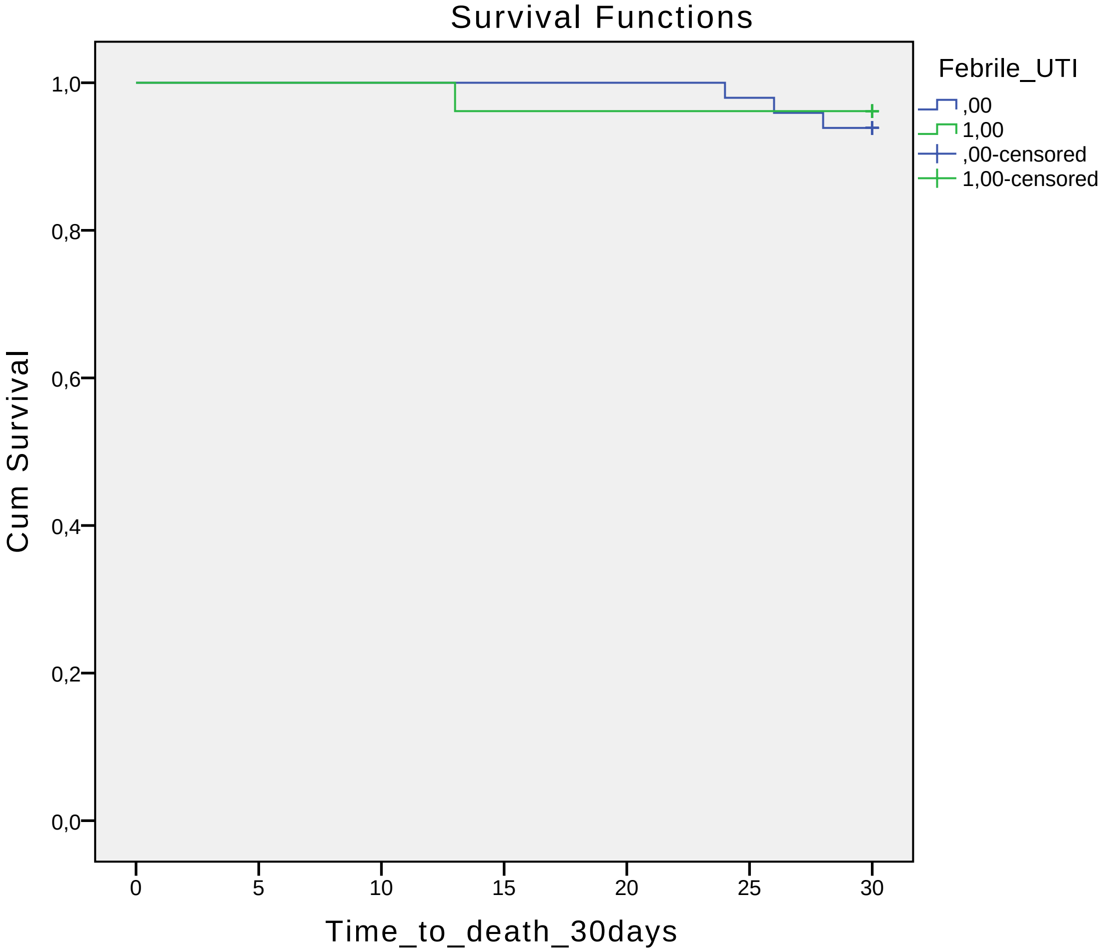

Kaplan-Meier

Warnings

No statistics are computed because all cases are censored.

Case Processing Summary

Pyelonephritis	Total N	N of Events	Censored	
			N	Percent
,00	62	4	58	93,5%
1,00	13	0	13	100,0%
Overall	75	4	71	94,7%

Overall Comparisons

	Chi-Square	df	Sig.
Log Rank (Mantel-Cox)	,860	1	,354
Breslow (Generalized Wilcoxon)	,860	1	,354

Test of equality of survival distributions for the different levels of Pyelonephritis.

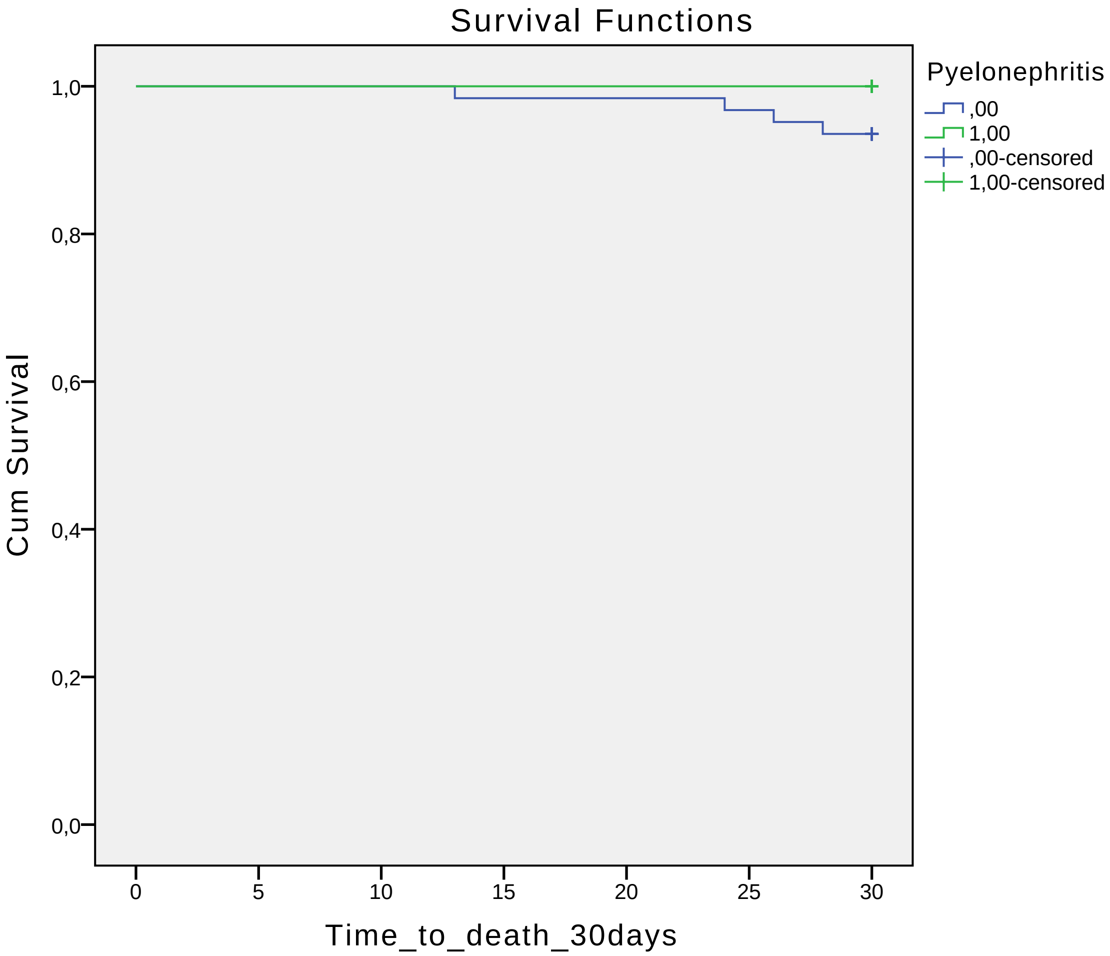

Kaplan-Meier

Warnings

For split file Sex=,0, no statistics are computed because all cases are censored.

Sex = ,0

Case Processing Summary[a]

Short_treatment	Total N	N of Events	Censored	
			N	Percent
,00	22	0	22	100,0%
1,00	10	1	9	90,0%
Overall	32	1	31	96,9%

a. Sex = ,0

Overall Comparisons[a]

	Chi-Square	df	Sig.
Log Rank (Mantel-Cox)	2,200	1	,138
Breslow (Generalized Wilcoxon)	2,200	1	,138

Test of equality of survival distributions for the different levels of Short_treatment.

a. Sex = ,0

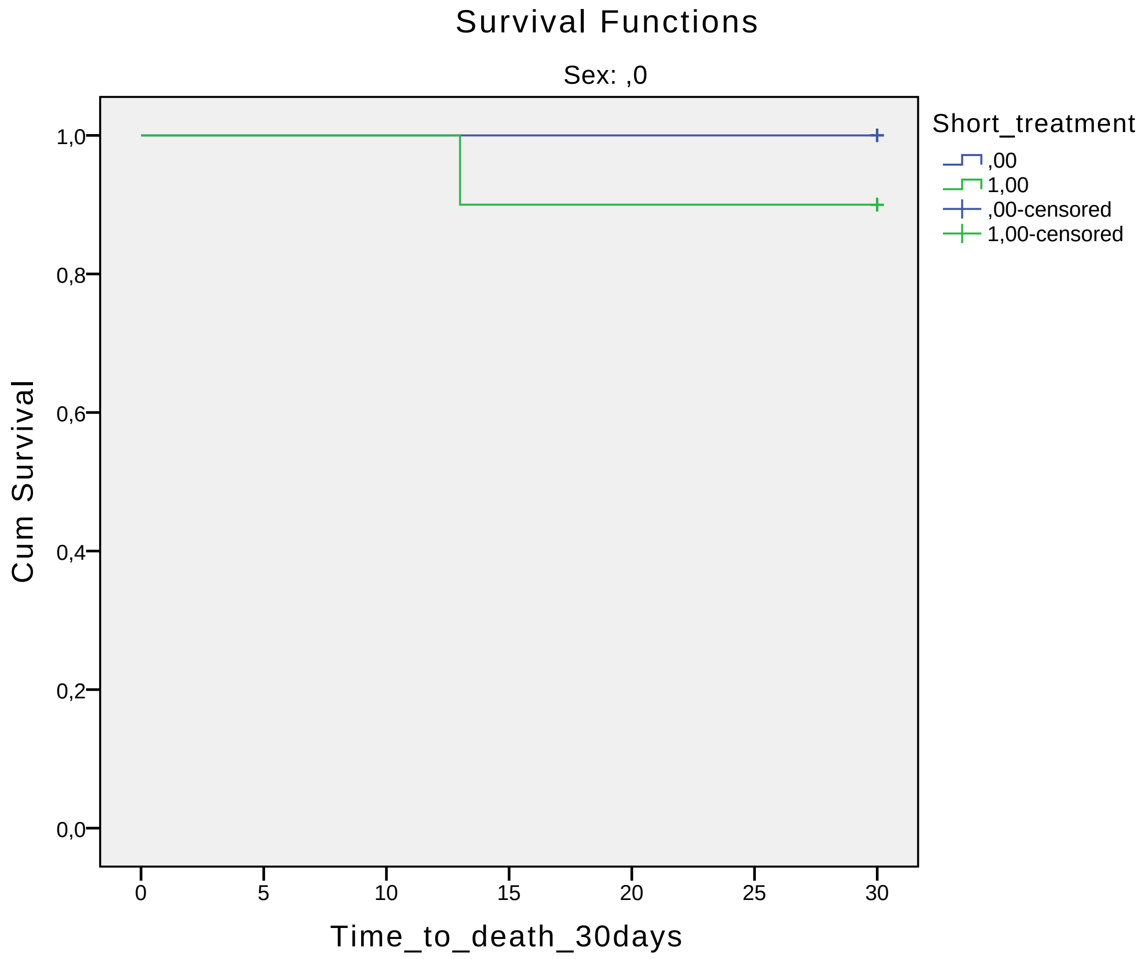

Sex = 1,0

Case Processing Summary[a]

Short_treatment	Total N	N of Events	Censored	
			N	Percent
,00	18	2	16	88,9%
1,00	25	1	24	96,0%
Overall	43	3	40	93,0%

a. Sex = 1,0

Means and Medians for Survival Time[a]

Short_treatment	Mean[b] Estimate	Mean Std. Error	Mean 95% Confidence Interval Lower Bound	Mean 95% Confidence Interval Upper Bound	Median Estimate	Median Std. Error	Median 95% … Lower Bound
,00	29,444	,379	28,702	30,187	.	.	.
1,00	29,920	,078	29,766	30,074	.	.	.
Overall	29,721	,169	29,390	30,052	.	.	.

Means and Medians for Survival Time[a]

Short_treatment	Median 95% … Upper Bound
,00	.
1,00	.
Overall	.

a. Sex = 1,0

b. Estimation is limited to the largest survival time if it is censored.

Overall Comparisons[a]

	Chi-Square	df	Sig.
Log Rank (Mantel-Cox)	,856	1	,355
Breslow (Generalized Wilcoxon)	,907	1	,341

Test of equality of survival distributions for the different levels of Short_treatment.

a. Sex = 1,0

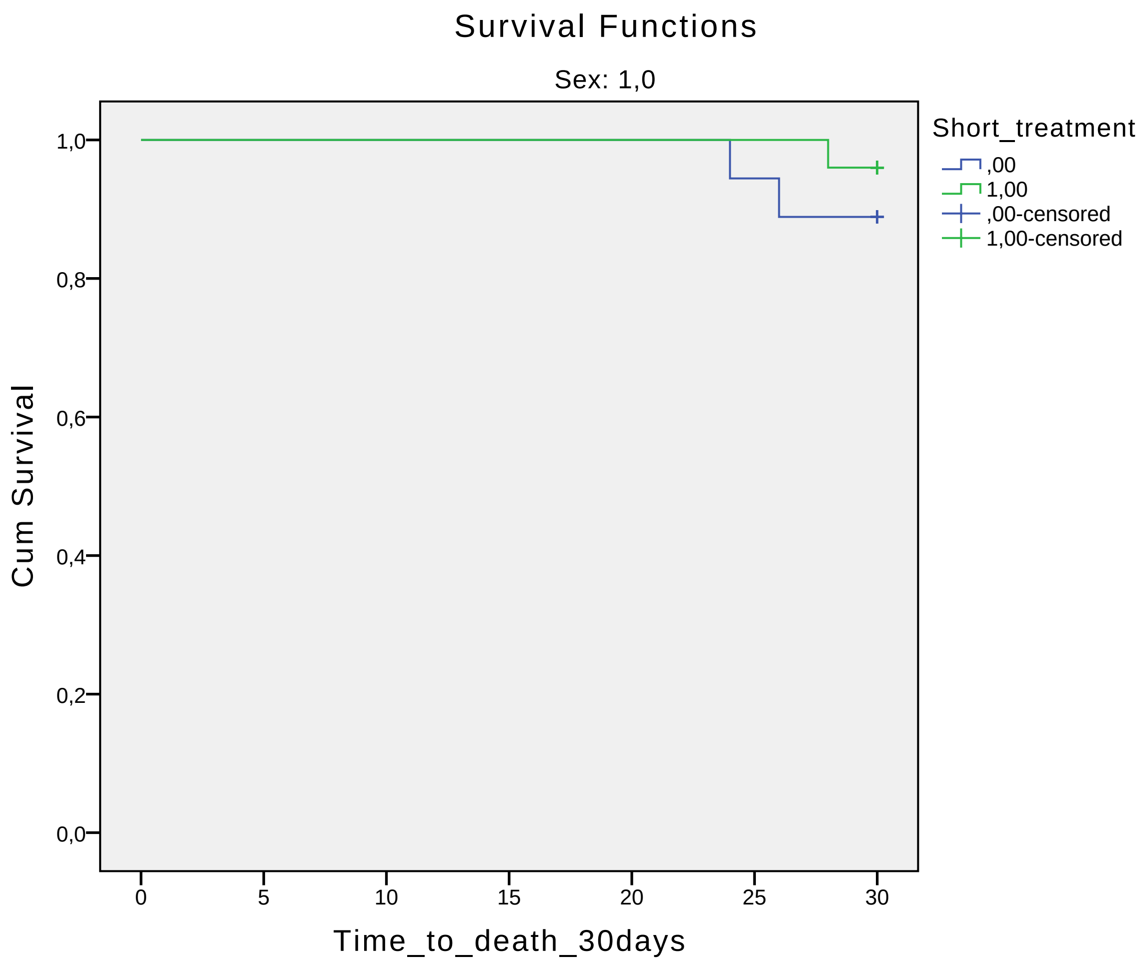

Supplement: S3 File — Bivariant analysis. (PDF) [file pone.0237365.s003.pdf]
