# Supplementary material for: Treatment duration of complicated urinary tract infections by extended-spectrum beta-lactamases producing enterobacterales
Source: PLoS One. 2020 Oct 19;15(10):e0237365. doi: 10.1371/journal.pone.0237365 (PMC7571686; doi:10.1371/journal.pone.0237365)
Supplement: S4 File — Bivariant analysis. (PDF) [file pone.0237365.s004.pdf]

Cox Regression

Case Processing Summary

		N	Percent
Cases available in analysis	Event[a]	4	5,3%
	Censored	67	89,3%
	Total	71	94,7%
Cases dropped	Cases with missing values	4	5,3%
	Cases with negative time	0	0,0%
	Censored cases before the earliest event in a stratum	0	0,0%
	Total	4	5,3%
Total		75	100,0%

a. Dependent Variable: Time_to_death_30days

Block 0: Beginning Block

Variables not in the Equation[a]

	Score	df	Sig.
Sex	,479	1	,489
Age	4,118	1	,042
Charlson_index	,297	1	,586
Lymphoma	8,277	1	,004
Metastatic_solid_tumor	8,277	1	,004
Leukopenia	7,002	1	,008
Short_treatment	,041	1	,840
PS	,479	1	,489

a. Residual Chi Square = 24,352 with 7 df Sig. = ,001

Block 1: Method = Forward Stepwise (Wald)

Omnibus Tests of Model Coefficients[c]

Step	-2 Log Likelihood	Overall (score)			Change From Previous Step			Change From Previous …	
		Chi-square	df	Sig.	Chi-square	df	Sig.	Chi-square	df
1[a]	30,894	8,277	1	,004	3,036	1	,081	3,036	1
2[b]	27,548	15,711	2	,000	3,345	1	,067	6,381	2

Omnibus Tests of Model Coefficients[c]

Step	Change …
	Sig.
1[a]	,081
2[b]	,041

a. Variable(s) Entered at Step Number 1: Metastatic_solid_tumor

b. Variable(s) Entered at Step Number 2: Leukopenia

c. Beginning Block Number 1. Method = Forward Stepwise (Wald)

Variables in the Equation

		B	SE	Wald	df	Sig.	Exp(B)	95,0% CI for Exp(B)	
								Lower	Upper
Step 1	Metastatic_solid_tumor	,429	,194	4,909	1	,027	1,536	1,051	2,245
Step 2	Metastatic_solid_tumor	,492	,205	5,735	1	,017	1,635	1,093	2,445
	Leukopenia	2,799	1,225	5,223	1	,022	16,429	1,490	181,187

Variables not in the Equation[a,b]

		Score	df	Sig.
Step 1	Sex	,118	1	,731
	Age	3,663	1	,056
	Charlson_index	,674	1	,412
	Lymphoma	1,030	1	,310
	Leukopenia	9,659	1	,002
	Short_treatment	,006	1	,937
	PS	,118	1	,731
Step 2	Sex	,175	1	,676
	Age	2,737	1	,098
	Charlson_index	1,606	1	,205
	Lymphoma	1,138	1	,286
	Short_treatment	,268	1	,605
	PS	,175	1	,676

a. Residual Chi Square = 16,767 with 6 df Sig. = ,010

b. Residual Chi Square = 5,076 with 5 df Sig. = ,407

Covariate Means

	Mean
Sex	,577
Age	75,549
Charlson_index	2,690
Lymphoma	,056
Metastatic_solid_tumor	,169
Leukopenia	,028
Short_treatment	,451
PS	,468
