# Supplementary material for: Treatment duration of complicated urinary tract infections by extended-spectrum beta-lactamases producing enterobacterales
Source: PLoS One. 2020 Oct 19;15(10):e0237365. doi: 10.1371/journal.pone.0237365 (PMC7571686; doi:10.1371/journal.pone.0237365)
Supplement: S5 File — Multivariate analysis. (PDF) [file pone.0237365.s005.pdf]

Kaplan-Meier

Case Processing Summary

Short_treatment	Total N	N of Events	Censored	
			N	Percent
,00	40	4	36	90,0%
1,00	35	3	32	91,4%
Overall	75	7	68	90,7%

Means and Medians for Survival Time

Short_treatment	Mean[a]				Median		
			95% Confidence Interval				95% …
	Estimate	Std. Error	Lower Bound	Upper Bound	Estimate	Std. Error	Lower Bound
,00	29,325	,345	28,648	30,002	.	.	.
1,00	29,171	,549	28,096	30,247	.	.	.
Overall	29,253	,316	28,635	29,872	.	.	.

Means and Medians for Survival Time

Short_treatment	Median
	95% …
	Upper Bound
,00	.
1,00	.
Overall	.

a. Estimation is limited to the largest survival time if it is censored.

Overall Comparisons

	Chi-Square	df	Sig.
Log Rank (Mantel-Cox)	,038	1	,845
Breslow (Generalized Wilcoxon)	,036	1	,850

Test of equality of survival distributions for the different levels of Short_treatment.

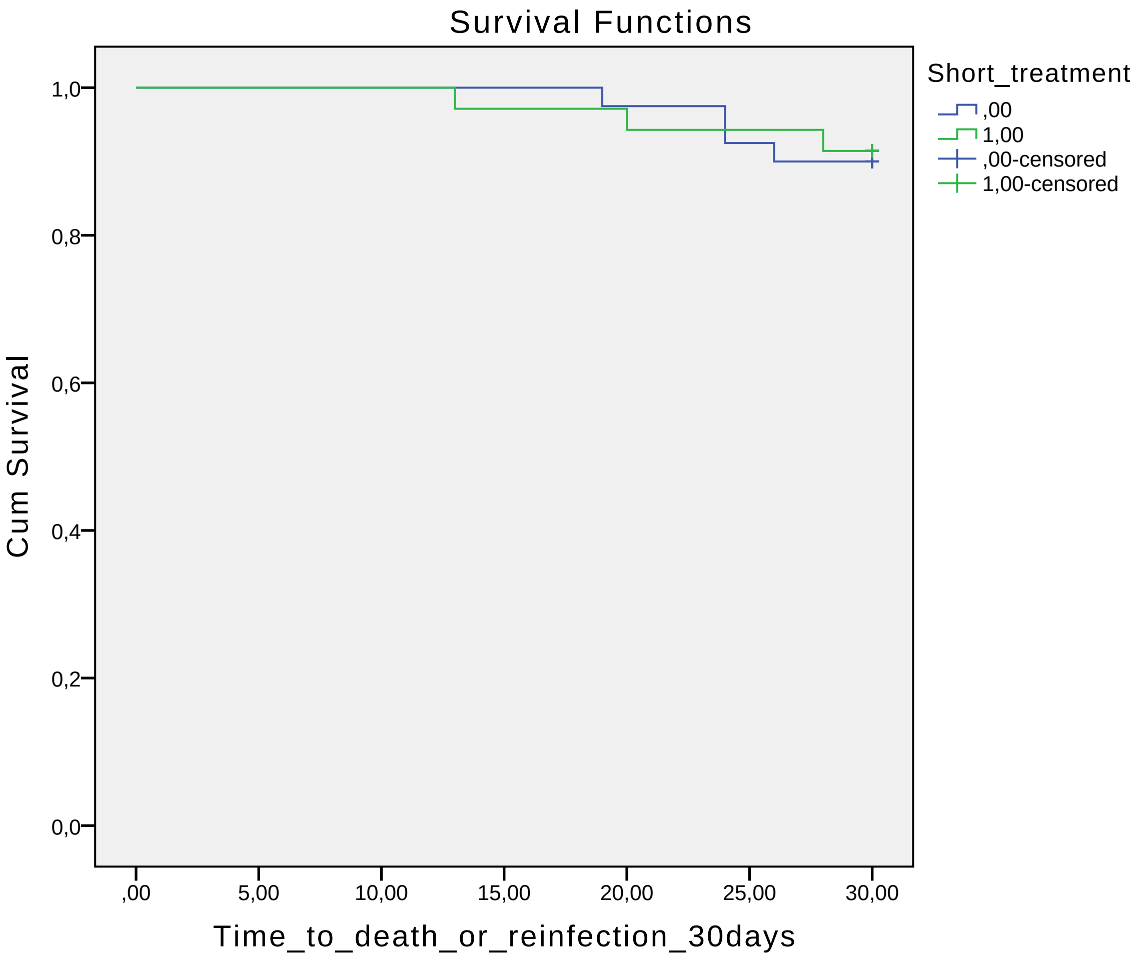

Kaplan-Meier

Case Processing Summary

Urolithiasis	Total N	N of Events	Censored	
			N	Percent
,00	62	6	56	90,3%
1,00	13	1	12	92,3%
Overall	75	7	68	90,7%

Means and Medians for Survival Time

Urolithiasis	Mean[a]				Median			
	Estimate	Std. Error	95% Confidence Interval		Estimate	Std. Error	95% Confidence Interval	
			Lower Bound	Upper Bound			Lower Bound	Upper Bound
,00	29,258	,349	28,574	29,942	.	.	.	.
1,00	29,231	,739	27,782	30,679	.	.	.	.
Overall	29,253	,316	28,635	29,872	.	.	.	.

a. Estimation is limited to the largest survival time if it is censored.

Overall Comparisons

	Chi-Square	df	Sig.
Log Rank (Mantel-Cox)	,043	1	,836
Breslow (Generalized Wilcoxon)	,038	1	,846

Test of equality of survival distributions for the different levels of Urolithiasis.

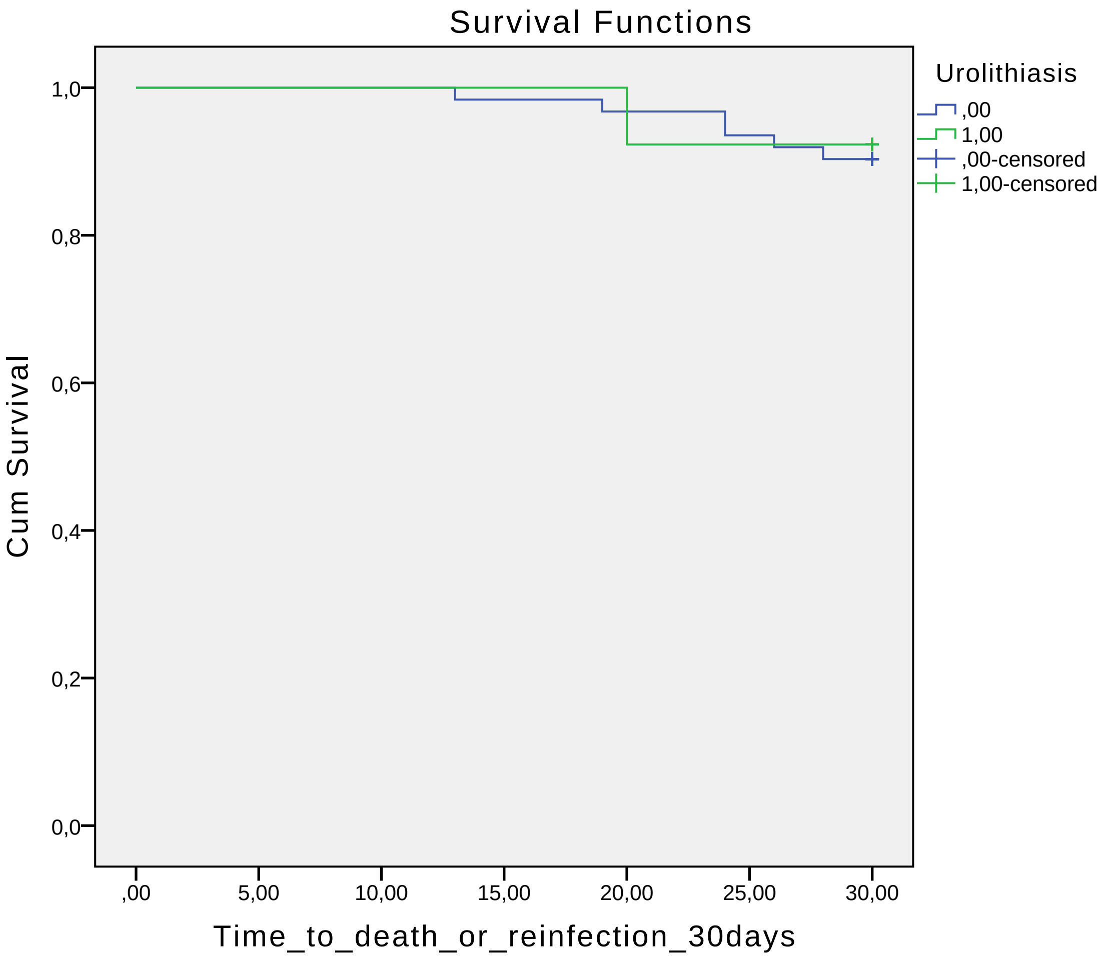

Kaplan-Meier

Warnings

No statistics are computed because all cases are censored.

Case Processing Summary

Nephrostomy	Total N	N of Events	Censored	
			N	Percent
,00	73	7	66	90,4%
1,00	2	0	2	100,0%
Overall	75	7	68	90,7%

Overall Comparisons

	Chi-Square	df	Sig.
Log Rank (Mantel-Cox)	,201	1	,654
Breslow (Generalized Wilcoxon)	,200	1	,654

Test of equality of survival distributions for the different levels of Nephrostomy.

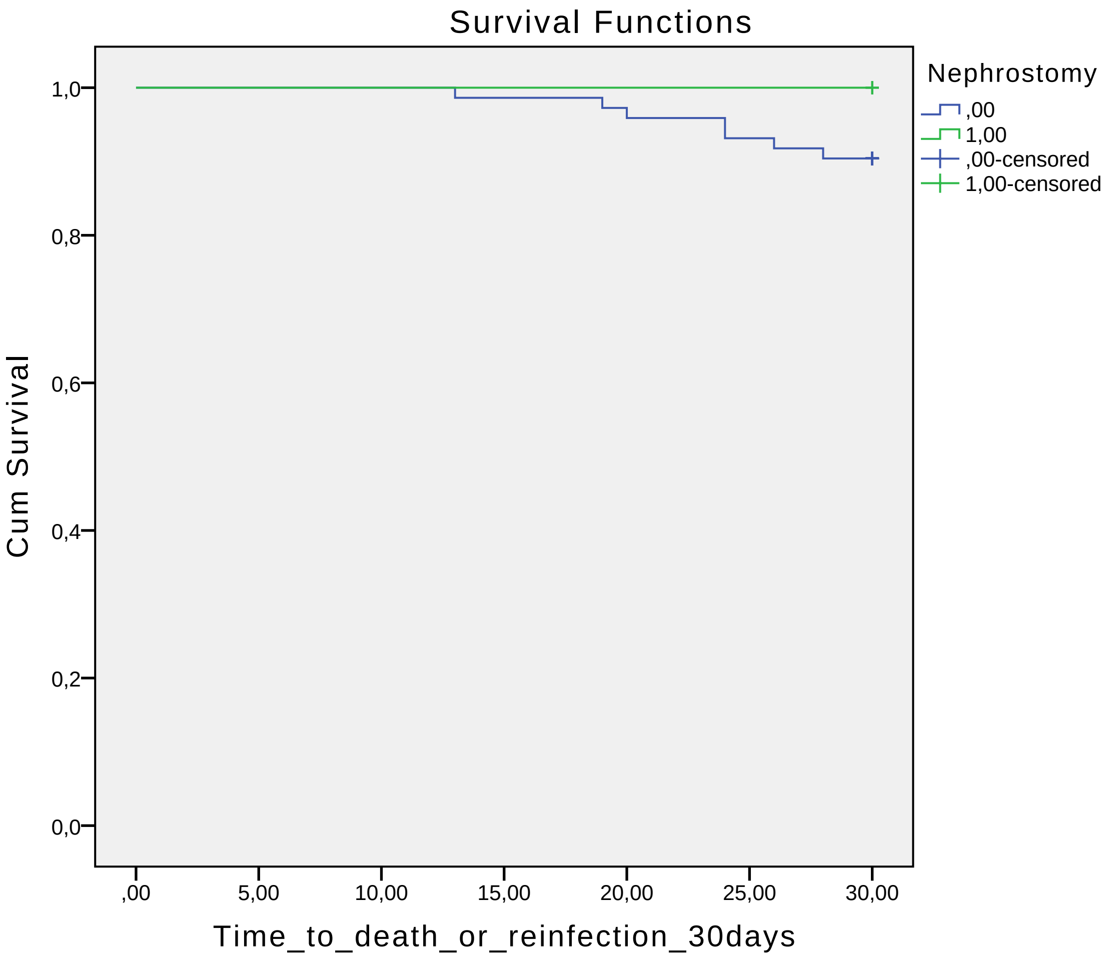

Kaplan-Meier

Case Processing Summary

E_COLLI	Total N	N of Events	Censored	
			N	Percent
,0	14	1	13	92,9%
1,0	61	6	55	90,2%
Overall	75	7	68	90,7%

Means and Medians for Survival Time

E_COLLI	Mean[a]				Median			
	Estimate	Std. Error	95% Confidence Interval		Estimate	Std. Error	95% Confidence Interval	
			Lower Bound	Upper Bound			Lower Bound	Upper Bound
,0	29,571	,413	28,762	30,381	.	.	.	.
1,0	29,180	,376	28,444	29,917	.	.	.	.
Overall	29,253	,316	28,635	29,872	.	.	.	.

a. Estimation is limited to the largest survival time if it is censored.

Overall Comparisons

	Chi-Square	df	Sig.
Log Rank (Mantel-Cox)	,102	1	,750
Breslow (Generalized Wilcoxon)	,103	1	,748

Test of equality of survival distributions for the different levels of E_COLLI.

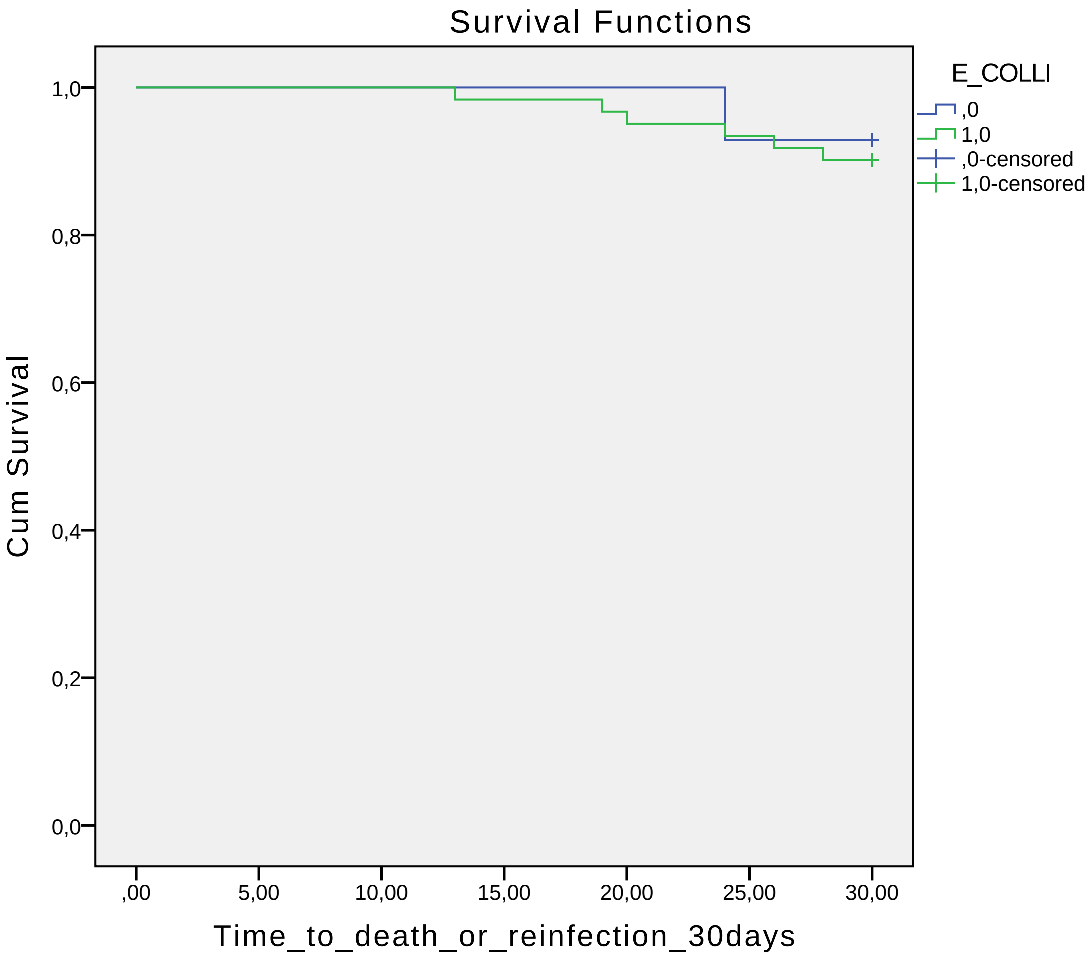

Kaplan-Meier

Case Processing Summary

KLEBSIELLA	Total N	N of Events	Censored	
			N	Percent
,0	64	6	58	90,6%
1,0	11	1	10	90,9%
Overall	75	7	68	90,7%

Means and Medians for Survival Time

KLEBSIELLA	Mean[a]				Median			
	Estimate	Std. Error	95% Confidence Interval		Estimate	Std. Error	95% Confidence Interval	
			Lower Bound	Upper Bound			Lower Bound	Upper Bound
,0	29,219	,359	28,516	29,922	.	.	.	.
1,0	29,455	,520	28,435	30,474	.	.	.	.
Overall	29,253	,316	28,635	29,872	.	.	.	.

a. Estimation is limited to the largest survival time if it is censored.

Overall Comparisons

	Chi-Square	df	Sig.
Log Rank (Mantel-Cox)	,002	1	,967
Breslow (Generalized Wilcoxon)	,002	1	,965

Test of equality of survival distributions for the different levels of KLEBSIELLA.

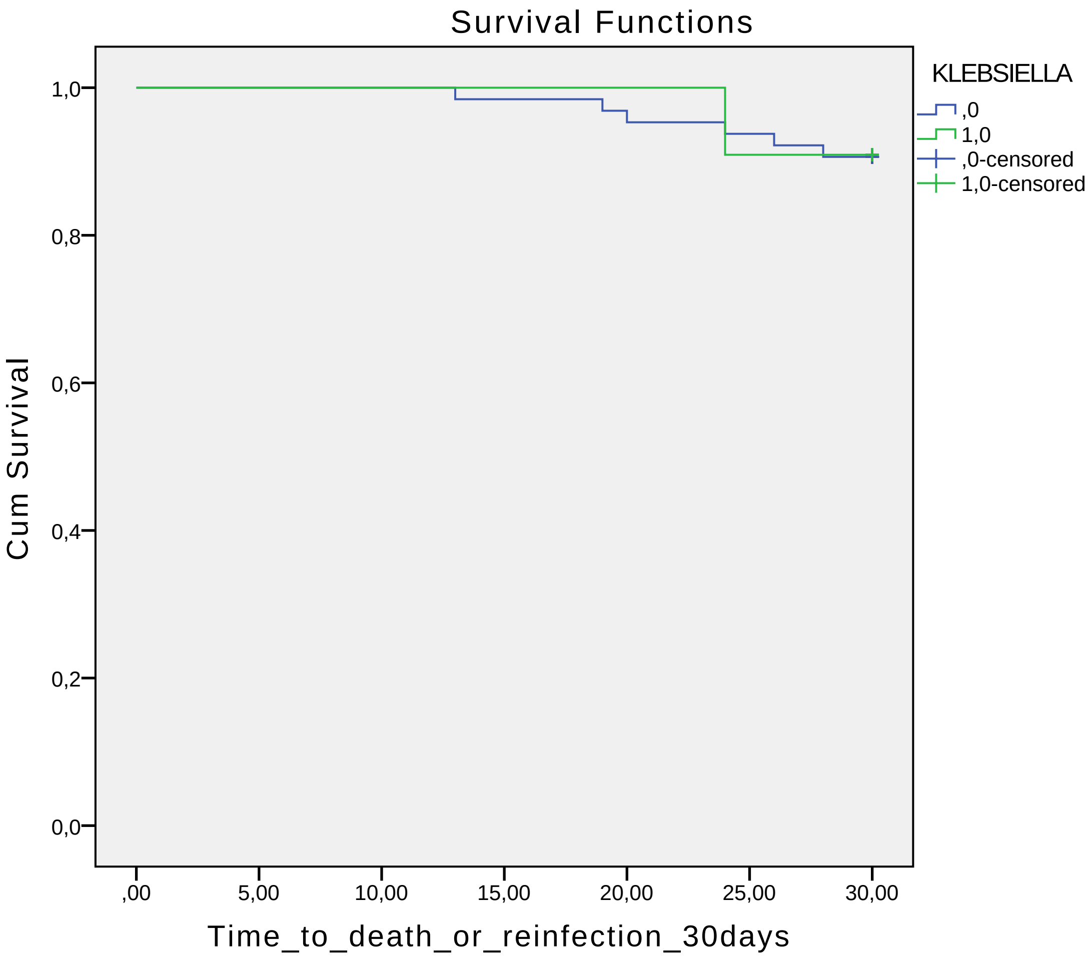

Kaplan-Meier

Warnings

No statistics are computed because all cases are censored.

Case Processing Summary

PROTEUS	Total N	N of Events	Censored	
			N	Percent
,0	73	7	66	90,4%
1,0	2	0	2	100,0%
Overall	75	7	68	90,7%

Overall Comparisons

	Chi-Square	df	Sig.
Log Rank (Mantel-Cox)	,201	1	,654
Breslow (Generalized Wilcoxon)	,200	1	,654

Test of equality of survival distributions for the different levels of PROTEUS.

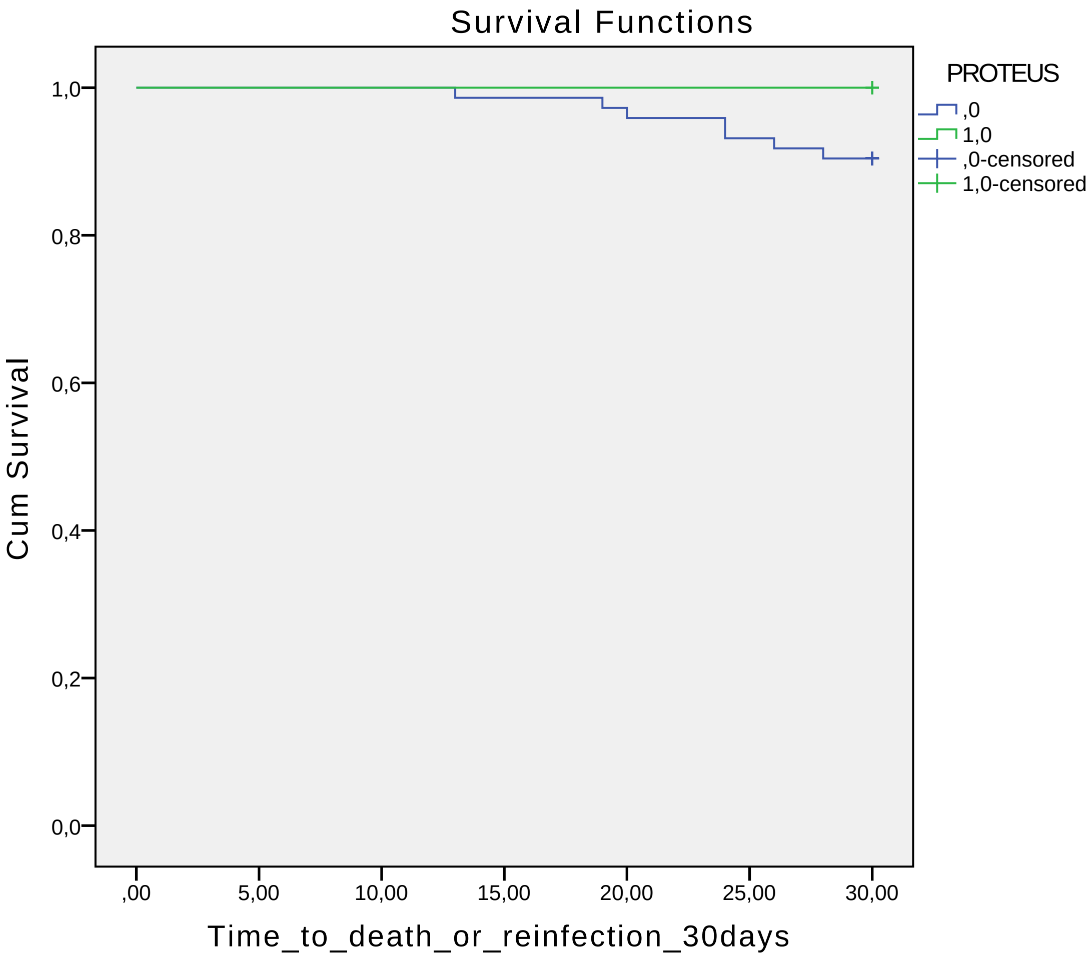

Kaplan-Meier

Warnings

No statistics are computed because all cases are censored.

Case Processing Summary

ENTEROBACTER	Total N	N of Events	Censored	
			N	Percent
,0	74	7	67	90,5%
1,0	1	0	1	100,0%
Overall	75	7	68	90,7%

Overall Comparisons

	Chi-Square	df	Sig.
Log Rank (Mantel-Cox)	,099	1	,753
Breslow (Generalized Wilcoxon)	,099	1	,753

Test of equality of survival distributions for the different levels of ENTEROBACTER.

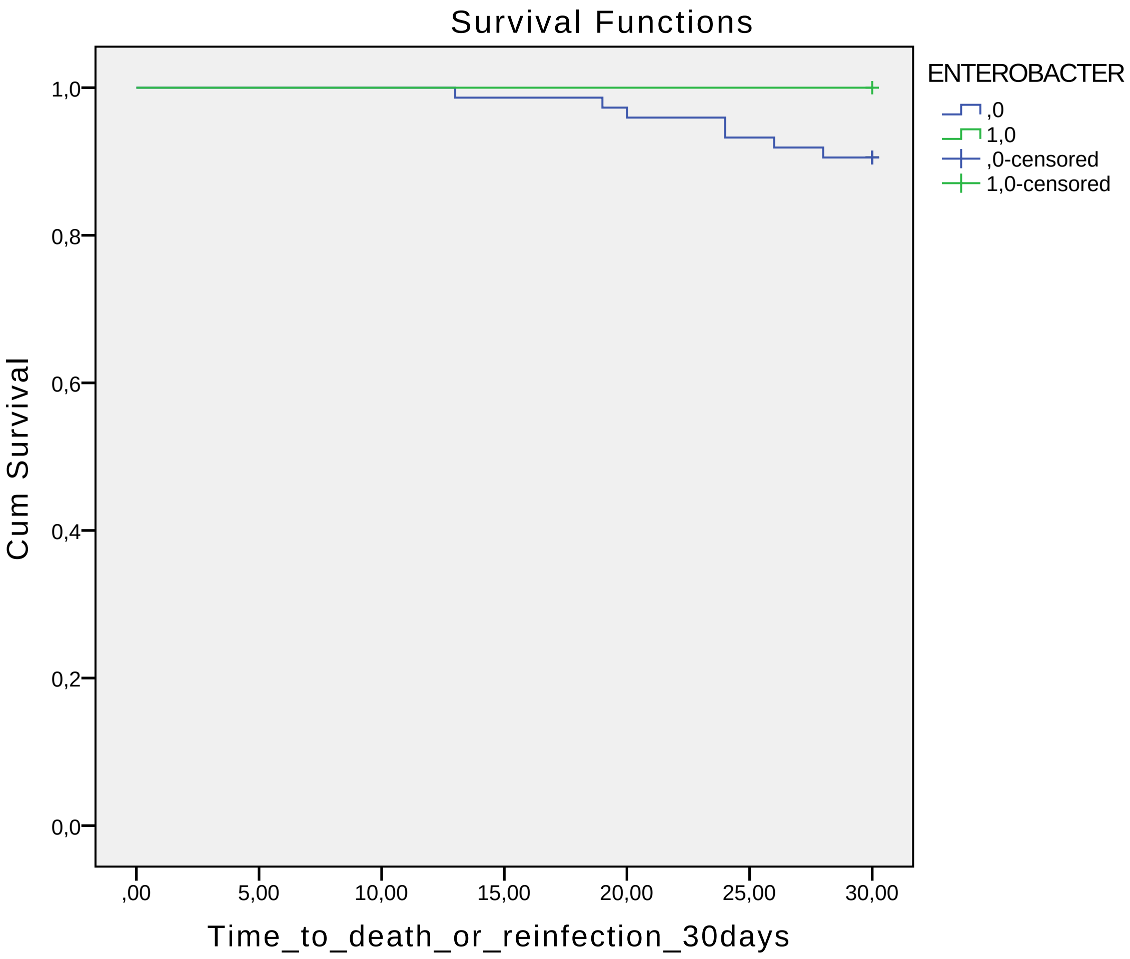

Kaplan-Meier

Warnings

No comparison analysis is performed because the factor variable has only one value for every stratum.

Case Processing Summary

Citrobacter	Total N	N of Events	Censored	
			N	Percent
,0	75	7	68	90,7%
Overall	75	7	68	90,7%

Means and Medians for Survival Time

Citrobacter	Mean[a]				Median			
	Estimate	Std. Error	95% Confidence Interval		Estimate	Std. Error	95% Confidence Interval	
			Lower Bound	Upper Bound			Lower Bound	Upper Bound
,0	29,253	,316	28,635	29,872	.	.	.	.
Overall	29,253	,316	28,635	29,872	.	.	.	.

a. Estimation is limited to the largest survival time if it is censored.

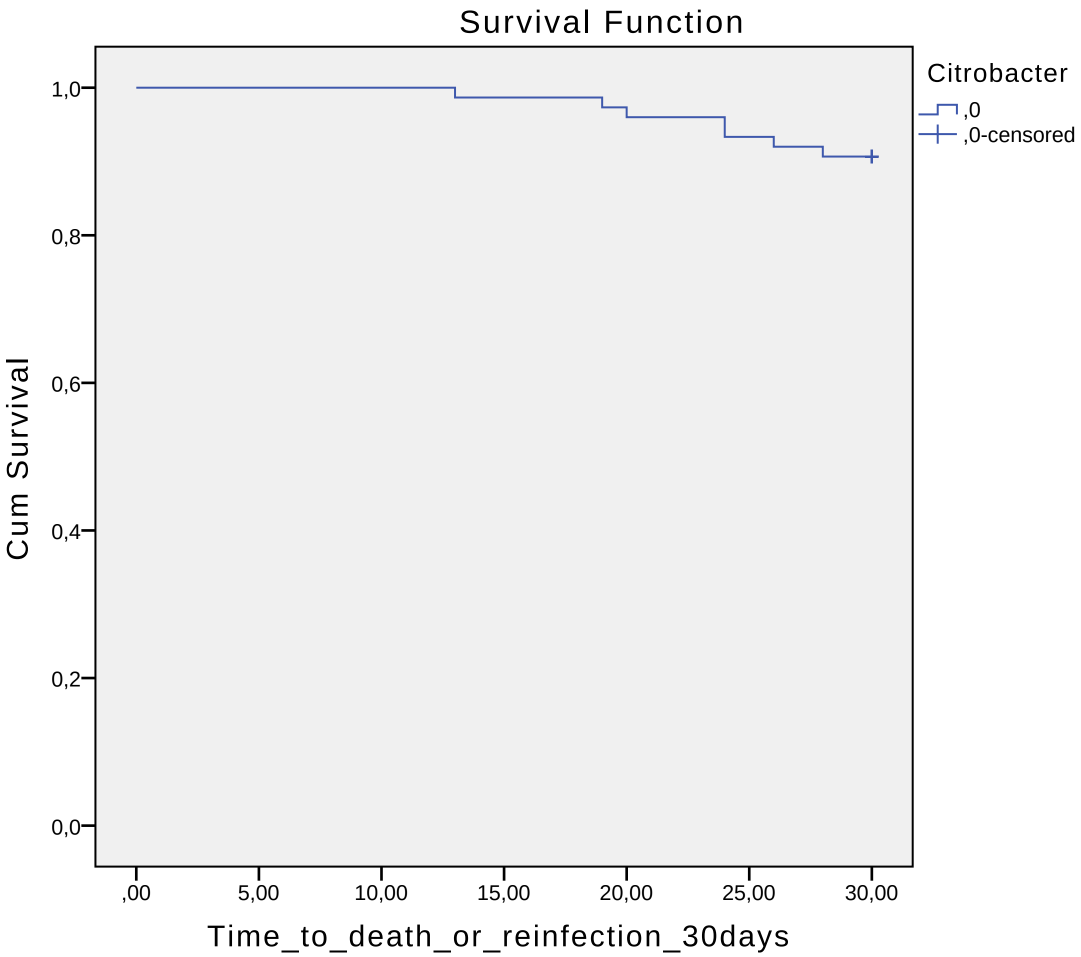

Kaplan-Meier

Warnings

No comparison analysis is performed because the factor variable has only one value for every stratum.

Case Processing Summary

Morganella	Total N	N of Events	Censored	
			N	Percent
,0	75	7	68	90,7%
Overall	75	7	68	90,7%

Means and Medians for Survival Time

Morganella	Mean[a]				Median			
	Estimate	Std. Error	95% Confidence Interval		Estimate	Std. Error	95% Confidence Interval	
			Lower Bound	Upper Bound			Lower Bound	Upper Bound
,0	29,253	,316	28,635	29,872	.	.	.	.
Overall	29,253	,316	28,635	29,872	.	.	.	.

a. Estimation is limited to the largest survival time if it is censored.

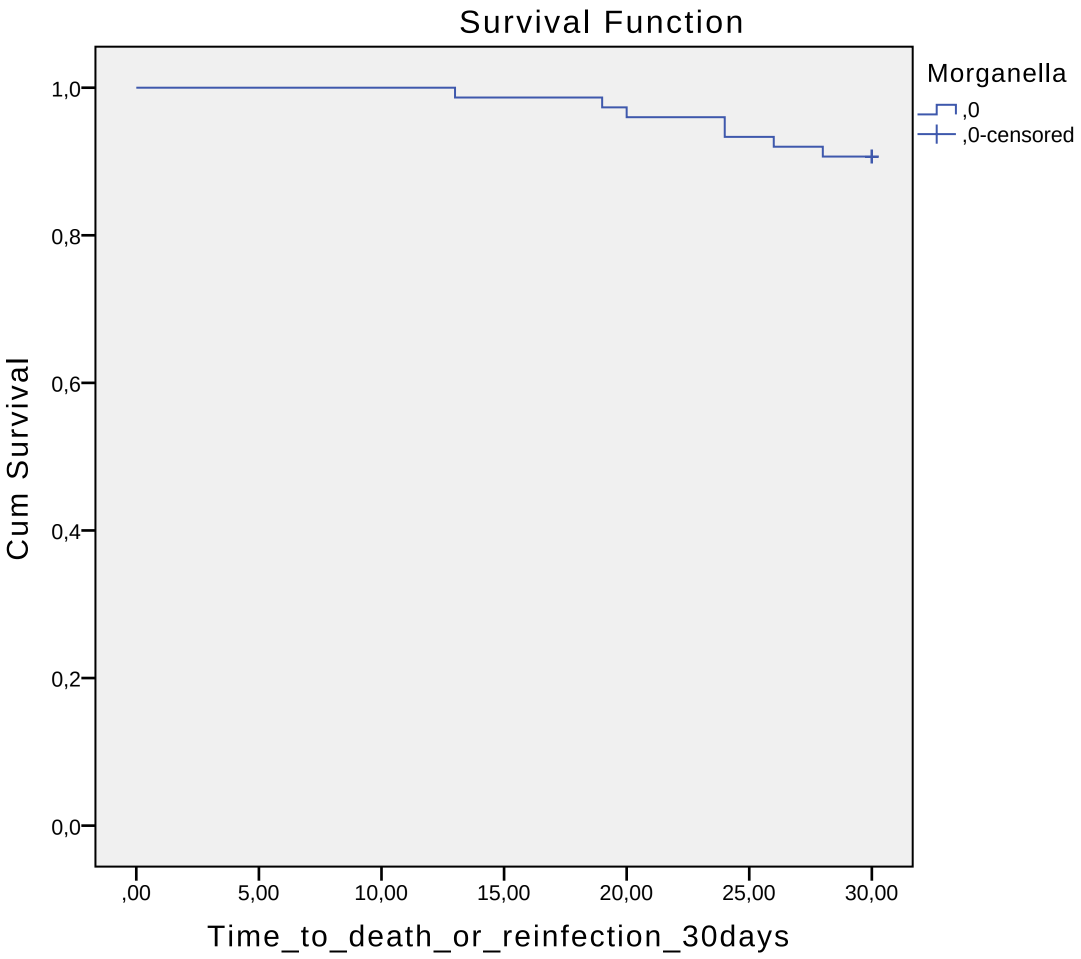

Kaplan-Meier

Warnings

No statistics are computed because all cases are censored.

Case Processing Summary

SEPSIS_SHOCK	Total N	N of Events	Censored	
			N	Percent
,00	67	7	60	89,6%
1,00	8	0	8	100,0%
Overall	75	7	68	90,7%

Overall Comparisons

	Chi-Square	df	Sig.
Log Rank (Mantel-Cox)	,877	1	,349
Breslow (Generalized Wilcoxon)	,877	1	,349

Test of equality of survival distributions for the different levels of SEPSIS_SHOCK.

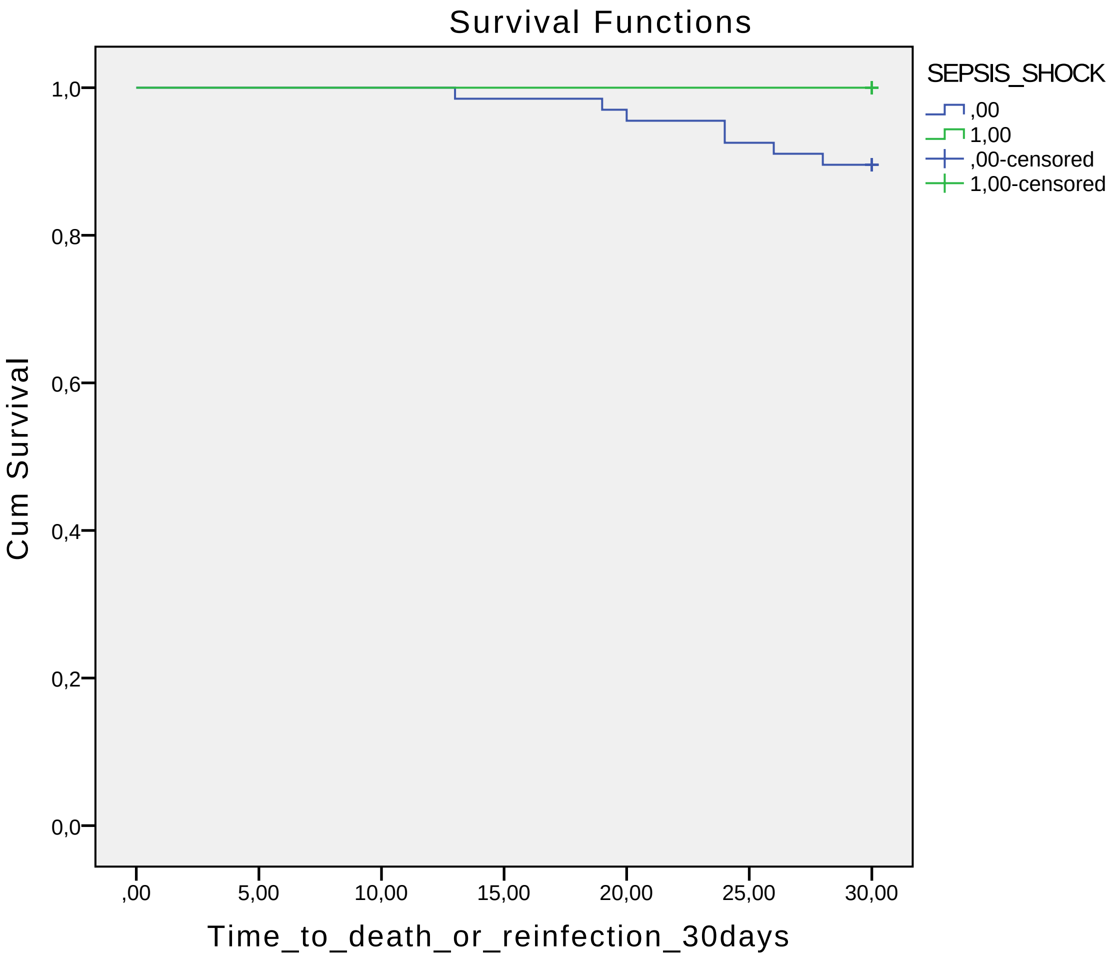

Kaplan-Meier

Warnings

No statistics are computed because all cases are censored.

Case Processing Summary

Sepsis	Total N	N of Events	Censored	
			N	Percent
,0	68	7	61	89,7%
1,0	7	0	7	100,0%
Overall	75	7	68	90,7%

Overall Comparisons

	Chi-Square	df	Sig.
Log Rank (Mantel-Cox)	,756	1	,385
Breslow (Generalized Wilcoxon)	,755	1	,385

Test of equality of survival distributions for the different levels of Sepsis.

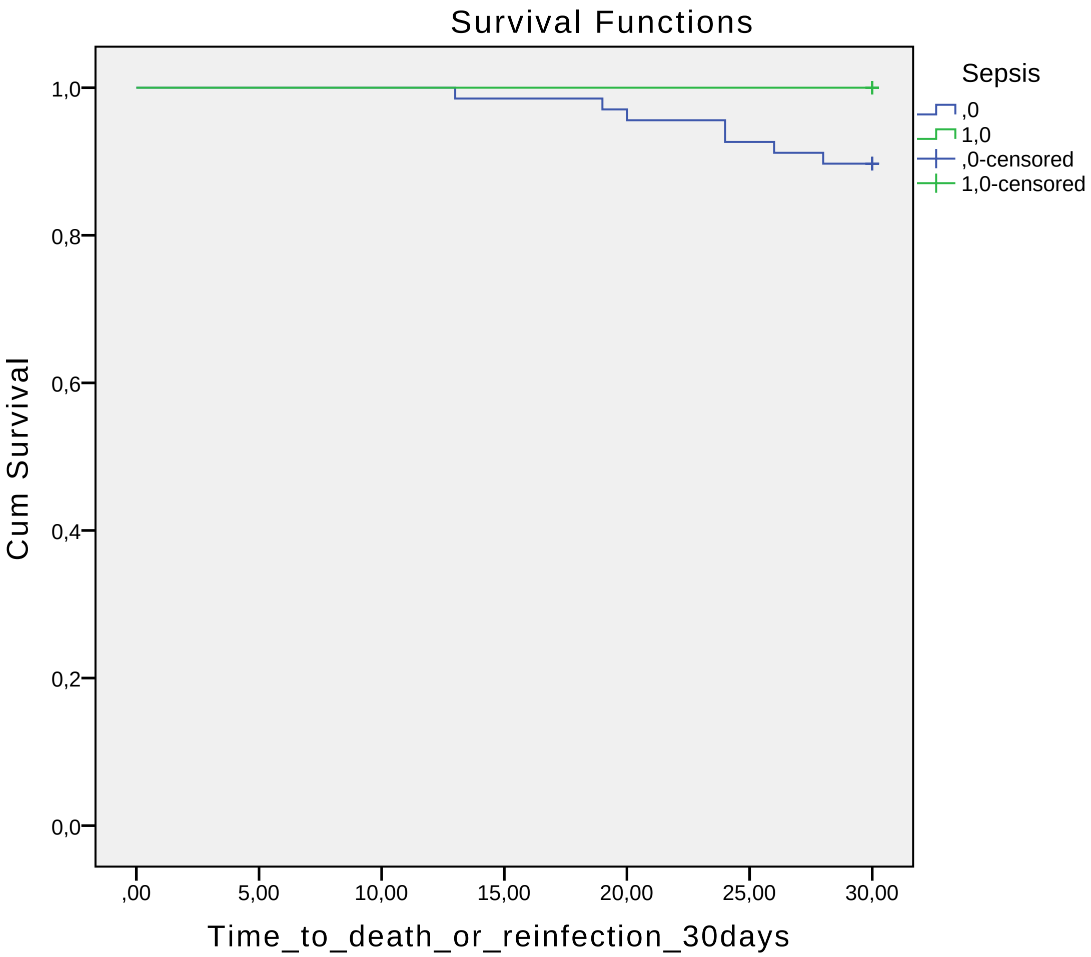

Kaplan-Meier

Warnings

No statistics are computed because all cases are censored.

Case Processing Summary

Septic_shock	Total N	N of Events	Censored	
			N	Percent
,0	74	7	67	90,5%
1,0	1	0	1	100,0%
Overall	75	7	68	90,7%

Overall Comparisons

	Chi-Square	df	Sig.
Log Rank (Mantel-Cox)	,099	1	,753
Breslow (Generalized Wilcoxon)	,099	1	,753

Test of equality of survival distributions for the different levels of Septic_shock.

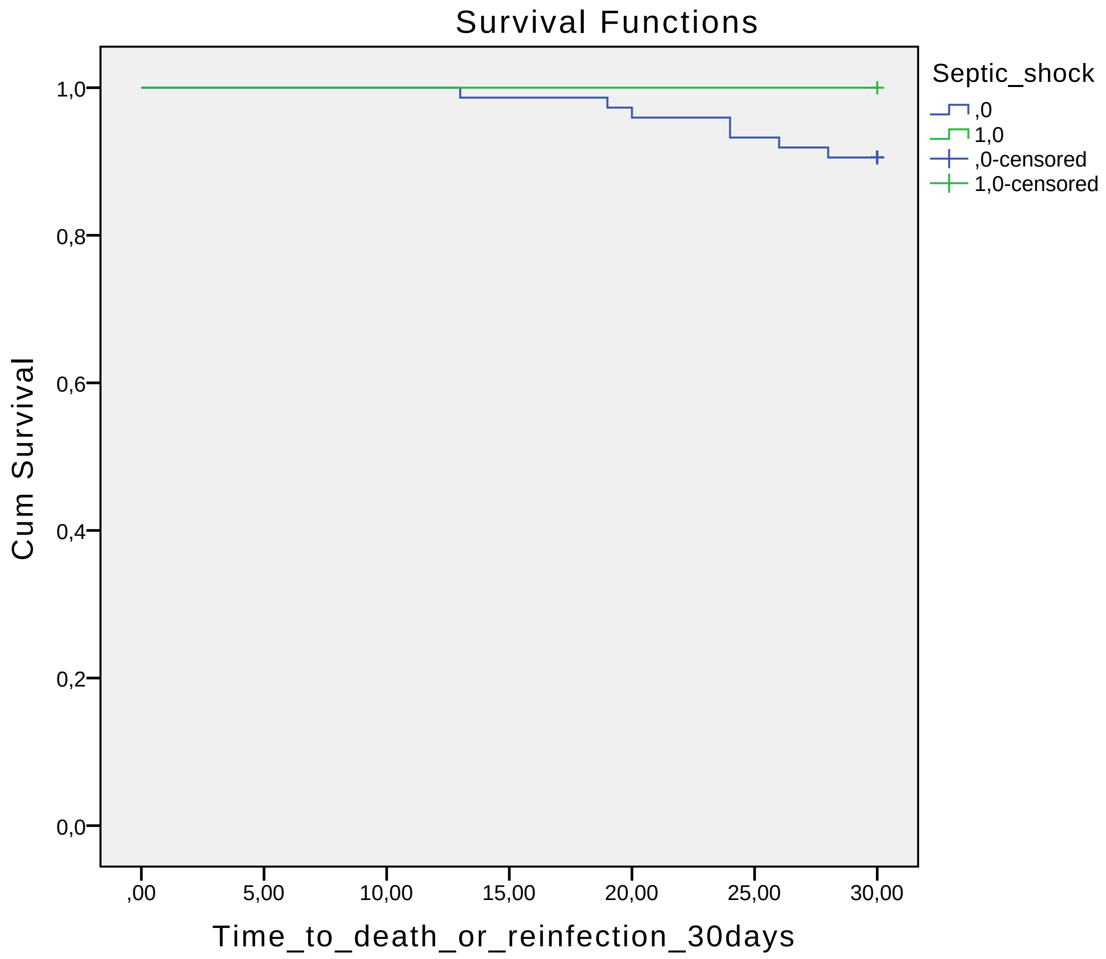

Kaplan-Meier

Case Processing Summary

Urological_abnormality	Total N	N of Events	Censored	
			N	Percent
,0	53	6	47	88,7%
1,0	22	1	21	95,5%
Overall	75	7	68	90,7%

Means and Medians for Survival Time

Urological_abnormality	Mean[a]				Median		
			95% Confidence Interval				95% …
	Estimate	Std. Error	Lower Bound	Upper Bound	Estimate	Std. Error	Lower Bound
,0	29,132	,406	28,337	29,927	.	.	.
1,0	29,545	,444	28,675	30,416	.	.	.
Overall	29,253	,316	28,635	29,872	.	.	.

Means and Medians for Survival Time

Urological_abnormality	Median
	95% …
	Upper Bound
,0	.
1,0	.
Overall	.

a. Estimation is limited to the largest survival time if it is censored.

Overall Comparisons

	Chi-Square	df	Sig.
Log Rank (Mantel-Cox)	,801	1	,371
Breslow (Generalized Wilcoxon)	,782	1	,376

Test of equality of survival distributions for the different levels of Urological_abnormality.

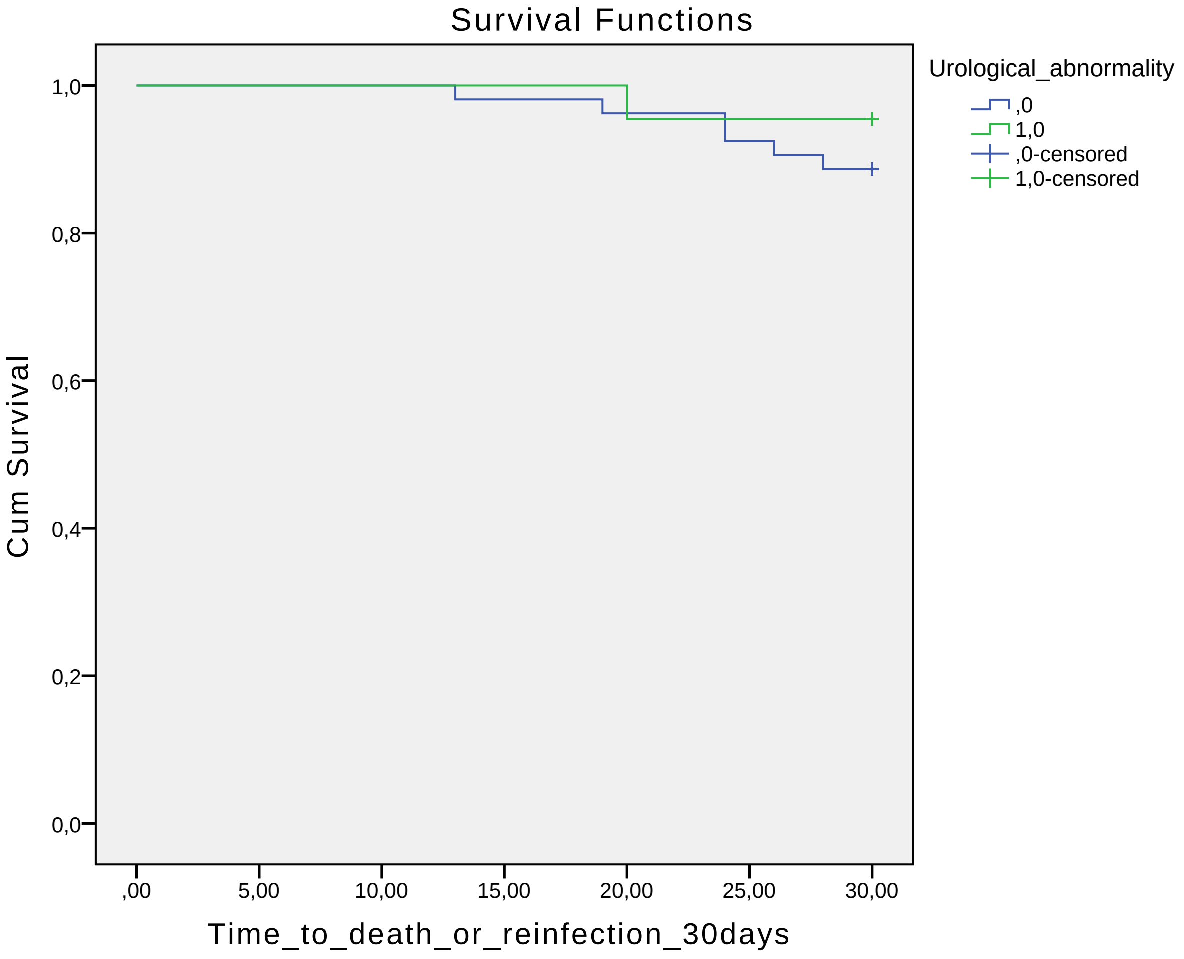

Kaplan-Meier

Case Processing Summary

Sex	Total N	N of Events	Censored	
			N	Percent
,0	32	3	29	90,6%
1,0	43	4	39	90,7%
Overall	75	7	68	90,7%

Means and Medians for Survival Time

Sex	Mean[a]				Median			
	Estimate	Std. Error	95% Confidence Interval		Estimate	Std. Error	95% Confidence Interval	
			Lower Bound	Upper Bound			Lower Bound	Upper Bound
,0	28,938	,633	27,697	30,178	.	.	.	.
1,0	29,488	,280	28,940	30,037	.	.	.	.
Overall	29,253	,316	28,635	29,872	.	.	.	.

a. Estimation is limited to the largest survival time if it is censored.

Overall Comparisons

	Chi-Square	df	Sig.
Log Rank (Mantel-Cox)	,003	1	,954
Breslow (Generalized Wilcoxon)	,011	1	,915

Test of equality of survival distributions for the different levels of Sex.

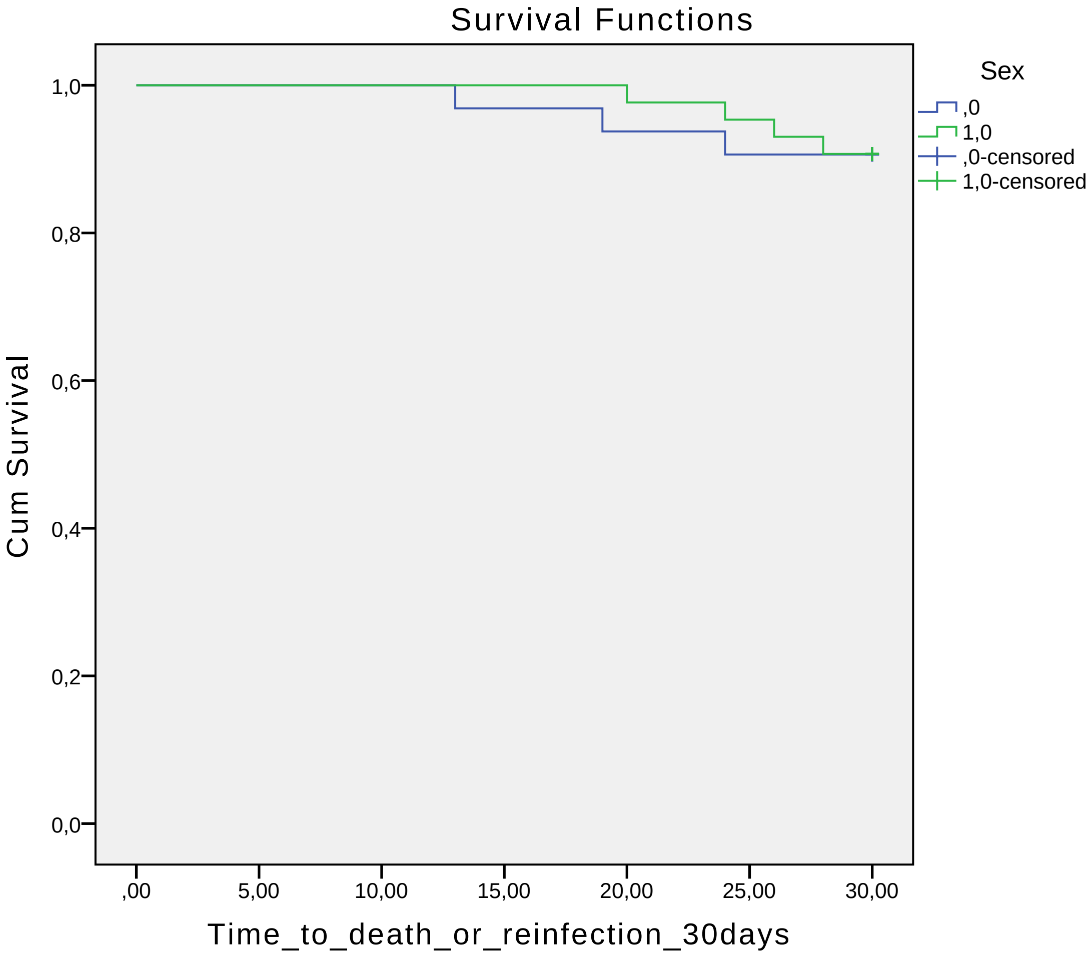

Kaplan-Meier

Case Processing Summary

Charlson_index_over_2	Total N	N of Events	Censored	
			N	Percent
,00	39	5	34	87,2%
1,00	36	2	34	94,4%
Overall	75	7	68	90,7%

Means and Medians for Survival Time

Charlson_index_over_2	Mean[a]				Median		
			95% Confidence Interval				95% ...
	Estimate	Std. Error	Lower Bound	Upper Bound	Estimate	Std. Error	Lower Bound
,00	28,949	,527	27,916	29,981	.	.	.
1,00	29,583	,318	28,961	30,206	.	.	.
Overall	29,253	,316	28,635	29,872	.	.	.

Means and Medians for Survival Time

Charlson_index_over_2	Median
	95% ...
	Upper Bound
,00	.
1,00	.
Overall	.

a. Estimation is limited to the largest survival time if it is censored.

Overall Comparisons

	Chi-Square	df	Sig.
Log Rank (Mantel-Cox)	1,143	1	,285
Breslow (Generalized Wilcoxon)	1,147	1	,284

Test of equality of survival distributions for the different levels of Charlson_index_over_2.

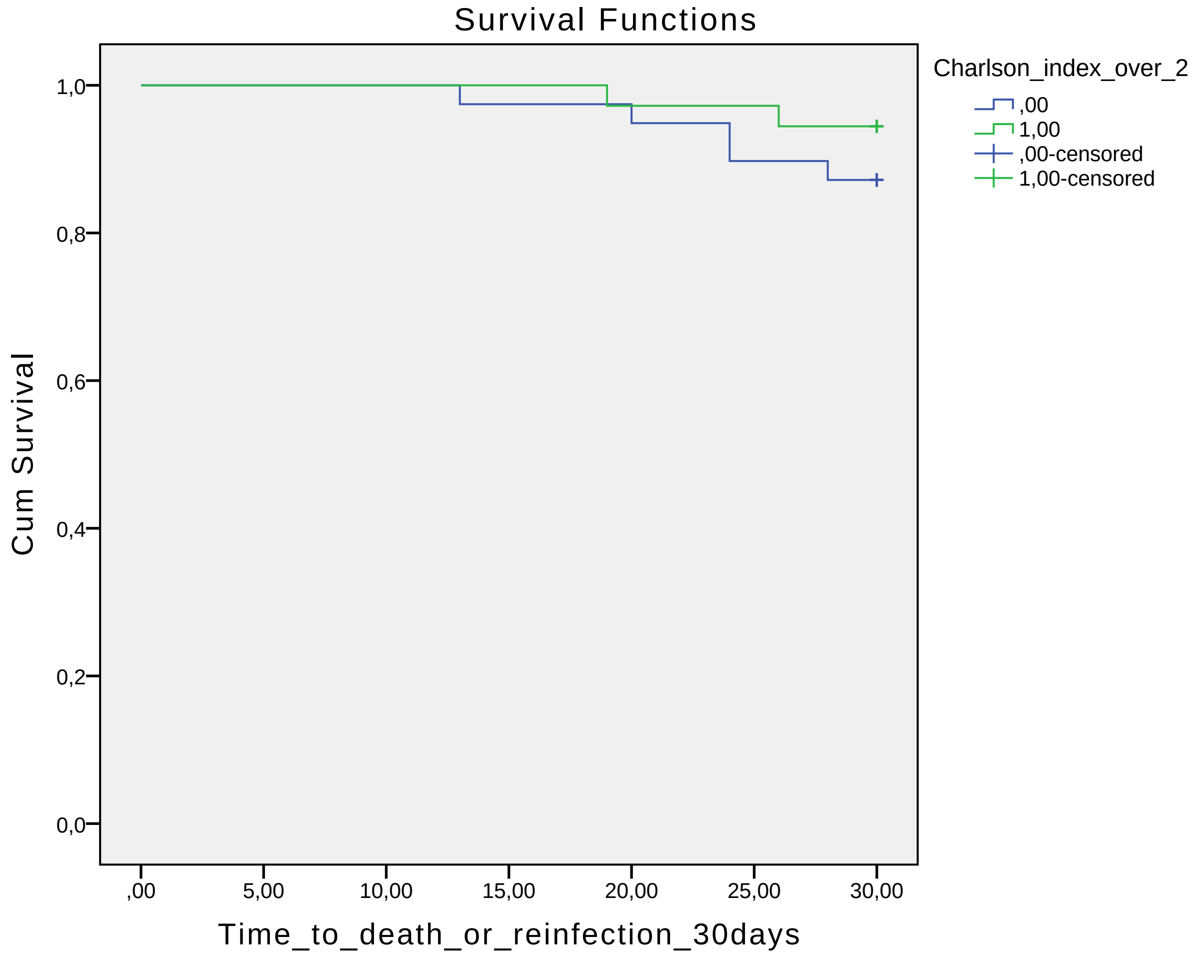

Kaplan-Meier

Case Processing Summary

Urinary_catheter	Total N	N of Events	Censored	
			N	Percent
0	59	4	55	93,2%
1	16	3	13	81,3%
Overall	75	7	68	90,7%

Means and Medians for Survival Time

Urinary_catheter	Mean[a]				Median			
	Estimate	Std. Error	95% Confidence Interval		Estimate	Std. Error	95% ...	
			Lower Bound	Upper Bound			Lower Bound	Upper Bound
0	29,627	,206	29,223	30,031	.	.	.	.
1	27,875	1,208	25,507	30,243	.	.	.	.
Overall	29,253	,316	28,635	29,872	.	.	.	.

Means and Medians for Survival Time

a. Estimation is limited to the largest survival time if it is censored.

Overall Comparisons

	Chi-Square	df	Sig.
Log Rank (Mantel-Cox)	2,419	1	,120
Breslow (Generalized Wilcoxon)	2,600	1	,107

Test of equality of survival distributions for the different levels of Urinary_catheter.

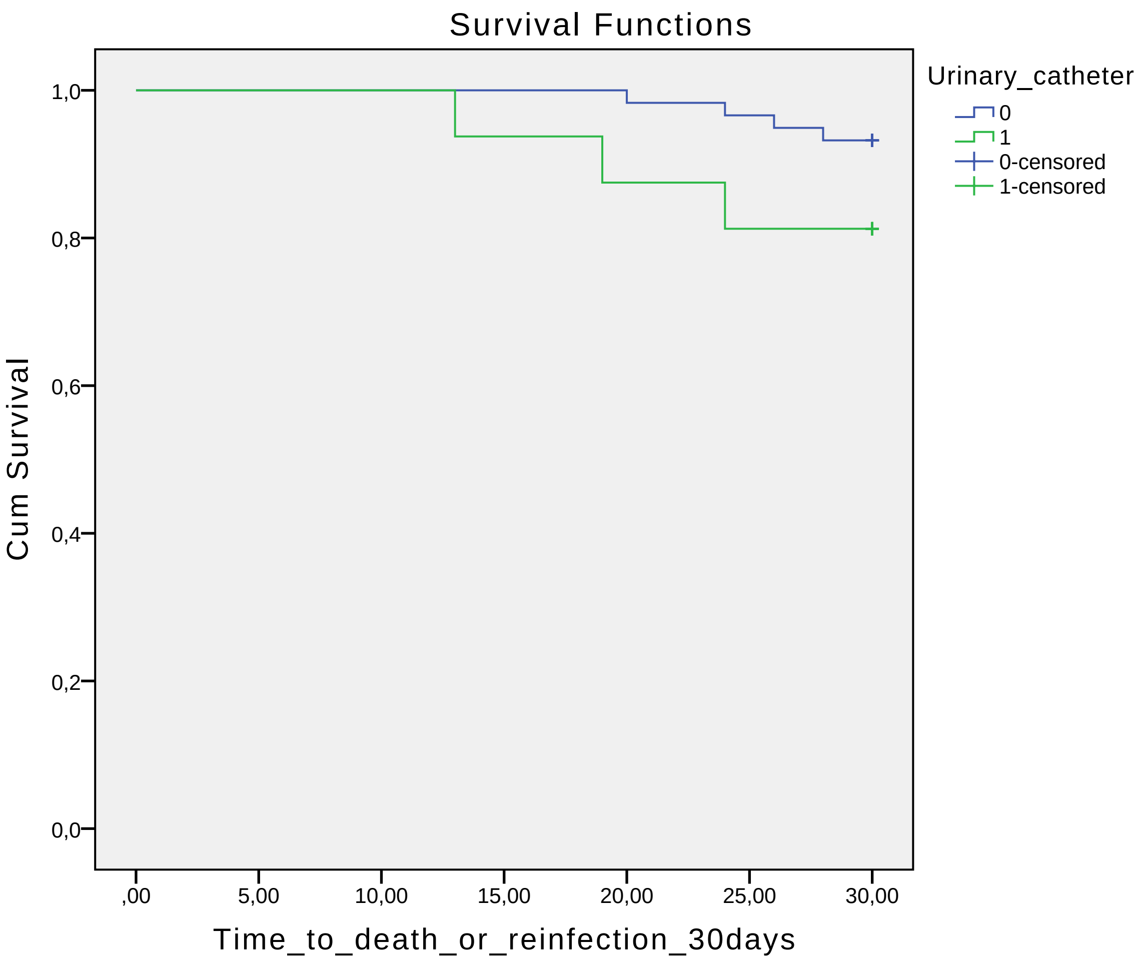

Kaplan-Meier

Case Processing Summary

Indwelling_urinary_catheter	Total N	N of Events	Censored	
			N	Percent
0	63	6	57	90,5%
1	12	1	11	91,7%
Overall	75	7	68	90,7%

Means and Medians for Survival Time

Indwelling_urinary_catheter	Mean[a]				Median		
			95% Confidence Interval				95% …
	Estimate	Std. Error	Lower Bound	Upper Bound	Estimate	Std. Error	Lower Bound
0	29,381	,270	28,852	29,910	.	.	.
1	28,583	1,356	25,925	31,242	.	.	.
Overall	29,253	,316	28,635	29,872	.	.	.

Means and Medians for Survival Time

Indwelling_urinary_catheter	Median
	95% …
	Upper Bound
0	.
1	.
Overall	.

a. Estimation is limited to the largest survival time if it is censored.

Overall Comparisons

	Chi-Square	df	Sig.
Log Rank (Mantel-Cox)	,007	1	,932
Breslow (Generalized Wilcoxon)	,002	1	,965

Test of equality of survival distributions for the different levels of Indwelling_urinary_catheter.

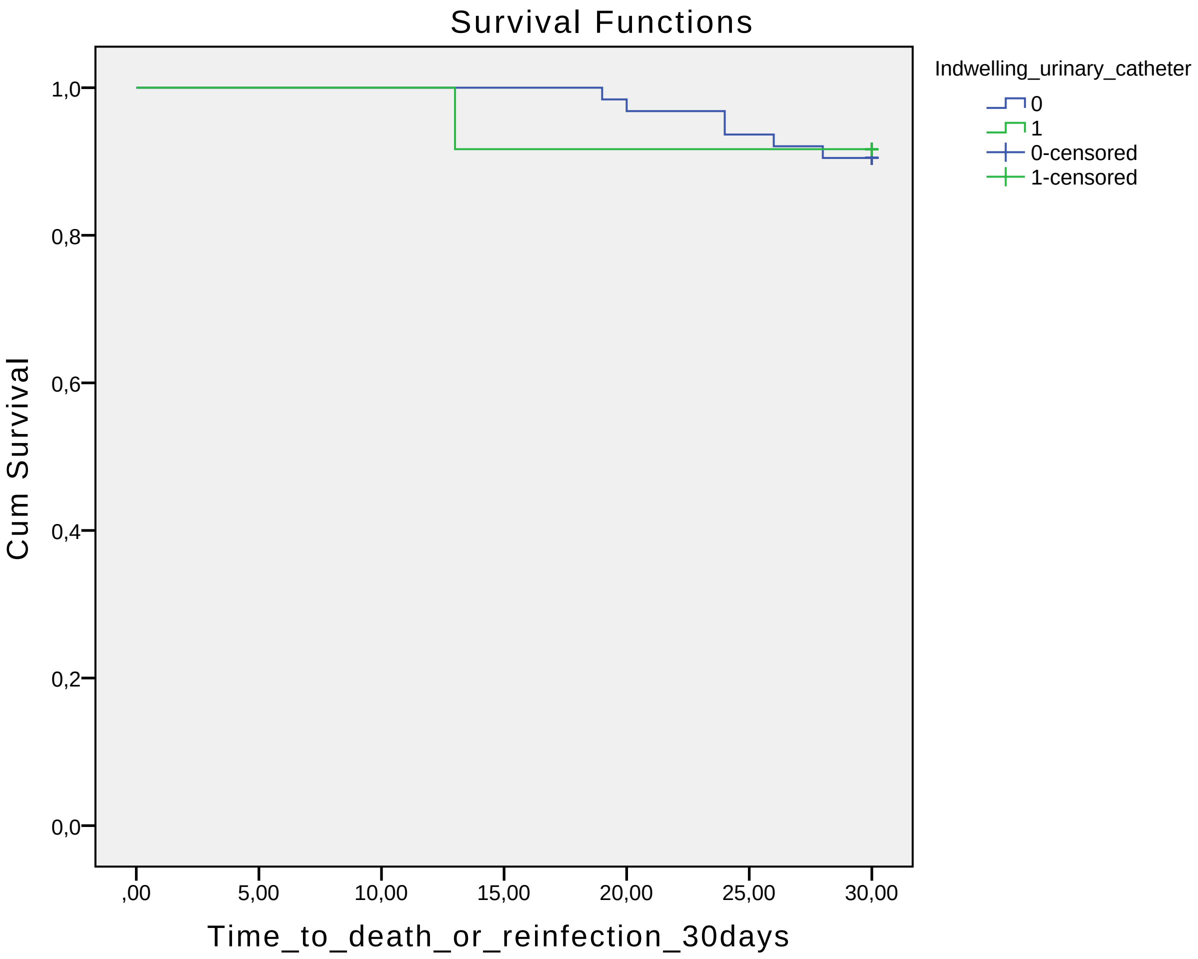

Kaplan-Meier

Case Processing Summary

Cognitive_impairment	Total N	N of Events	Censored	
			N	Percent
0	56	5	51	91,1%
1	19	2	17	89,5%
Overall	75	7	68	90,7%

Means and Medians for Survival Time

Cognitive_impairment	Mean[a]				Median		
			95% Confidence Interval				95% …
	Estimate	Std. Error	Lower Bound	Upper Bound	Estimate	Std. Error	Lower Bound
0	29,339	,301	28,749	29,930	.	.	.
1	29,000	,871	27,292	30,708	.	.	.
Overall	29,253	,316	28,635	29,872	.	.	.

Means and Medians for Survival Time

Cognitive_impairment	Median
	95% …
	Upper Bound
0	.
1	.
Overall	.

a. Estimation is limited to the largest survival time if it is censored.

Overall Comparisons

	Chi-Square	df	Sig.
Log Rank (Mantel-Cox)	,043	1	,835
Breslow (Generalized Wilcoxon)	,042	1	,837

Test of equality of survival distributions for the different levels of Cognitive_impairment.

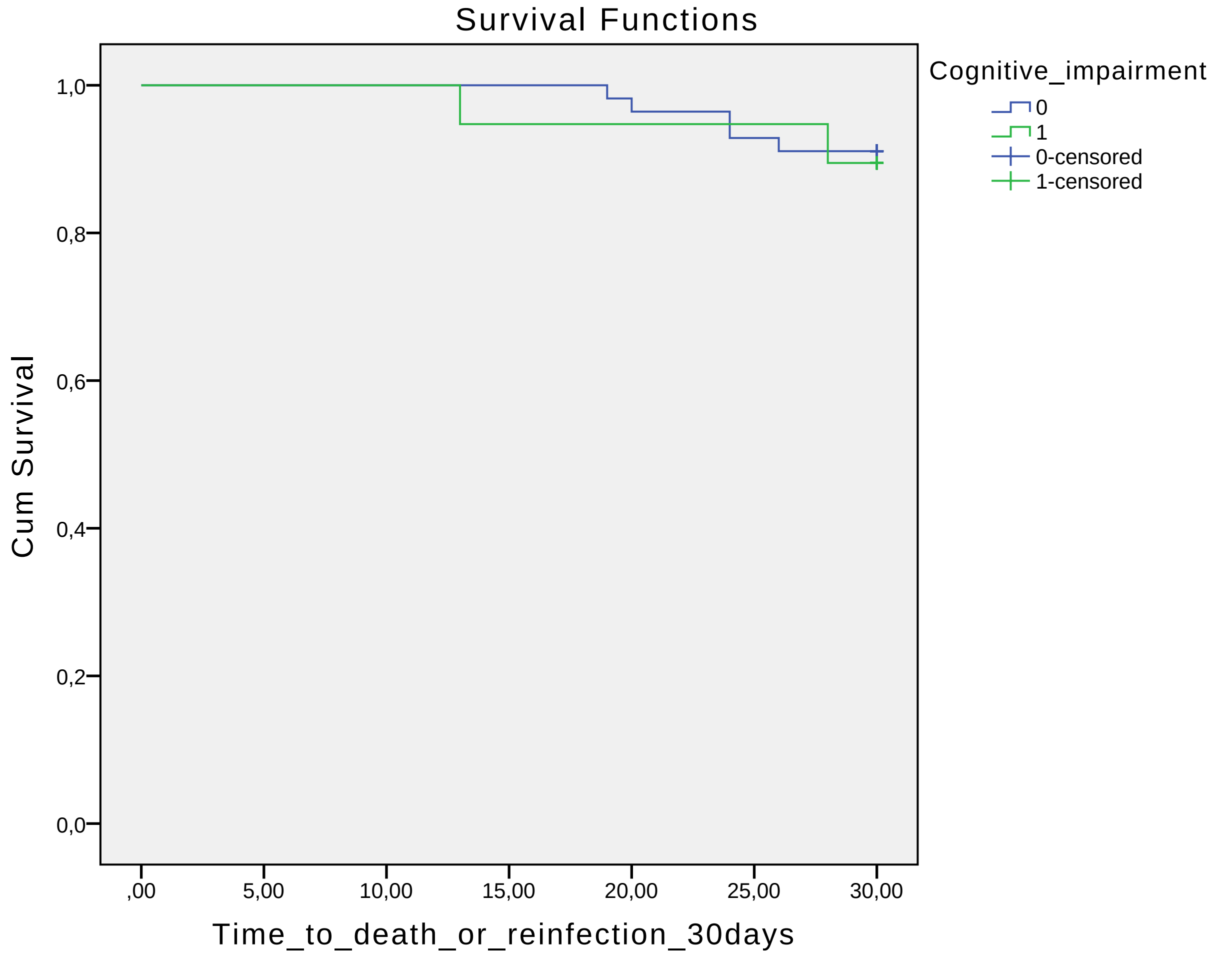

Kaplan-Meier

Case Processing Summary

Solid_tumor	Total N	N of Events	Censored	
			N	Percent
0	65	6	59	90,8%
2	10	1	9	90,0%
Overall	75	7	68	90,7%

Means and Medians for Survival Time

Solid_tumor	Mean[a]				Median			
	Estimate	Std. Error	95% Confidence Interval		Estimate	Std. Error	95% Confidence Interval	
			Lower Bound	Upper Bound			Lower Bound	Upper Bound
0	29,308	,326	28,668	29,947	.	.	.	.
2	28,900	1,044	26,855	30,945	.	.	.	.
Overall	29,253	,316	28,635	29,872	.	.	.	.

a. Estimation is limited to the largest survival time if it is censored.

Overall Comparisons

	Chi-Square	df	Sig.
Log Rank (Mantel-Cox)	,013	1	,911
Breslow (Generalized Wilcoxon)	,020	1	,888

Test of equality of survival distributions for the different levels of Solid_tumor.

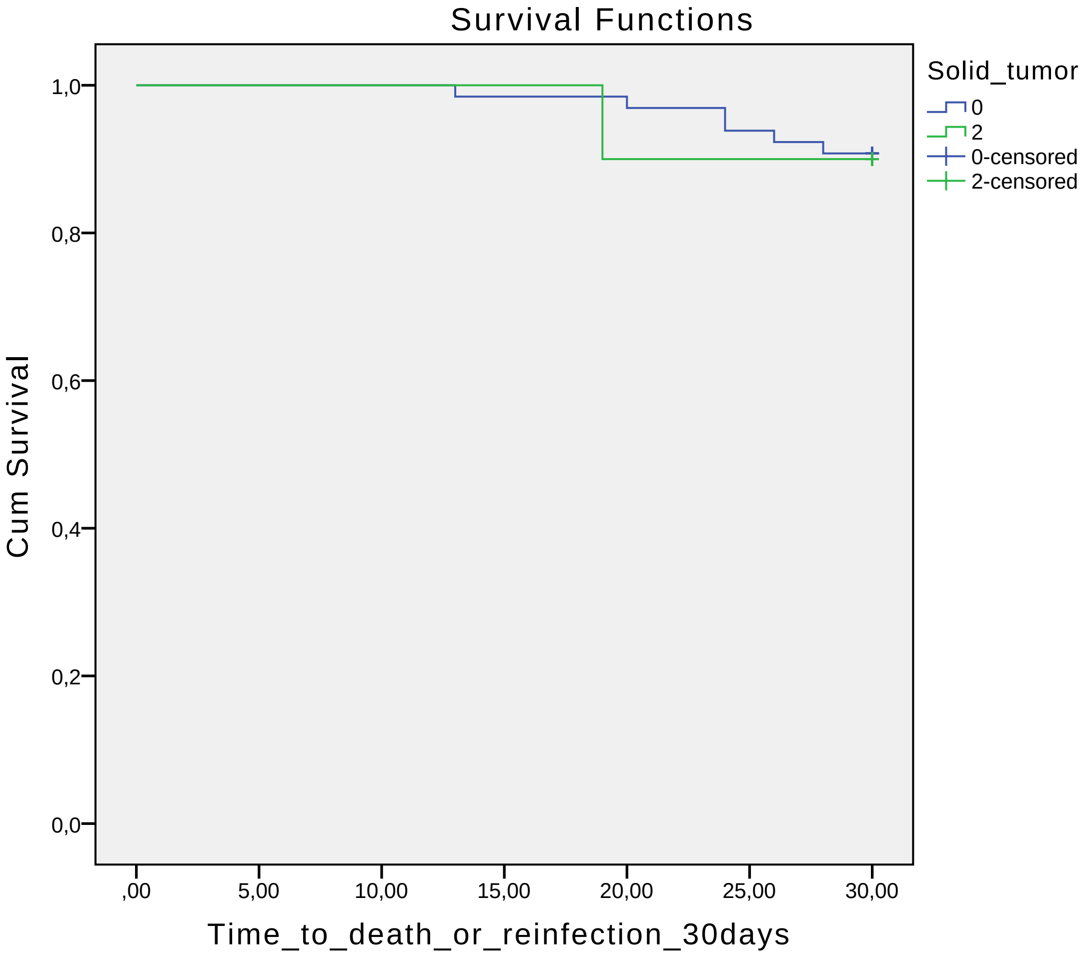

Kaplan-Meier

Case Processing Summary

Metastatic_solid_tumor	Total N	N of Events	Censored	
			N	Percent
0	73	6	67	91,8%
6	2	1	1	50,0%
Overall	75	7	68	90,7%

Means and Medians for Survival Time

Metastatic_solid_tumor	Mean[a]				Median		
			95% Confidence Interval				95% ...
	Estimate	Std. Error	Lower Bound	Upper Bound	Estimate	Std. Error	Lower Bound
0	29,288	,321	28,659	29,917	.	.	.
6	28,000	1,414	25,228	30,772	26,000	.	.
Overall	29,253	,316	28,635	29,872	.	.	.

Means and Medians for Survival Time

Metastatic_solid_tumor	Median
	95% ...
	Upper Bound
0	.
6	.
Overall	.

a. Estimation is limited to the largest survival time if it is censored.

Overall Comparisons

	Chi-Square	df	Sig.
Log Rank (Mantel-Cox)	3,863	1	,049
Breslow (Generalized Wilcoxon)	3,562	1	,059

Test of equality of survival distributions for the different levels of Metastatic_solid_tumor.

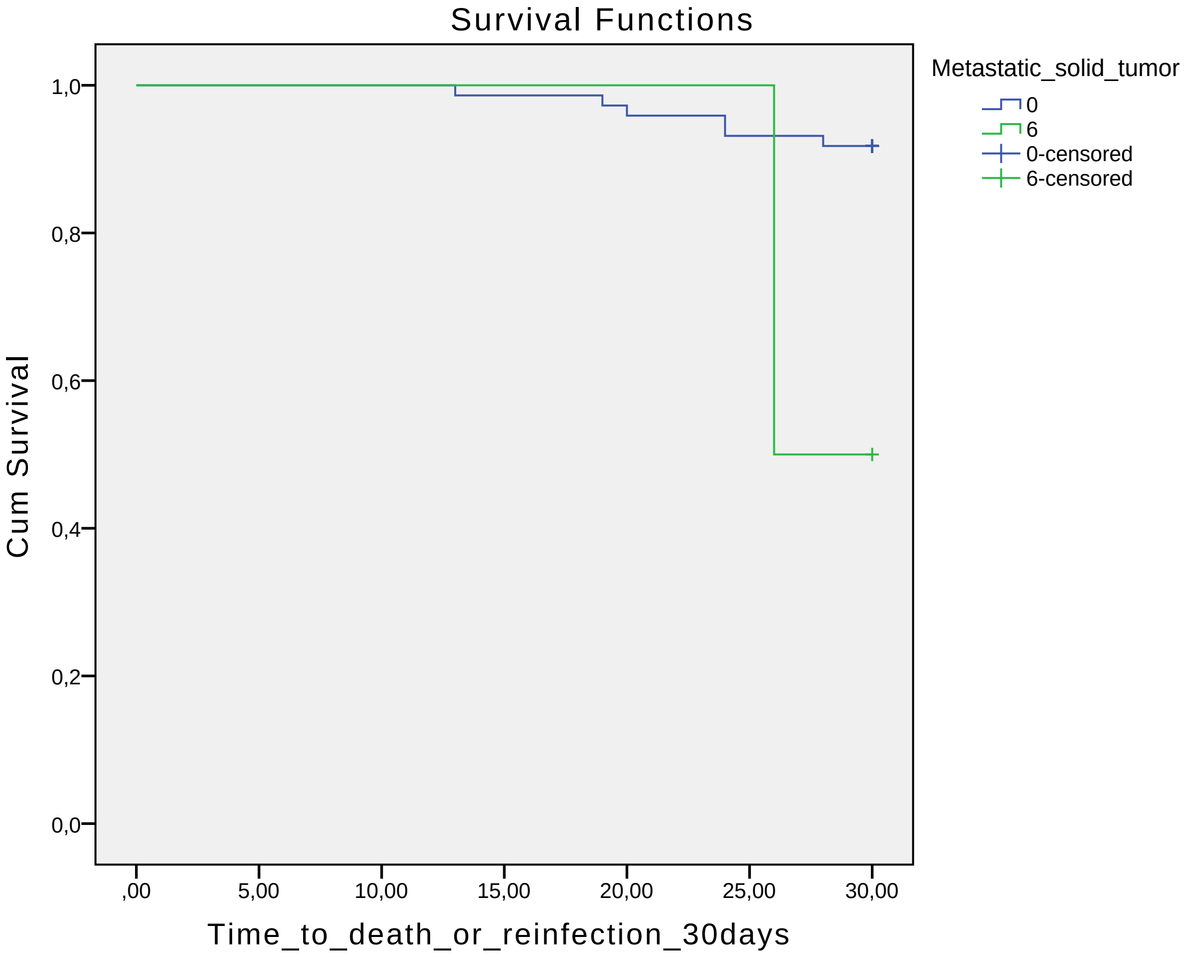

Kaplan-Meier

Case Processing Summary

Lymphoma	Total N	N of Events	Censored	
			N	Percent
0	73	6	67	91,8%
2	2	1	1	50,0%
Overall	75	7	68	90,7%

Means and Medians for Survival Time

Lymphoma	Mean[a]				Median			
	Estimate	Std. Error	95% Confidence Interval		Estimate	Std. Error	95% Confidence Interval	
			Lower Bound	Upper Bound			Lower Bound	Upper Bound
0	29,288	,321	28,659	29,917	.	.	.	.
2	28,000	1,414	25,228	30,772	26,000	.	.	.
Overall	29,253	,316	28,635	29,872	.	.	.	.

a. Estimation is limited to the largest survival time if it is censored.

Overall Comparisons

	Chi-Square	df	Sig.
Log Rank (Mantel-Cox)	3,863	1	,049
Breslow (Generalized Wilcoxon)	3,562	1	,059

Test of equality of survival distributions for the different levels of Lymphoma.

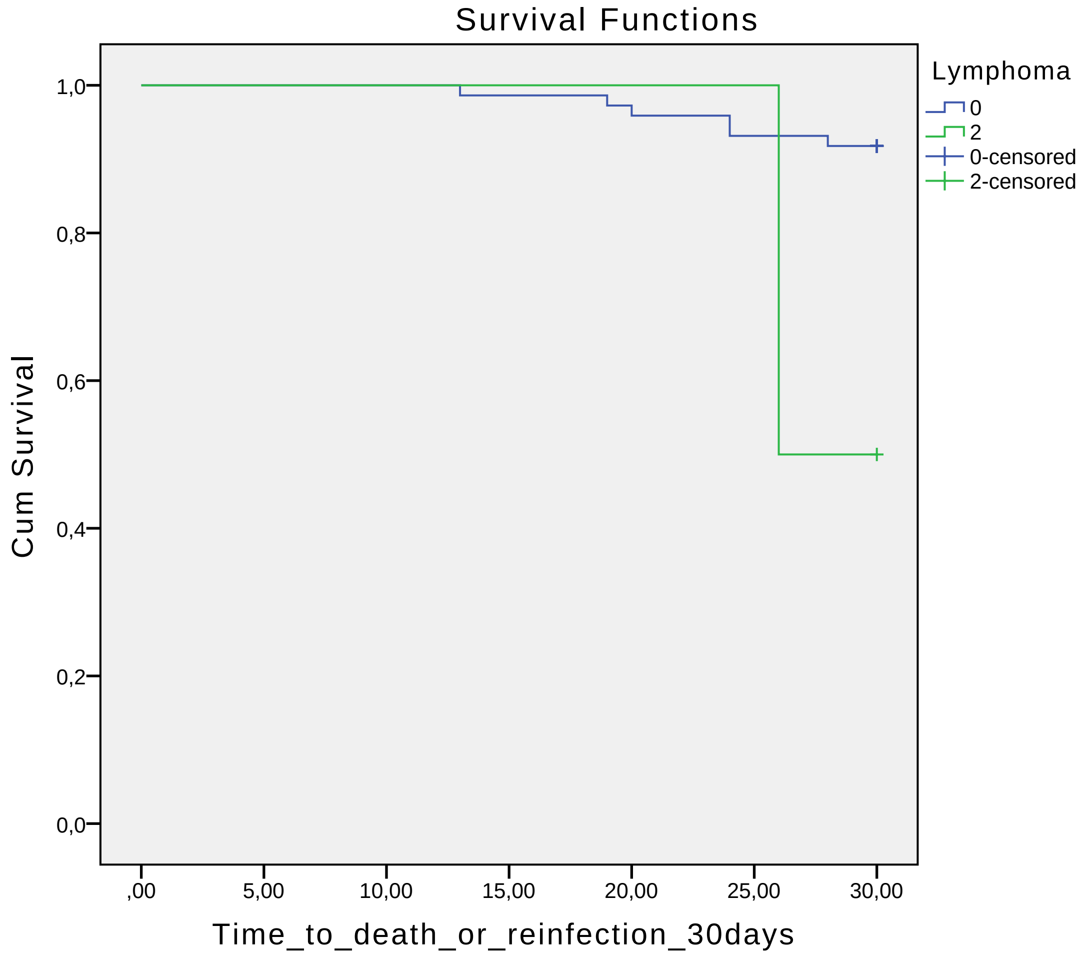

Kaplan-Meier

Warnings

No comparison analysis is performed because the factor variable has only one value for every stratum.

Case Processing Summary

Leukemia	Total N	N of Events	Censored	
			N	Percent
0	75	7	68	90,7%
Overall	75	7	68	90,7%

Means and Medians for Survival Time

Leukemia	Mean[a]				Median			
	Estimate	Std. Error	95% Confidence Interval		Estimate	Std. Error	95% Confidence Interval	
			Lower Bound	Upper Bound			Lower Bound	Upper Bound
0	29,253	,316	28,635	29,872	.	.	.	.
Overall	29,253	,316	28,635	29,872	.	.	.	.

a. Estimation is limited to the largest survival time if it is censored.

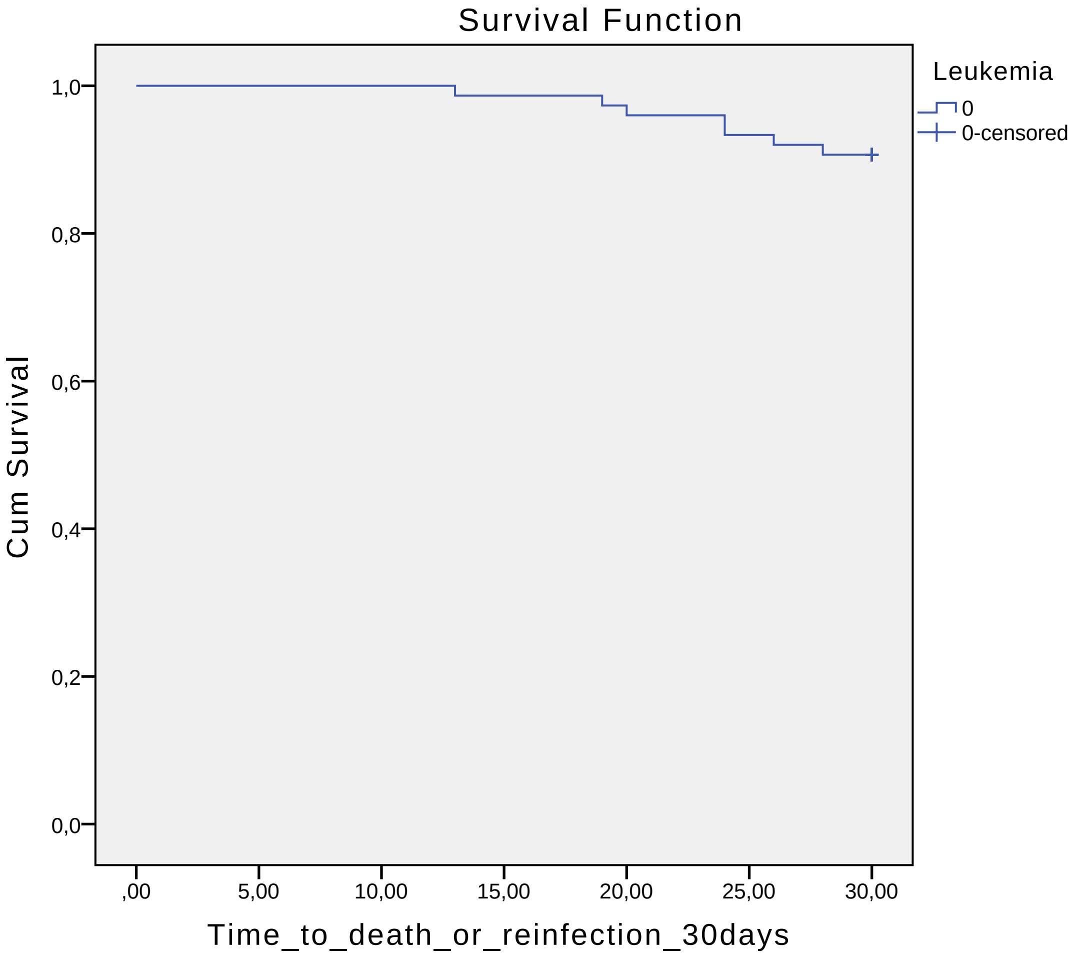

Kaplan-Meier

Case Processing Summary

Chronic_liver_disease	Total N	N of Events	Censored	
			N	Percent
0	67	6	61	91,0%
1	8	1	7	87,5%
Overall	75	7	68	90,7%

Means and Medians for Survival Time

Chronic_liver_disease	Mean[a]				Median		
			95% Confidence Interval				95% ...
	Estimate	Std. Error	Lower Bound	Upper Bound	Estimate	Std. Error	Lower Bound
0	29,254	,343	28,581	29,926	.	.	.
1	29,250	,702	27,875	30,625	.	.	.
Overall	29,253	,316	28,635	29,872	.	.	.

Means and Medians for Survival Time

Chronic_liver_disease	Median
	95% ...
	Upper Bound
0	.
1	.
Overall	.

a. Estimation is limited to the largest survival time if it is censored.

Overall Comparisons

	Chi-Square	df	Sig.
Log Rank (Mantel-Cox)	,095	1	,757
Breslow (Generalized Wilcoxon)	,093	1	,760

Test of equality of survival distributions for the different levels of Chronic_liver_disease.

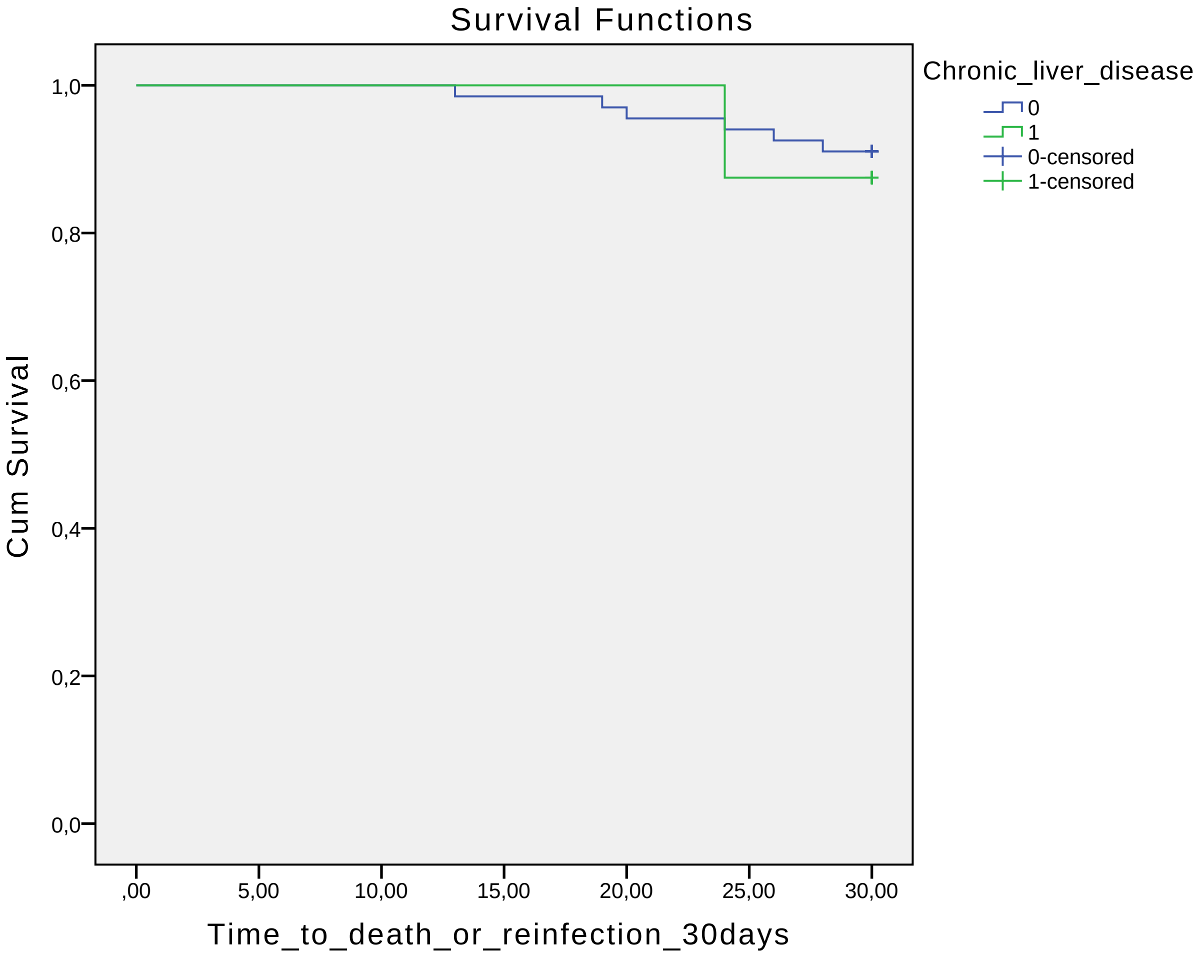

Kaplan-Meier

Case Processing Summary

Chronic_liver_disease_without_portal_hypertension	Total N	N of Events	Censored	
			N	Percent
0	69	6	63	91,3%
1	6	1	5	83,3%
Overall	75	7	68	90,7%

Means and Medians for Survival Time

Chronic_liver_disease_without_portal_hypertension	Mean[a]				Median		
			95% Confidence Interval				95% …
	Estimate	Std. Error	Lower Bound	Upper Bound	Estimate	Std. Error	Lower Bound
0	29,275	,334	28,622	29,929	.	.	.
1	29,000	,913	27,211	30,789	.	.	.
Overall	29,253	,316	28,635	29,872	.	.	.

Means and Medians for Survival Time

Chronic_liver_disease_without_portal_hypertension	Median
	95% …
	Upper Bound
0	.
1	.
Overall	.

a. Estimation is limited to the largest survival time if it is censored.

Overall Comparisons

	Chi-Square	df	Sig.
Log Rank (Mantel-Cox)	,392	1	,531
Breslow (Generalized Wilcoxon)	,386	1	,534

Test of equality of survival distributions for the different levels of Chronic_liver_disease_without_portal_hypertension.

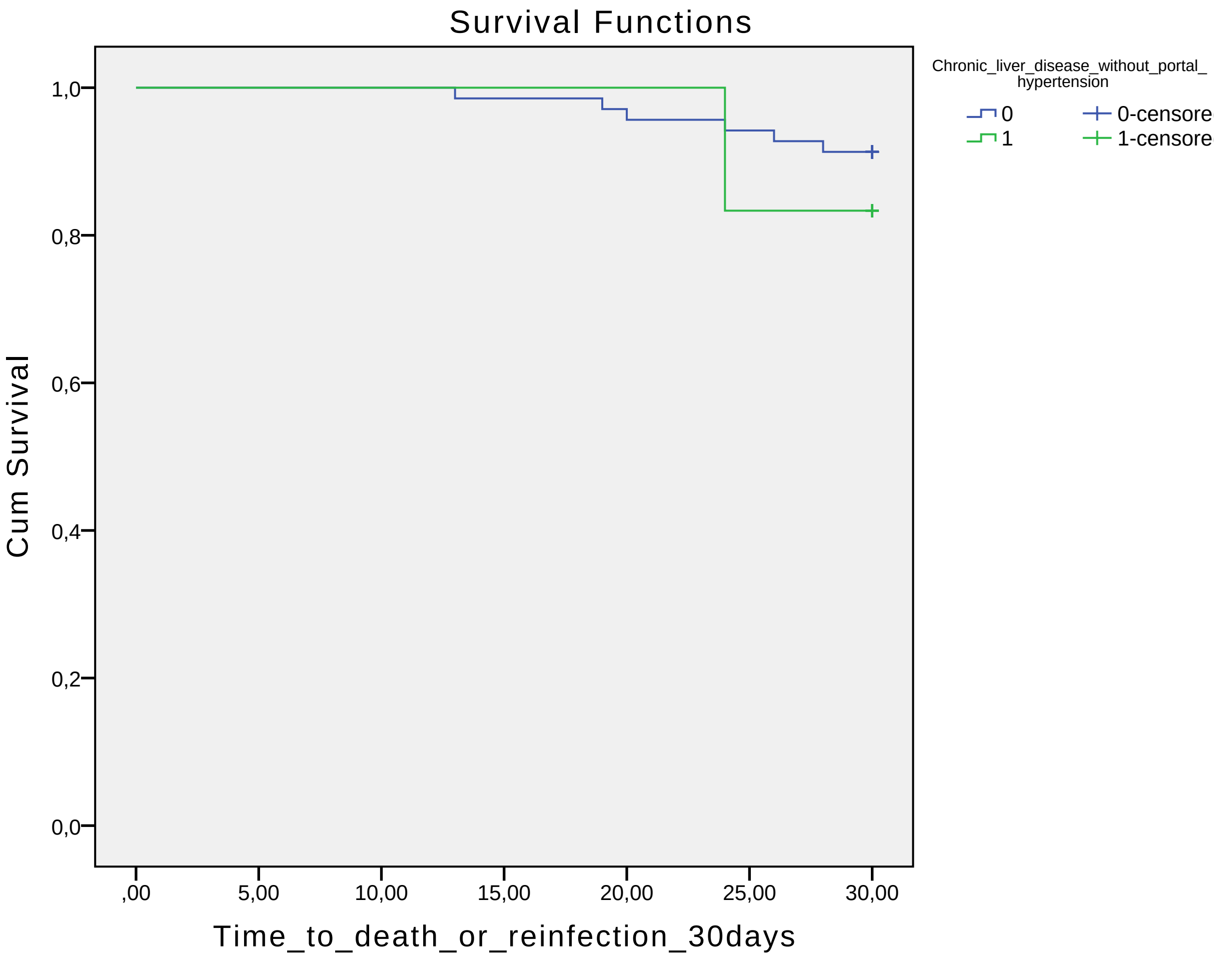

Kaplan-Meier

Warnings

No statistics are computed because all cases are censored.

Case Processing Summary

Chronic_liver_disease_with_portal_hypertension	Total N	N of Events	Censored	
			N	Percent
0	73	7	66	90,4%
3	2	0	2	100,0%
Overall	75	7	68	90,7%

Overall Comparisons

	Chi-Square	df	Sig.
Log Rank (Mantel-Cox)	,201	1	,654
Breslow (Generalized Wilcoxon)	,200	1	,654

Test of equality of survival distributions for the different levels of Chronic_liver_disease_with_portal_hypertension.

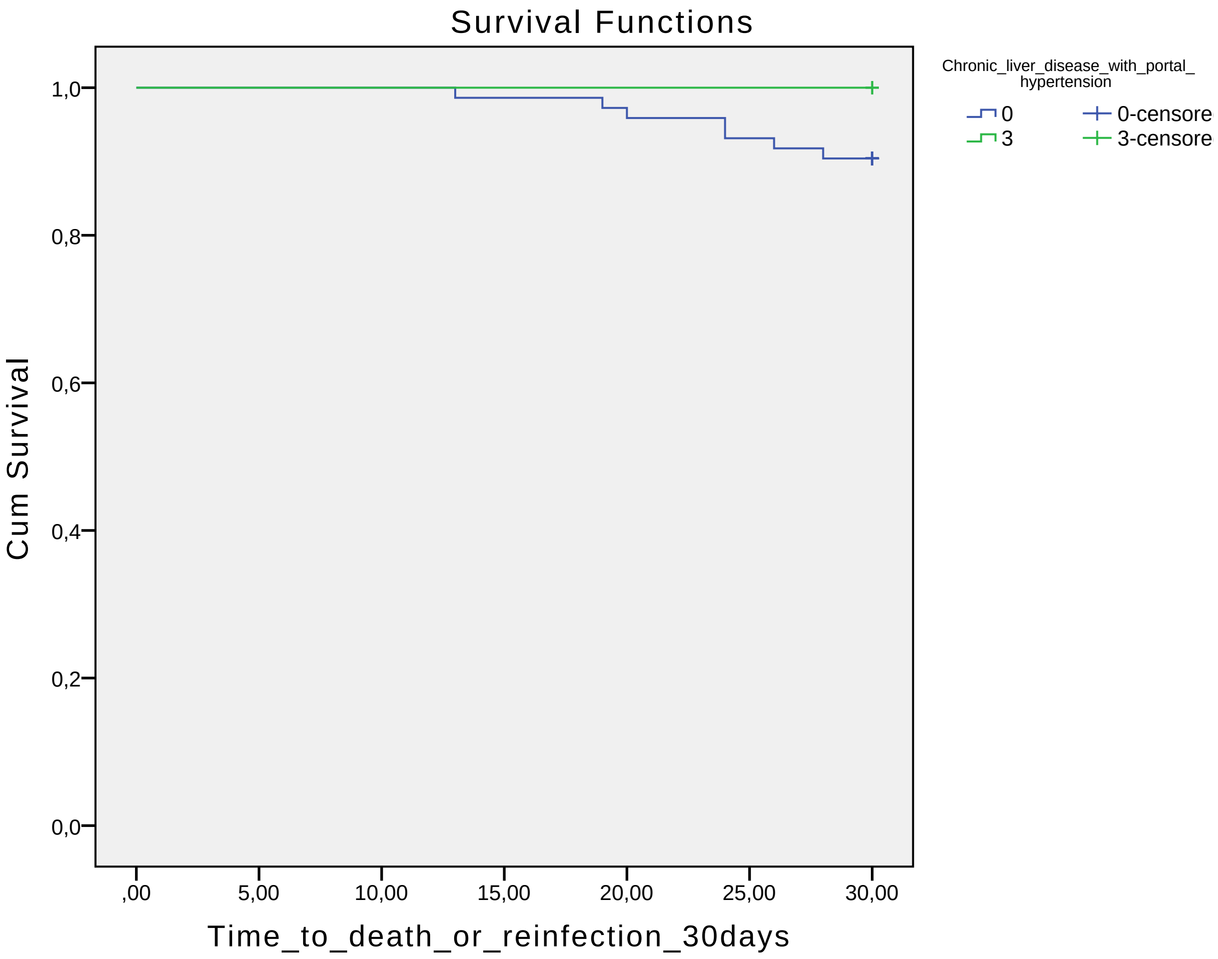

Kaplan-Meier

Warnings

No statistics are computed because all cases are censored.

Case Processing Summary

Connective_tissue_disease	Total N	N of Events	Censored	
			N	Percent
0	71	7	64	90,1%
1	4	0	4	100,0%
Overall	75	7	68	90,7%

Overall Comparisons

	Chi-Square	df	Sig.
Log Rank (Mantel-Cox)	,413	1	,520
Breslow (Generalized Wilcoxon)	,413	1	,521

Test of equality of survival distributions for the different levels of Connective_tissue_disease.

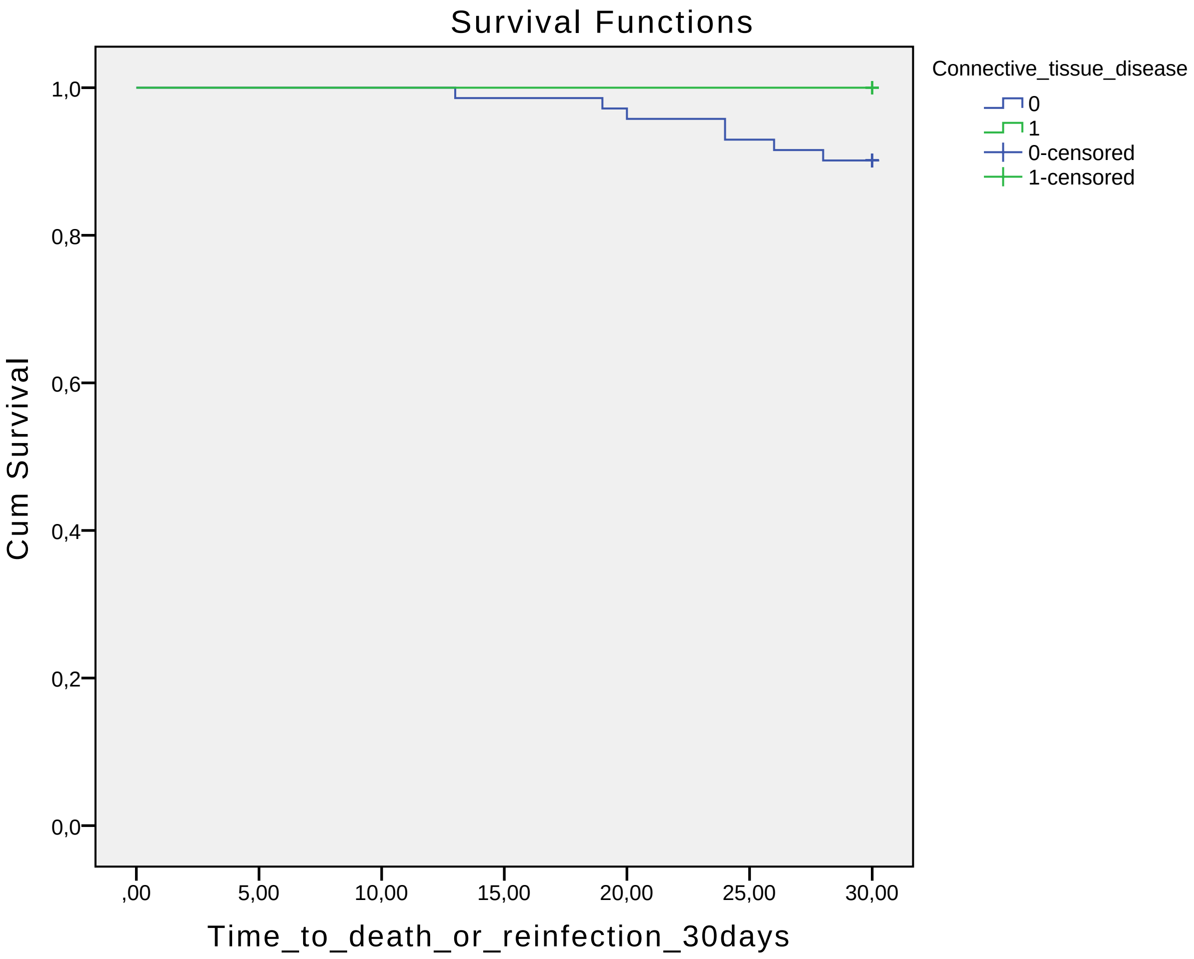

Kaplan-Meier

Case Processing Summary

Diabetes_mellitus	Total N	N of Events	Censored	
			N	Percent
0	51	6	45	88,2%
1	24	1	23	95,8%
Overall	75	7	68	90,7%

Means and Medians for Survival Time

Diabetes_mellitus	Mean[a]				Median		
	Estimate	Std. Error	95% Confidence Interval		Estimate	Std. Error	95% …
			Lower Bound	Upper Bound			Lower Bound
0	28,941	,456	28,047	29,835	.	.	.
1	29,917	,082	29,757	30,077	.	.	.
Overall	29,253	,316	28,635	29,872	.	.	.

Means and Medians for Survival Time

Diabetes_mellitus	Median
	95% …
	Upper Bound
0	.
1	.
Overall	.

a. Estimation is limited to the largest survival time if it is censored.

Overall Comparisons

	Chi-Square	df	Sig.
Log Rank (Mantel-Cox)	1,144	1	,285
Breslow (Generalized Wilcoxon)	1,217	1	,270

Test of equality of survival distributions for the different levels of Diabetes_mellitus.

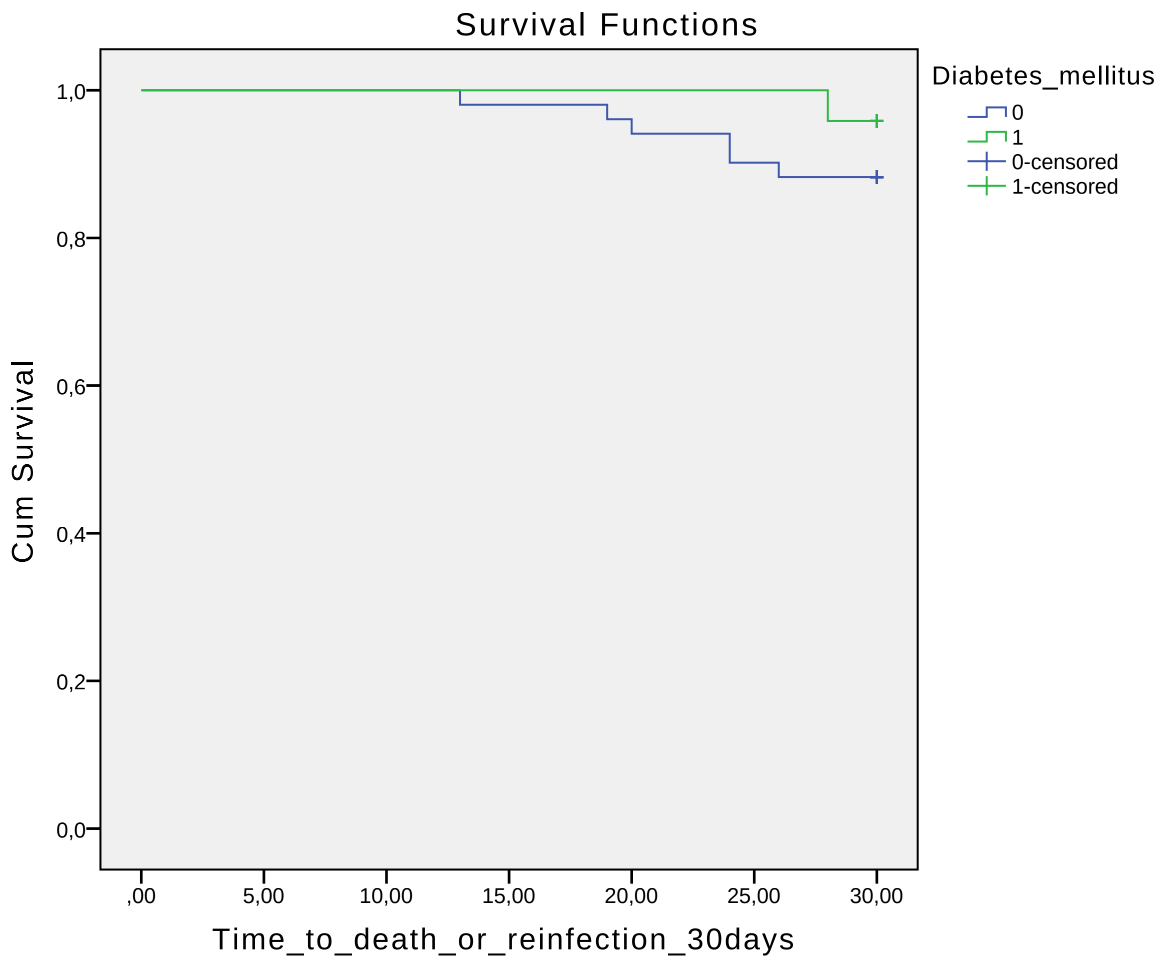

Kaplan-Meier

Case Processing Summary

DM_without_target_organ_damage	Total N	N of Events	Censored	
			N	Percent
0	60	6	54	90,0%
1	15	1	14	93,3%
Overall	75	7	68	90,7%

Means and Medians for Survival Time

DM_without_target_organ_damage	Mean[a]				Median		
			95% Confidence Interval				95% …
	Estimate	Std. Error	Lower Bound	Upper Bound	Estimate	Std. Error	Lower Bound
0	29,100	,391	28,334	29,866	.	.	.
1	29,867	,129	29,614	30,119	.	.	.
Overall	29,253	,316	28,635	29,872	.	.	.

Means and Medians for Survival Time

DM_without_target_organ_damage	Median
	95% ...
	Upper Bound
0	.
1	.
Overall	.

a. Estimation is limited to the largest survival time if it is censored.

Overall Comparisons

	Chi-Square	df	Sig.
Log Rank (Mantel-Cox)	,181	1	,670
Breslow (Generalized Wilcoxon)	,217	1	,641

Test of equality of survival distributions for the different levels of DM_without_target_organ_damage.

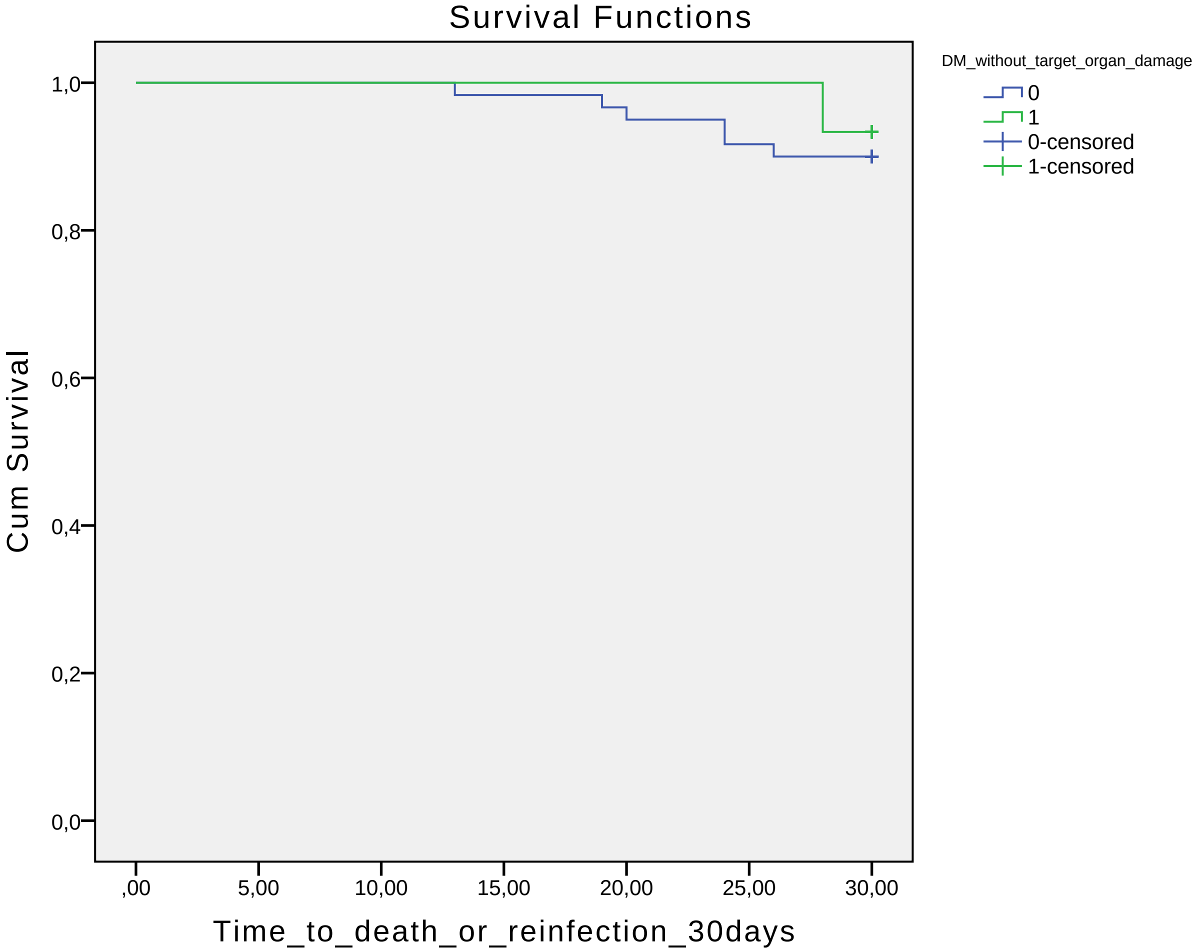

Kaplan-Meier

Warnings

No statistics are computed because all cases are censored.

Case Processing Summary

DM_with_target_organ_damage	Total N	N of Events	Censored	
			N	Percent
0	67	7	60	89,6%
2	8	0	8	100,0%
Overall	75	7	68	90,7%

Overall Comparisons

	Chi-Square	df	Sig.
Log Rank (Mantel-Cox)	,877	1	,349
Breslow (Generalized Wilcoxon)	,877	1	,349

Test of equality of survival distributions for the different levels of DM_with_target_organ_damage.

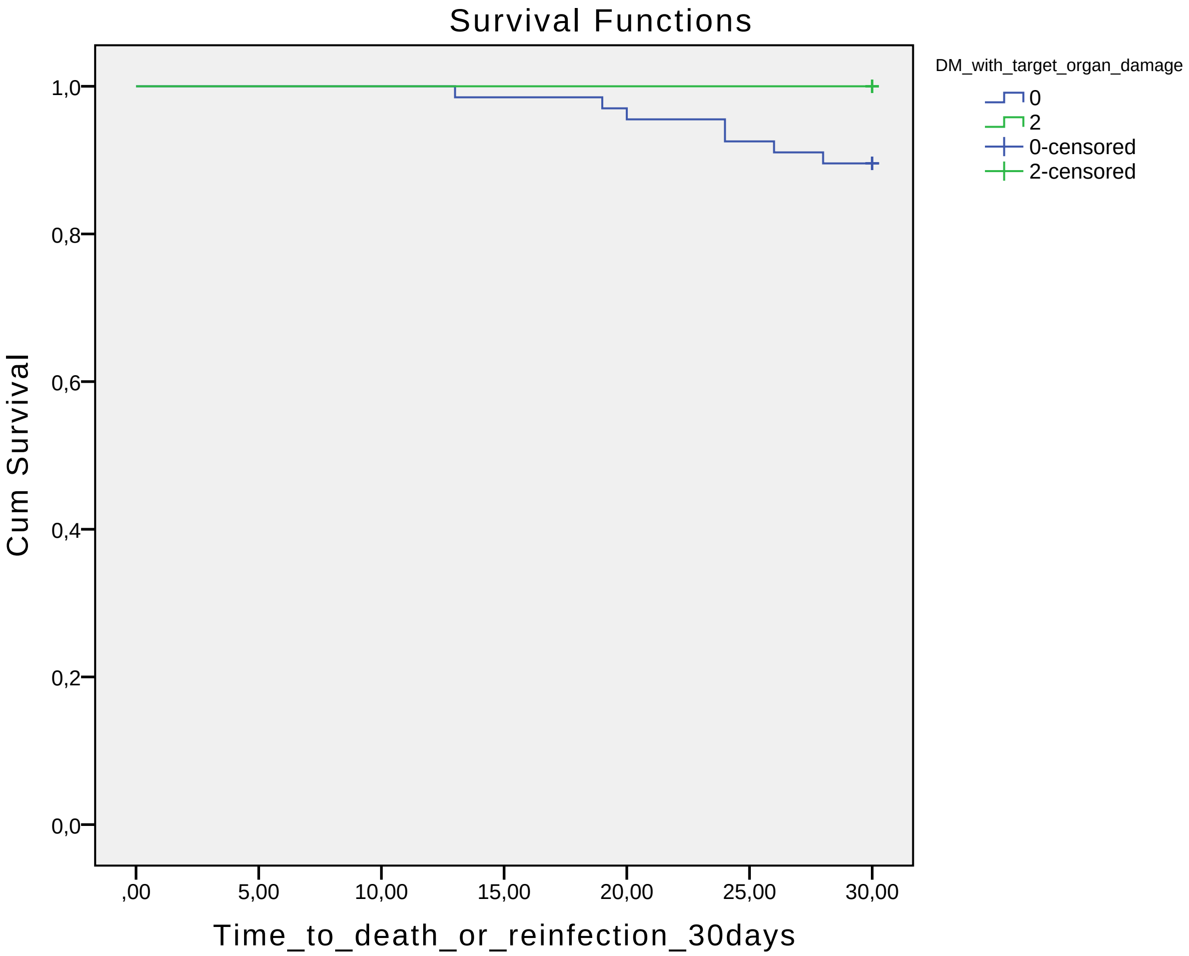

Kaplan-Meier

Case Processing Summary

Hypertension	Total N	N of Events	Censored	
			N	Percent
0	31	4	27	87,1%
1	44	3	41	93,2%
Overall	75	7	68	90,7%

Means and Medians for Survival Time

Hypertension	Mean[a]				Median		
	Estimate	Std. Error	95% Confidence Interval		Estimate	Std. Error	95% …
			Lower Bound	Upper Bound			Lower Bound
0	28,581	,709	27,190	29,971	.	.	.
1	29,727	,165	29,404	30,051	.	.	.
Overall	29,253	,316	28,635	29,872	.	.	.

Means and Medians for Survival Time

Hypertension	Median
	95% …
	Upper Bound
0	.
1	.
Overall	.

a. Estimation is limited to the largest survival time if it is censored.

Overall Comparisons

	Chi-Square	df	Sig.
Log Rank (Mantel-Cox)	,907	1	,341
Breslow (Generalized Wilcoxon)	1,017	1	,313

Test of equality of survival distributions for the different levels of Hypertension.

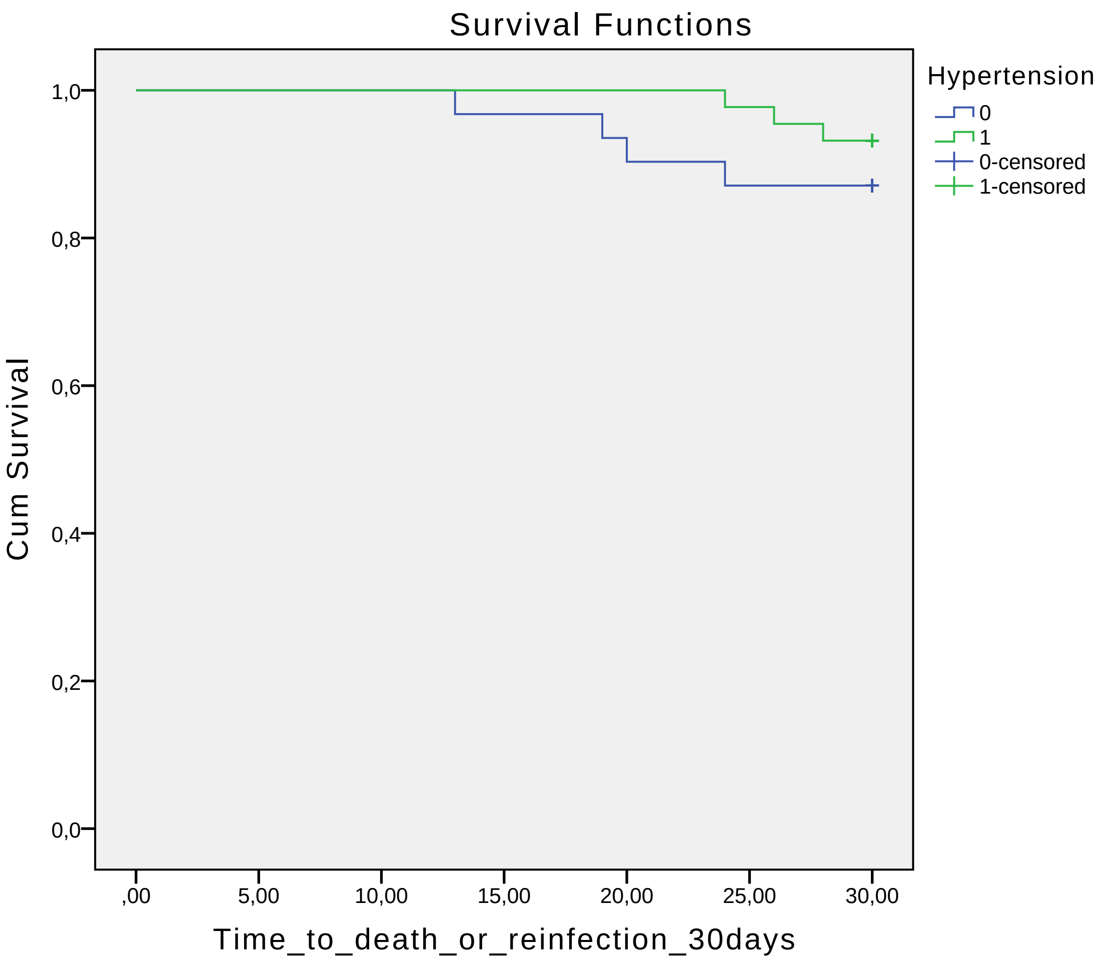

Kaplan-Meier

Case Processing Summary

Dyslipidemia	Total N	N of Events	Censored	
			N	Percent
0	42	5	37	88,1%
1	33	2	31	93,9%
Overall	75	7	68	90,7%

Means and Medians for Survival Time

Dyslipidemia	Mean[a]				Median		
	Estimate	Std. Error	95% Confidence Interval		Estimate	Std. Error	95% …
			Lower Bound	Upper Bound			Lower Bound
0	28,905	,532	27,862	29,948	.	.	.
1	29,697	,212	29,281	30,113	.	.	.
Overall	29,253	,316	28,635	29,872	.	.	.

Means and Medians for Survival Time

Dyslipidemia	Median
	95% …
	Upper Bound
0	.
1	.
Overall	.

a. Estimation is limited to the largest survival time if it is censored.

Overall Comparisons

	Chi-Square	df	Sig.
Log Rank (Mantel-Cox)	,781	1	,377
Breslow (Generalized Wilcoxon)	,823	1	,364

Test of equality of survival distributions for the different levels of Dyslipidemia.

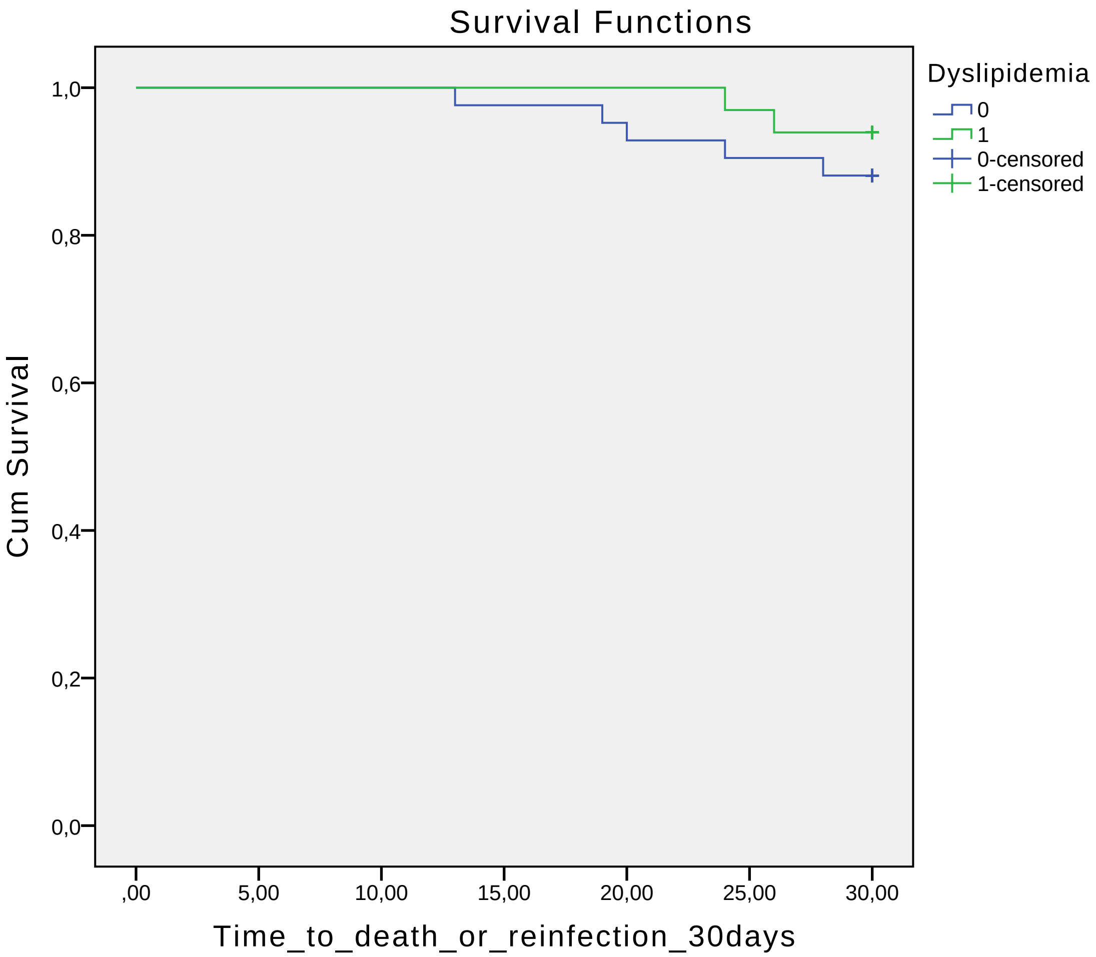

Kaplan-Meier

Warnings

No statistics are computed because all cases are censored.

Case Processing Summary

Chronic_respiratory_disease	Total N	N of Events	Censored	
			N	Percent
0	64	7	57	89,1%
1	11	0	11	100,0%
Overall	75	7	68	90,7%

Overall Comparisons

	Chi-Square	df	Sig.
Log Rank (Mantel-Cox)	1,266	1	,261
Breslow (Generalized Wilcoxon)	1,264	1	,261

Test of equality of survival distributions for the different levels of Chronic_respiratory_disease.

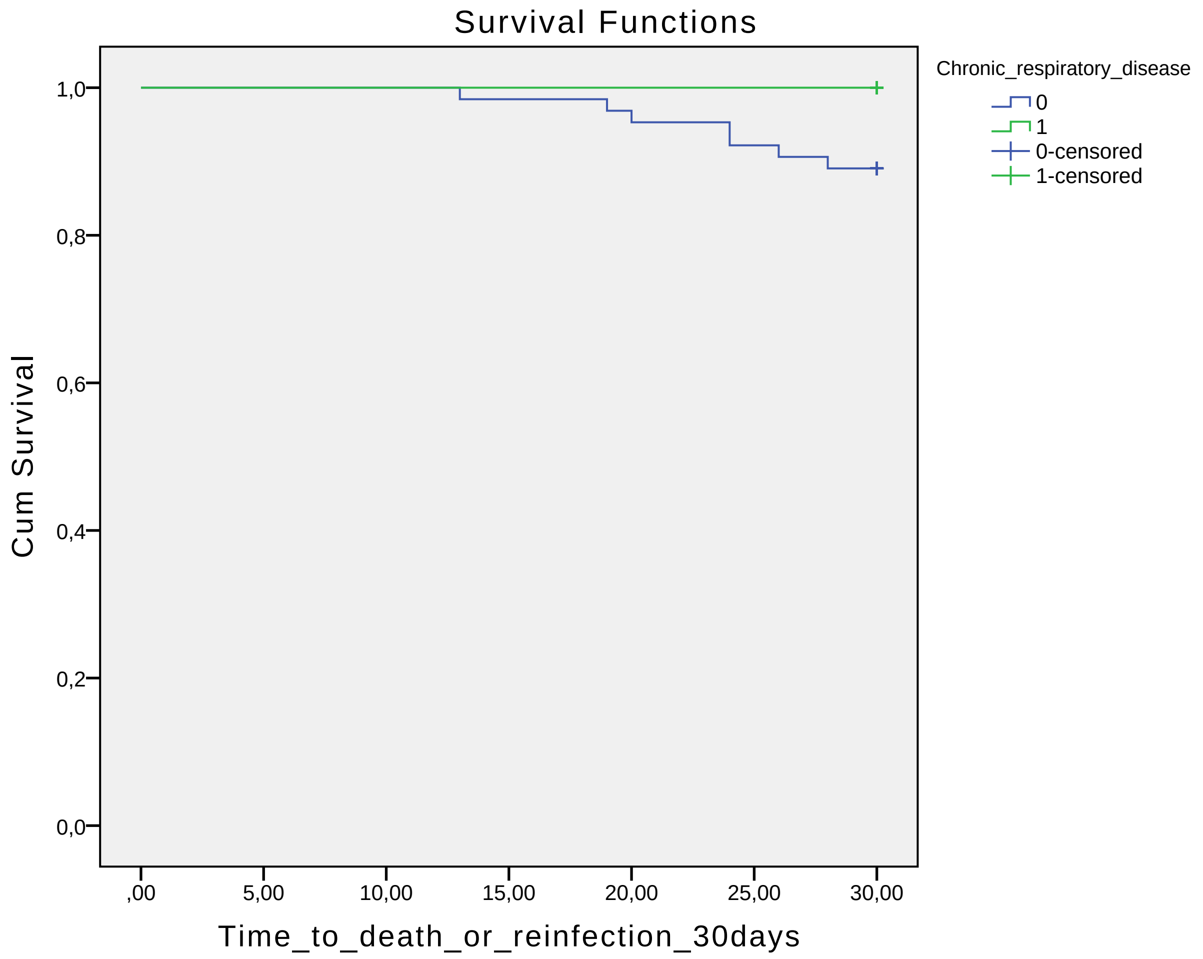

Kaplan-Meier

Warnings

No statistics are computed because all cases are censored.

Case Processing Summary

COPD	Total N	N of Events	Censored	
			N	Percent
0	70	7	63	90,0%
1	5	0	5	100,0%
Overall	75	7	68	90,7%

Overall Comparisons

	Chi-Square	df	Sig.
Log Rank (Mantel-Cox)	,524	1	,469
Breslow (Generalized Wilcoxon)	,523	1	,469

Test of equality of survival distributions for the different levels of COPD.

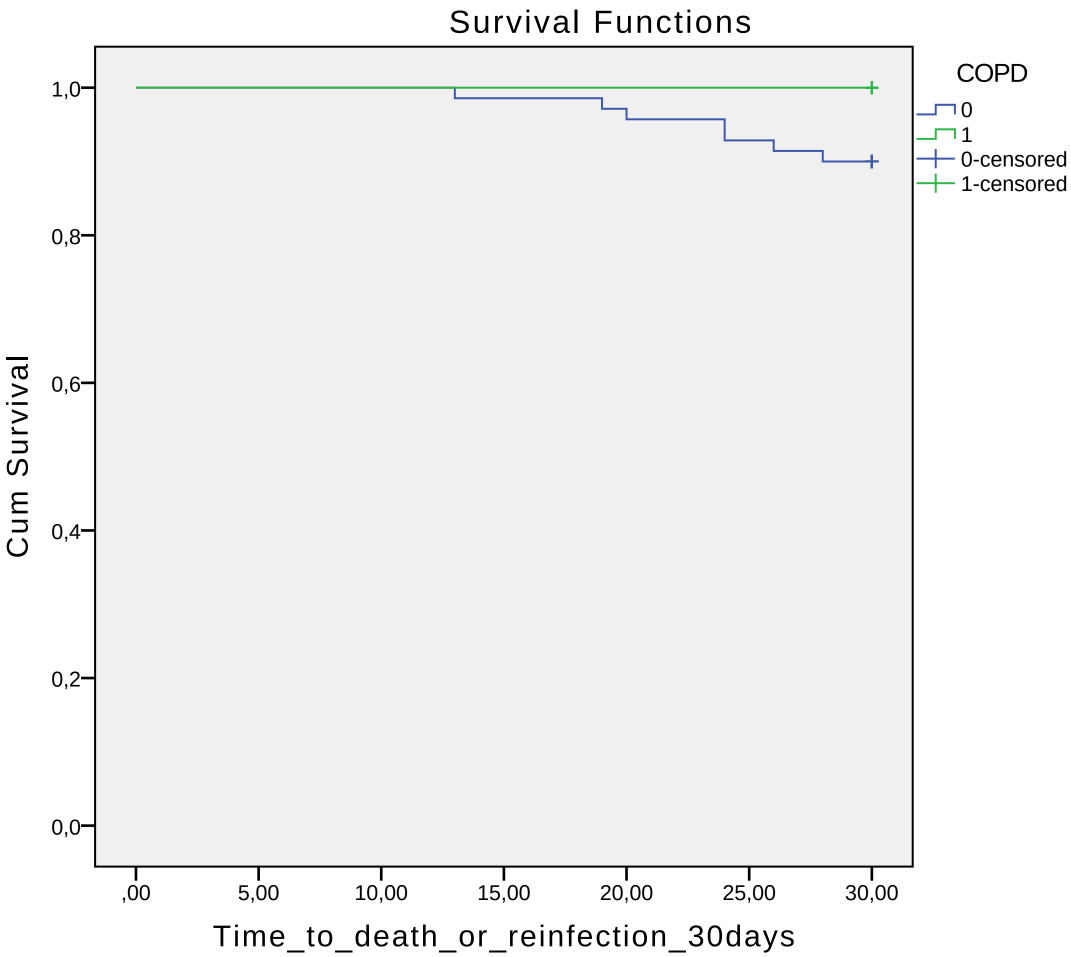

Kaplan-Meier

Case Processing Summary

Heart_failure	Total N	N of Events	Censored	
			N	Percent
0	63	6	57	90,5%
1	12	1	11	91,7%
Overall	75	7	68	90,7%

Means and Medians for Survival Time

Heart_failure	Mean[a]				Median		
	Estimate	Std. Error	95% Confidence Interval		Estimate	Std. Error	95% …
			Lower Bound	Upper Bound			Lower Bound
0	29,206	,364	28,493	29,920	.	.	.
1	29,500	,479	28,562	30,438	.	.	.
Overall	29,253	,316	28,635	29,872	.	.	.

Means and Medians for Survival Time

Heart_failure	Median
	95% …
	Upper Bound
0	.
1	.
Overall	.

a. Estimation is limited to the largest survival time if it is censored.

Overall Comparisons

	Chi-Square	df	Sig.
Log Rank (Mantel-Cox)	,020	1	,889
Breslow (Generalized Wilcoxon)	,020	1	,887

Test of equality of survival distributions for the different levels of Heart_failure.

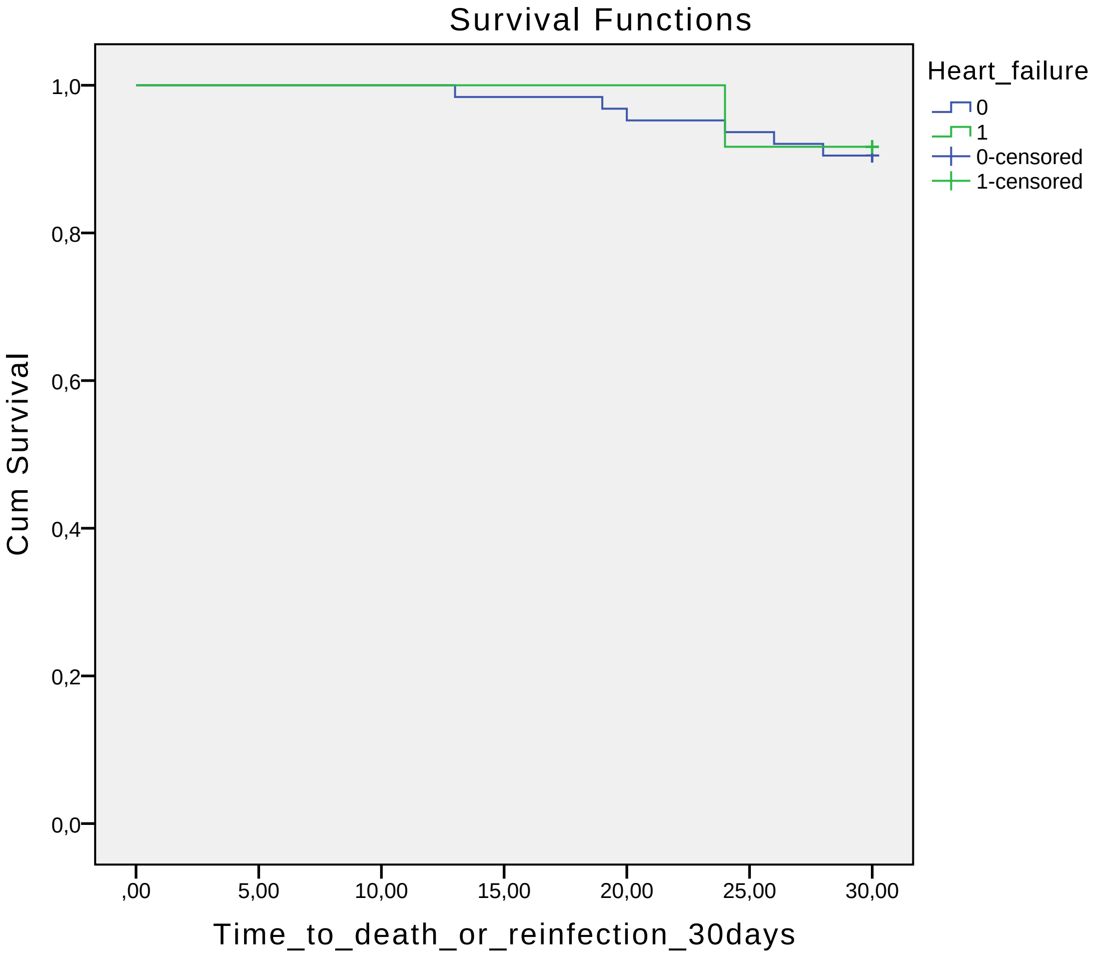

Kaplan-Meier

Warnings

No statistics are computed because all cases are censored.

Case Processing Summary

Myocardial_infarction	Total N	N of Events	Censored	
			N	Percent
0	66	7	59	89,4%
1	9	0	9	100,0%
Overall	75	7	68	90,7%

Overall Comparisons

	Chi-Square	df	Sig.
Log Rank (Mantel-Cox)	1,003	1	,317
Breslow (Generalized Wilcoxon)	1,002	1	,317

Test of equality of survival distributions for the different levels of Myocardial_infarction.

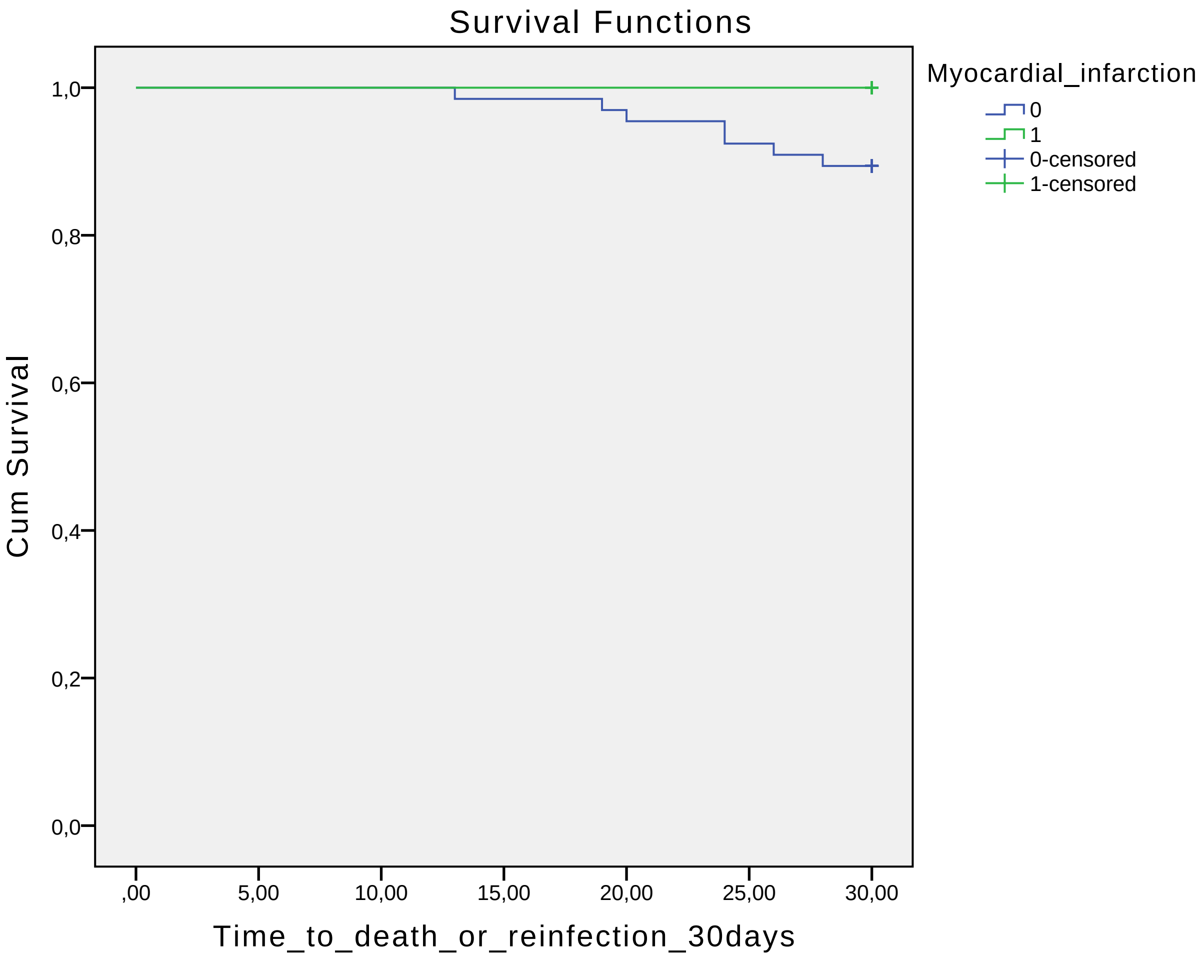

Kaplan-Meier

Case Processing Summary

Peripheral_arterial_disease	Total N	N of Events	Censored	
			N	Percent
0	67	6	61	91,0%
1	8	1	7	87,5%
Overall	75	7	68	90,7%

Means and Medians for Survival Time

Peripheral_arterial_disease	Mean[a]				Median		
	Estimate	Std. Error	95% Confidence Interval		Estimate	Std. Error	95% …
			Lower Bound	Upper Bound			Lower Bound
0	29,328	,317	28,707	29,949	.	.	.
1	28,625	1,286	26,104	31,146	.	.	.
Overall	29,253	,316	28,635	29,872	.	.	.

Means and Medians for Survival Time

Peripheral_arterial_disease	Median
	95% …
	Upper Bound
0	.
1	.
Overall	.

a. Estimation is limited to the largest survival time if it is censored.

Overall Comparisons

	Chi-Square	df	Sig.
Log Rank (Mantel-Cox)	,136	1	,712
Breslow (Generalized Wilcoxon)	,160	1	,689

Test of equality of survival distributions for the different levels of Peripheral_arterial_disease.

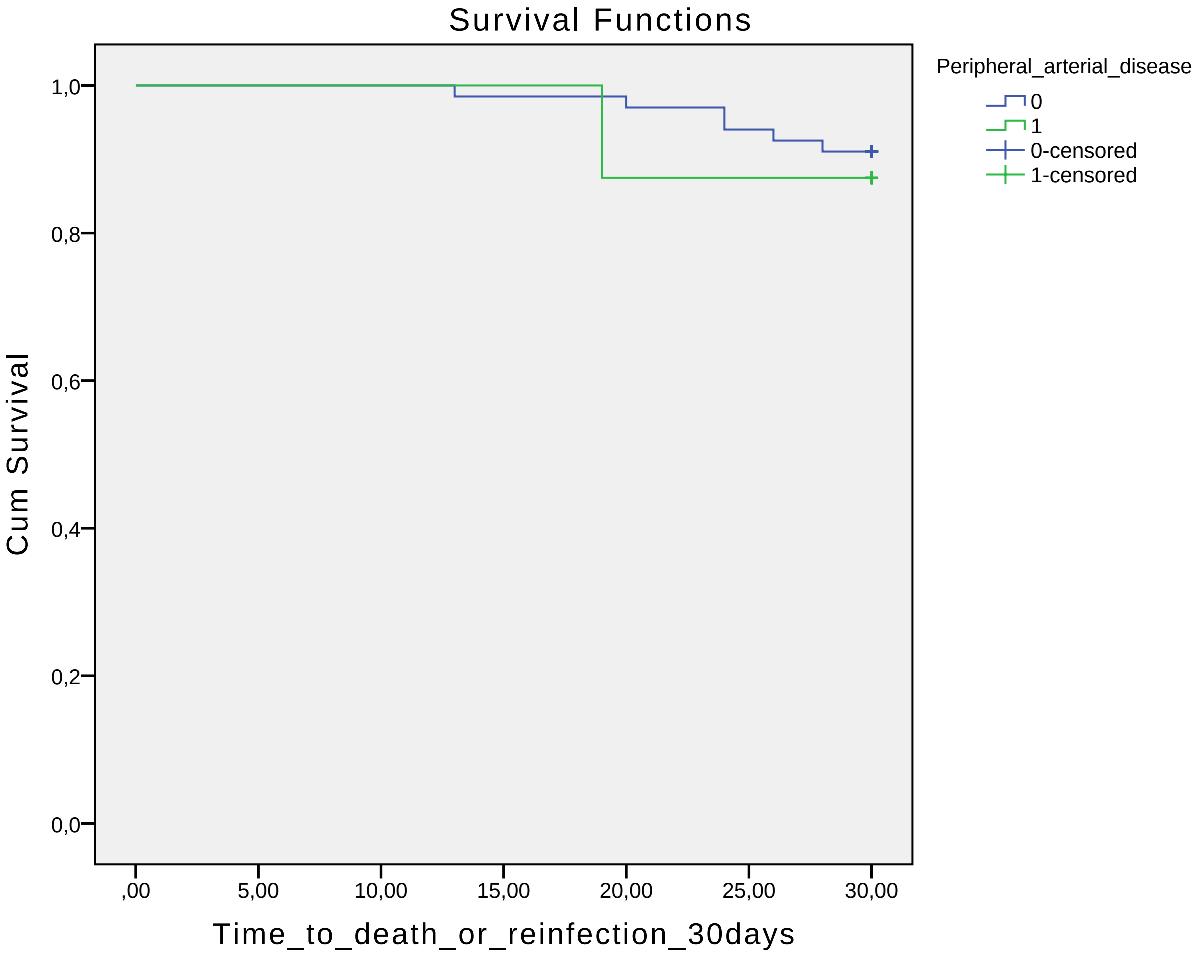

Kaplan-Meier

Warnings

No statistics are computed because all cases are censored.

Case Processing Summary

Cerebrovascular_disease	Total N	N of Events	Censored	
			N	Percent
0	59	7	52	88,1%
1	16	0	16	100,0%
Overall	75	7	68	90,7%

Overall Comparisons

	Chi-Square	df	Sig.
Log Rank (Mantel-Cox)	2,005	1	,157
Breslow (Generalized Wilcoxon)	2,003	1	,157

Test of equality of survival distributions for the different levels of Cerebrovascular_disease.

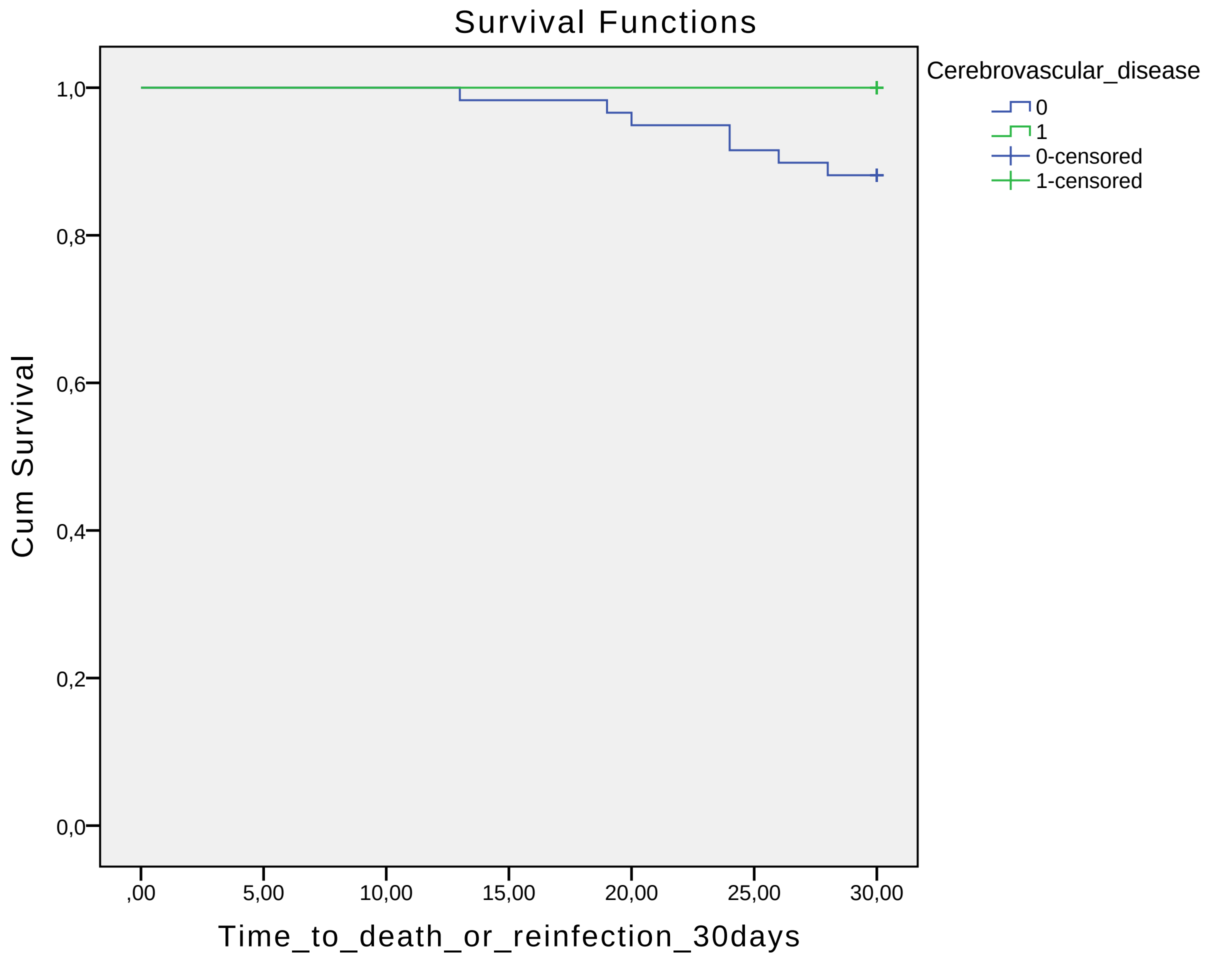

Kaplan-Meier

Case Processing Summary

Hemiplegia	Total N	N of Events	Censored	
			N	Percent
0	65	6	59	90,8%
2	10	1	9	90,0%
Overall	75	7	68	90,7%

Means and Medians for Survival Time

Hemiplegia	Mean[a]				Median			
	Estimate	Std. Error	95% Confidence Interval		Estimate	Std. Error	95% Confidence Interval	
			Lower Bound	Upper Bound			Lower Bound	Upper Bound
0	29,231	,353	28,538	29,923	.	.	.	.
2	29,400	,569	28,284	30,516	.	.	.	.
Overall	29,253	,316	28,635	29,872	.	.	.	.

a. Estimation is limited to the largest survival time if it is censored.

Overall Comparisons

	Chi-Square	df	Sig.
Log Rank (Mantel-Cox)	,004	1	,949
Breslow (Generalized Wilcoxon)	,004	1	,951

Test of equality of survival distributions for the different levels of Hemiplegia.

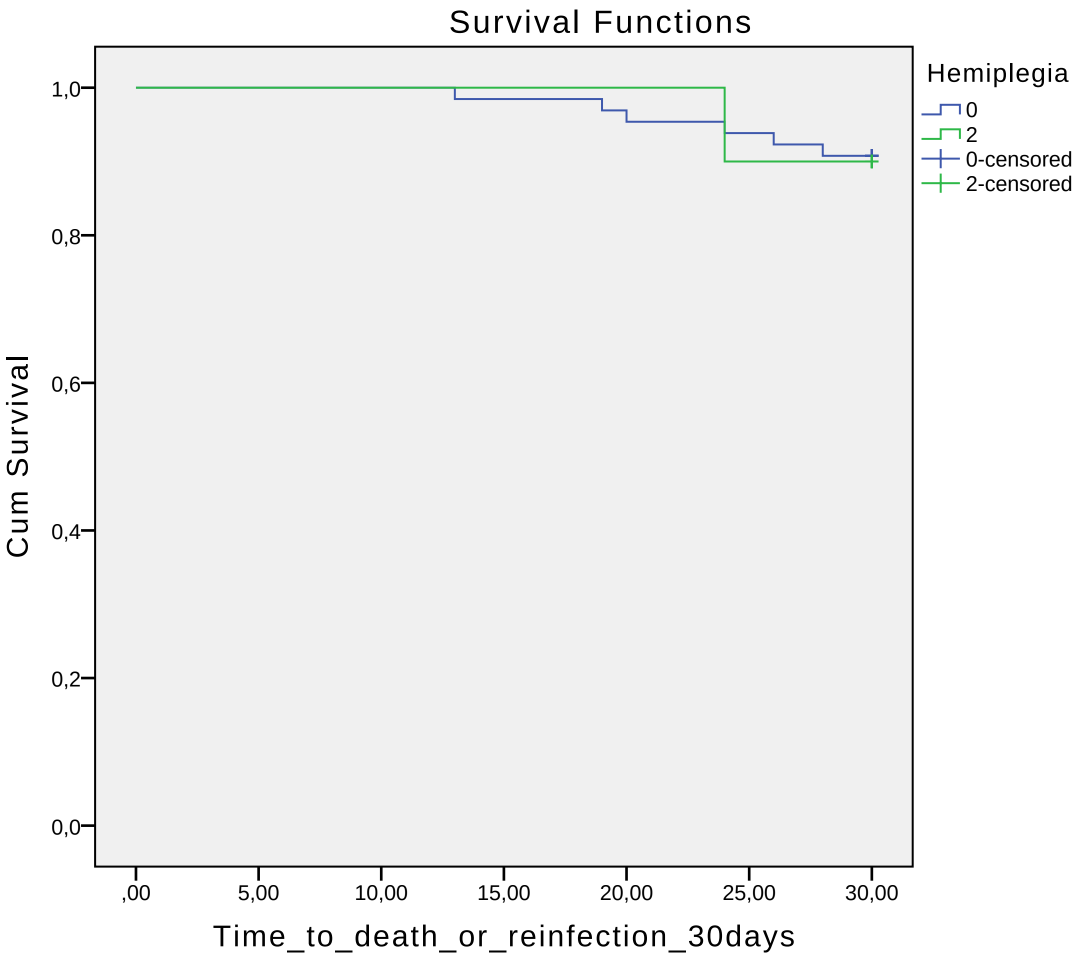

Kaplan-Meier

Warnings

No statistics are computed because all cases are censored.

Case Processing Summary

Gastroduodenal_ulcer	Total N	N of Events	Censored	
			N	Percent
0	69	7	62	89,9%
1	6	0	6	100,0%
Overall	75	7	68	90,7%

Overall Comparisons

	Chi-Square	df	Sig.
Log Rank (Mantel-Cox)	,638	1	,424
Breslow (Generalized Wilcoxon)	,638	1	,425

Test of equality of survival distributions for the different levels of Gastroduodenal_ulcer.

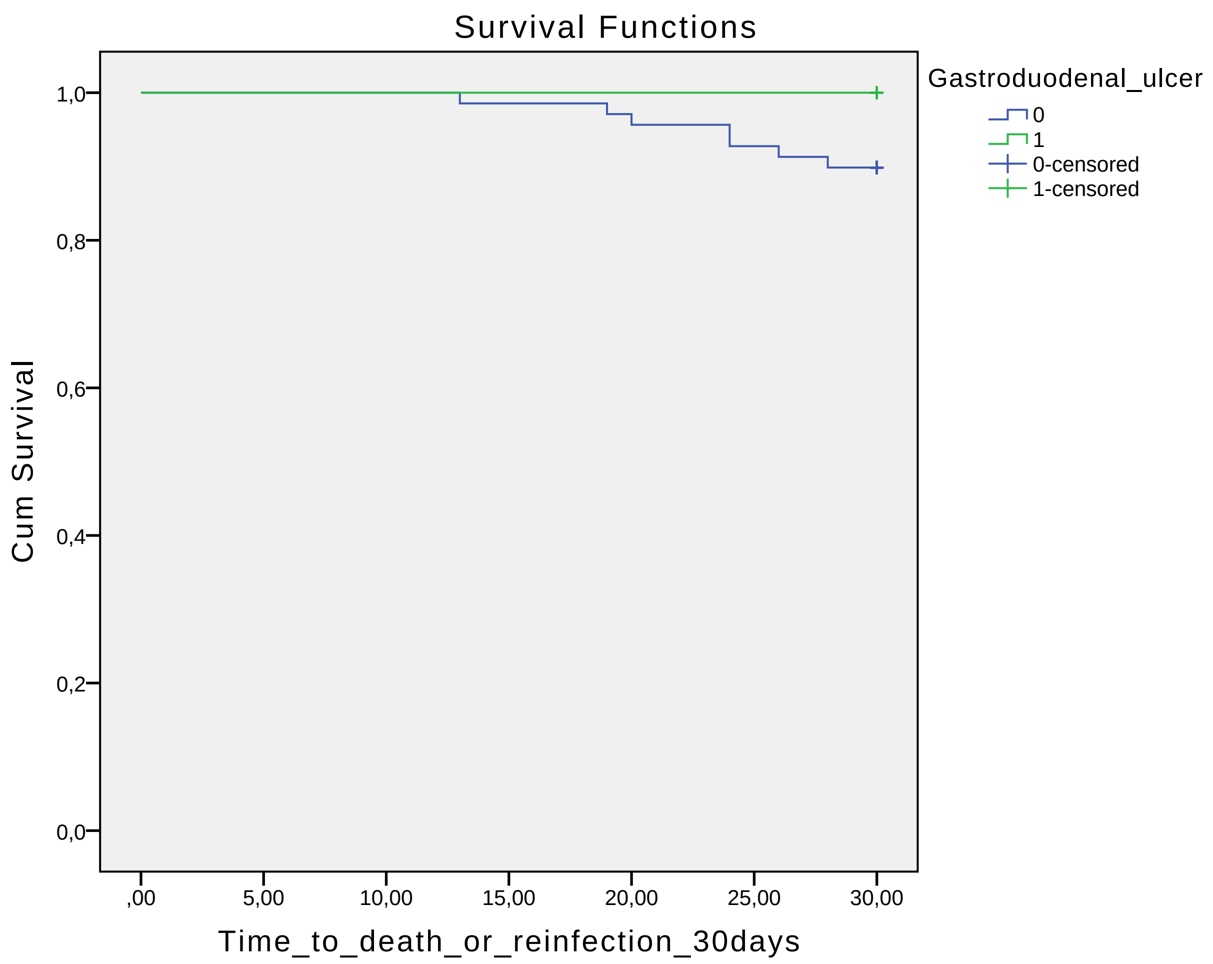

Kaplan-Meier

Warnings

No statistics are computed because all cases are censored.

Case Processing Summary

Chronic_kidney_disease	Total N	N of Events	Censored	
			N	Percent
0	55	7	48	87,3%
1	20	0	20	100,0%
Overall	75	7	68	90,7%

Overall Comparisons

	Chi-Square	df	Sig.
Log Rank (Mantel-Cox)	2,699	1	,100
Breslow (Generalized Wilcoxon)	2,696	1	,101

Test of equality of survival distributions for the different levels of Chronic_kidney_disease.

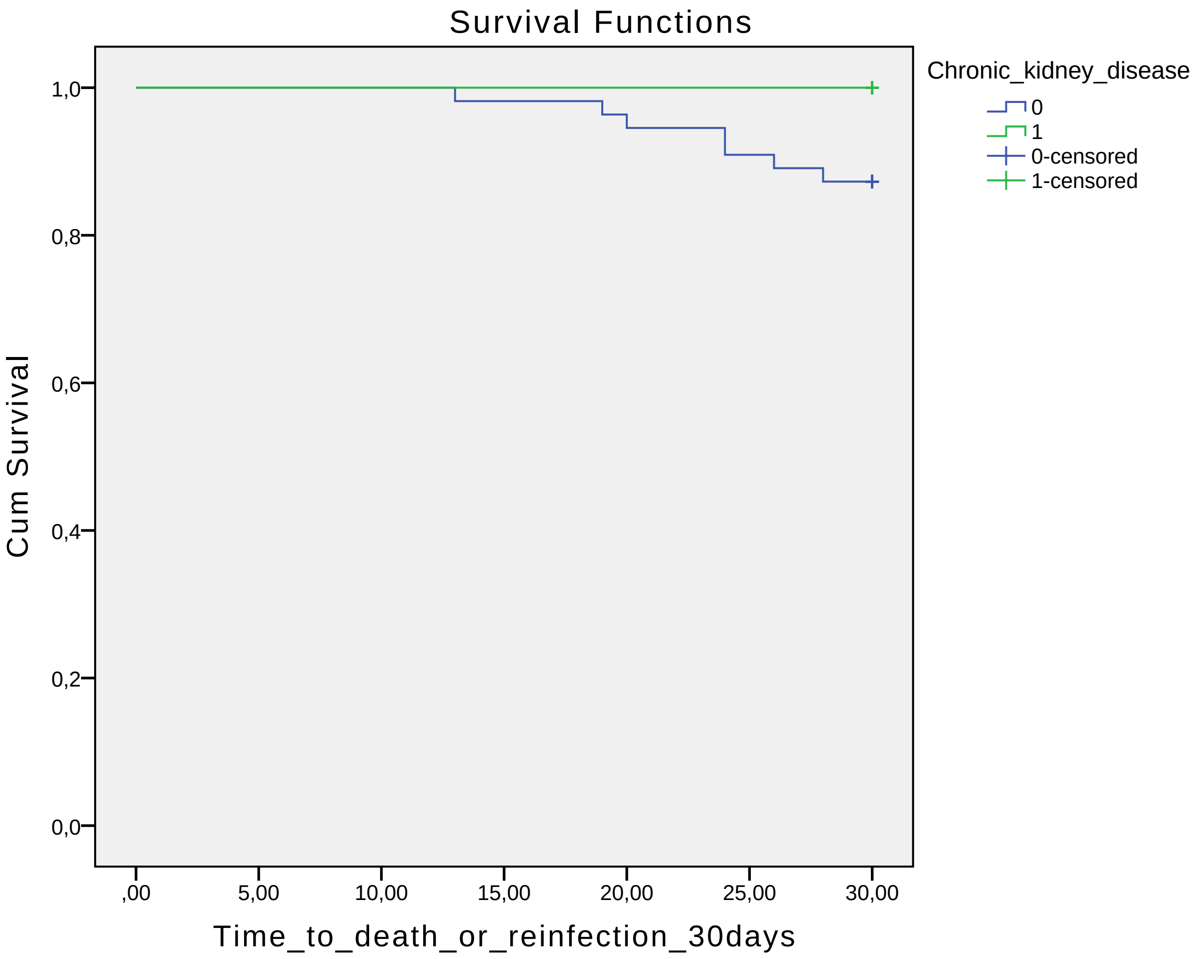

Kaplan-Meier

Warnings

No statistics are computed because all cases are censored.

Case Processing Summary

Moderate_severe_chronic_kidney_disease	Total N	N of Events	Censored	
			N	Percent
0	66	7	59	89,4%
2	9	0	9	100,0%
Overall	75	7	68	90,7%

Overall Comparisons

	Chi-Square	df	Sig.
Log Rank (Mantel-Cox)	1,003	1	,317
Breslow (Generalized Wilcoxon)	1,002	1	,317

Test of equality of survival distributions for the different levels of Moderate_severe_chronic_kidney_disease.

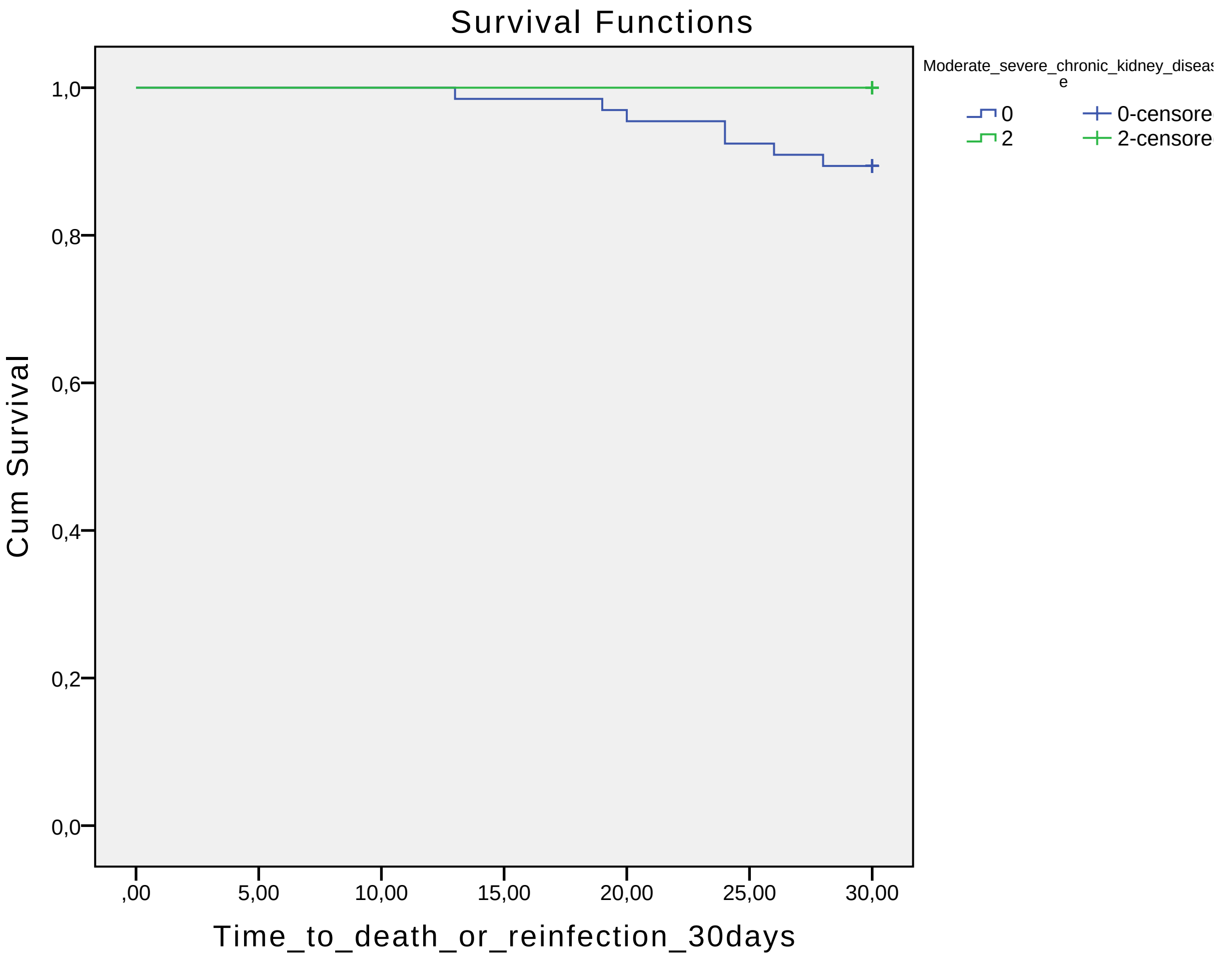

Kaplan-Meier

Warnings

No comparison analysis is performed because the factor variable has only one value for every stratum.

Case Processing Summary

Dyalisis	Total N	N of Events	Censored	
			N	Percent
0	75	7	68	90,7%
Overall	75	7	68	90,7%

Means and Medians for Survival Time

Dyalisis	Mean[a]				Median			
	Estimate	Std. Error	95% Confidence Interval		Estimate	Std. Error	95% Confidence Interval	
			Lower Bound	Upper Bound			Lower Bound	Upper Bound
0	29,253	,316	28,635	29,872	.	.	.	.
Overall	29,253	,316	28,635	29,872	.	.	.	.

a. Estimation is limited to the largest survival time if it is censored.

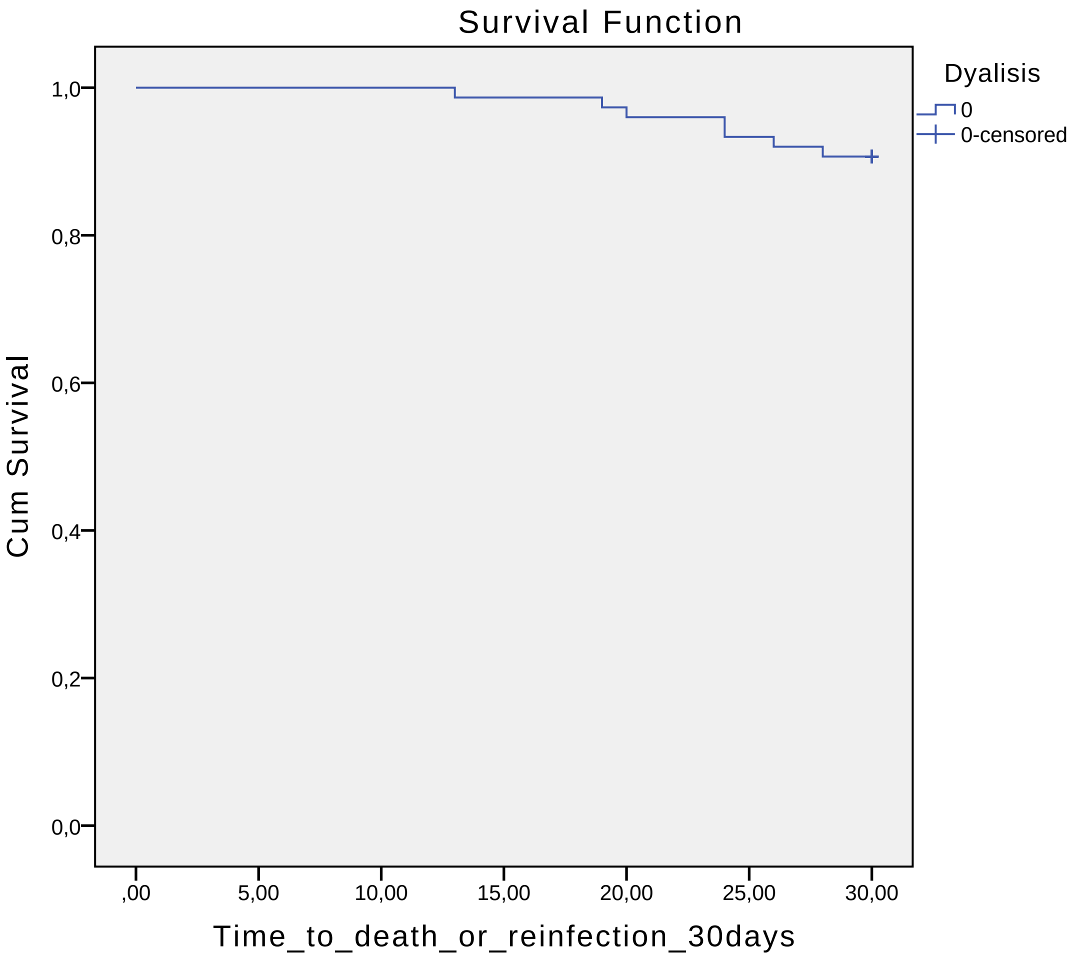

Kaplan-Meier

Warnings

No comparison analysis is performed because the factor variable has only one value for every stratum.

Case Processing Summary

HIV	Total N	N of Events	Censored	
			N	Percent
0	75	7	68	90,7%
Overall	75	7	68	90,7%

Means and Medians for Survival Time

HIV	Mean[a]				Median			
	Estimate	Std. Error	95% Confidence Interval		Estimate	Std. Error	95% Confidence Interval	
			Lower Bound	Upper Bound			Lower Bound	Upper Bound
0	29,253	,316	28,635	29,872	.	.	.	.
Overall	29,253	,316	28,635	29,872	.	.	.	.

a. Estimation is limited to the largest survival time if it is censored.

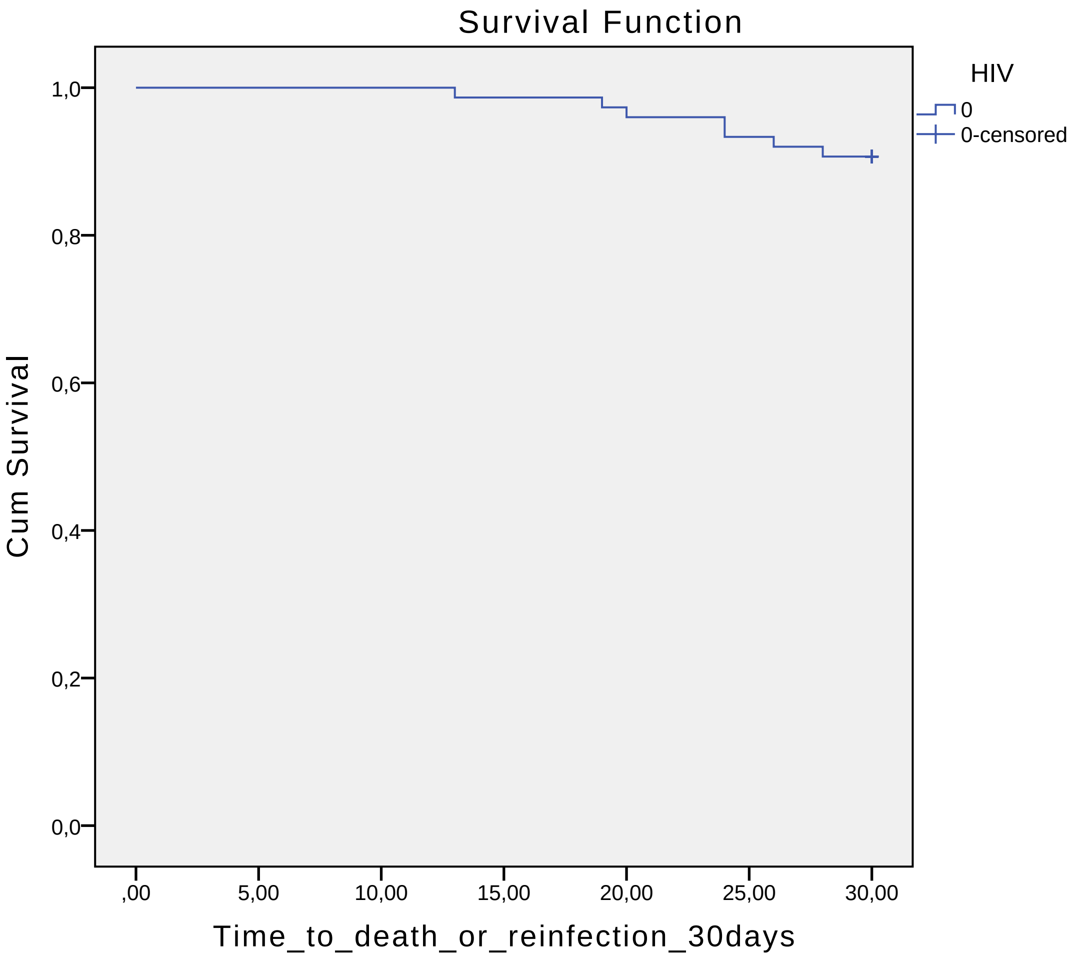

Kaplan-Meier

Warnings

No comparison analysis is performed because the factor variable has only one value for every stratum.

Case Processing Summary

AIDS	Total N	N of Events	Censored	
			N	Percent
0	75	7	68	90,7%
Overall	75	7	68	90,7%

Means and Medians for Survival Time

AIDS	Mean[a]				Median			
	Estimate	Std. Error	95% Confidence Interval		Estimate	Std. Error	95% Confidence Interval	
			Lower Bound	Upper Bound			Lower Bound	Upper Bound
0	29,253	,316	28,635	29,872	.	.	.	.
Overall	29,253	,316	28,635	29,872	.	.	.	.

a. Estimation is limited to the largest survival time if it is censored.

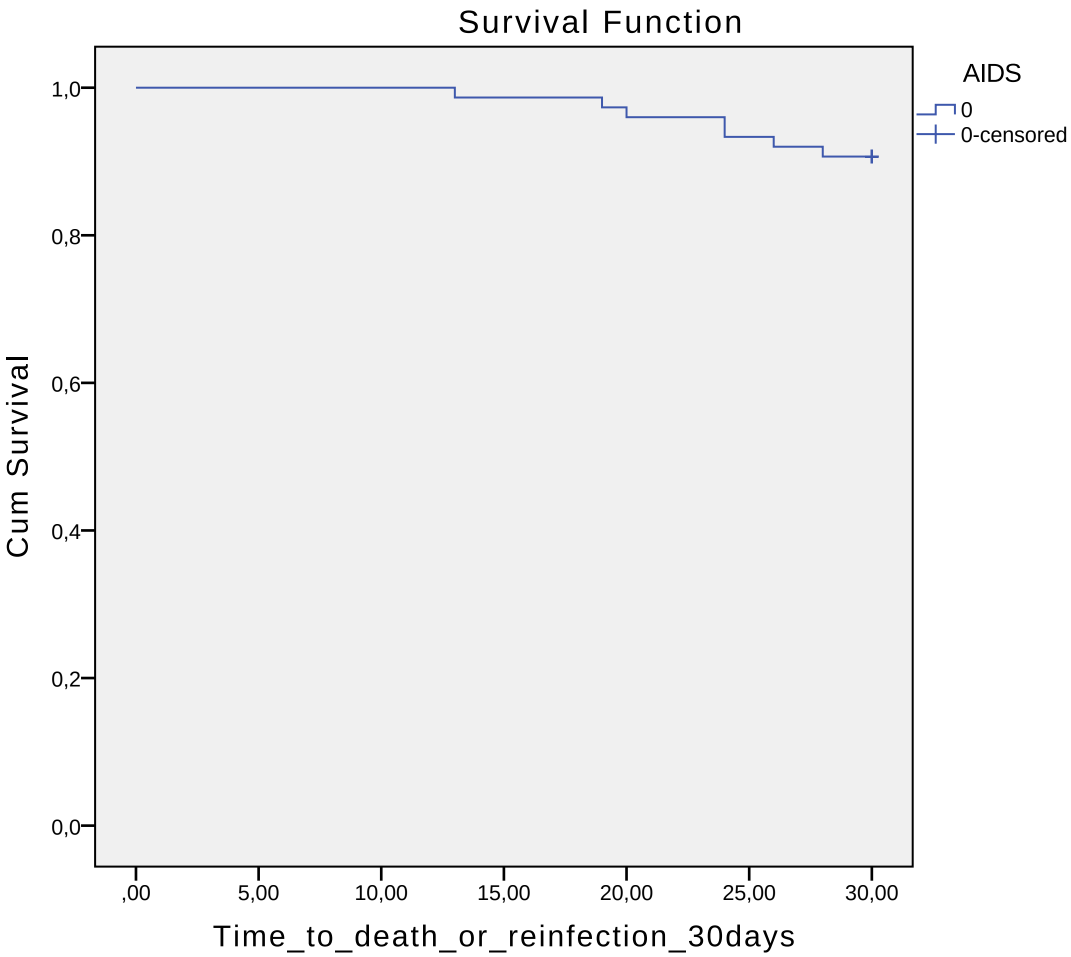

Kaplan-Meier

Warnings

No comparison analysis is performed because the factor variable has only one value for every stratum.

Case Processing Summary

Neutrophil_below_1000	Total N	N of Events	Censored	
			N	Percent
0	75	7	68	90,7%
Overall	75	7	68	90,7%

Means and Medians for Survival Time

Neutrophil_below_1000	Mean[a]				Median		
			95% Confidence Interval				95% ...
	Estimate	Std. Error	Lower Bound	Upper Bound	Estimate	Std. Error	Lower Bound
0	29,253	,316	28,635	29,872	.	.	.
Overall	29,253	,316	28,635	29,872	.	.	.

Means and Medians for Survival Time

Neutrophil_below_1000	Median
	95% ...
	Upper Bound
0	.
Overall	.

a. Estimation is limited to the largest survival time if it is censored.

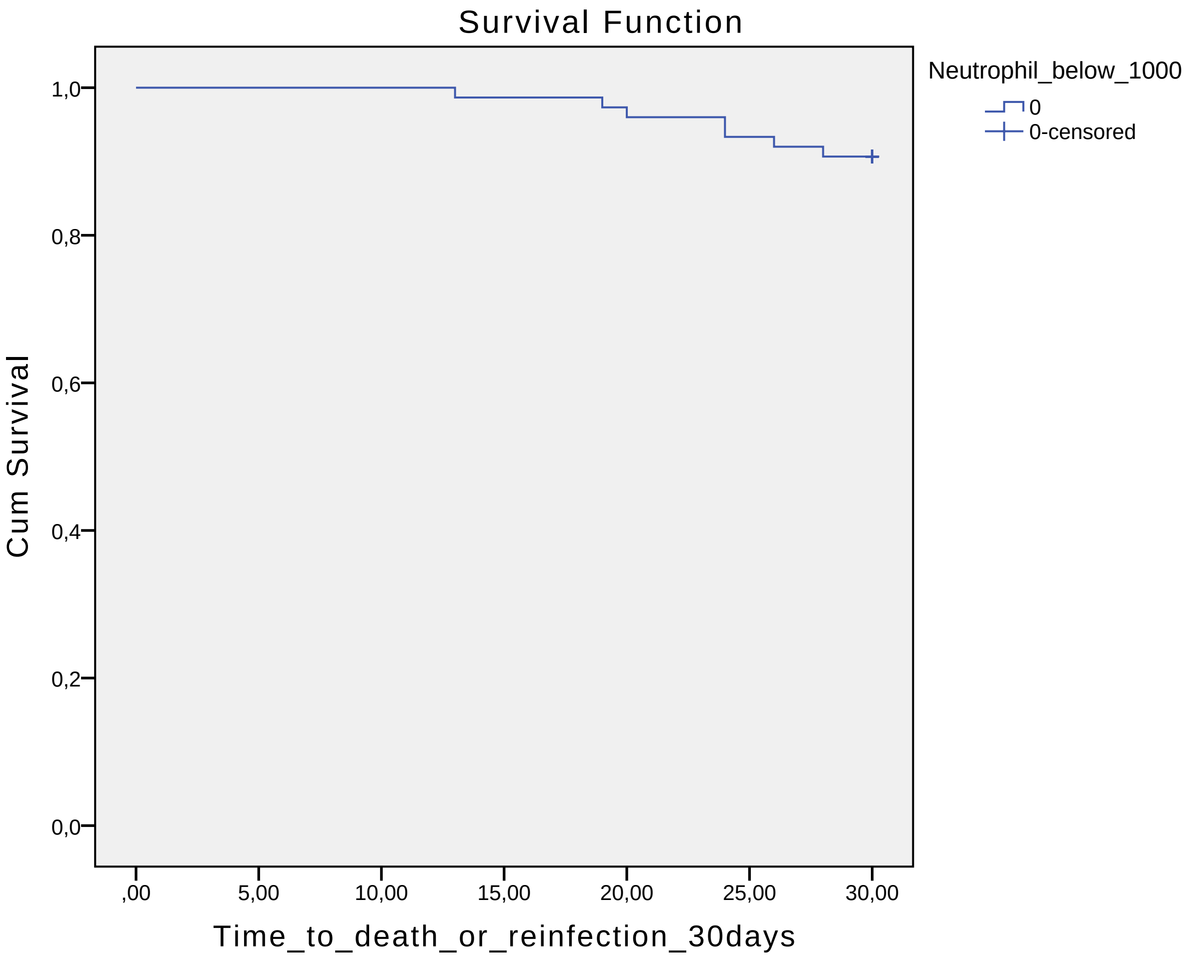

Kaplan-Meier

Warnings

No comparison analysis is performed because the factor variable has only one value for every stratum.

Case Processing Summary

Neutrophil_below_500	Total N	N of Events	Censored	
			N	Percent
0	75	7	68	90,7%
Overall	75	7	68	90,7%

Means and Medians for Survival Time

Neutrophil_below_500	Mean[a]				Median		
	Estimate	Std. Error	95% Confidence Interval		Estimate	Std. Error	95% …
			Lower Bound	Upper Bound			Lower Bound
0	29,253	,316	28,635	29,872	.	.	.
Overall	29,253	,316	28,635	29,872	.	.	.

Means and Medians for Survival Time

Neutrophil_below_500	Median
	95% …
	Upper Bound
0	.
Overall	.

a. Estimation is limited to the largest survival time if it is censored.

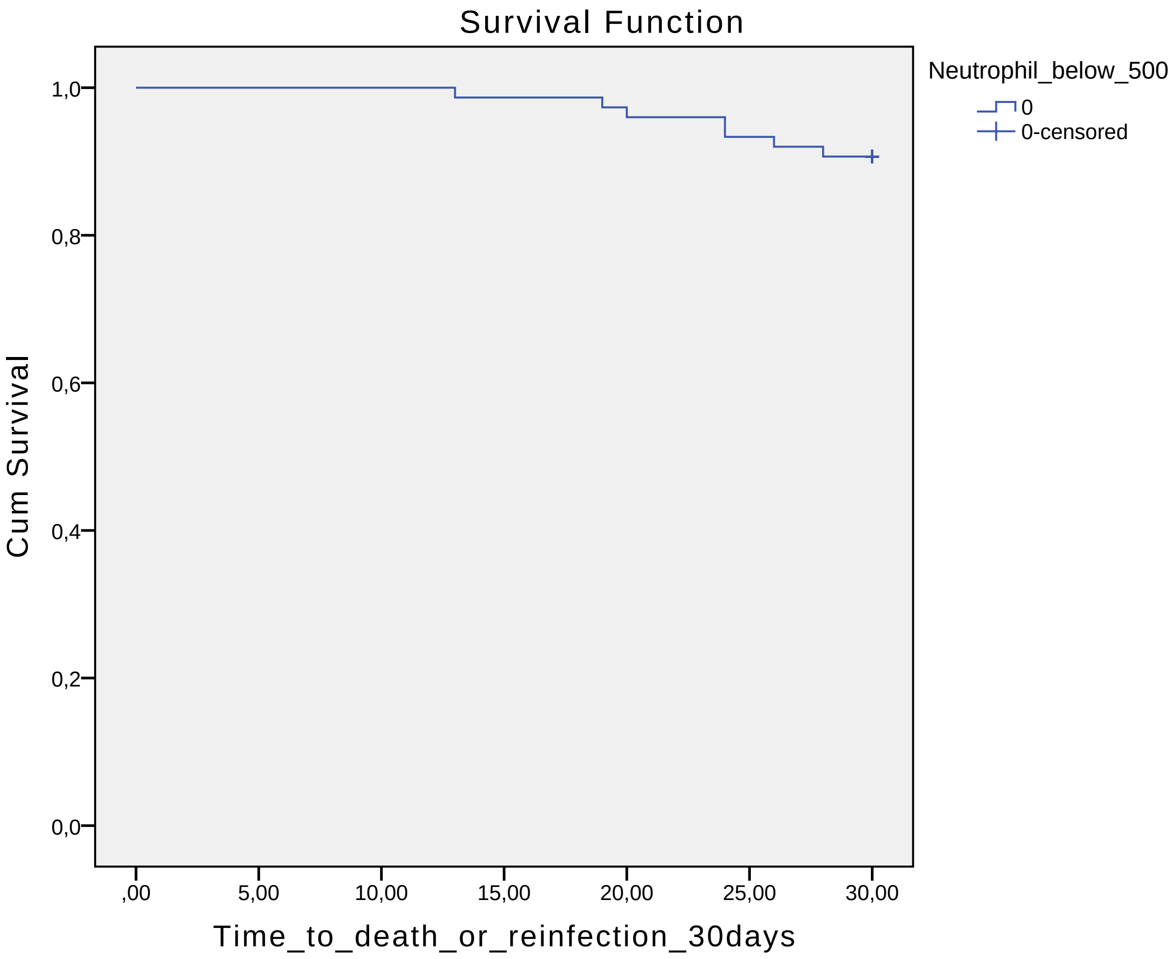

Kaplan-Meier

Warnings

No statistics are computed because all cases are censored.

Case Processing Summary

Corticosteroids	Total N	N of Events	Censored	
			N	Percent
0	71	7	64	90,1%
1	4	0	4	100,0%
Overall	75	7	68	90,7%

Overall Comparisons

	Chi-Square	df	Sig.
Log Rank (Mantel-Cox)	,413	1	,520
Breslow (Generalized Wilcoxon)	,413	1	,521

Test of equality of survival distributions for the different levels of Corticosteroids.

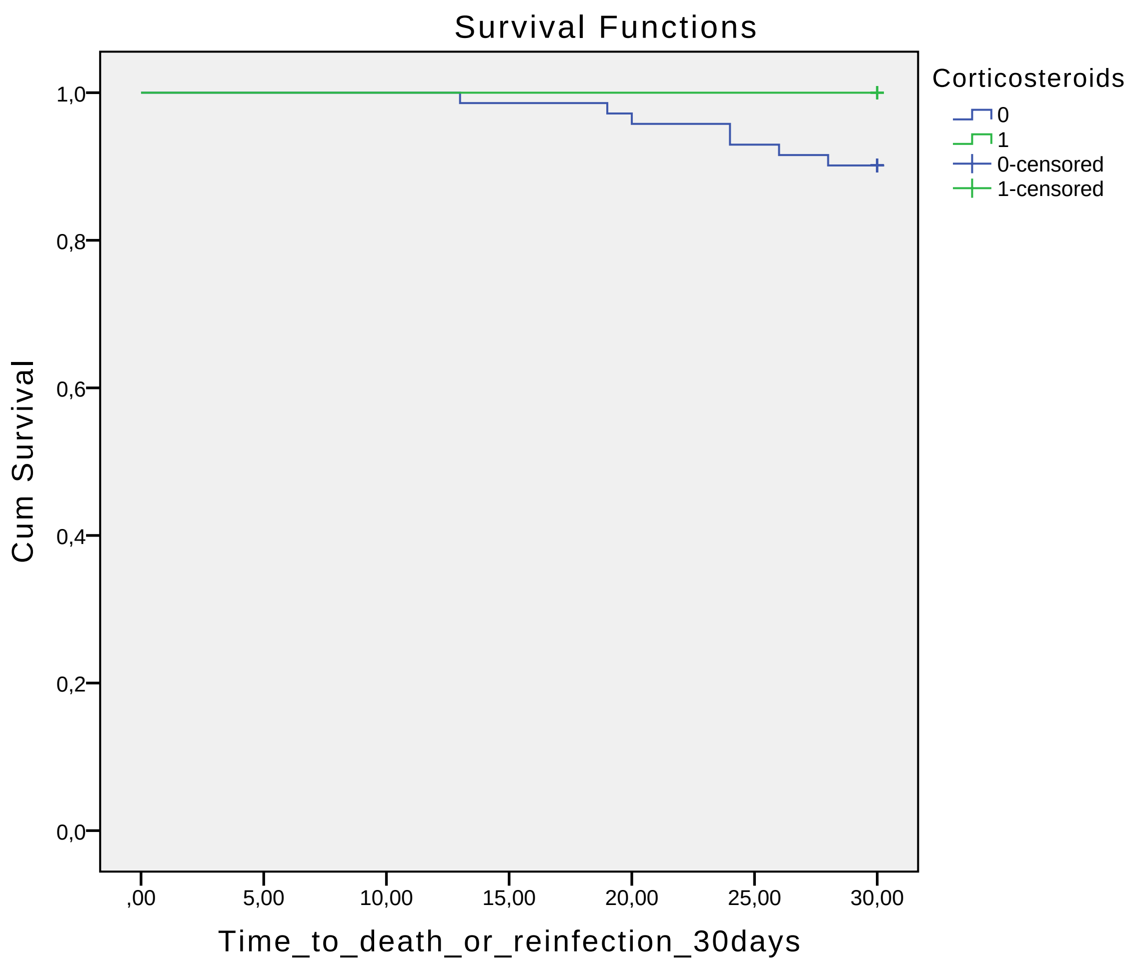

Kaplan-Meier

Warnings

No statistics are computed because all cases are censored.

Case Processing Summary

Transplant	Total N	N of Events	Censored	
			N	Percent
0	73	7	66	90,4%
1	1	0	1	100,0%
Overall	74	7	67	90,5%

Overall Comparisons

	Chi-Square	df	Sig.
Log Rank (Mantel-Cox)	,100	1	,751
Breslow (Generalized Wilcoxon)	,100	1	,752

Test of equality of survival distributions for the different levels of Transplant.

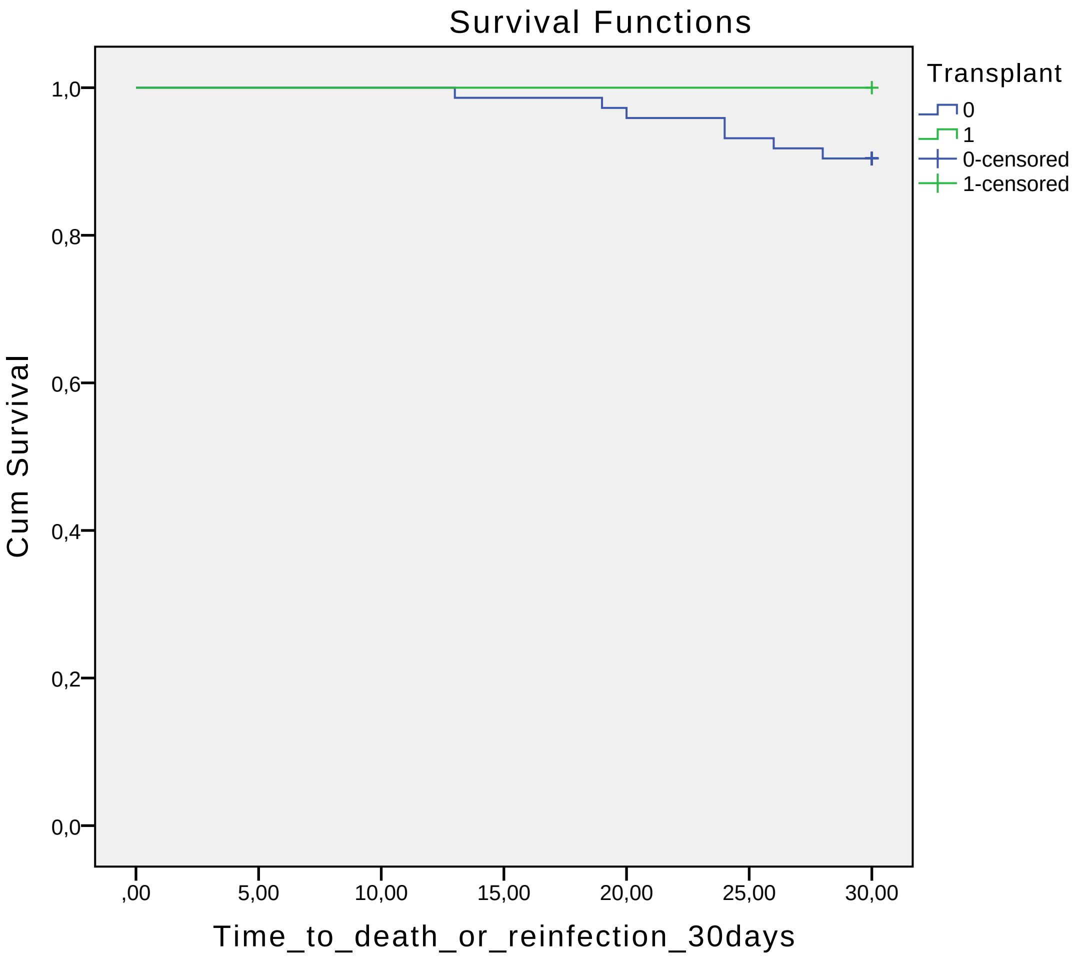

Kaplan-Meier

Warnings

No statistics are computed because all cases are censored.

Case Processing Summary

Immunosuppression	Total N	N of Events	Censored	
			N	Percent
0	71	7	64	90,1%
1	4	0	4	100,0%
Overall	75	7	68	90,7%

Overall Comparisons

	Chi-Square	df	Sig.
Log Rank (Mantel-Cox)	,413	1	,520
Breslow (Generalized Wilcoxon)	,413	1	,521

Test of equality of survival distributions for the different levels of Immunosuppression.

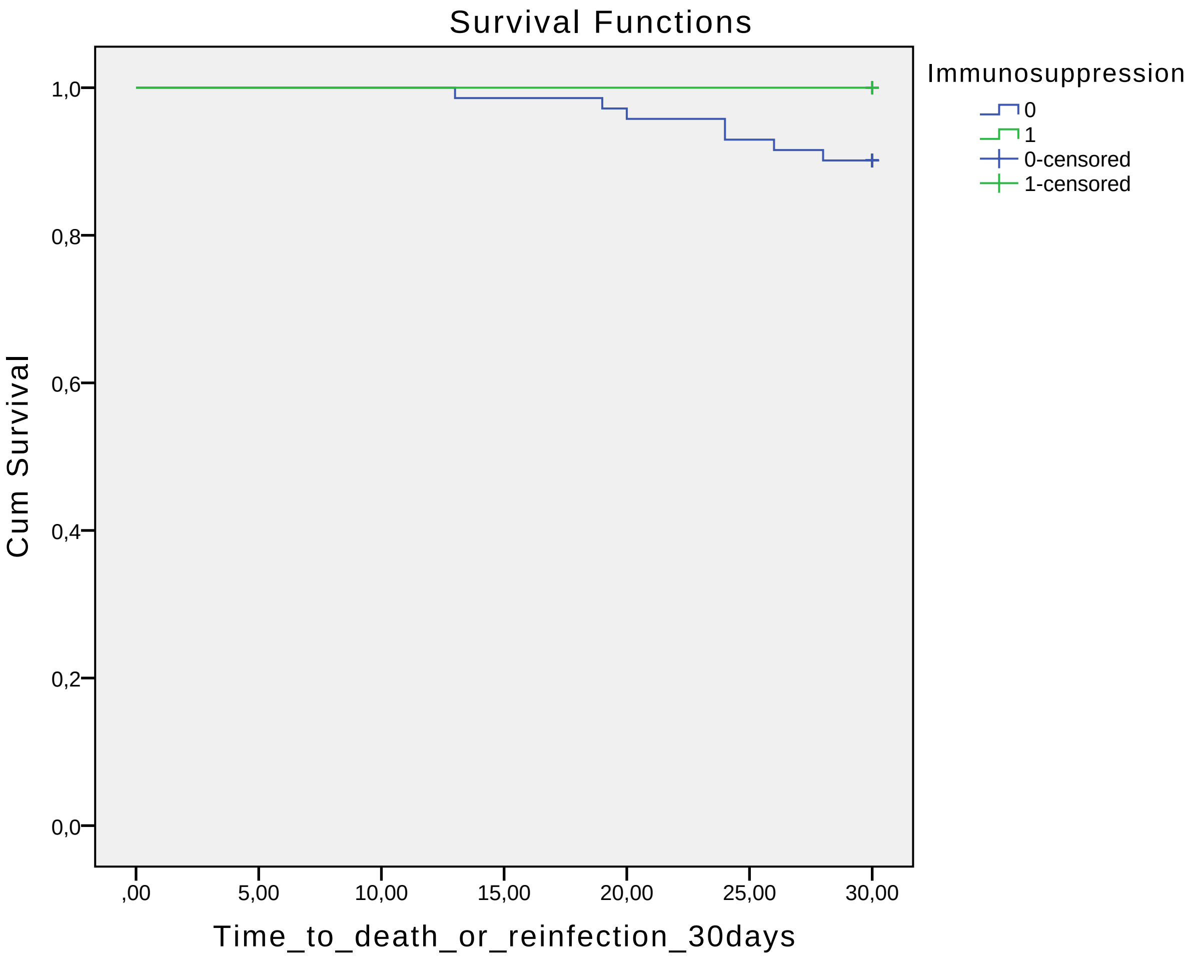

Kaplan-Meier

Warnings

No statistics are computed because all cases are censored.

Case Processing Summary

Leukocytosis	Total N	N of Events	Censored	
			N	Percent
0	51	7	44	86,3%
1	20	0	20	100,0%
Overall	71	7	64	90,1%

Overall Comparisons

	Chi-Square	df	Sig.
Log Rank (Mantel-Cox)	2,925	1	,087
Breslow (Generalized Wilcoxon)	2,920	1	,087

Test of equality of survival distributions for the different levels of Leukocytosis.

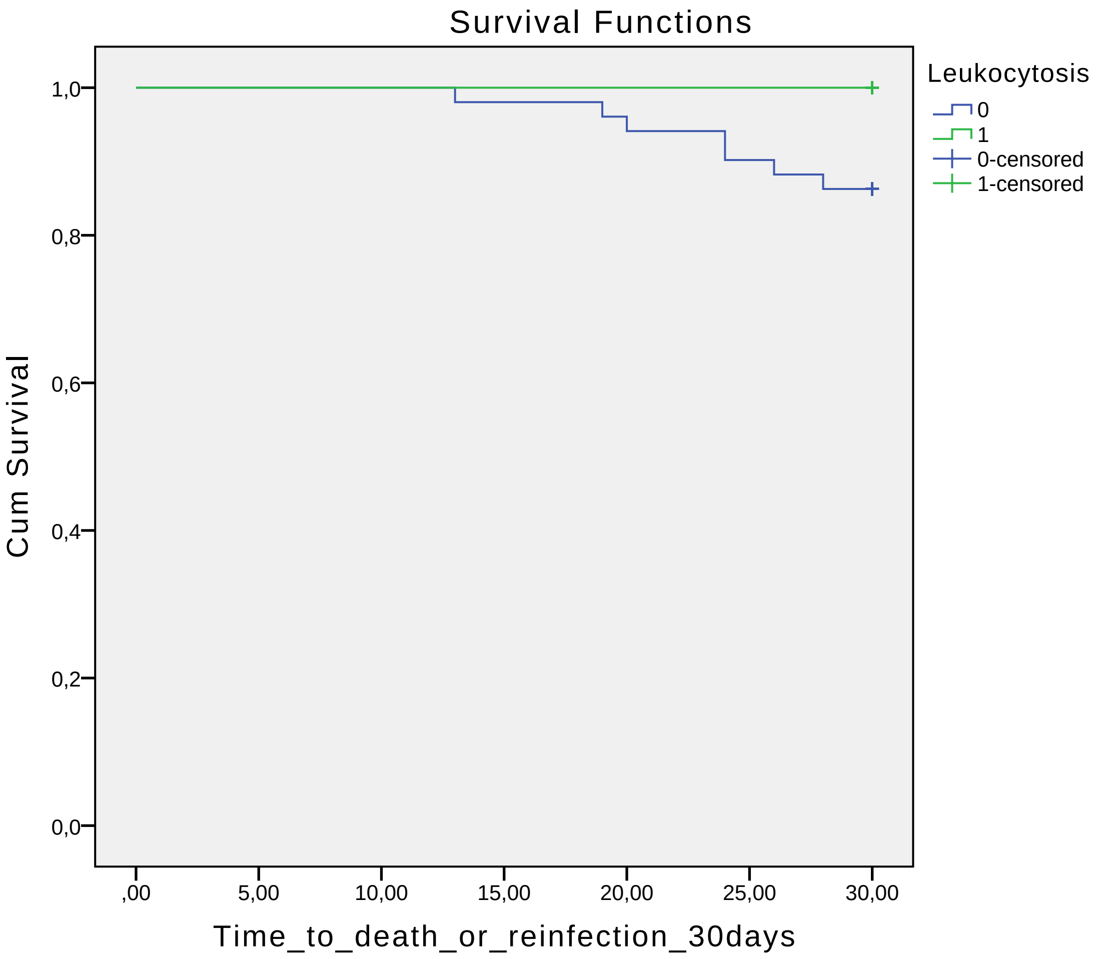

Kaplan-Meier

Case Processing Summary

Leukopenia	Total N	N of Events	Censored	
			N	Percent
0	69	6	63	91,3%
1	2	1	1	50,0%
Overall	71	7	64	90,1%

Means and Medians for Survival Time

Leukopenia	Mean[a]				Median			
	Estimate	Std. Error	95% Confidence Interval		Estimate	Std. Error	95% Confidence Interval	
			Lower Bound	Upper Bound			Lower Bound	Upper Bound
0	29,217	,342	28,548	29,887	.	.	.	.
1	29,000	,707	27,614	30,386	28,000	.	.	.
Overall	29,211	,333	28,559	29,863	.	.	.	.

a. Estimation is limited to the largest survival time if it is censored.

Overall Comparisons

	Chi-Square	df	Sig.
Log Rank (Mantel-Cox)	3,175	1	,075
Breslow (Generalized Wilcoxon)	2,821	1	,093

Test of equality of survival distributions for the different levels of Leukopenia.

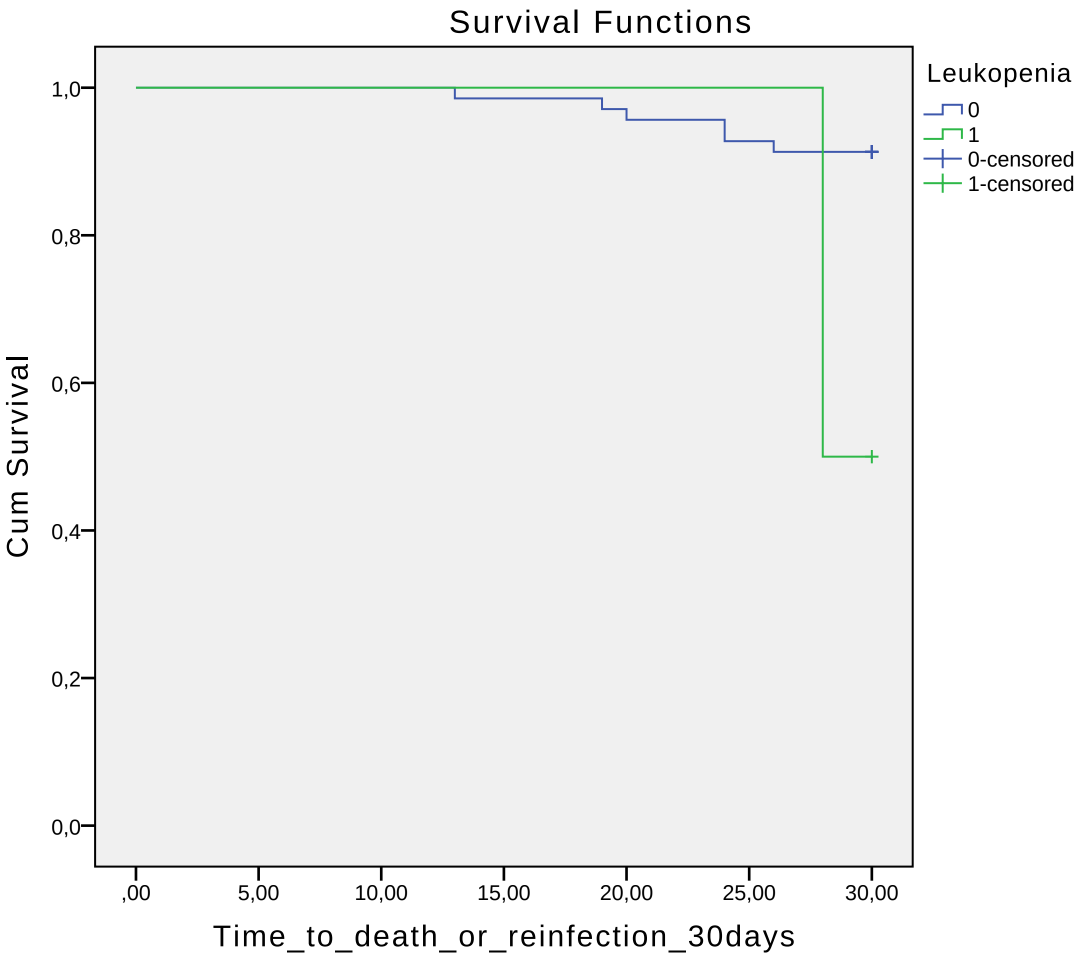

Kaplan-Meier

Case Processing Summary

Thrombopenia	Total N	N of Events	Censored	
			N	Percent
0	65	6	59	90,8%
1	6	1	5	83,3%
Overall	71	7	64	90,1%

Means and Medians for Survival Time

Thrombopenia	Mean[a]				Median		
	Estimate	Std. Error	95% Confidence Interval		Estimate	Std. Error	95% ...
			Lower Bound	Upper Bound			Lower Bound
0	29,169	,362	28,460	29,878	.	.	.
1	29,667	,304	29,070	30,263	.	.	.
Overall	29,211	,333	28,559	29,863	.	.	.

Means and Medians for Survival Time

Thrombopenia	Median
	95% ...
	Upper Bound
0	.
1	.
Overall	.

a. Estimation is limited to the largest survival time if it is censored.

Overall Comparisons

	Chi-Square	df	Sig.
Log Rank (Mantel-Cox)	,262	1	,609
Breslow (Generalized Wilcoxon)	,204	1	,652

Test of equality of survival distributions for the different levels of Thrombopenia.

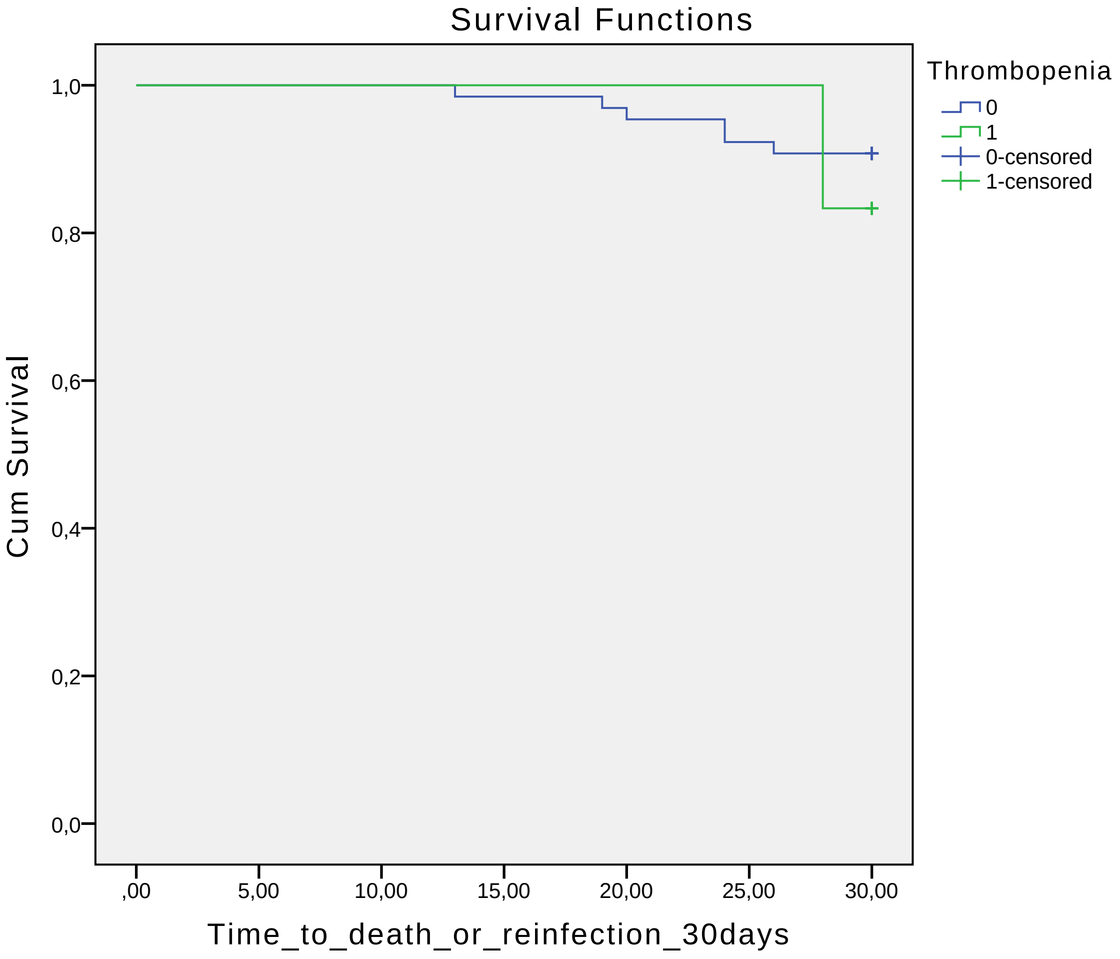

Kaplan-Meier

Case Processing Summary

Renal_failure	Total N	N of Events	Censored	
			N	Percent
0	40	5	35	87,5%
1	31	2	29	93,5%
Overall	71	7	64	90,1%

Means and Medians for Survival Time

Renal_failure	Mean[a]				Median		
			95% Confidence Interval				95% …
	Estimate	Std. Error	Lower Bound	Upper Bound	Estimate	Std. Error	Lower Bound
0	29,075	,414	28,263	29,887	.	.	.
1	29,387	,541	28,327	30,448	.	.	.
Overall	29,211	,333	28,559	29,863	.	.	.

Means and Medians for Survival Time

Renal_failure	Median
	95% ...
	Upper Bound
0	.
1	.
Overall	.

a. Estimation is limited to the largest survival time if it is censored.

Overall Comparisons

	Chi-Square	df	Sig.
Log Rank (Mantel-Cox)	,702	1	,402
Breslow (Generalized Wilcoxon)	,704	1	,401

Test of equality of survival distributions for the different levels of Renal_failure.

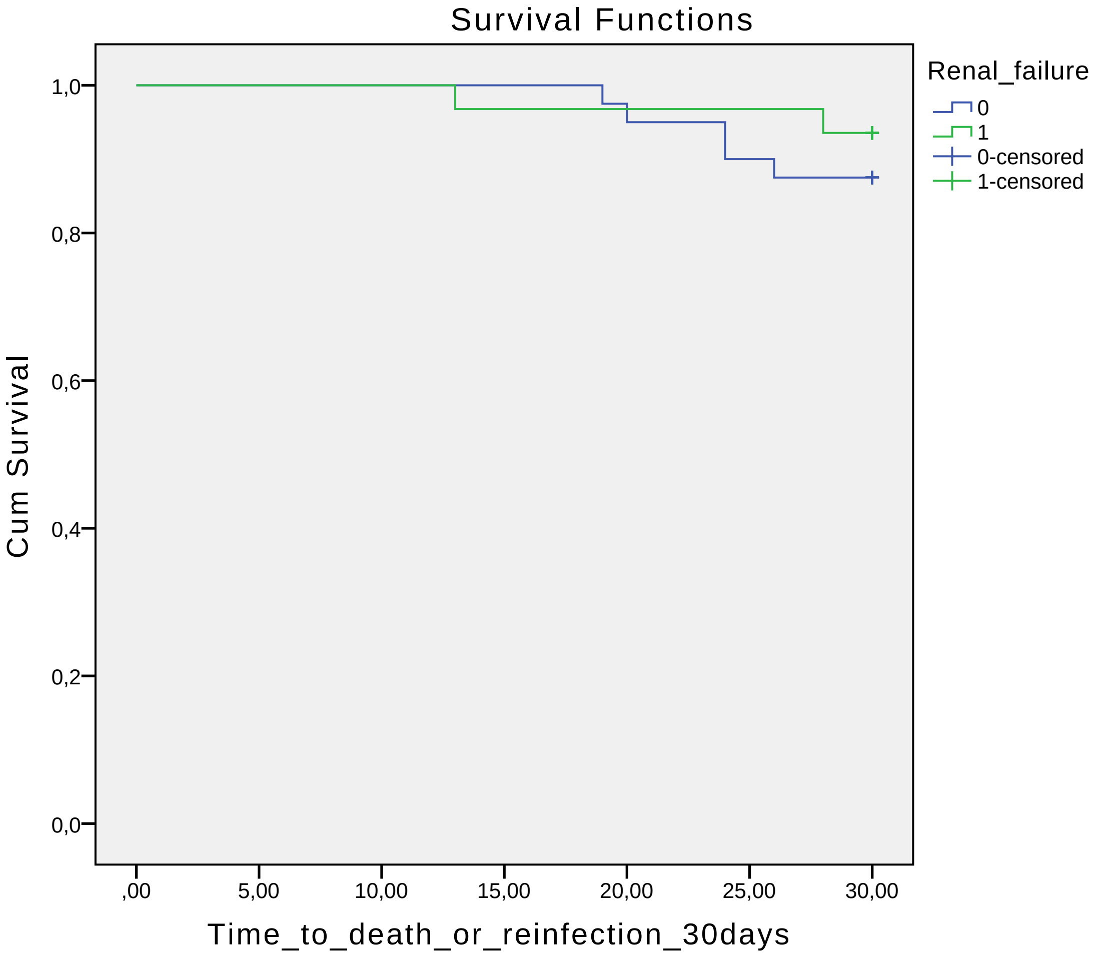

Kaplan-Meier

Case Processing Summary

Recurrent_UTI	Total N	N of Events	Censored	
			N	Percent
0	60	6	54	90,0%
1	14	1	13	92,9%
Overall	74	7	67	90,5%

Means and Medians for Survival Time

Recurrent_UTI	Mean[a]				Median		
	Estimate	Std. Error	95% Confidence Interval		Estimate	Std. Error	95% ...
			Lower Bound	Upper Bound			Lower Bound
0	29,133	,388	28,374	29,893	.	.	.
1	29,714	,275	29,175	30,254	.	.	.
Overall	29,243	,320	28,617	29,870	.	.	.

Means and Medians for Survival Time

Recurrent_UTI	Median
	95% ...
	Upper Bound
0	.
1	.
Overall	.

a. Estimation is limited to the largest survival time if it is censored.

Overall Comparisons

	Chi-Square	df	Sig.
Log Rank (Mantel-Cox)	,121	1	,728
Breslow (Generalized Wilcoxon)	,142	1	,707

Test of equality of survival distributions for the different levels of Recurrent_UTI.

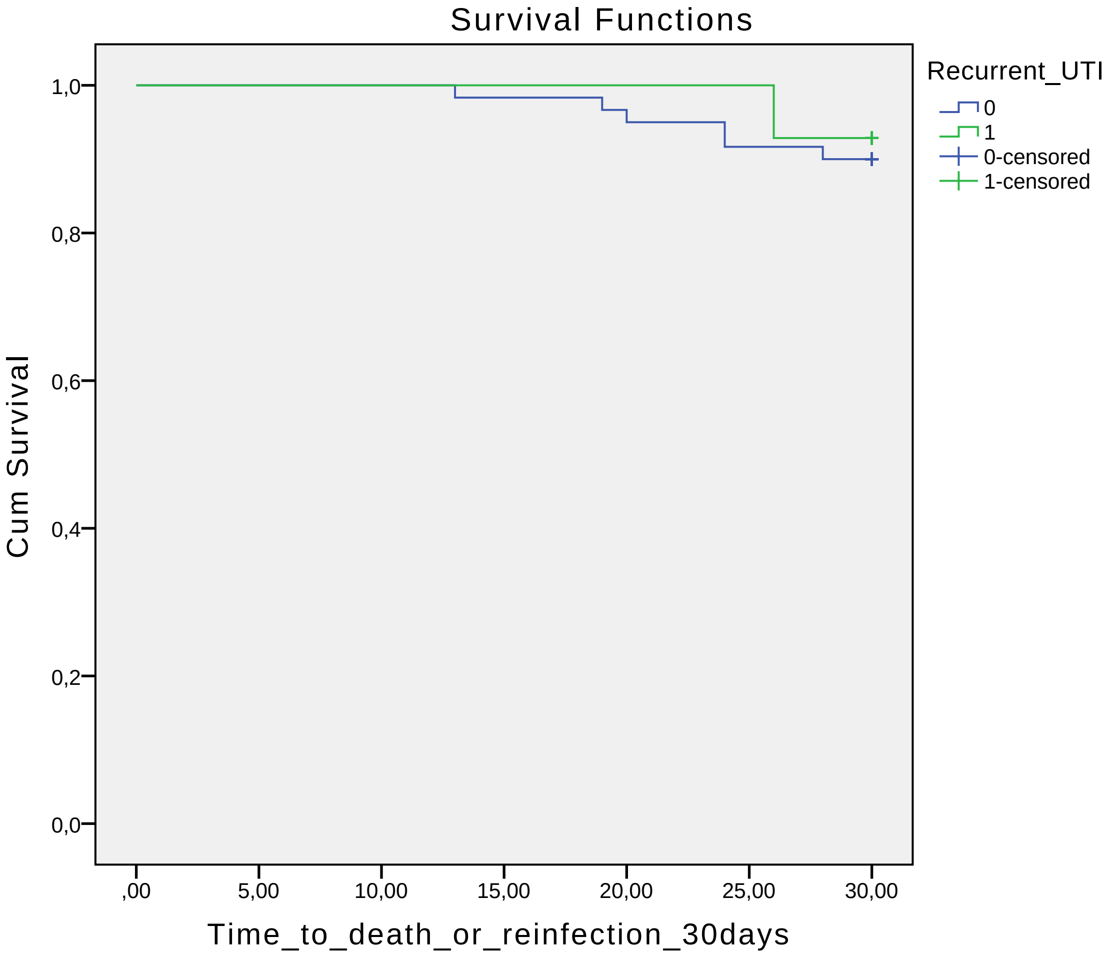

Kaplan-Meier

Case Processing Summary

Community	Total N	N of Events	Censored	
			N	Percent
0	43	5	38	88,4%
1	32	2	30	93,8%
Overall	75	7	68	90,7%

Means and Medians for Survival Time

Community	Mean[a]				Median			
	Estimate	Std. Error	95% Confidence Interval		Estimate	Std. Error	95% Confidence Interval	
			Lower Bound	Upper Bound			Lower Bound	Upper Bound
0	28,930	,520	27,910	29,950	.	.	.	.
1	29,688	,218	29,259	30,116	.	.	.	.
Overall	29,253	,316	28,635	29,872	.	.	.	.

a. Estimation is limited to the largest survival time if it is censored.

Overall Comparisons

	Chi-Square	df	Sig.
Log Rank (Mantel-Cox)	,660	1	,417
Breslow (Generalized Wilcoxon)	,698	1	,403

Test of equality of survival distributions for the different levels of Community.

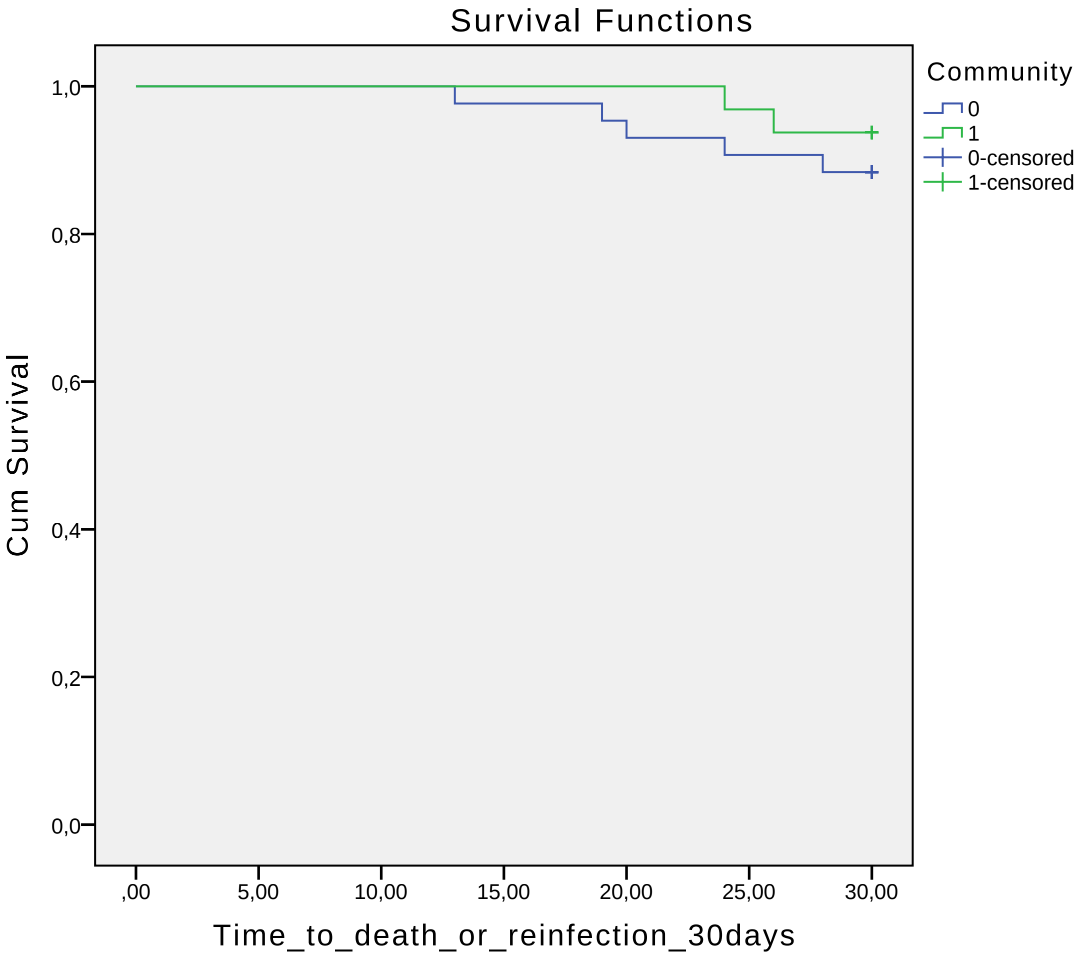

Kaplan-Meier

Case Processing Summary

Healthcare_associated	Total N	N of Events	Censored	
			N	Percent
0	41	4	37	90,2%
1	34	3	31	91,2%
Overall	75	7	68	90,7%

Means and Medians for Survival Time

Healthcare_associated	Mean[a]				Median		
			95% Confidence Interval				95% …
	Estimate	Std. Error	Lower Bound	Upper Bound	Estimate	Std. Error	Lower Bound
0	29,341	,337	28,680	30,003	.	.	.
1	29,147	,564	28,041	30,253	.	.	.
Overall	29,253	,316	28,635	29,872	.	.	.

Means and Medians for Survival Time

Healthcare_associated	Median
	95% …
	Upper Bound
0	.
1	.
Overall	.

a. Estimation is limited to the largest survival time if it is censored.

Overall Comparisons

	Chi-Square	df	Sig.
Log Rank (Mantel-Cox)	,015	1	,902
Breslow (Generalized Wilcoxon)	,013	1	,908

Test of equality of survival distributions for the different levels of Healthcare_associated.

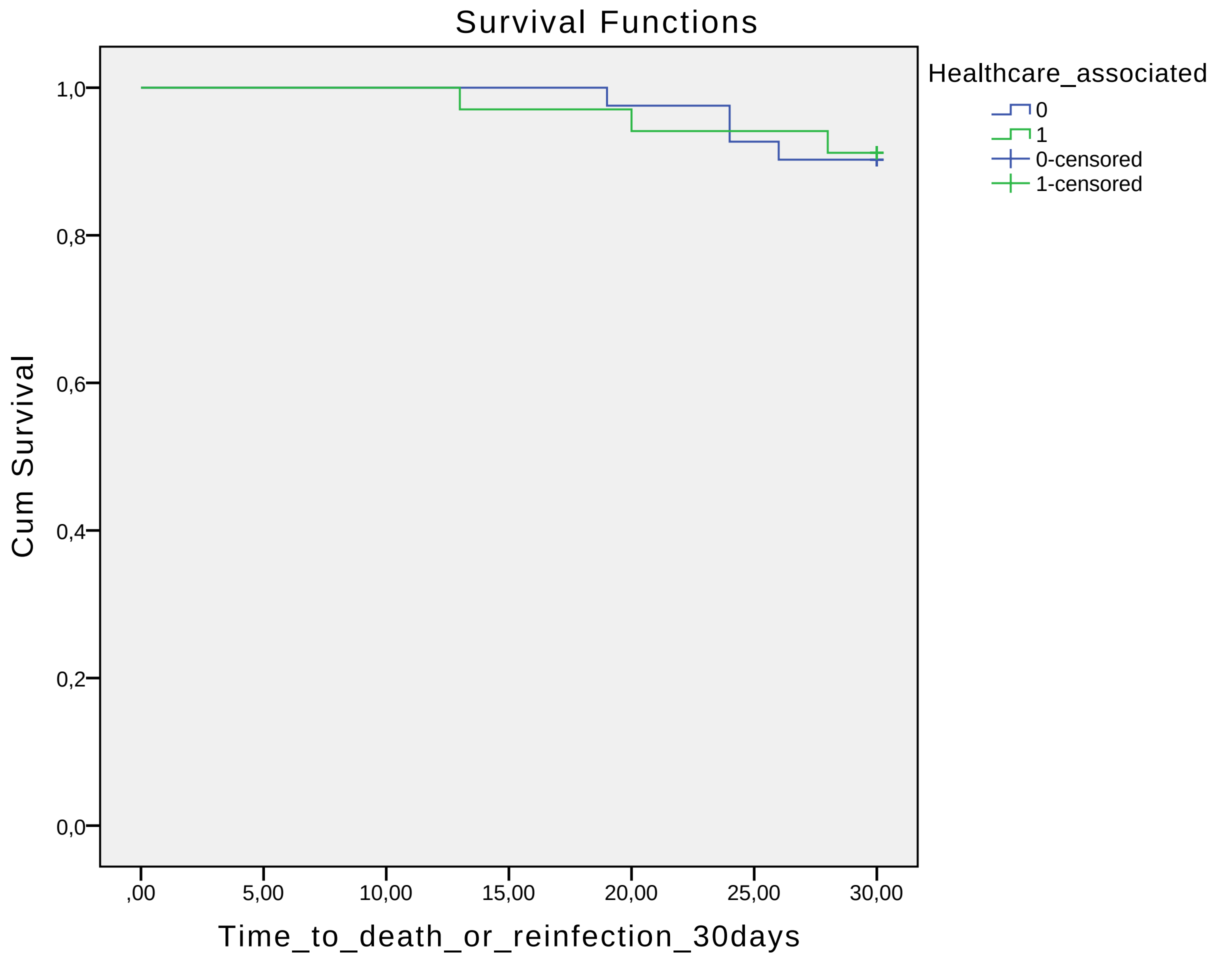

Kaplan-Meier

Case Processing Summary

Nosocomial	Total N	N of Events	Censored	
			N	Percent
0	66	5	61	92,4%
1	9	2	7	77,8%
Overall	75	7	68	90,7%

Means and Medians for Survival Time

Nosocomial	Mean[a]				Median			
	Estimate	Std. Error	95% Confidence Interval		Estimate	Std. Error	95% Confidence Interval	
			Lower Bound	Upper Bound			Lower Bound	Upper Bound
0	29,409	,311	28,799	30,019	.	.	.	.
1	28,111	1,242	25,677	30,545	.	.	.	.
Overall	29,253	,316	28,635	29,872	.	.	.	.

a. Estimation is limited to the largest survival time if it is censored.

Overall Comparisons

	Chi-Square	df	Sig.
Log Rank (Mantel-Cox)	2,188	1	,139
Breslow (Generalized Wilcoxon)	2,260	1	,133

Test of equality of survival distributions for the different levels of Nosocomial.

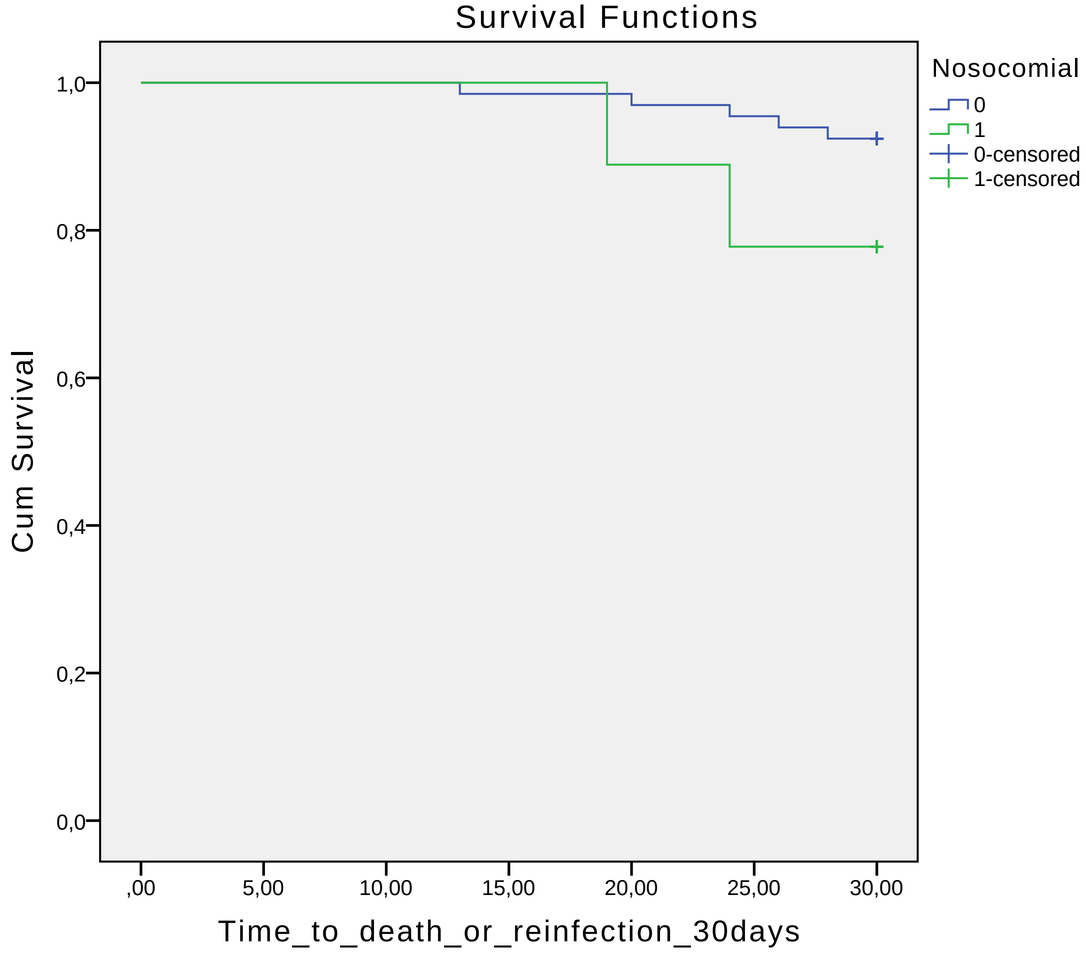

Kaplan-Meier

Case Processing Summary

Nursing_home	Total N	N of Events	Censored	
			N	Percent
0	68	6	62	91,2%
1	7	1	6	85,7%
Overall	75	7	68	90,7%

Means and Medians for Survival Time

Nursing_home	Mean[a]				Median		
			95% Confidence Interval				95% …
	Estimate	Std. Error	Lower Bound	Upper Bound	Estimate	Std. Error	Lower Bound
0	29,206	,346	28,527	29,885	.	.	.
1	29,714	,265	29,196	30,233	.	.	.
Overall	29,253	,316	28,635	29,872	.	.	.

Means and Medians for Survival Time

Nursing_home	Median
	95% …
	Upper Bound
0	.
1	.
Overall	.

a. Estimation is limited to the largest survival time if it is censored.

Overall Comparisons

	Chi-Square	df	Sig.
Log Rank (Mantel-Cox)	,168	1	,682
Breslow (Generalized Wilcoxon)	,126	1	,723

Test of equality of survival distributions for the different levels of Nursing_home.

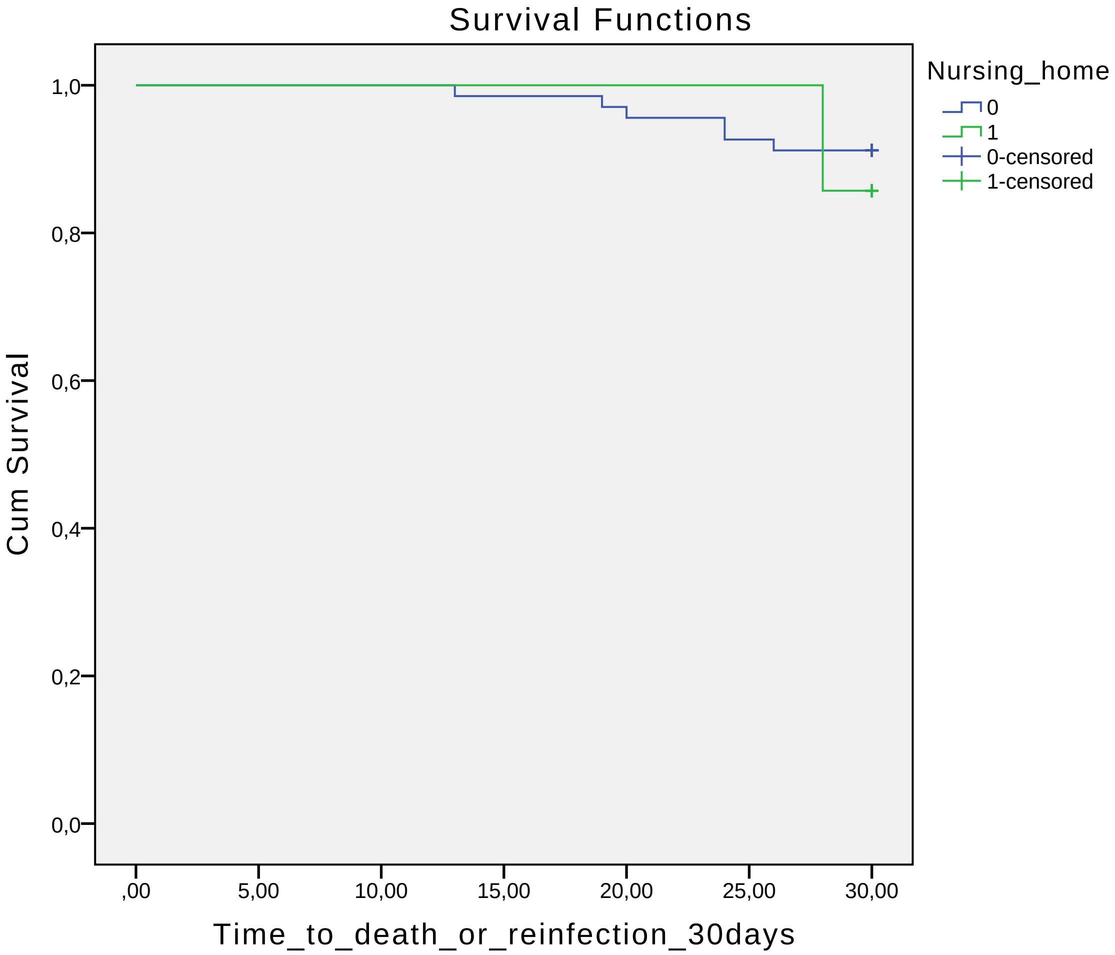

Kaplan-Meier

Case Processing Summary

Previous_ESBL_UTI	Total N	N of Events	Censored	
			N	Percent
0	61	6	55	90,2%
1	13	1	12	92,3%
Overall	74	7	67	90,5%

Means and Medians for Survival Time

Previous_ESBL_UTI	Mean[a]				Median		
			95% Confidence Interval				95% ...
	Estimate	Std. Error	Lower Bound	Upper Bound	Estimate	Std. Error	Lower Bound
0	29,148	,382	28,400	29,895	.	.	.
1	29,692	,296	29,113	30,272	.	.	.
Overall	29,243	,320	28,617	29,870	.	.	.

Means and Medians for Survival Time

Previous_ESBL_UTI	Median
	95% ...
	Upper Bound
0	.
1	.
Overall	.

a. Estimation is limited to the largest survival time if it is censored.

Overall Comparisons

	Chi-Square	df	Sig.
Log Rank (Mantel-Cox)	,068	1	,794
Breslow (Generalized Wilcoxon)	,084	1	,771

Test of equality of survival distributions for the different levels of Previous_ESBL_UTI.

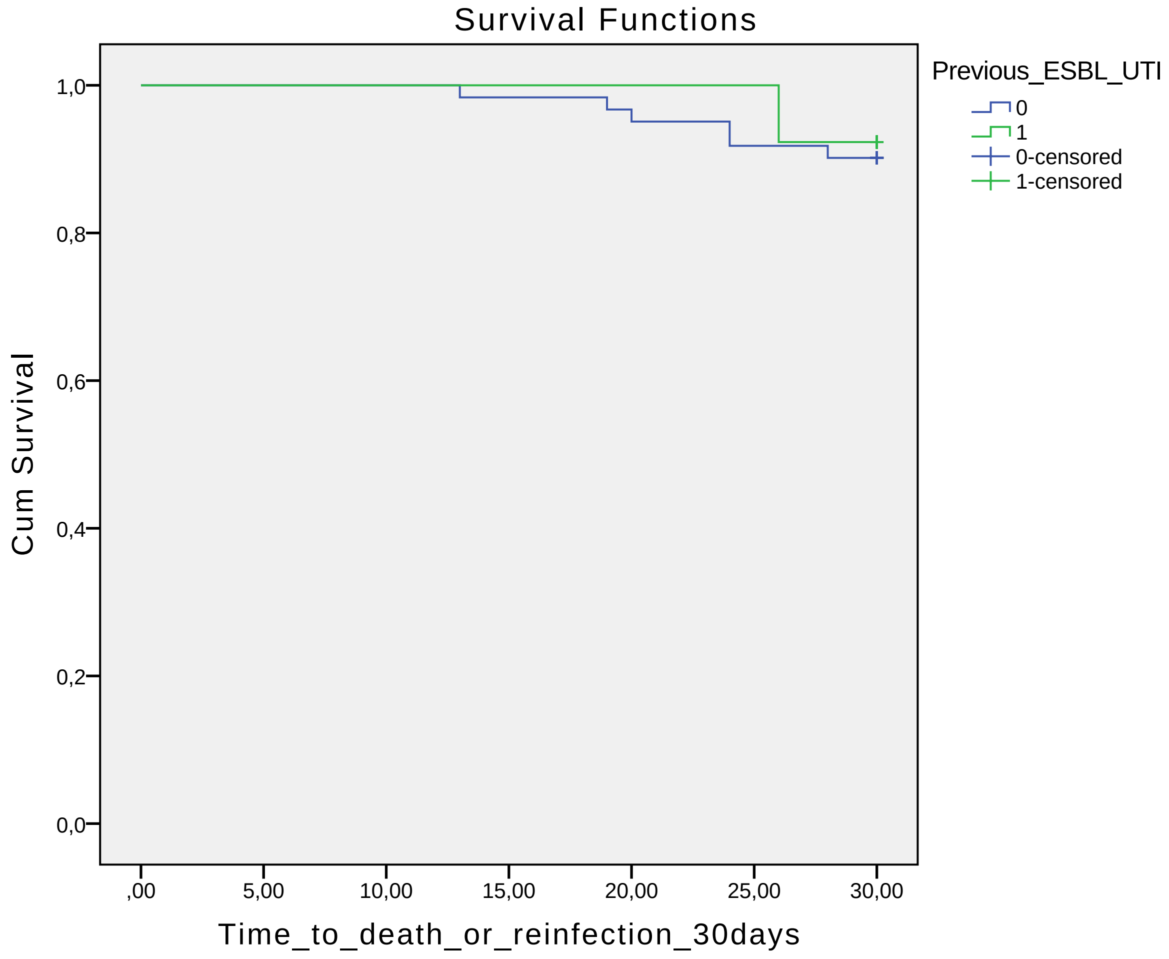

Kaplan-Meier

Case Processing Summary

Carbapenem	Total N	N of Events	Censored	
			N	Percent
,00	39	4	35	89,7%
1,00	36	3	33	91,7%
Overall	75	7	68	90,7%

Means and Medians for Survival Time

Carbapenem	Mean[a]				Median		
			95% Confidence Interval				95% ...
	Estimate	Std. Error	Lower Bound	Upper Bound	Estimate	Std. Error	Lower Bound
,00	29,256	,397	28,479	30,034	.	.	.
1,00	29,250	,497	28,275	30,225	.	.	.
Overall	29,253	,316	28,635	29,872	.	.	.

Means and Medians for Survival Time

Carbapenem	Median
	95% ...
	Upper Bound
,00	.
1,00	.
Overall	.

a. Estimation is limited to the largest survival time if it is censored.

Overall Comparisons

	Chi-Square	df	Sig.
Log Rank (Mantel-Cox)	,078	1	,780
Breslow (Generalized Wilcoxon)	,075	1	,785

Test of equality of survival distributions for the different levels of Carbapenem.

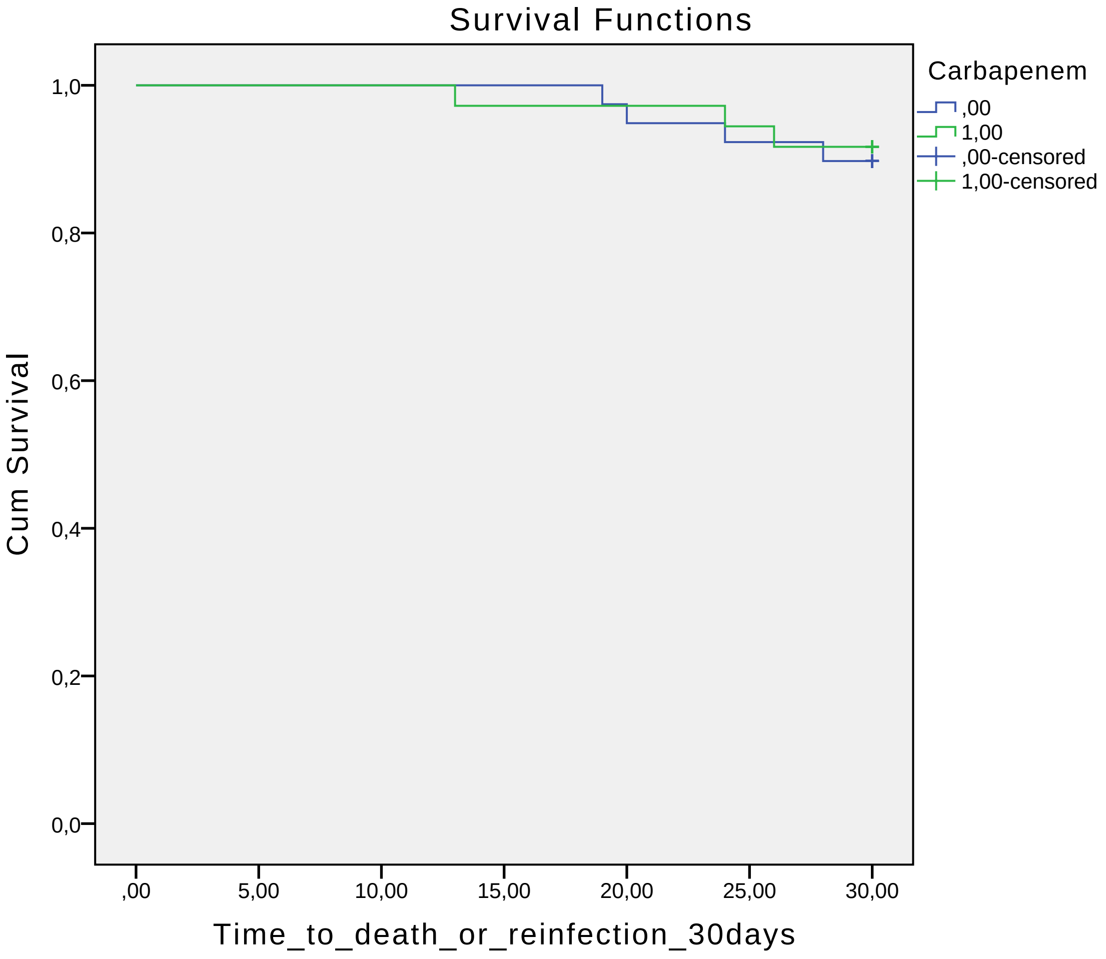

Kaplan-Meier

Case Processing Summary

Betalactam_betalactamase_inhibitor	Total N	N of Events	Censored	
			N	Percent
,00	70	5	65	92,9%
1,00	5	2	3	60,0%
Overall	75	7	68	90,7%

Means and Medians for Survival Time

Betalactam_betalactamase_inhibitor	Mean[a]				Median		
	Estimate	Std. Error	95% Confidence Interval		Estimate	Std. Error	95% …
			Lower Bound	Upper Bound			Lower Bound
,00	29,443	,294	28,867	30,019	.	.	.
1,00	26,600	1,992	22,696	30,504	.	.	.
Overall	29,253	,316	28,635	29,872	.	.	.

Means and Medians for Survival Time

Betalactam_betalactamase_inhibitor	Median
	95% …
	Upper Bound
,00	.
1,00	.
Overall	.

a. Estimation is limited to the largest survival time if it is censored.

Overall Comparisons

	Chi-Square	df	Sig.
Log Rank (Mantel-Cox)	7,170	1	,007
Breslow (Generalized Wilcoxon)	7,304	1	,007

Test of equality of survival distributions for the different levels of Betalactam_betalactamase_inhibitor.

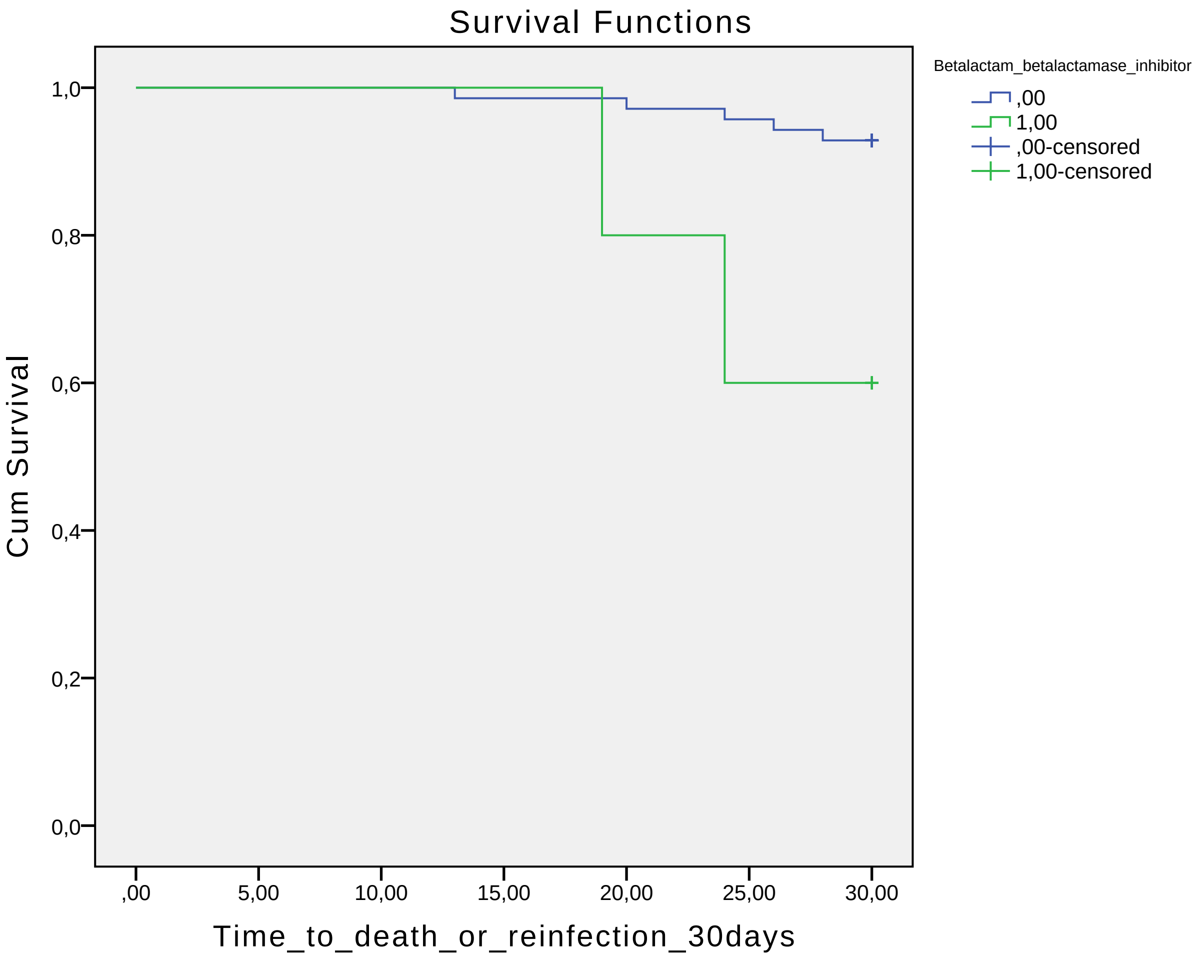

Kaplan-Meier

Warnings

No statistics are computed because all cases are censored.

Case Processing Summary

Quinolone	Total N	N of Events	Censored	
			N	Percent
,00	63	7	56	88,9%
1,00	12	0	12	100,0%
Overall	75	7	68	90,7%

Overall Comparisons

	Chi-Square	df	Sig.
Log Rank (Mantel-Cox)	1,404	1	,236
Breslow (Generalized Wilcoxon)	1,402	1	,236

Test of equality of survival distributions for the different levels of Quinolone.

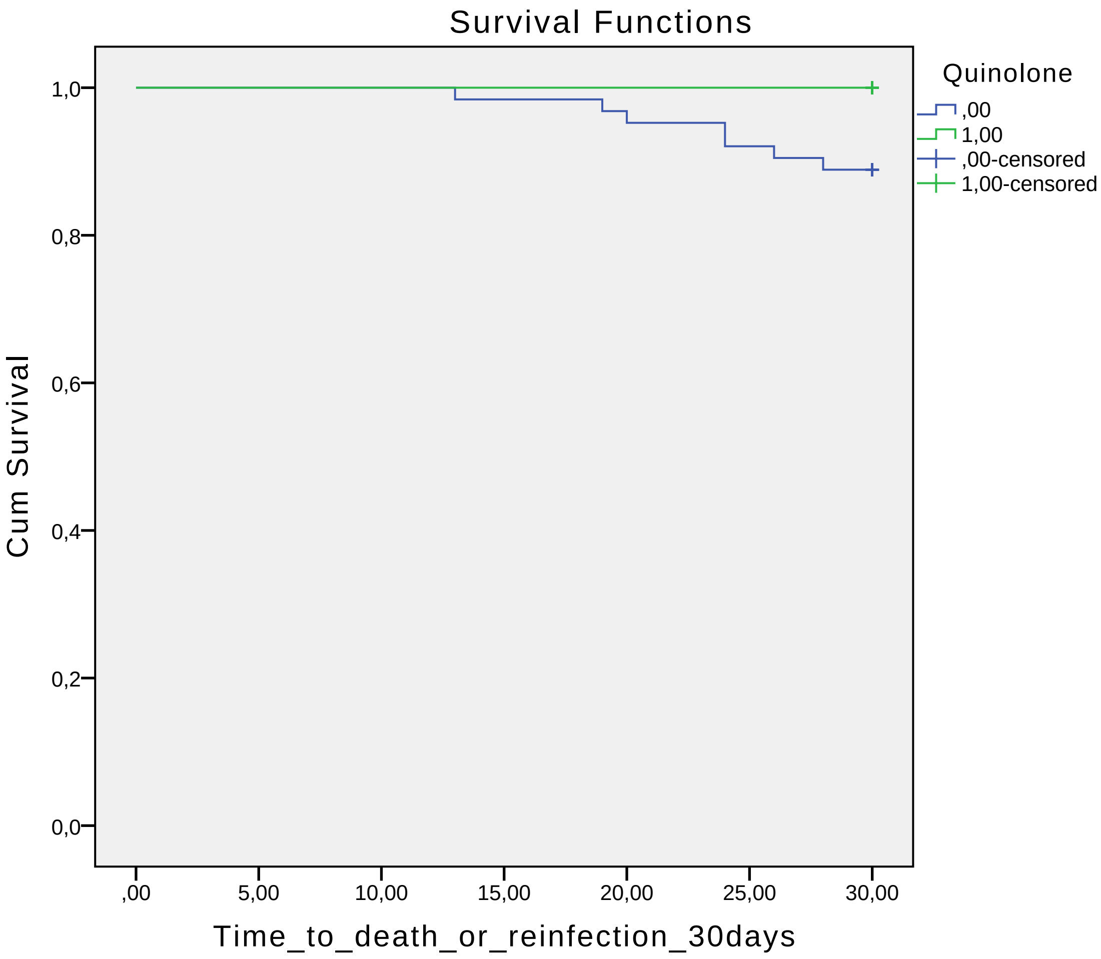

Kaplan-Meier

Case Processing Summary

Trimethoprim_sulfamethoxazole	Total N	N of Events	Censored	
			N	Percent
,00	68	6	62	91,2%
1,00	7	1	6	85,7%
Overall	75	7	68	90,7%

Means and Medians for Survival Time

Trimethoprim_sulfamethoxazole	Mean[a]				Median		
			95% Confidence Interval				95% …
	Estimate	Std. Error	Lower Bound	Upper Bound	Estimate	Std. Error	Lower Bound
,00	29,324	,319	28,698	29,949	.	.	.
1,00	28,571	1,323	25,979	31,164	.	.	.
Overall	29,253	,316	28,635	29,872	.	.	.

Means and Medians for Survival Time

Trimethoprim_sulfamethoxazole	Median
	95% …
	Upper Bound
,00	.
1,00	.
Overall	.

a. Estimation is limited to the largest survival time if it is censored.

Overall Comparisons

	Chi-Square	df	Sig.
Log Rank (Mantel-Cox)	,251	1	,616
Breslow (Generalized Wilcoxon)	,266	1	,606

Test of equality of survival distributions for the different levels of Trimethoprim_sulfamethoxazole.

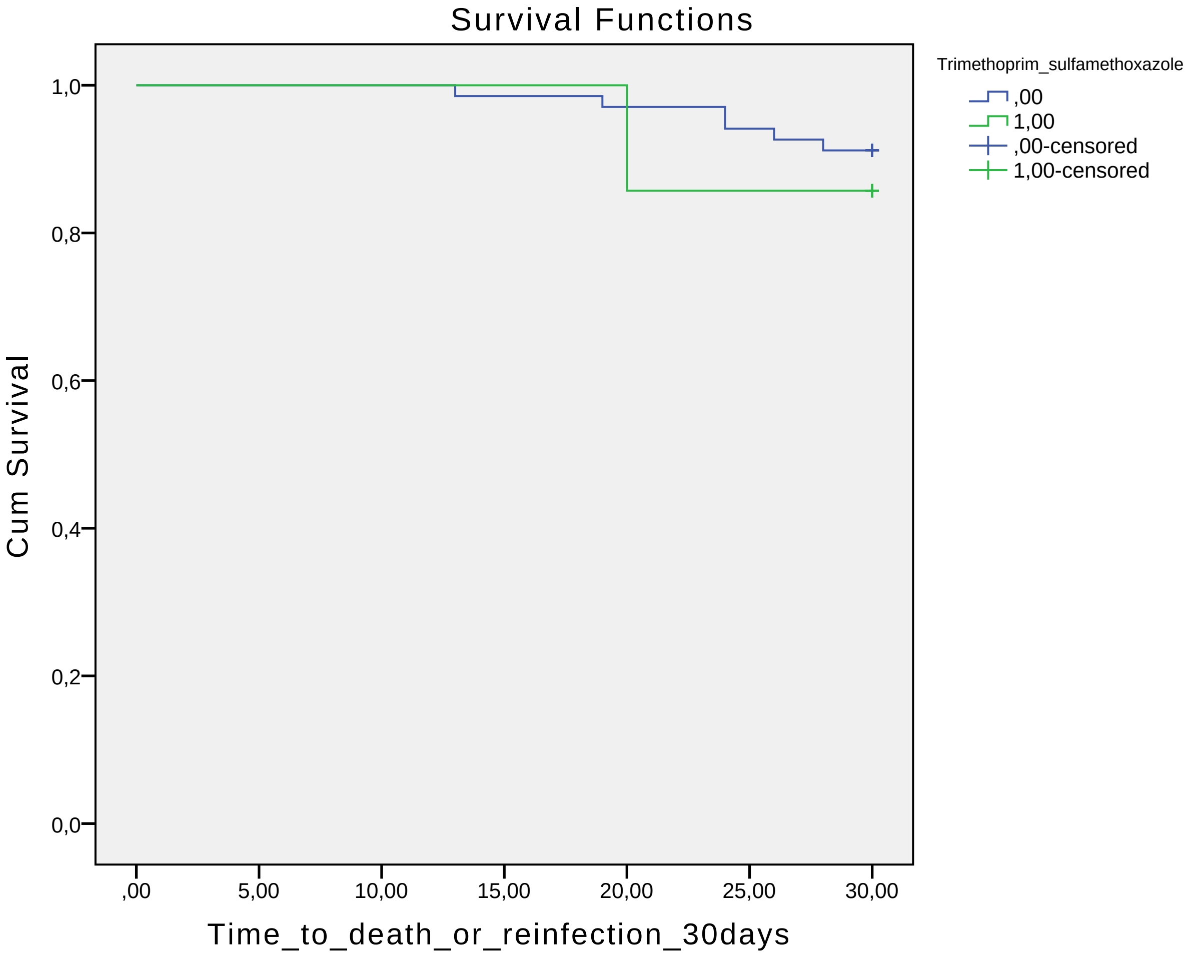

Kaplan-Meier

Case Processing Summary

Phosfomycin	Total N	N of Events	Censored	
			N	Percent
,00	62	6	56	90,3%
1,00	13	1	12	92,3%
Overall	75	7	68	90,7%

Means and Medians for Survival Time

Phosfomycin	Mean[a]				Median		
			95% Confidence Interval				95% ...
	Estimate	Std. Error	Lower Bound	Upper Bound	Estimate	Std. Error	Lower Bound
,00	29,129	,379	28,387	29,871	.	.	.
1,00	29,846	,148	29,556	30,136	.	.	.
Overall	29,253	,316	28,635	29,872	.	.	.

Means and Medians for Survival Time

Phosfomycin	Median 95% ... Upper Bound
,00	.
1,00	.
Overall	.

a. Estimation is limited to the largest survival time if it is censored.

Overall Comparisons

	Chi-Square	df	Sig.
Log Rank (Mantel-Cox)	,067	1	,796
Breslow (Generalized Wilcoxon)	,090	1	,764

Test of equality of survival distributions for the different levels of Phosfomycin.

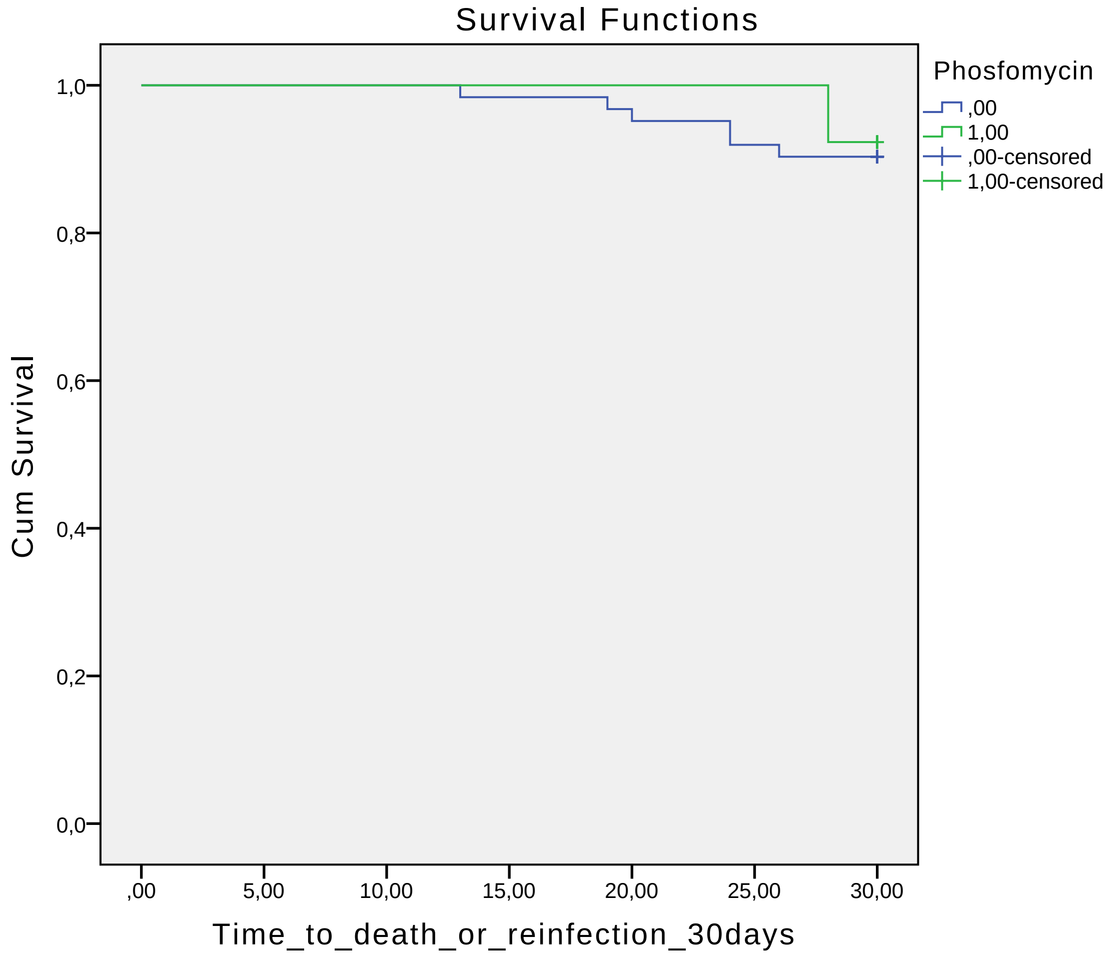

Kaplan-Meier

Case Processing Summary

Phosfomycin	Total N	N of Events	Censored N	Censored Percent
,00	62	6	56	90,3%
1,00	13	1	12	92,3%
Overall	75	7	68	90,7%

Means and Medians for Survival Time

Phosfomycin	Mean[a] Estimate	Std. Error	95% Confidence Interval Lower Bound	Upper Bound	Median Estimate	Std. Error	95% … Lower Bound
,00	29,129	,379	28,387	29,871	.	.	.
1,00	29,846	,148	29,556	30,136	.	.	.
Overall	29,253	,316	28,635	29,872	.	.	.

Means and Medians for Survival Time

Phosfomycin	Median 95% … Upper Bound
,00	.
1,00	.
Overall	.

a. Estimation is limited to the largest survival time if it is censored.

Overall Comparisons

	Chi-Square	df	Sig.
Log Rank (Mantel-Cox)	,067	1	,796
Breslow (Generalized Wilcoxon)	,090	1	,764

Test of equality of survival distributions for the different levels of Phosfomycin.

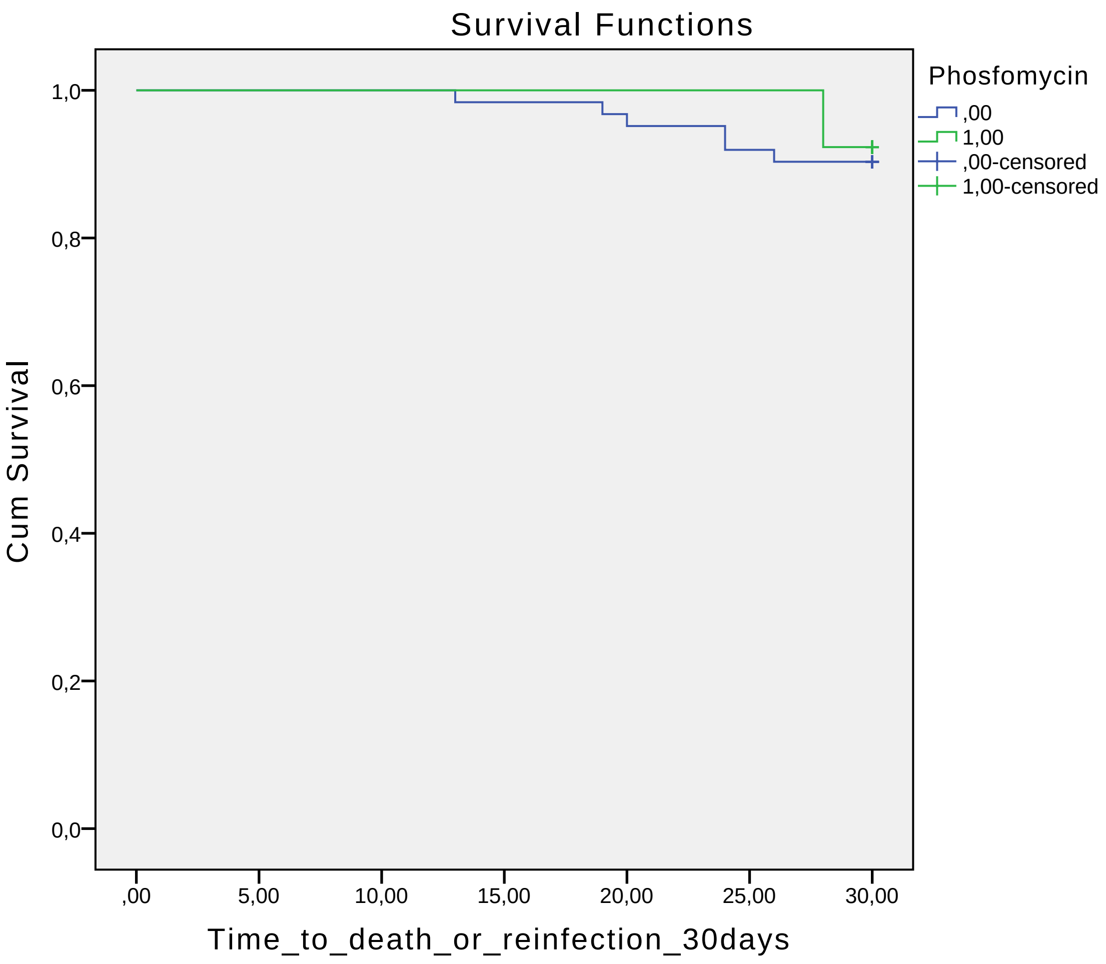

Kaplan-Meier

Warnings

No statistics are computed because all cases are censored.

Case Processing Summary

Furantoin	Total N	N of Events	Censored	
			N	Percent
,00	73	7	66	90,4%
1,00	2	0	2	100,0%
Overall	75	7	68	90,7%

Overall Comparisons

	Chi-Square	df	Sig.
Log Rank (Mantel-Cox)	,201	1	,654
Breslow (Generalized Wilcoxon)	,200	1	,654

Test of equality of survival distributions for the different levels of Furantoin.

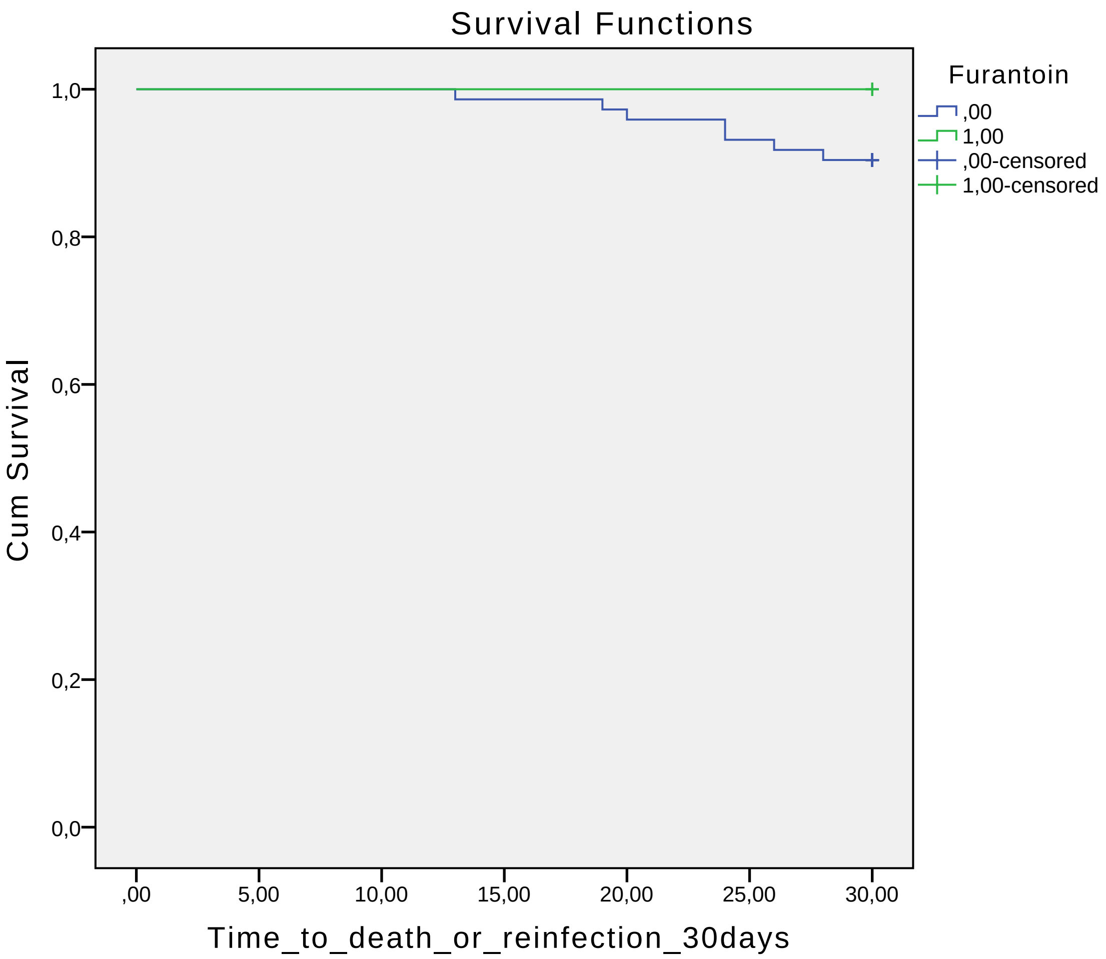

Kaplan-Meier

Warnings

No statistics are computed because all cases are censored.

Case Processing Summary

Aminoglicosyde	Total N	N of Events	Censored	
			N	Percent
,00	74	7	67	90,5%
1,00	1	0	1	100,0%
Overall	75	7	68	90,7%

Overall Comparisons

	Chi-Square	df	Sig.
Log Rank (Mantel-Cox)	,099	1	,753
Breslow (Generalized Wilcoxon)	,099	1	,753

Test of equality of survival distributions for the different levels of Aminoglicosyde.

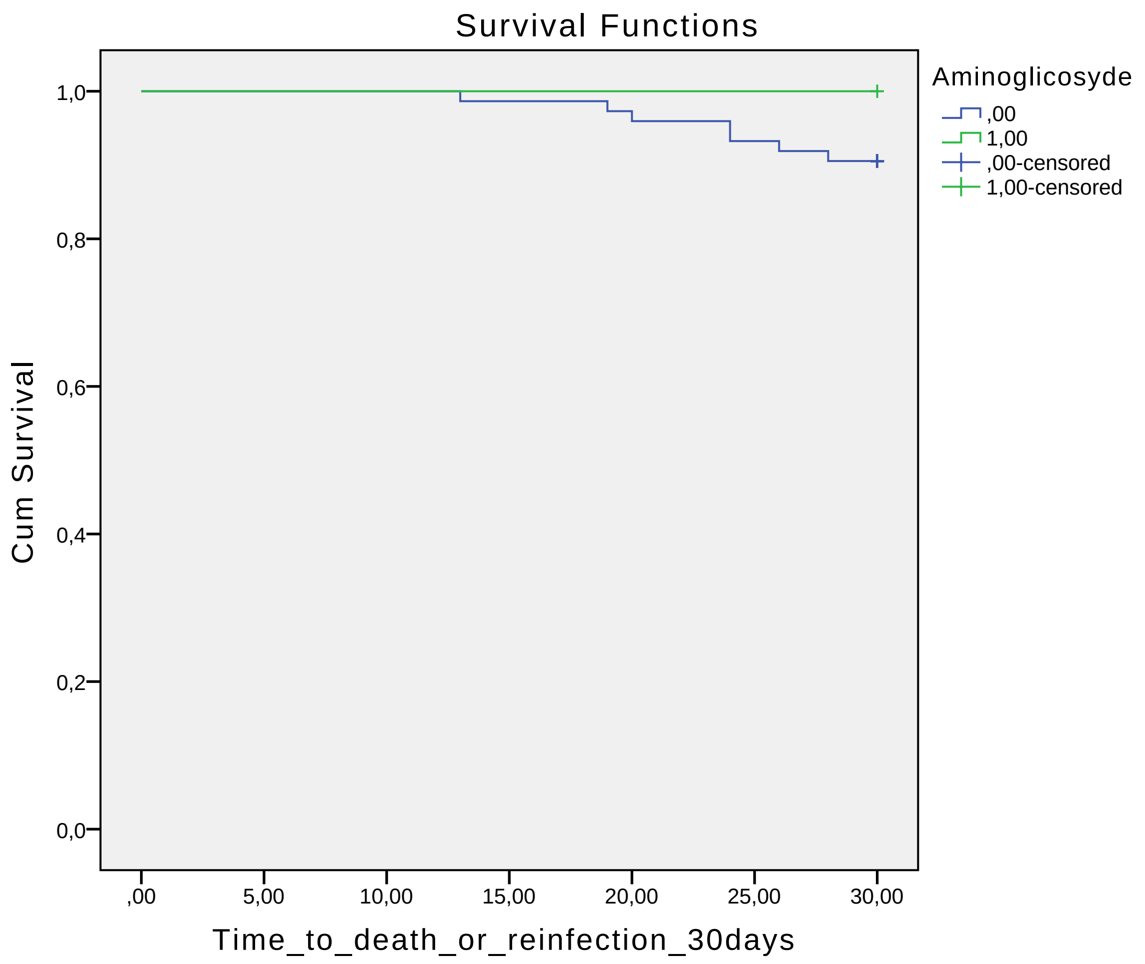

Kaplan-Meier

Case Processing Summary

Barthel_index_below_20	Total N	N of Events	Censored	
			N	Percent
,00	38	5	33	86,8%
1,00	21	1	20	95,2%
Overall	59	6	53	89,8%

Means and Medians for Survival Time

Barthel_index_below_20	Mean[a]				Median			
			95% Confidence Interval				95% ...	
	Estimate	Std. Error	Lower Bound	Upper Bound	Estimate	Std. Error	Lower Bound	Upper Bound
,00	28,737	,589	27,582	29,892	.	.	.	.
1,00	29,905	,093	29,723	30,087	.	.	.	.
Overall	29,153	,388	28,392	29,913	.	.	.	.

a. Estimation is limited to the largest survival time if it is censored.

Overall Comparisons

	Chi-Square	df	Sig.
Log Rank (Mantel-Cox)	1,084	1	,298
Breslow (Generalized Wilcoxon)	1,159	1	,282

Test of equality of survival distributions for the different levels of Barthel_index_below_20.

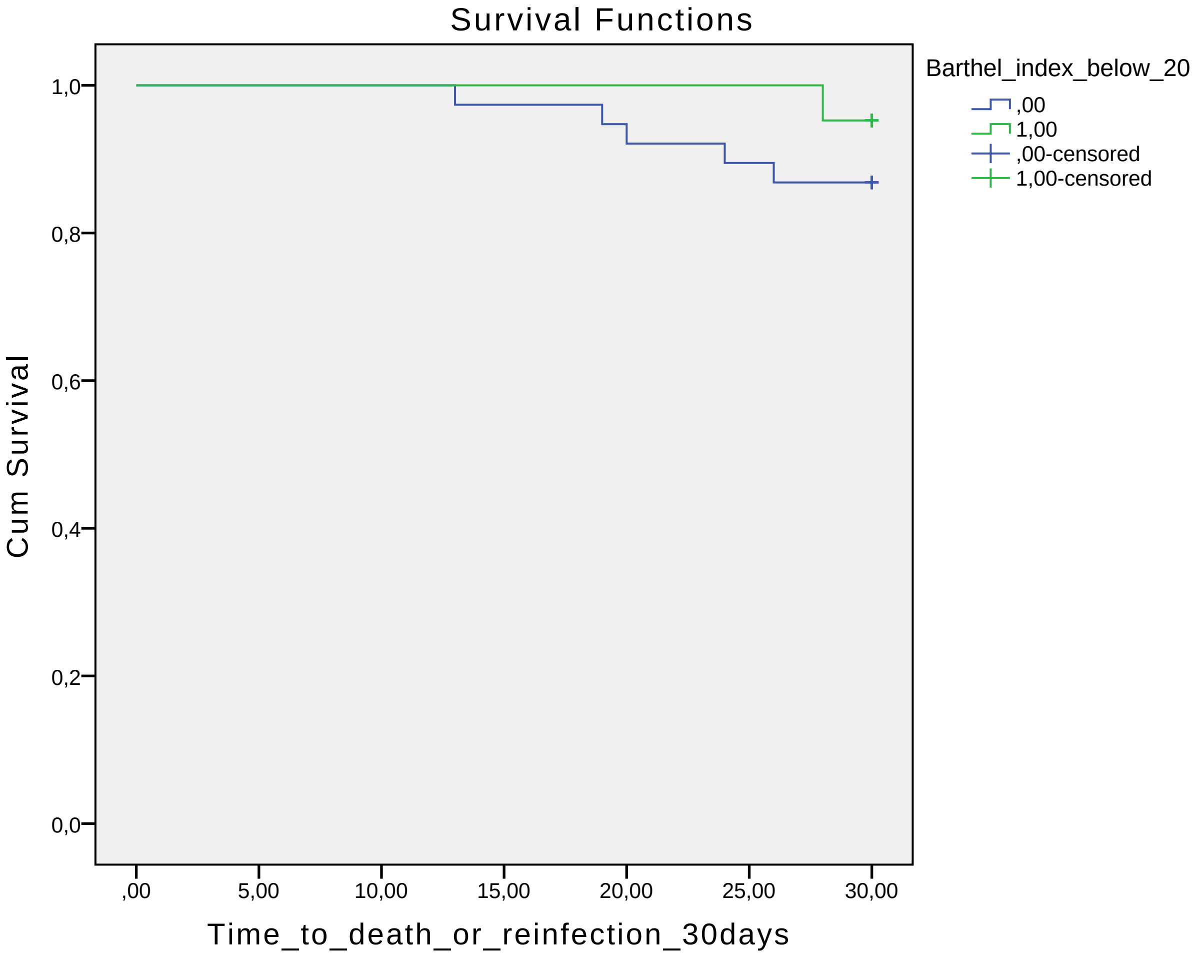

Kaplan-Meier

Case Processing Summary

Age_above_79	Total N	N of Events	Censored	
			N	Percent
,00	41	3	38	92,7%
1,00	34	4	30	88,2%
Overall	75	7	68	90,7%

Means and Medians for Survival Time

Age_above_79	Mean[a]				Median		
	Estimate	Std. Error	95% Confidence Interval		Estimate	Std. Error	95% …
			Lower Bound	Upper Bound			Lower Bound
,00	29,341	,377	28,602	30,081	.	.	.
1,00	29,147	,526	28,115	30,179	.	.	.
Overall	29,253	,316	28,635	29,872	.	.	.

Means and Medians for Survival Time

Age_above_79	Median
	95% …
	Upper Bound
,00	.
1,00	.
Overall	.

a. Estimation is limited to the largest survival time if it is censored.

Overall Comparisons

	Chi-Square	df	Sig.
Log Rank (Mantel-Cox)	,394	1	,530
Breslow (Generalized Wilcoxon)	,361	1	,548

Test of equality of survival distributions for the different levels of Age_above_79.

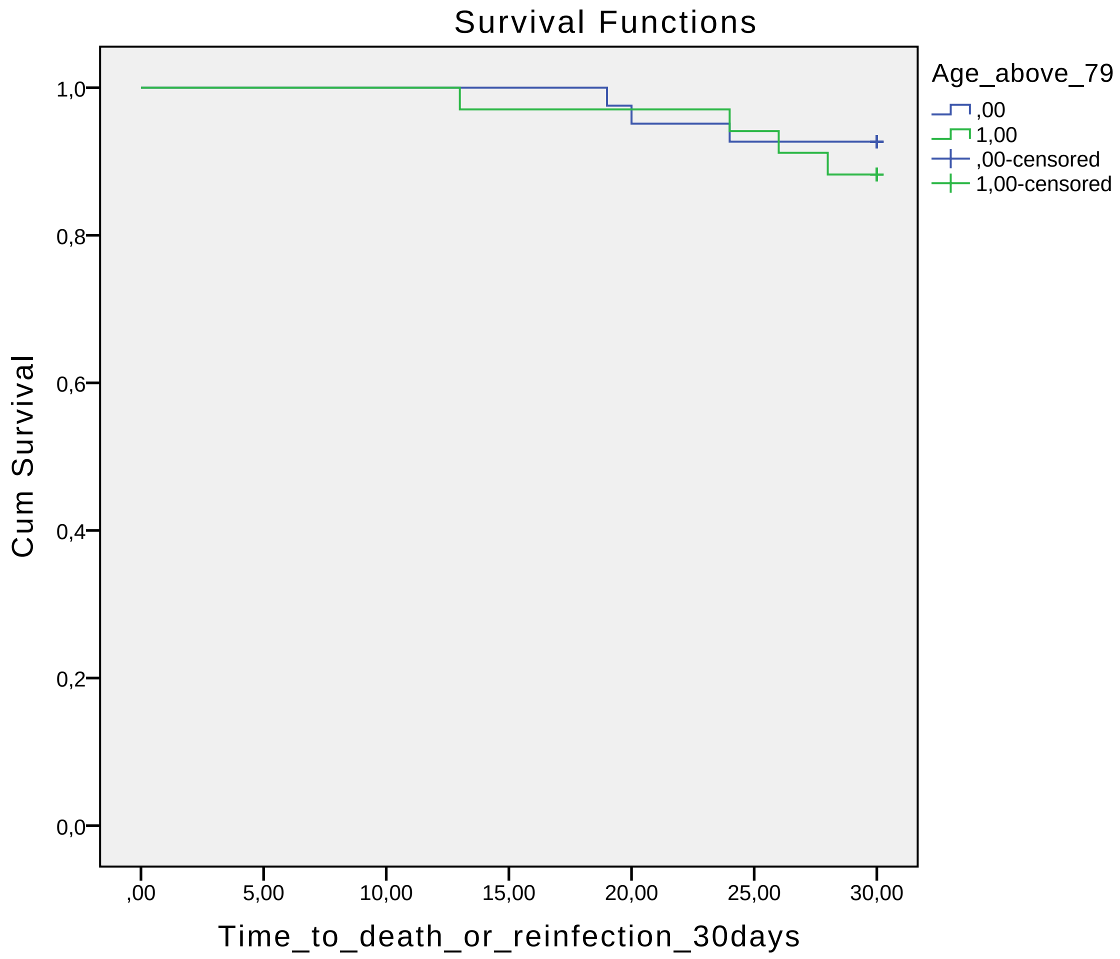

Kaplan-Meier

Case Processing Summary

Cystitis	Total N	N of Events	Censored	
			N	Percent
,00	39	2	37	94,9%
1,00	36	5	31	86,1%
Overall	75	7	68	90,7%

Means and Medians for Survival Time

Cystitis	Mean[a]				Median			
	Estimate	Std. Error	95% Confidence Interval		Estimate	Std. Error	95% Confidence Interval	
			Lower Bound	Upper Bound			Lower Bound	Upper Bound
,00	29,308	,493	28,341	30,275	.	.	.	.
1,00	29,194	,383	28,445	29,944	.	.	.	.
Overall	29,253	,316	28,635	29,872	.	.	.	.

a. Estimation is limited to the largest survival time if it is censored.

Overall Comparisons

	Chi-Square	df	Sig.
Log Rank (Mantel-Cox)	1,559	1	,212
Breslow (Generalized Wilcoxon)	1,460	1	,227

Test of equality of survival distributions for the different levels of Cystitis.

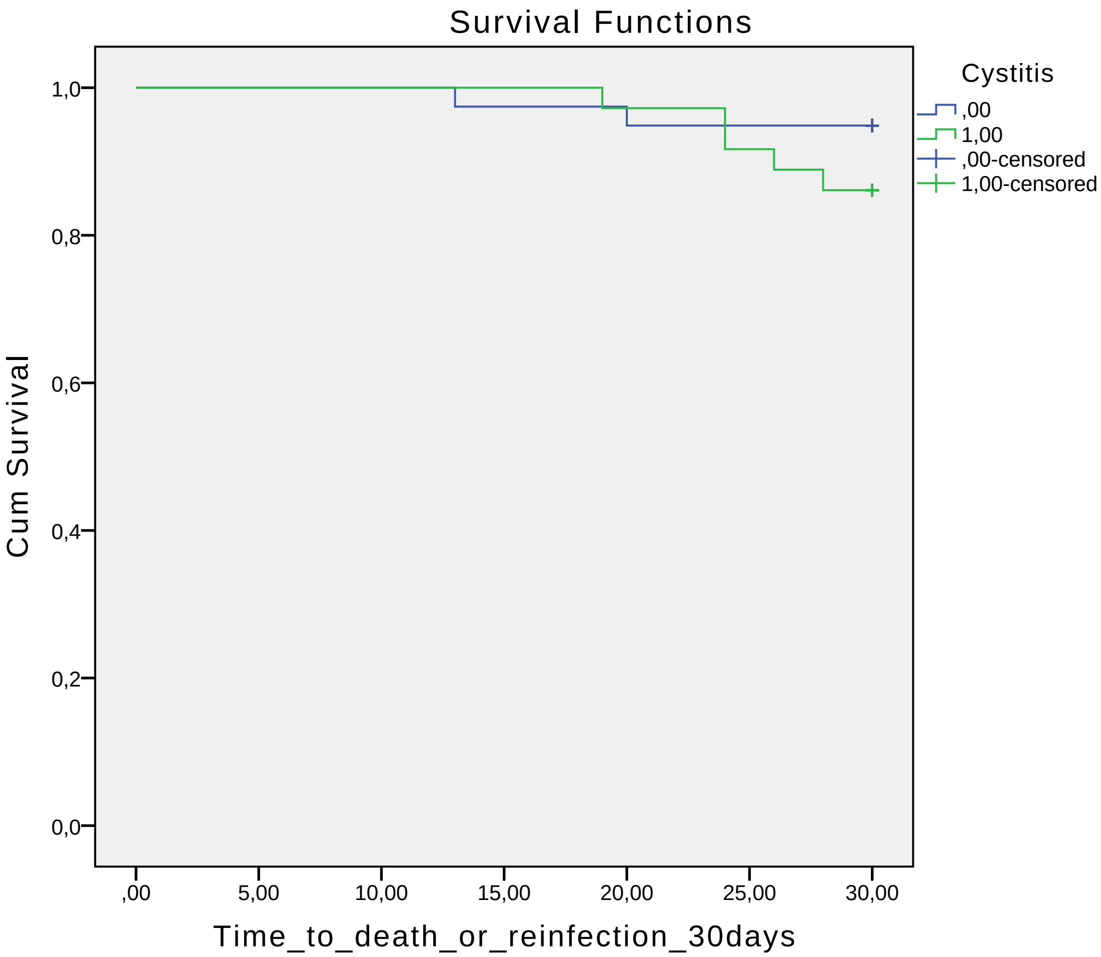

Kaplan-Meier

Case Processing Summary

Febrile_UTI	Total N	N of Events	Censored	
			N	Percent
,00	49	6	43	87,8%
1,00	26	1	25	96,2%
Overall	75	7	68	90,7%

Means and Medians for Survival Time

Febrile_UTI	Mean[a]				Median			
	Estimate	Std. Error	95% Confidence Interval		Estimate	Std. Error	95% Confidence Interval	
			Lower Bound	Upper Bound			Lower Bound	Upper Bound
,00	29,204	,343	28,532	29,876	.	.	.	.
1,00	29,346	,641	28,090	30,603	.	.	.	.
Overall	29,253	,316	28,635	29,872	.	.	.	.

a. Estimation is limited to the largest survival time if it is censored.

Overall Comparisons

	Chi-Square	df	Sig.
Log Rank (Mantel-Cox)	1,312	1	,252
Breslow (Generalized Wilcoxon)	1,238	1	,266

Test of equality of survival distributions for the different levels of Febrile_UTI.

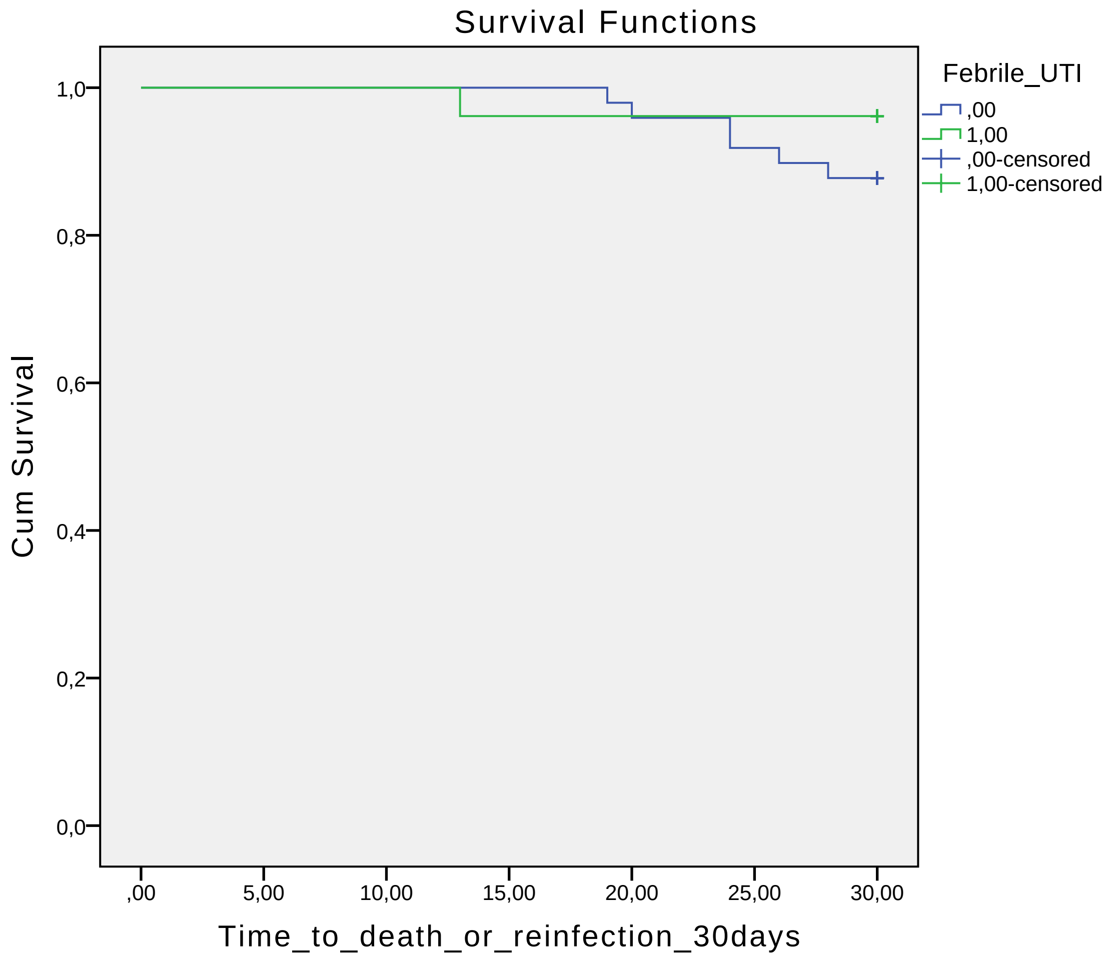

Kaplan-Meier

Case Processing Summary

Pyelonephritis	Total N	N of Events	Censored	
			N	Percent
,00	62	6	56	90,3%
1,00	13	1	12	92,3%
Overall	75	7	68	90,7%

Means and Medians for Survival Time

Pyelonephritis	Mean[a]				Median		
			95% Confidence Interval				95% …
	Estimate	Std. Error	Lower Bound	Upper Bound	Estimate	Std. Error	Lower Bound
,00	29,258	,349	28,574	29,942	.	.	.
1,00	29,231	,739	27,782	30,679	.	.	.
Overall	29,253	,316	28,635	29,872	.	.	.

Means and Medians for Survival Time

Pyelonephritis	Median
	95% …
	Upper Bound
,00	.
1,00	.
Overall	.

a. Estimation is limited to the largest survival time if it is censored.

Overall Comparisons

	Chi-Square	df	Sig.
Log Rank (Mantel-Cox)	,043	1	,836
Breslow (Generalized Wilcoxon)	,038	1	,846

Test of equality of survival distributions for the different levels of Pyelonephritis.

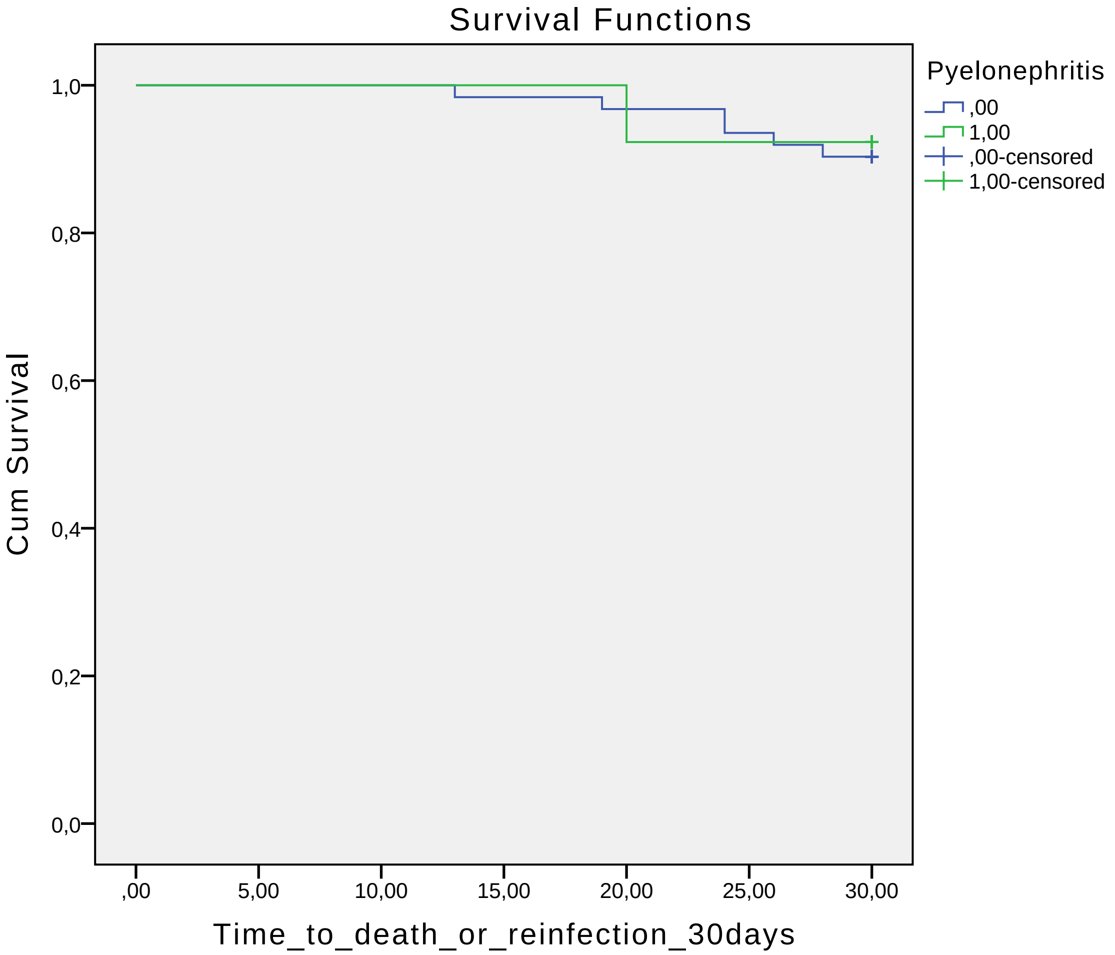

Kaplan-Meier

Sex = ,0

Case Processing Summary[a]

Short_treatment	Total N	N of Events	Censored	
			N	Percent
,00	22	2	20	90,9%
1,00	10	1	9	90,0%
Overall	32	3	29	90,6%

a. Sex = ,0

Means and Medians for Survival Time[a]

Short_treatment	Mean[b] Estimate	Std. Error	95% Confidence Interval Lower Bound	Upper Bound	Median Estimate	Std. Error	95% ... Lower Bound
,00	29,227	,545	28,159	30,296	.	.	.
1,00	28,300	1,613	25,139	31,461	.	.	.
Overall	28,938	,633	27,697	30,178	.	.	.

Means and Medians for Survival Time[a]

Short_treatment	Median 95% ... Upper Bound
,00	.
1,00	.
Overall	.

a. Sex = ,0

b. Estimation is limited to the largest survival time if it is censored.

Overall Comparisons[a]

	Chi-Square	df	Sig.
Log Rank (Mantel-Cox)	,015	1	,903
Breslow (Generalized Wilcoxon)	,026	1	,871

Test of equality of survival distributions for the different levels of Short_treatment.

a. Sex = ,0

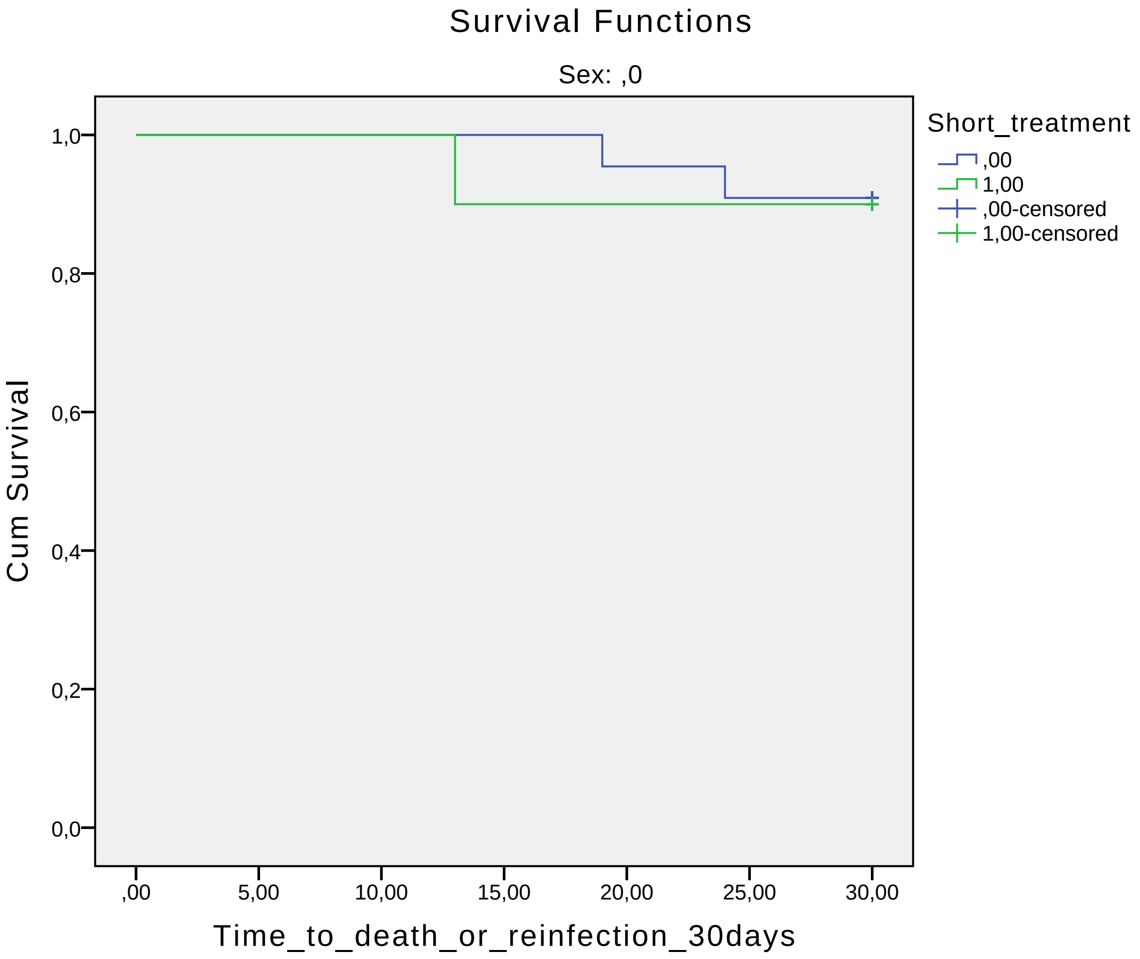

Sex = 1,0

Case Processing Summary[a]

Short_treatment	Total N	N of Events	Censored	
			N	Percent
,00	18	2	16	88,9%
1,00	25	2	23	92,0%
Overall	43	4	39	90,7%

a. Sex = 1,0

Means and Medians for Survival Time[a]

Short_treatment	Mean[b]				Median		
	Estimate	Std. Error	95% Confidence Interval		Estimate	Std. Error	95% …
			Lower Bound	Upper Bound			Lower Bound
,00	29,444	,379	28,702	30,187	.	.	.
1,00	29,520	,396	28,743	30,297	.	.	.
Overall	29,488	,280	28,940	30,037	.	.	.

Means and Medians for Survival Time[a]

Short_treatment	Median
	95% …
	Upper Bound
,00	.
1,00	.
Overall	.

a. Sex = 1,0

b. Estimation is limited to the largest survival time if it is censored.

Overall Comparisons[a]

	Chi-Square	df	Sig.
Log Rank (Mantel-Cox)	,118	1	,731
Breslow (Generalized Wilcoxon)	,117	1	,732

Test of equality of survival distributions for the different levels of Short_treatment.

a. Sex = 1,0

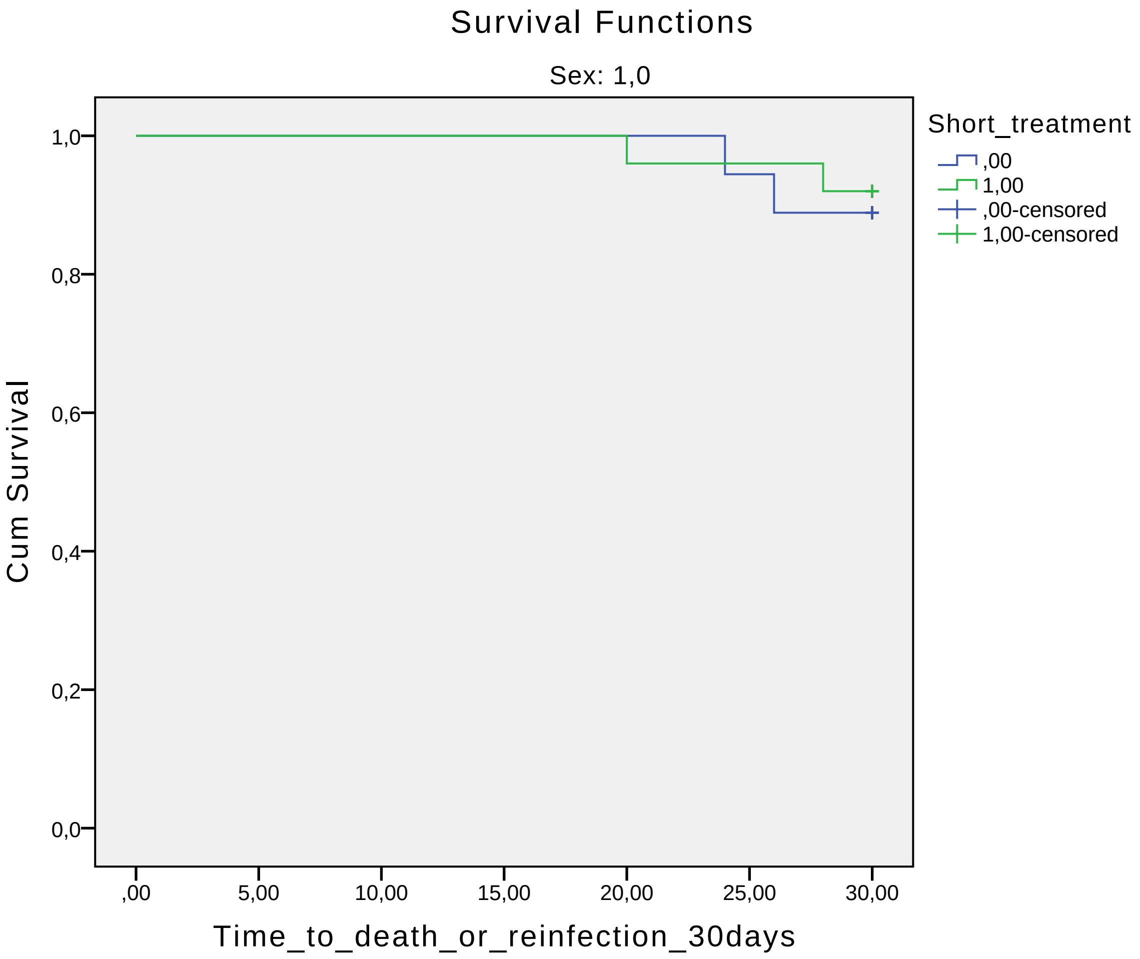
Survival Functions
Sex: 1,0
Cum Survival
1,0
0,8
0,6
0,4
0,2
0,0
,00
5,00
10,00
15,00
20,00
25,00
30,00
Time_to_death_or_reinfection_30days
Short_treatment
,00
1,00
,00-censored
1,00-censored
