# Supplementary material for: Treatment duration of complicated urinary tract infections by extended-spectrum beta-lactamases producing enterobacterales
Source: PLoS One. 2020 Oct 19;15(10):e0237365. doi: 10.1371/journal.pone.0237365 (PMC7571686; doi:10.1371/journal.pone.0237365)
Supplement: S6 File — Multivariate analysis. (PDF) [file pone.0237365.s006.pdf]

Cox Regression

Case Processing Summary

		N	Percent
Cases available in analysis	Event[a]	7	9,3%
	Censored	68	90,7%
	Total	75	100,0%
Cases dropped	Cases with missing values	0	0,0%
	Cases with negative time	0	0,0%
	Censored cases before the earliest event in a stratum	0	0,0%
	Total	0	0,0%
Total		75	100,0%

a. Dependent Variable: Time_to_death_or_reinfection_30days

Categorical Variable Codings[a,c,d,e,f]

		Frequency	(1)
Sex[b]	,0	32	0
	1,0	43	1
Metastatic_solid_tumor[b]	0	73	0
	6	2	1
Lymphoma[b]	0	73	0
	2	2	1
Betalactam_betalactamase_inhibitor[b]	,00	70	0
	1,00	5	1
Short_treatment[b]	,00	40	0
	1,00	35	1

a. Category variable: Sex

b. Indicator Parameter Coding

c. Category variable: Metastatic_solid_tumor

d. Category variable: Lymphoma

e. Category variable: Betalactam_betalactamase_inhibitor

f. Category variable: Short_treatment

Block 0: Beginning Block

Variables not in the Equation[a]

	Score	df	Sig.
Sex	,003	1	,954
Age	,281	1	,596
Charlson_index	,042	1	,838
Lymphoma	3,846	1	,050
Metastatic_solid_tumor	3,846	1	,050
Short_treatment	,038	1	,845
Betalactam_betalactamase_inhibitor	7,141	1	,008
PS	,003	1	,954

a. Residual Chi Square = 15,448 with 7 df Sig. = ,031

Block 1: Method = Forward Stepwise (Wald)

Omnibus Tests of Model Coefficients[c]

Step	-2 Log Likelihood	Overall (score)			Change From Previous Step			Change From Previous ...		Change ...
		Chi-square	df	Sig.	Chi-square	df	Sig.	Chi-square	df	Sig.
1[a]	56,106	7,141	1	,008	3,790	1	,052	3,790	1	,052
2[b]	53,668	11,422	2	,003	2,438	1	,118	6,228	2	,044

a. Variable(s) Entered at Step Number 1: Betalactam_betalactamase_inhibitor

b. Variable(s) Entered at Step Number 2: Lymphoma

c. Beginning Block Number 1. Method = Forward Stepwise (Wald)

Variables in the Equation

		B	S.E.	Wald	df	Sig.	Exp(B)	95,0% CI ...	
								Lower	Upper
Step 1	Betalactam_betalactamase_inhibitor	1,931	,839	5,291	1	,021	6,893	1,331	35,713
Step 2	Lymphoma	2,200	1,121	3,854	1	,050	9,029	1,004	81,226
	Betalactam_betalactamase_inhibitor	2,129	,869	6,004	1	,014	8,402	1,531	46,115

Variables not in the Equation[a,b]

		Score	df	Sig.
Step 1	Sex	,002	1	,967
	Age	,934	1	,334
	Charlson_index	,006	1	,937
	Lymphoma	5,680	1	,017
	Metastatic_solid_tumor	5,680	1	,017
	Short_treatment	,007	1	,935
	PS	,002	1	,967
Step 2	Sex	,017	1	,897
	Age	,646	1	,421
	Charlson_index	,265	1	,607
	Metastatic_solid_tumor	,770	1	,380
	Short_treatment	,202	1	,653
	PS	,017	1	,897

a. Residual Chi Square = 10,843 with 6 df Sig. = ,093

b. Residual Chi Square = 4,578 with 5 df Sig. = ,470

Covariate Means

	Mean
Sex	,573
Age	74,307
Charlson_index	2,693
Lymphoma	,027
Metastatic_solid_tumor	,027
Short_treatment	,467
Betalactam_betalactamase_inhibitor	,067
PS	,467
